# Properties of Bisdiazo Compounds and Their Derived Carbenes via Density Functional Theory

**DOI:** 10.1002/cphc.202500438

**Published:** 2025-12-05

**Authors:** Xiaosong Liu, Mark Gerard Moloney

**Affiliations:** ^1^ Oxford Suzhou Center for Advanced Research 388 Ruoshui Road, Suzhou Industrial Park Suzhou Jiangsu 215123 China; ^2^ Department of Chemistry Oxford University Oxford 215123 UK

**Keywords:** biscarbene, bisdiazo, density functional theory, electronic structures, physisorption

## Abstract

To better understand the properties of carbene and biscarbene species derived from bisdiazo compounds with varied terminal groups, a density functional theory (DFT) study was conducted on bisdiazo compounds with four terminal groups (bisdiazo‐X, where X=H, Me, NO_2_ and NH_2_) and their mono‐ and dicarbene derivatives. The studies included computation of their frontier molecular orbitals (FMOs), electronic structures, electrostatic potential (ESP) and polarity, as well as their IR and UV‐vis spectra and their color in THF solutions. For bisdiazo compounds at both ground and excited states, the computational results matched well with published experimental data. The formation of carbene species from bisdiazo compounds was confirmed via a generalized IRC path calculation and IGMH analysis. The reaction sites and the lone pair electron locations were predicted using minimum ESP (i.e., ESPmin) and orbital‐weighted Fukui dual descriptor for the possible intermediates in the transition state, along with spin density analysis through EPR/ESR predictions. Additionally, physisorption of bisdiazo and carbene species onto single‐layer graphene was evaluated through geometry optimization, in which π‐π stacking among the aromatic‐ring likely determines surface packing via the simulated scanning tunnelling microscope (STM) images. The carbene species permit controlled growth of the patterned functional organic surfaces.

## Graphical abstract

Density function theory (DFT) calculation at level of B3LYP‐(D3)BJ/6‐311G** reveals the fundamental electronic structure of substituted bisdiazo compounds as well as their derived carbene and biscarbene species at both ground and excited states. Extension to physisorption onto single‐layer graphene provides more insight of the surface behaviour of this kind of bisdiazo and its carbene species.

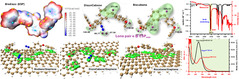

## 1 Introduction

Despite their inherent instability, exothermic decomposition,^[^ ^1^ ^]^ and potential explosive behavior upon heating or UV activation,^[^ ^2^ ^]^ conditions that lead to nitrogen gas release and the formation of carbene or biscarbene intermediates, diazo, and bisdiazo compounds have found widespread applications in polymer surface modification,^[^ ^3^, ^4^ ^]^ carbene insertion,^[^ ^5^, ^6^ ^]^ and coupling reactions.^[^ ^7^, ^8^ ^]^ Diaryldiazo compounds, which are typically oils or low‐melting solids,^[^ ^9^, ^10^ ^]^ are stable indefinitely when stored at low temperatures but decompose exothermally upon heating.^[^ ^11^ ^]^ Notably, bis(diaryldiazo) compounds exhibit greater stability over simple carbenes, are easier to handle, and remain highly reactive under thermal conditions, making them particularly useful for polymer surface modification reactions.^[^ ^12^ ^]^ The thermal and photochemical instability of diazo and bisdiazo compounds is the key to their utility, as it enables the in situ generation of reactive carbene and biscarbene intermediates that can readily interact with other compounds and polymeric substrates.

While diazo and bisdiazo compounds have been primarily used in academic laboratories,^[^ ^13^, ^14^ ^]^ recent efforts have begun to explore their commercial potential.^[^ ^15^ ^]^ To broaden their practical applications, a deeper understanding of their electronic properties is desired, and particularly the influence of terminal groups. Previous density functional theory (DFT) studies have explored various aspects of diazo reactivity, including carbene generation via four‐electron reduction mediated by metal complexes,^[^ ^16^ ^]^ the use of triarylboranes in stoichiometric and catalytic transformations,^[^ ^17^ ^]^ and the role of donor–acceptor interactions in modulating catalyst efficiency of B(C_6_F_5_)_3_,^[^ ^18^ ^]^ catalytic cyclopropanation reactions,^[^ ^19^ ^]^ concerted Wolff rearrangements,^[^ ^20^ ^]^ and metal carbene or metal‐ligated free carbene and subsequent carbene transformation pathways using *N*‐heterocyclic carbenes (NHC) as ligands.^[^ ^21^ ^]^ Mechanistic investigations have also been conducted on diazo‐ induced carbene formation, such as copper(I)‐catalyzed cross‐coupling reaction,^[^ ^22^ ^]^ acid‐catalyzed formal carbene transfer from diazo compounds to nucleophilic substrates,^[^ ^23^ ^]^ Pd‐catalyzed disilylation of carbene to construct disilylmethane derivatives,^[^ ^24^ ^]^ and the photochemical decomposition of diazo and diazirine compounds.^[^ ^25^ ^]^ In this study, we report a comprehensive DFT investigation of four bisdiazo compounds, designated as bisdiazo‐X, where X represents electronically neutral (X = H, Me), electron‐donating (X = NH_2_), and electron‐withdrawing (X = NO_2_) substituents. Building on literature experimental data, these model systems were selected to systematically analyze their electronic structures and reactivity.

## 2 Computational Details

DFT calculations were performed using GaussView 6.0^[^ ^26^ ^]^ and the Gaussian 16 program suite.^[^ ^27^ ^]^ Geometry optimization and frequency analyses were carried out using the popular hybrid functional B3LYP method (Becke's three‐parameter)^[^ ^28^ ^]^ with the correlation functional of Lee, Yang, and Parr^[^ ^29^ ^]^ in conjunction with 6 – 311 + G(d,p) basis set^[^ ^30^ ^]^ with dispersion correction using the schemes of Grimme GD3BJ. The structures of all the reactants, reaction intermediates, and products involved in the reaction process were fully optimized. All stationary points were confirmed as true minima by the absence of imaginary frequencies. The optimized structures were subsequently used as inputs for UV–vis and NMR spectral calculations in various solvents. These calculations employed the long‐range‐corrected functional CAM‐B3LYP^[^ ^31^ ^]^ with the basis set of 6 – 311 + G(d,p) and dispersion correction of Grimme type scheme GD3BJ^[^ ^32^, ^33^ ^]^ along with the implicit solvation model density (SMD), a widely used continuum solvation model in computational chemistry.

To model the physisorption of bisdiazo compounds and their derived species (diazo‐carbene and biscarbene) onto a single‐layer graphene surface, geometry optimization was performed using the free software program ORCA version 5.0.3.^[^ ^34^, ^35^ ^]^ The r^2^SCAN‐3c^[^ ^36^ ^]^ theory level for composite electronic structure method was applied, which is well‐suited for efficient and accurate optimization of composites,^[^ ^37^ ^]^ utilizing the default speed‐up algorithm.^[^ ^38^, ^39^, ^40^, ^41^ ^]^

For postprocessing of electronic wavefunctions, checkpoint files (.chk) generated from Gaussian calculations (used for structure optimization and frequency calculation) were converted to formatted checkpoint files (.fchk) using the formchk utility. These files (.fchk) were then analyzed using Multiwfn 3.8(dev),^[^ ^42^ ^]^ a multifunctional wavefunction analyzer, to extract electronic structure data and generate grid‐based outputs. The resulting data were further rendered by VMD 1.9.3 software^[^ ^43^ ^]^ for isosurface mapping.

**Scheme 1:**
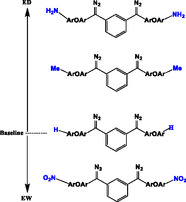
Structure and electronic order of bisdiazo‐X compounds with different terminal groups (X = H, Me, NO_2_, and NH_2_).

Bisdiazo compounds bearing various terminal groups have been synthesized and applied in surface modification,^[^ ^3^, ^4^, ^9^, ^12^ ^]^ as have alternative NHCs.^[^ ^44^, ^45^, ^46^, ^47^ ^]^ The general structure and electronic order of these bisdiazo derivatives are illustrated in **Scheme** **1** with detailed structures in Scheme S1, Supporting Information. Synthetic procedures and full characterization data are available in our latest work.^[^ ^48^ ^]^

## 3 Results and Discussion

### 3.1 Frontier Molecular Orbital and Surface Potential Analysis

**Figure 1:**
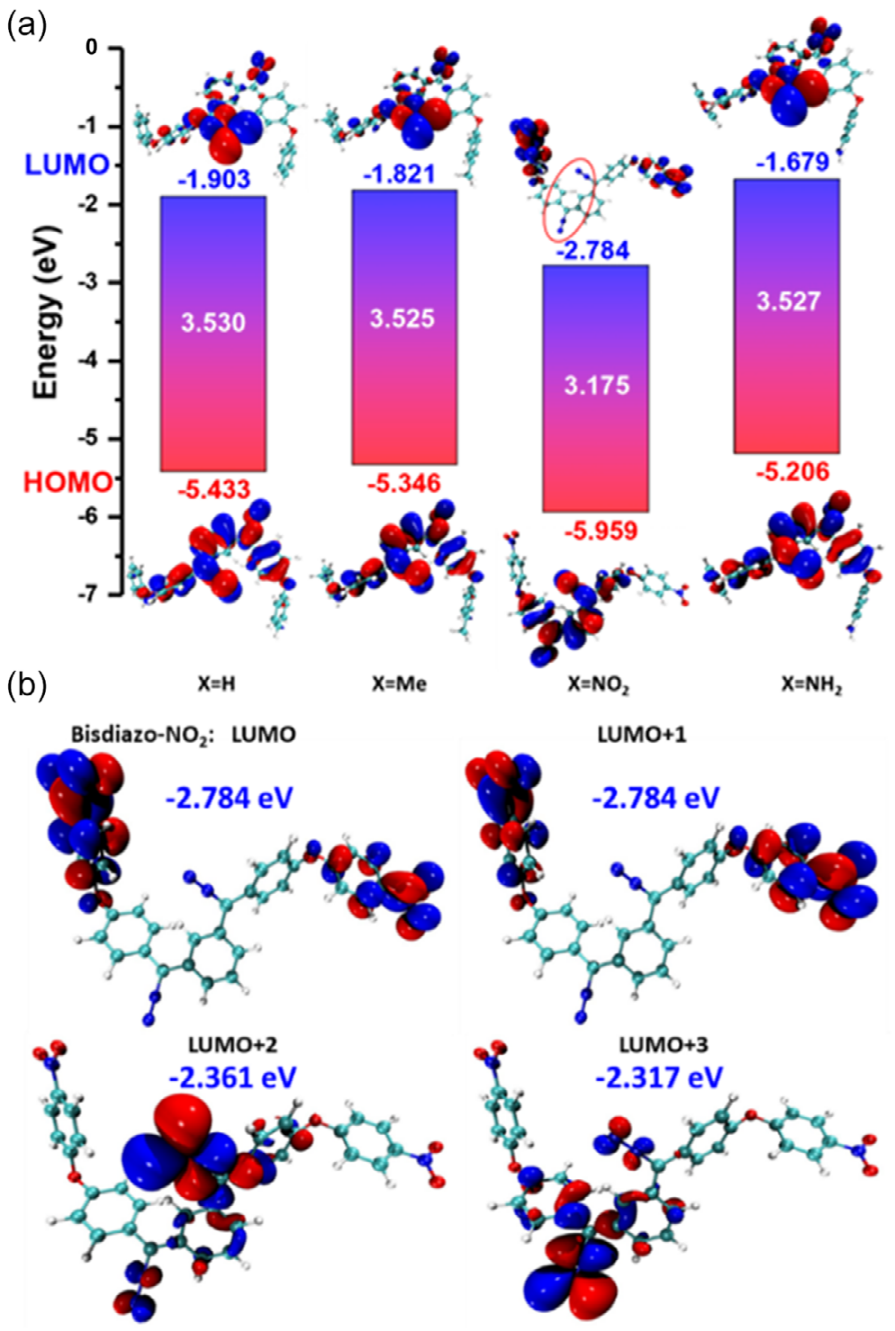
a) Frontier MO analysis of bisdiazo‐X compound (white numbers are the HOMO–LUMO gap energy in eV unit). b) Extra LUMO orbitals and energy level of bisdiazo‐NO_2_.

Frontier molecular orbitals (FMOs) play a vital role in describing and predicting molecular properties, particularly chemical reactivity. They are widely employed to interpret reactivity patterns and regioselectivity across various chemical systems^[^ ^49^, ^50^, ^51^, ^52^, ^53^, ^54^ ^]^ and are fundamental to understanding phenomena in both biological and materials science contexts. The energy gap between the highest occupied molecular orbital (HOMO) and the lowest unoccupied molecular orbital (LUMO), as calculated by DFT, is a key indicator of molecular stability and chemical behavior.^[^ ^49^ ^]^ A smaller HUMO–LUMO gap often correlates with increased activity, while a larger gap suggests great chemical inertness^[^ ^55^ ^]^ and stability.^[^ ^56^ ^]^ **Figure** **1**a illustrates the HOMO and LUMO distribution for the bisdiazo series of compounds. The hydrogen (—H) and methyl (—Me) terminated compounds exhibit nearly identical orbital distributions, whereas the amine (—NH_2_) terminated compound shows some deviation. Notably, the nitro (–NO_2_) terminated compound displays a distinct LUMO, with significant electron density localized on the nitro groups (highlighted in red), rather than on the diazo sites. Further analysis of the LUMO+1 to LUMO+3 orbitals for bisdiazo‐NO_2_ compound (Figure 1b) reveals continued electron density localization on the nitro groups. The relatively larger HOMO–LUMO gap in bisdiazo‐NO_2_ suggests enhanced chemical inertness compared to the other derivatives, which exhibit smaller gaps and thus greater reactivity. These orbital characteristics are reflected in their broader molecular properties (see below).

For larger systems, the orbital delocalization index (ODI), defined on a scale from 0 to 100,^[^ ^57^, ^58^, ^59^, ^60^, ^61^, ^62^ ^]^ provides a more nuanced measure of electron distribution. A lower ODI value indicates greater orbital delocalization. **Table** **1** lists the ODI values for the bisdiazo‐X compounds, calculated using both Mulliken and Hirshfeld methods. The ODI values for the HOMO and LUMO orbitals of the X = H and X = Me terminated compounds are very close, consistent with their similar orbital distribution (Figure 1a). In contrast, the LUMO of the bisdiazo‐NO_2_ shows a significantly lower ODI value compared to its LUMO+2 and LUMO+3 orbitals, reinforcing the localized nature of the LUMO as observed in Figure 1b.

**Table 1:** ODI.

X	Orbital	ODI	
		Mulliken	Hirshfeld
H	HOMO	7.91	6.07
	LUMO	34.16	29.60
Me	HOMO	7.87	6.03
	LUMO	32.02	27.74
NH_2_	HOMO	8.07	6.15
	LUMO	28.33	24.53
NO_2_	HOMO	8.89	6.72
	LUMO	10.25	9.18
	LUMO+1	10.33	9.25
	LUMO+2	35.53	30.88
	LUMO+3	35.37	30.89

**Figure 2:**
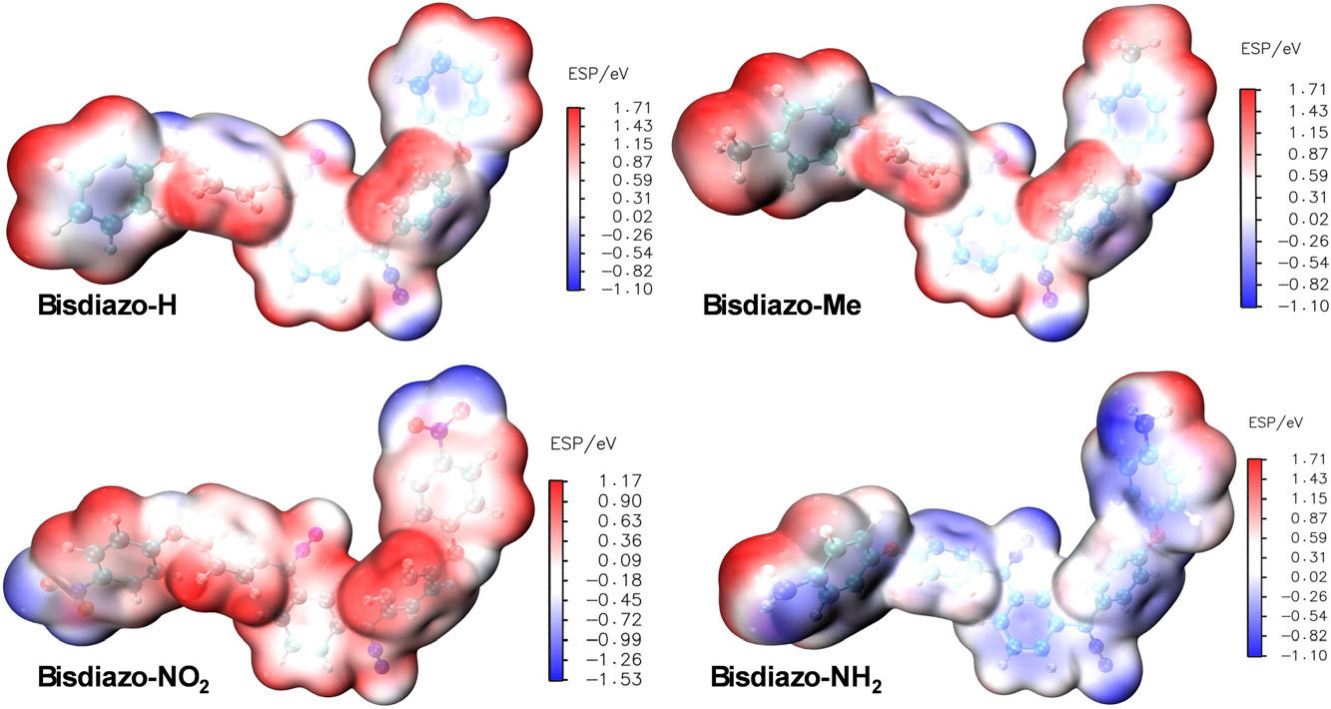
Molecular ESP of bisdiazo‐X compounds with varied terminal groups.

In addition to FMO analysis, molecular electrostatic potential (ESP)^[^ ^63^ ^]^ mapping is a valuable tool for predicting chemical reactivity,^[^ ^64^ ^]^ reaction processes,^[^ ^65^ ^]^ physical absorption, and intermolecular interactive behavior.^[^ ^66^ ^]^ **Figure** **2** displays the ESP distribution for various bisdiazo compounds. As with the FMOs, the ESP profiles of X = H and X = Me are nearly indistinguishable. However, marked differences are observed between the cases X = NO_2_ and X = NH_2_. For X = NO_2_, the most negative (or minimum) ESP regions are concentrated around the nitro group and, to a lesser extent, the diazo sites. For X = NH_2_, the ESP minima are located around diazo sites and the aromatic rings adjacent to the amine substituents.

### 3.2 Molecular Polarity Analysis

#### 3.2.1 Dipole Moment

Dipole moment can be accurately determined through quantum computational chemistry calculations. However, the dipole moment is heavily sensitive to molecular conformation, particularly in larger macromolecules. The calculated dipole moment magnitudes for the bisdiazo‐X compounds, both in gas phase and in different solvents, are summarized in **Table** **2** and illustrated in Figure S1, Supporting Information. A consistent trend is observed across all systems, with solvent effects playing a significant role in modulating electrophile and nucleophile interactions.^[^ ^67^, ^68^ ^]^

**Table 2:** Dipole moment magnitude (unit: Debye) of bisdiazo‐X in gas phase and different solvents.

X	Gas Phase	THF	Toluene	Chlorobenzene
H	2.8390	3.5395	3.2056	3.4708
Me	4.0374	5.6261	5.2735	5.6512
NO_2_	5.6479	6.4445	6.1275	6.3995
NH_2_	6.4318	7.7317	7.0726	7.6259

#### 3.2.2 Molecular Polarity Index (MPI)

The molecular polarity index (MPI), proposed by Tian Lu et al.^[^ ^62^, ^69^ ^]^ stems from the uneven charge distribution within molecules and is evident in the ESP distribution on the molecular surface. This index serves as a means to assess molecule polarity. A more uneven charge distribution results in a more pronounced positive or negative ESP on the molecule surface, thereby yielding a higher MPI. The MPI values for various compounds were determined following geometry optimization and frequency analysis in the gas phase. As indicated in **Table** **3**, bisdiazo‐NO_2_ is the only compound with moderate polarity, while the others exhibit only weak polarity. Regarding the contributions of nonpolar (SANP) and polar surface area (SAP), the X = H and X = Me compounds display a consistent pattern, with nonpolarity being dominant in both the gas phase and solvents. Conversely, X = NO_2_ shows the reverse trend. Notably, for X = NH_2_, the contributions of both non‐polar and polar surface area are influenced by solvents. Specifically, X = NH_2_ is significantly more nonpolar than polar in the gas phase, but becomes nearly balanced in solvents.

**Table 3:** Calculated MPI of bisdiazo‐X in gas phase and different solvents.

X	Gas Phase			THF			Toluene			Chlorobenzenea)		
	*MPI*	*SANP*	*SAP*	*MPI*	*SANP*	*SAP*	*MPI*	*SANP*	*SAP*	*MPI*	*SANP*	*SAP*
H	7.64	67.61	32.39	9.09	56.64	43.36	8.47	61.16	38.84	8.98	57.34	42.66
Me	7.59	69.05	30.95	9.12	57.69	42.31	8.55	61.52	34.48	9.04	58.20	41.80
NO_2_	12.65	44.48	55.52	14.97	37.46	62.54	13.92	40.33	59.67	14.79	37.95	62.05
NH_2_	9.65	59.90	40.10	11.48	49.10	50.91	10.69	52.99	47.01	11.35	49.65	50.35

a) Polarity classification by MPI (kcal mol^−1^): strong: >15 kcal mol^−1^, moderate: 8–15 kcal mol^−1^, weak: 4–8 kcal mol^−1^, and non‐polar: <4 kcal mol^−1^. SANP and SAP are for the nonpolar (NP) and polar (P) surface area (SA) with |ESP| ≤ 10 kcal mol^−1^ in percentage (%).

### 3.3 Electronic Structure Analysis of Ground State

#### 3.3.1 Electron Localization Function (ELF) Analysis

**Figure 3:**
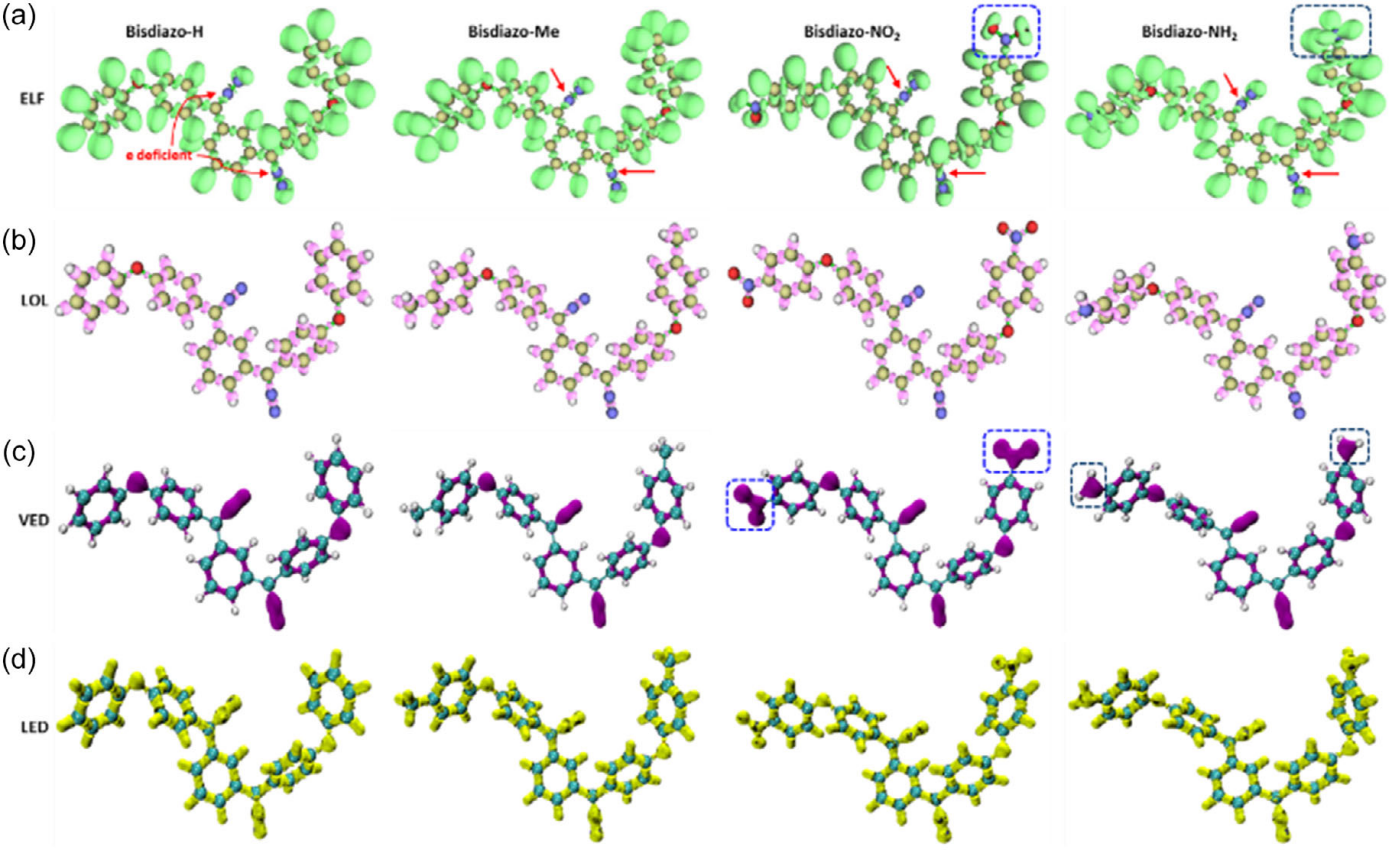
Isosurface plots of bisdiazo‐X compounds: a) ELF (red arrow points at the most electron‐deficient atom, N^−^ in the diazo site; blue dashed line boxed the outstanding ELF signatures in both NO_2_‐ and NH_2_‐terminated compounds), b) LOL, c) VED (blue dashed line boxed the most valence electron enriched groups in both NO_2_‐ and NH_2_‐terminated compounds), and d) LED (isovalue: 0.84 a.u. for ELF, 0.625 a.u. for LOL, 0.275 a.u. for VED, and −0.250 a.u. for LED).

The electron localization function (ELF) is a simple measure of assessing electron localization in atomic and molecular systems.^[^ ^70^ ^]^ It quantifies the likelihood of finding a second electron with the same spin in the vicinity of a reference electron. A lower probability indicates higher electron localization, reflected by a higher ELF value. **Figure** **3**a presents ELF isoplots of bisdiazo‐X compounds, with the red arrow highlighting electron‐deficient nitrogen atoms at the diazo sites. A similar electron deficiency is observed at the nitrogen atoms of the nitro group in bisdiazo‐NO_2_ (see boxed area in ELF), accompanied by pronounced electron density lobes around the oxygen atoms. In contrast, this deficiency is less noticeable in the nitrogen atoms of bisdiazo‐NH_2_ compound (indicated by the blue dashed line in the boxed area).

#### 3.3.2 Localized Orbital Locator (LOL) Analysis

The localized orbital locator (LOL) is used to identify regions of electron depletion and electron richness,^[^ ^42^, ^71^ ^]^ where electrons are highly localized.^[^ ^71^ ^]^ A higher LOL value indicates areas of electron localization. As shown in Figure 3b, regions of high electron depletion in LOL isoplots are primarily located on the aromatic rings, the diazo sites (C=N^−^=N^+^), and the three terminal groups, with the exception of the nitro group in bisdiazo‐NO_2_.

#### 3.3.3 Valence Electron Density (VED) Analysis

Valence electrons, though only a fraction of the total electron count, are critical in determining chemical reactivity, bonding, and material functionality. Mapping valence electron density (VED) is therefore essential for understanding and controlling chemical reactions, and VED distribution has been successfully visualized in crystalline materials in real space^[^ ^72^, ^73^ ^]^ and even tracked over a multifemtosecond time span.^[^ ^74^ ^]^ As shown in Figure 3c, the VED isosurfaces reveal quite noticeable electron density around nitrogen and oxygen atoms, consistent with the presence of lone‐pair electrons. Notably, the VED distribution differs markedly between for bisdiazo‐NO_2_ and bisdiazo‐NH_2_, particularly in the regions highlighted by blue dashed line boxes.

#### 3.3.4 Laplacian of Electron Density (LED) Analysis

The Laplacian electron density (LED),^[^ ^75^ ^]^ denoted as ∇^2^ *ρ(r)*, provides insights into local electron concentration or depletion. A positive value (∇^2^ *ρ(r) > 0*) indicates electron depletion, while a negative value (∇^2^ *ρ(r) < 0*) suggests electron concentration, typically associated with covalent bonding. Figure 3d displays negative LED isosurfaces for bisdiazo‐X compounds, highlighting regions of concentrated electron density. These regions, particularly around carbon atoms at the diazo sites, are potential sites for nucleophilic attack.

#### 3.3.5 Molecular Planarity

**Figure 4:**
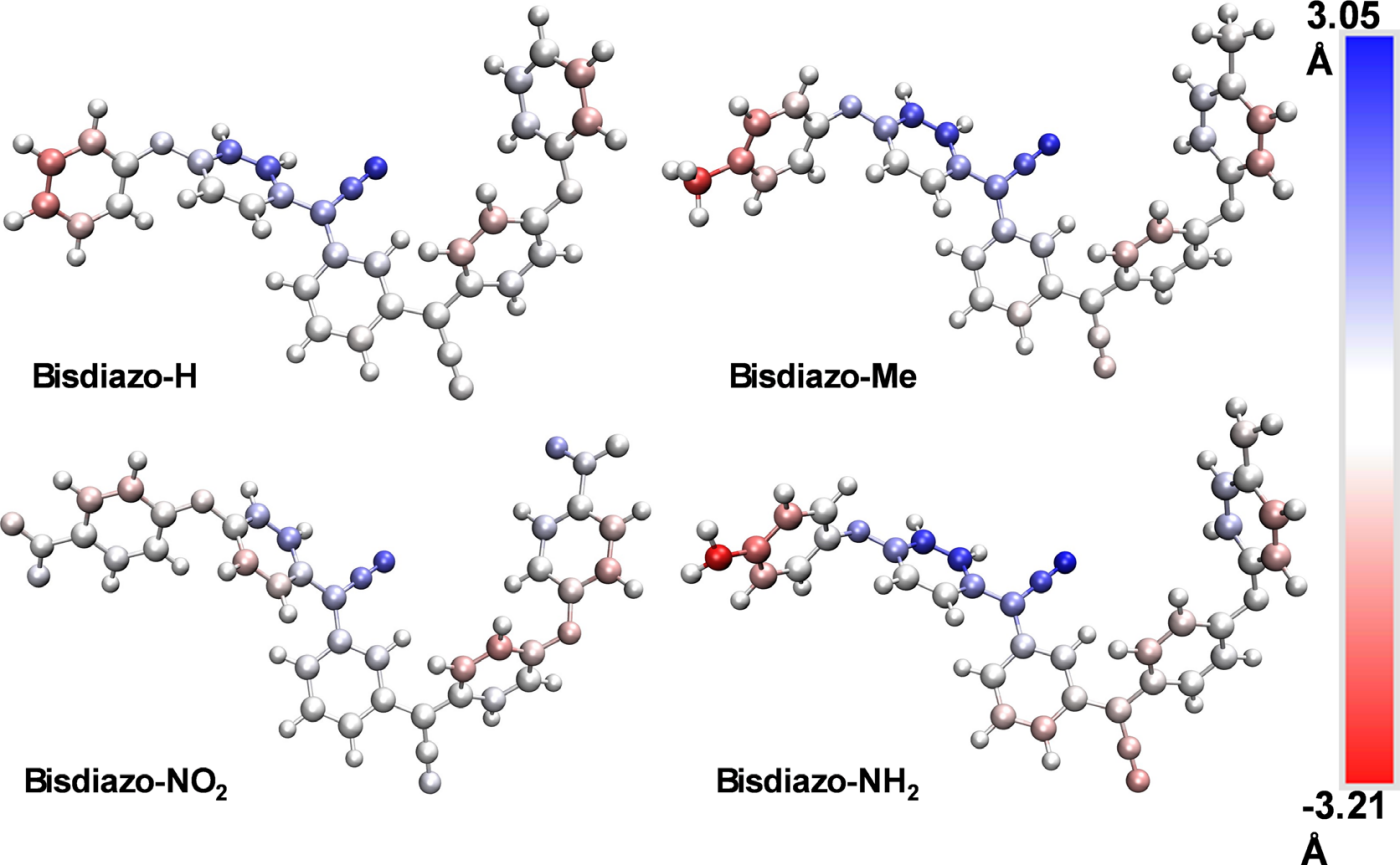
Molecular (non)‐planarity of bisdiazo‐X compounds in the gas phase. Atoms are colored according to the ds values of the signed distance to the fitting plane. The bluer (redder) the color, the larger the distance of the atom below (above) the fitting plane (color bar scale: −3.21 to 3.05 Å).

**Figure 5:**
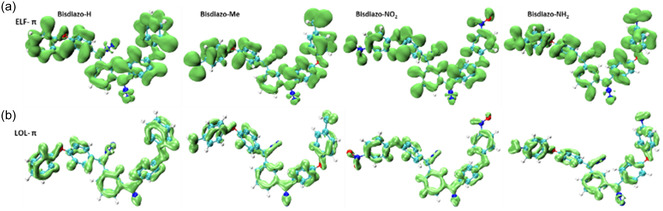
a) ELF–*π* and b) LOL–*π* electron distribution isosurface of bisdiazo‐X compounds. (isovalue: 0.75 a.u for ELF–*π* and 0.45 a.u. for LOL–*π*).

Molecular planarity significantly impacts the conjugation of *π* electrons in *π*‐conjugated systems, thereby influencing photophysical properties^[^ ^76^ ^]^ and aromaticity.^[^ ^77^, ^78^ ^]^ To quantify the planarity of bisdiazo‐X compounds, the molecular planarity parameter (MPP) and the span of the deviation from plane (SDP),^[^ ^69^ ^]^ proposed by Lu et al.^[^ ^38^ ^]^ were employed. MPP measures the root‐mean‐square deviation of atoms from the fitting plane, while SDP measures the maximum deviation. Lower values of MPP and SDP indicate higher planarity. The calculated MPP and SDP values for bisdiazo‐X compounds in the gas phase are presented in **Table** **4** and **Figure** **4**. Quantitatively, the planarity difference between bisdiazo‐H and bisdiazo‐Me is minimal, as indicated by MPP. Both MPP and SDP values reveal that the X = NO_2_ compound is the most planar, while the X = NH_2_ compound exhibits the least planarity. The nonplanar bisdiazo compounds display ELF–*π* and LOL–*π* electron distribution (**Figure** **5**), which will be elaborated in detail later. Table 4 and Figure 4 demonstrate that MPP and SDP effectively quantify the planarity of the bisdiazo compounds, aligning with the degree of deviation from planarity observed in molecular structure mappings. Figure 4 highlights the two atoms with the greatest deviation in bisdiazo‐NH_2_, corresponding to the SDP calculation. This indicates that the two diazo sites are on opposite sides of the fitted plane, particularly for bisdiazo–NH_2_. Consequently, all the bisdiazo compounds are nonplanar systems with diazo sites pointing in opposite directions in their minimum energy states. Additionally, a surface distance projection map^[^ ^79^, ^80^ ^]^ (Figure S2, Supporting Information) provides a semiquantitative visualization of the nonplanarity in the *X*–*Y* plane after geometry optimization and frequency calculations. These maps provide a graphical representation of the nonplanar nature of the compounds, as exhibited in Figure 4.

**Table 4:** Calculated MPP and SDP of bisdiazo‐X compounds.

X	MPP [Å]	SDP [Å]	Charge scale [Å]
H	1.004	4.452	−1.84‐2.61
Me	1.164	4.452	−2.78‐2.81
NO_2_	0.784	3.622	−1.44‐2.19
NH_2_	1.303	6.254	−3.21‐3.05

#### 3.3.6 ELF–π and LOL–π Electron Distribution

For nonplanar bisdiazo compounds (see Table 4 and Figure 4), MOs consist of both *σ* and *π* contributions, which can be separated using localized MO theory. This separation is particularly useful for analyzing *π*‐electron distribution in the aromatic systems, even when adjacent aromatic rings are twisted due to nonplanar geometry. By combining ELF and LOL analyses, ELF–*π* and LOL–*π* distributions have been successfully applied to nonplanar *π*‐system^[^ ^81^, ^82^ ^]^ such as cycloacenes, short carbon nanotubes, and C20 fullerenes.^[^ ^83^ ^]^ As illustrated in Figure 5, a similar analysis for bisdiazo‐X compounds shows that the majority of *π* electron density, both in ELF–*π* and LOL–*π* electrons, is localized within the aromatic rings (Figure 5b).

#### 3.3.7 Intramolecular Interactions Analysis

**Figure 6:**
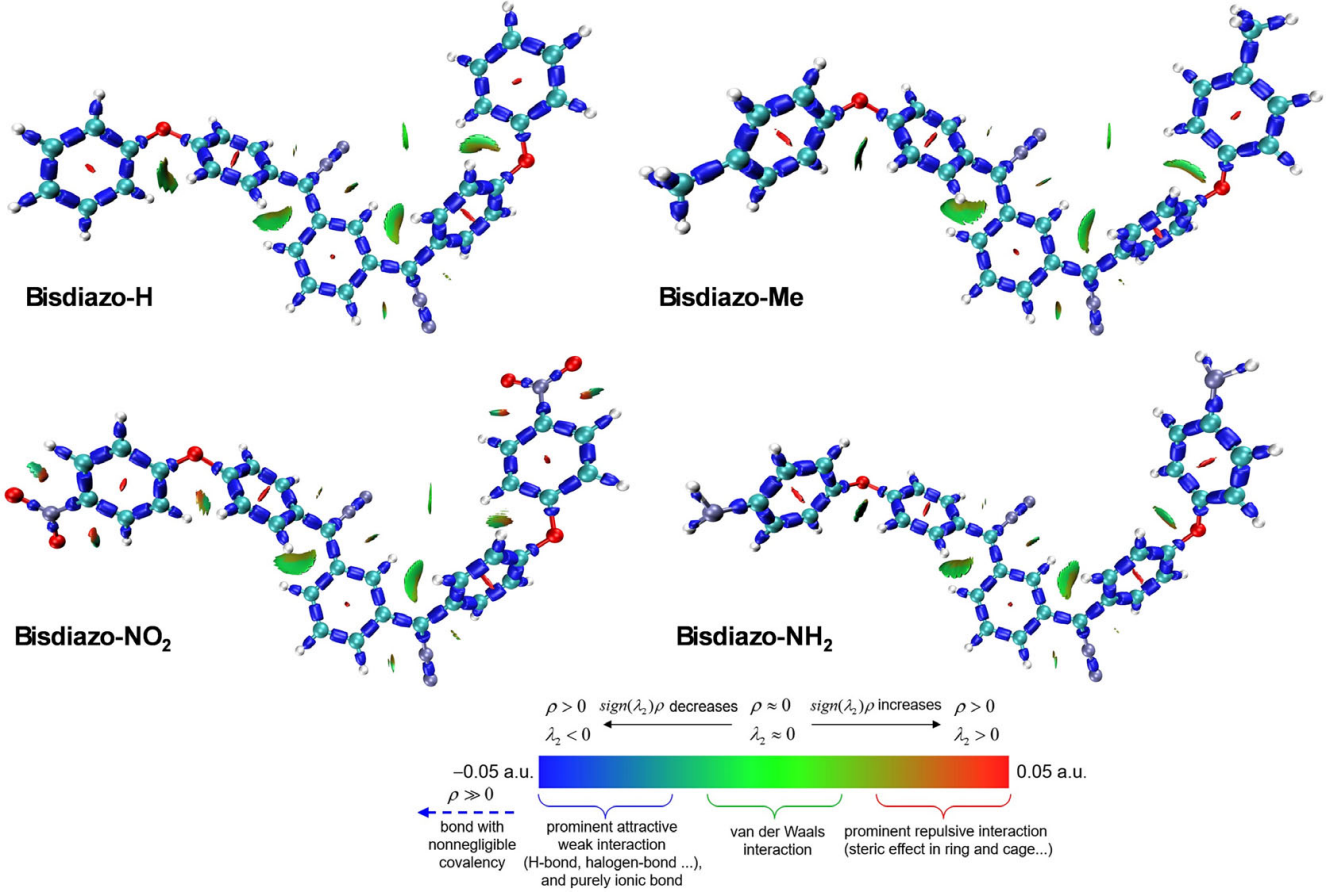
IRI isosurface maps of bisdiazo‐X compounds with varied terminal groups (IRI = 1.00, sign*(λ*_2_ *)ρ* is mapped on the isosurface for differentiating the interactions).

**Figure 7:**
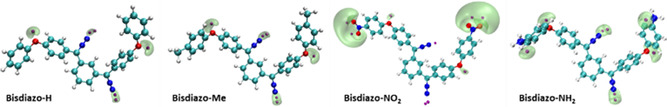
Possible locations of lone pair electrons in bisdiazo‐X compounds. (isosurface is plotted at the isovalue of −0.0275 a.u. = −72.20 kJ mol^−1^, and the purple beads locate the very min values of ESP region of localization of electron density in the lone pair. Lime isosurface is for the structural features and chemical environment (strength of the lone pair).

The interaction region indicator (IRI)^[^ ^84^ ^]^ is a new real‐space function derived from a minor modification of the reduced density gradient (RDG). It is particularly effective for identifying chemical bonding and weak interaction regions. The IRI analysis of bisdiazo‐X compounds with varied terminal groups is portrayed in **Figure** **6**, where blue barrels represent covalent bonds within the molecules, the red areas at the centers of aromatic rings indicate the steric effect due to the notable repulsion, while the irregular green flakes signify van del Waals interaction as well as hydrogen bonds. Notably, Figure 6 shows that the IRI region around the NO_2_ group and its adjacent aromatic ring is unique and not observed in the Me‐ or NH_2_‐termined systems. Such observation further underscores the distinctive features of bisdiazo‐NO_2_ compound, previously identified through the frontier MOs (Figure 1b), ESP (Figure 2), ELF, and VED (Figure 3a,c), as well as *π*‐electron (Figure 5), especially lone pair electron distribution (**Figure** **7**).

### 3.4 Reaction Site Prediction

#### 3.4.1 Quantum Theory of Atoms in Molecules Analysis

The locations of lone pair regions associated with negative ESP minima can be identified through a combined approach involving *ESP*_min_ and topological analysis based on Bader's atoms‐in‐molecules (AIM) theory. This method enables the localization of critical points that correspond to lone pairs, *π*‐bonds, and *π*‐electrons.^[^ ^85^ ^]^ The resulting distribution patterns for H‐ and Me‐terminated bisdiazo compounds are similar. However, in the NO_2_‐terminated compound, the most negative ESP regions are localized at the nitro group rather than the diazo sites (Figure 7). This observation aligns with earlier findings from FMO, ESP, and IRI analysis, further confirming that the bisdiazo‐NO_2_ compound exhibits distinct electronic behavior and therefore reactivity compared to the other derivatives.

#### 3.4.2 Conceptual Density Functional Theory (CDFT) Analysis

Conceptual DFT (CDFT) provides insights into the reactivity of chemical systems, predicting favorable reactive sites and character. The calculated results for bisdiazo compounds are summarized in **Table** **5**. Mulliken electronegativity (*χ*_*L*_) is defined for elements with high first ionization energies and very negative electron affinities (EAs). Conversely, elements with low first ionization energies have slightly negative or positive EAs and tend to lose electrons in reactions.

**Table 5:** CDFT features of bisdiazo‐X compounds in the gas phase under standard conditions.

X	*VIP* [eV]	*VEA* [eV]	*χ*_*L*_ [eV]	*μ* [eV]	*η* [eV]	*ω* [eV]	*N*_nu_ [eV]a)
H	6.5279	0.4068	3.4673	−3.4673	6.1210	0.9821	3.8064
Me	6.4361	0.3397	3.3879	−3.3879	6.0964	0.9414	3.8909
NO_2_	7.0875	1.4197	4.2536	−4.2536	5.6678	1.5961	3.2891
NH_2_	6.2663	0.1924	3.2293	−3.2293	6.0739	0.8585	4.0518

a) Note: VIP, VEA, Mulliken electronegativity *χ*_*L*_, chemical potential *μ* (= −*χ*_*L*_), hardness *η* (=fundamental gap), Softness *S* *=* *1/η*, electrophilicity index *ω*, nucleophilicity index *N*_nu_.

The vertical ionization potential (VIP) is crucial for understanding electronegativity, hardness, and softness, as well as the electronic structure and stability of molecules. The vertical electron affinity (VEA) measures a molecule's ability to accept one electron from a donor. The electrophilicity index (*ω)* is related to EA, as both *ω* and EA measure electron‐accepting capability. However, EA reflects the acceptance of one electron, while *ω* measures the energy lowering due to electron flow between donor and acceptor, which may be less or more than one electron. From the molecular structure and chemical intuition, X = H and X = Me terminated systems are similar based on VIP and VEA values as listed in Table 5, which are slightly higher than those of X‐NH_2_ but noticeably lower than those of X = NO_2_. Similar trends are observed in Mulliken electronegativity (*χ*_*L*_), hardness (*η*), and electrophilicity indices (*ω*). Thus, the X = NO_2_ compound is considered to be an electrophile, while the others are nucleophiles based on the electrophilicity index (*ω*). From the nucleophilicity index (*N*_nu_) perspective, the X = NH_2_ compound is much more likely to be nucleophilic.^[^ ^86^ ^]^ Lower VEA indicates a tendency to absorb electrons and high electron attachment capacity, which correlates with the LUMOs for the electron attachments. The VEA values listed in Table 5 also highlight the distinct nature of X = NO_2_ due to its unique terminal group, as shown in Scheme 1 and Figure 1 and 2.

#### 3.4.3 LEAE, ALIE, and LEA Analysis

**Figure 8:**
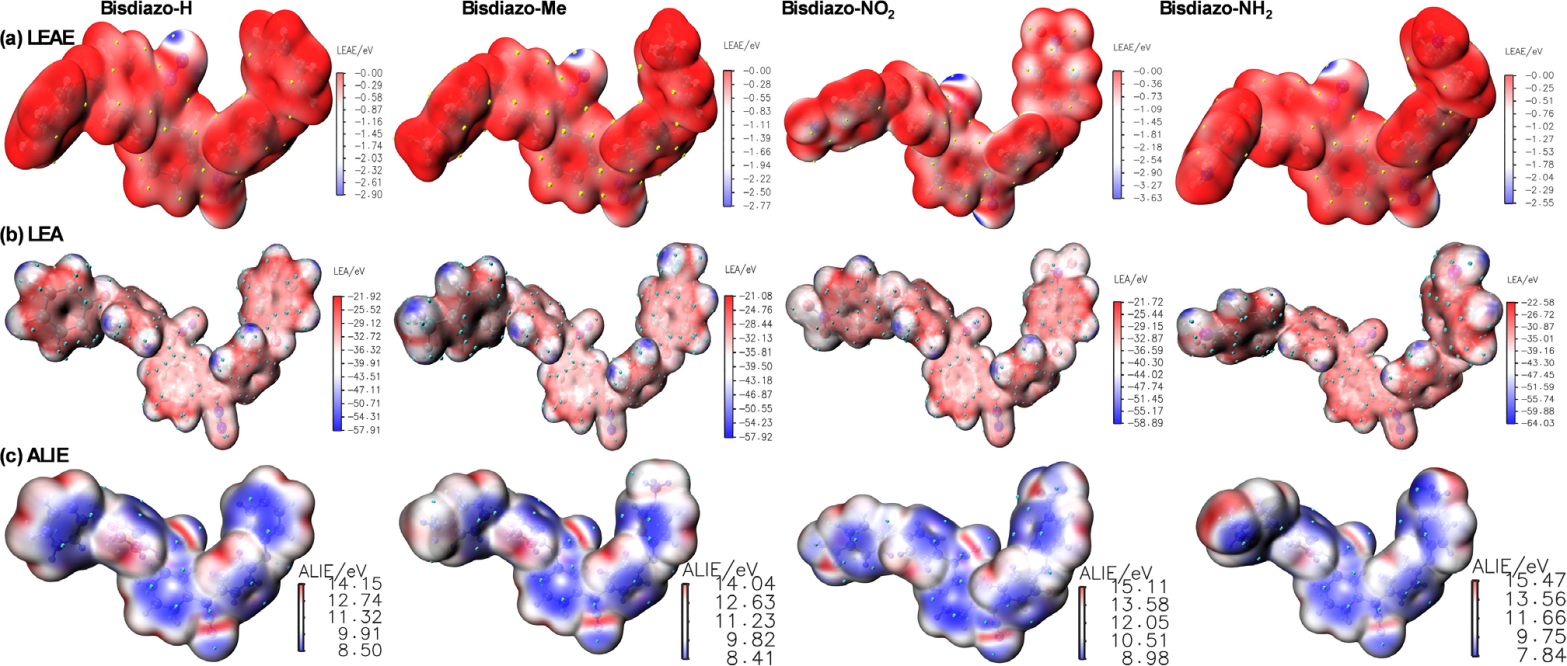
a) LEAE, b) LEA, and c) ALIE mappings on the vdW surface of bisdiazo‐X compounds. (Yellow spheres indicate the maximum LEAE on the surface, cyan spheres correspond to the maximum of LEA on this surface, the blue area is the low ALIE region, and the cyan spots are the minimum of ALIE and possible electrophilic reactive sites.).

Quantum chemistry offers theoretical insights into electron attraction and donation sites. Here, similar to the MO wavefunction and the average local ionization energy (ALIE), the local electron affinity (LEA)^[^ ^87^, ^88^ ^]^ is used to measure the local electrophilicity. These metrics help identify nucleophilic reaction sites, as shown in **Figure** **8**. Typically, ESP and ALIE are projected onto an isosurface with an electron density of 0.001 a.u., while LEAE mapping is recommended for a 0.004 a.u. isosurface^[^ ^88^ ^]^ for better visualization. More negative LEAE spots indicate unoccupied orbitals with negative energy levels, making the diazo site regions more electrophilic and likely to react with nucleophiles for all the bisdiazo‐X compounds, as shown in Figure 8a. The more negative the LEAE, the higher the priority for nucleophilic attack. For similar molecules, the more negative the LEAE regions on the molecular surface are more reactive and have a higher rate constant for nucleophilic reaction. Quantitative molecular surface analysis can identify the minimum LEAE values on the molecular surface,^[^ ^89^ ^]^ aiding in electrophilicity assessment of specific local areas like chemical bonds and *σ*‐ and *π*‐holes. Furthermore, LEAE is more reliable than LEA due to the latter's sensitivity to basis sets and especially diffuse functions. LEAE depends only on unoccupied orbitals with energy less than zero. LEA describes the acceptor properties of the molecule, with positive affinity sites on the aromatic (Figure 8b), but can also be combined with ALIE to provide local Mulliken electronegativity (*χ*_L_) and local hardness (*η*_L_) as summarized in Table 5.

ALIE (Figure 8c) is commonly used to determine electron ionization at a specific location in 3D space.^[^ ^90^ ^]^ Smaller ALIE values indicate weaker electron binding and a higher likelihood of electrophilic reaction. ALIE also reflects the localized nucleophilicity at certain atoms, predicting and explaining the rate and regioselectivity of electrophilic reaction sites. Minimum ALIE values are typically found around weakly bound electrons, such as lone pair electrons. For instance, in bisdiazo‐NO_2_, the nitrogen atom in the nitro group possesses the maximum ALIE, while in bisdiazo‐NH_2_, the nitrogen atom has the minimum ALIE, consistent with donating (X = NH_2_) and withdrawing (X = NO_2_) groups as illustrated in Scheme 1.

Overall, LEA complements ALIE by providing an electron‐acceptor perspective. The strongest electron‐accepting capacity is indicated by the most positive (or least negative) LEA values. LEA is crucial in quantitative structure–property relationship models^[^ ^91^, ^92^, ^93^, ^94^, ^95^ ^]^ and predictions of biological activity.^[^ ^96^ ^]^ In these applications, ALIE and LEA often play a more significant role than ESP, likely due to solvation effects that shield electrostatic interactions.

### 3.5 Spectra and Excited States Analysis

#### 3.5.1 Infrared (IR) Spectra

**Figure 9:**
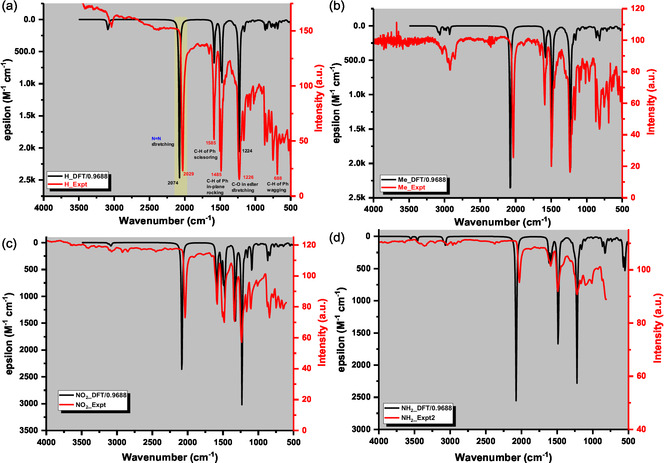
Comparison of calculated DFT and experimental IR of bisdiazo‐X compounds in THF: a) X = H, b) X = Me, c) X = NO_2_, d) X = NH_2_.

The calculated IR spectra were broadened using a scaling factor of 0.9688, appropriate for the theory level of B3LYP/6 – 311 + G(d, p),^[^ ^97^ ^]^ as shown in **Figure** **9**. Experimental spectra were collected in the range of 4000–600 cm^−1^ at a resolution of 4 cm^−1^ averaged over eight scans. The theoretical and experimental spectra matched quite well, with clearly identifiable vibrational modes corresponding to each spectral band. This is clearly exemplified by the IR spectrum of bisdiazo‐H in Figure 9a.

#### 3.5.2 Ultraviolet–Visible (UV–vis) Spectrum in Varied Solvents

**Figure 10:**
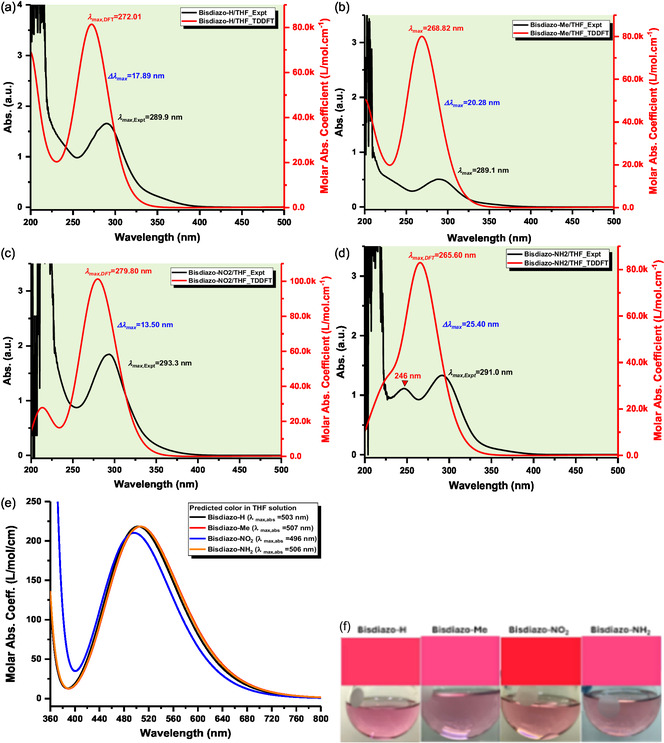
Comparison on UV–vis spectrum of bisdiazo‐X compounds in THF obtained by TDDFT calculation and experiment: a) X = H, b) X = Me, c) X = NO_2_, d) X = NH_2_, e) Calculated absorption plots for color prediction and f) predicted color tag and actual colored images of bisdiazo‐X solutions in THF (0.4 mg mL^−1^).

**Figure** **10** compares the theoretical and experimental UV–vis spectra of bisdiazo compounds in THF solutions. For bisdiazo‐H and bisdiazo‐Me, differences in the major absorption peaks between calculated and measured spectra are minor, with deviations of 17.89 and 20.28 nm, respectively. In the case of bisdiazo‐NO_2_, a minor measured absorption peak at ≈246 nm appears as a shoulder in the calculated UV–vis curve. Additional comparisons for bisdiazo‐NO_2_ and bisdiazo‐NH_2_ in THF are exhibited in Figure S3, Supporting Information. Overall, the Time‐Dependent Density‐Functional Theory (TDDFT) Independent gradient model based on Hirshfeld partition calculations, performed at the theoretical level of CAM‐B3LYP‐D3(BJ) along with a SMD solvation model, show good agreement with experimental data in the major UV–vis absorption peak.

To further validate the reliability of the TDDFT results, color prediction was performed, as demonstrated in Figure 10e. The spectral characteristics of bisdiazo compounds in varied solvents are summarized in **Table** **6**. Figure 10a–d and Table 6 imply that the visible range absorption peaks remain largely consistent across solvents. The predicted color tags (Figure 10f) closely match the observed actual colors at a concentration of 0.4 mg mL^−1^ in THF solution.

**Table 6:** Calculated maximum absorption peak (*λ*_max_) in UV–vis of bisdiazo‐X compounds in varied solvents.

Solvents	*λ*_max_ [nm]			
	X = H	X = Me	X = NO_2_	X = NH_2_
THF	272.01	268.82	279.80	265.60
Toluene	273.44	269.67	278.58	267.24
Chlorobenzene	273.18	269.56	280.38	266.54

### 3.6 Electronic Structures Analysis of Excited States

#### 3.6.1 Hole and Electron Delocalization Index Analysis

**Figure 11:**
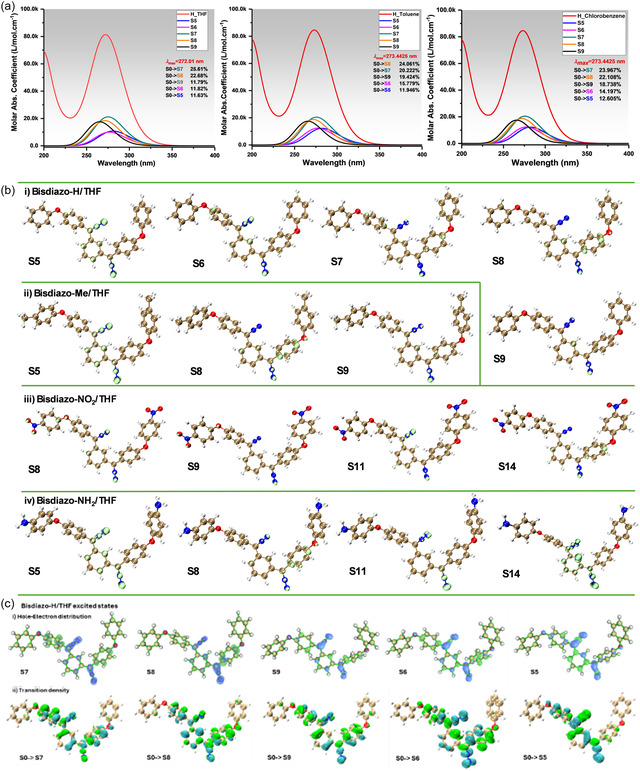
a) Calculated UV–vis spectrum and its major contributed excited states of bisdiazo‐H in varied solvents (THF, toluene, and chlorobenzene) via TDDFT calculation with SMD solvent model, b) Sr isoplots from hole and electron analysis of the excited states of varied bisdiazo compounds in THF as solvent (Sr lime plots of all the singlet excited states with the isovalue of 0.0035 a.u.), c) isosurfaces on hole–electron distribution (isovalue = 0.0025 a.u.), and transition density (isovalue = 0.001 a.u.) of bisdiazo‐H in THF.

The hole delocalization index (HDI) and electron delocalization index (EDI) indicate the degree of hole and electron delocalization, respectively. Lower values imply higher delocalization and a more uniform distribution. Increased delocalization spreads the positive charge of the hole, enhancing Coulomb attraction between the electron and hole, which in turn requires higher exciton binding energy.^[^ ^98^, ^99^, ^100^, ^101^, ^102^, ^103^, ^104^ ^]^ The calculated orbital transition contribution for electron excitations of bisdiazo compunds in THF solutions is listed in **Table** **7**. The *S*_r_ index quantifies the overlap between hole and electron distributions in the major excited states, as shown in the calculated UV–vis bands in **Figure** **11**a,b. Specifically in Table 7, the *D* index represents the total charge transfer length, measured by the distance between the centroid of the hole and the electron in corresponding directions (X/Y/Z). The *S*_r_ index characterizes the overlapping extent of hole and electron, the *H* index measures the spatial extent of hole and electron distribution in X/Y/Z, and the *t* index assesses the separation degree between hole and electron along the charge transfer direction.^[^ ^105^ ^]^ A positive *t* index indicates clear separation due to charge transfer, while a negative value suggests minimal separation. Thus, all the bisdiazo compounds show no significant separation (*t* < 0 in Table 7) because of the charge transfer, supported by the calculated *S*_r_ values ranging from 0.40 to 0.70, indicating substantial overlap of hole and electron distributions.

**Table 7:** Hole and electron delocalization index (HDI and EDI) analysis of bisdiazo compounds in THF.

*λ*_abs,max_	Bisdiazo‐H/THF					
Ex. Trans. [%]	*D* [Å]	*Sr* [a.u.]	*H* [Å]	*t* [Å]	*HDI*	*EDI*
S0‐>S7/25.61	1.503	0.53	4.657	−1.355	5.25	3.27
S0‐>S8/22.68	1.510	0.50	4.570	−1.080	5.35	3.37
S0‐>S9/11.79	1.313	0.44	4.832	−1.116	4.20	2.48
S0‐>S6/11.82	0.675	0.56	4.391	−1.685	4.94	3.29
S0‐>S5/11.63	0.908	0.67	4.107	−2.153	5.50	4.66

*λ*_abs,max_	Bisdiazo‐Me/THF					
Ex. Trans. [%]	*D* [Å]	*Sr* [a.u.]	*H* [Å]	*t* [Å]	*HDI*	*EDI*
S0‐>S5/34.25	0.774	0.64	3.808	−1.594	5.36	4.90
S0‐>S8/29.01	1.858	0.50	4.058	−0.517	6.14	3.98
S0‐>S9/16.18	1.246	0.48	4.749	−1.223	4.58	2.73

*λ*_abs,max_	Bisdiazo‐NO_2_/THF					
Ex. Trans. [%]	*D* [Å]	*Sr* [a.u.]	*H* [Å]	*t* [Å]	*HDI*	*EDI*
S0‐>S8/18.55	1.000	0.59	6.181	−4.148	4.60	4.99
S0‐>S9/16.02	2.139	0.50	6.884	−1.969	4.66	6.09
S0‐>S11/13.12	1.880	0.54	5.413	−1.528	5.20	4.01
S0‐>S14/12.31	1.308	0.56	5.549	−1.671	4.90	3.43

*λ*_abs,max_	Bisdiazo‐HN_2_/THF					
Ex. Trans. [%]	*D* [Å]	*Sr* [a.u.]	*H* [Å]	*t* [Å]	*HDI*	*EDI*
S0‐>S5/15.75	0.780	0.67	3.674	−1.352	5.76	5.41
S0‐>S8/29.72	1.459	0.53	4.203	−0.556	5.92	4.16
S0‐>S11/20.06	1.961	0.49	4.238	−0.434	5.25	6.29

Furthermore, isoplots of the transition density for bisdiazo compounds in various solvents are shown in Figure 11c, using the excited states of bisdiazo‐H in THF as an example to investigate electron excitation characteristics. These plots display hole–electron distribution and transition density between the ground state (GS) (*S*_0_) and major excited states (*S*_*i*_ *, i* > 0). The green and blue meshes represent positive and negative isosurfaces, indicating the distribution of electrons and holes at each excited states^[^ ^106^ ^]^ for bisdiazo‐H compound.

### 3.7 Transition State Confirmation and Analysis

#### 3.7.1 Transition State (TS) Analysis

**Figure 12:**
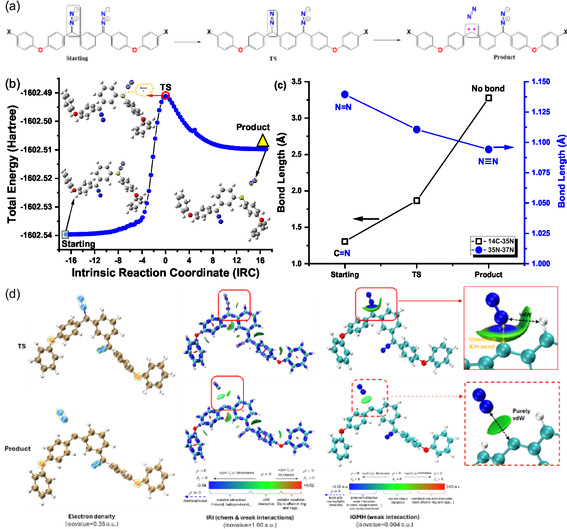
a) Scheme of producing diazo‐carbene and biscarbene from bisdiazo‐X compounds with varied terminal groups. b) IRC path plot of bisdiazo‐H confirming its TS, starting, and product (here the product is diazocarbene one plus nitrogen). c) Bond length varied at the same diazo‐carbene site of both TS and product from bisdiazo‐H. d) Electron density, IRI, and IGMH analysis of the corresponding TS, starting, and product from bisdiazo‐H compound. (Geometry optimization and IRC path were both obtained at theory level of B3LYP‐D3BJ/6 – 311 + G**, and further electron density, IRI, and IGMH analysis are on the wavefunction of single‐point energy calculation at theory level of M062X‐D3/6 – 311 + G**.)

Transition state searching is essential for elucidating reaction mechanisms and exploring reaction networks. Due to the complexity of potential energy surfaces (PESs), accurately determining transition state structures requires computationally intensive quantum chemistry calculations. These calculations yield the geometries of the reactant, transition state, and product involved in an elementary reaction. Studying carbene generation from bisdiazo compounds helps elucidate the unique reactions of carbenes.^[^ ^107^, ^108^, ^109^, ^110^, ^111^ ^]^ However, activation energy, a key property linking kinetics to electronic structure, requires both the reactant and the transition state geometries, typically obtained by following the PES gradient to a minimum or saddle point, respectively. As an example, the intrinsic reaction coordinate (IRC) path for bisdiazo‐H compound is shown in **Figure** **12**a, with the corresponding energy profile in Figure 12b. The IRC path connects a given transition structure to local minima of the reactant and products. Although Figure 12b confirms the starting material, product, and TS, the bond length in TS (Figure 12c) reflects no clear bond formation (here the atomic distance between two adjacent ones in the diazo site). Further analysis of structural differences is portrayed in Figure 12d using electronic features, such as electron density, IRI, and Independent gradient model based on Hirshfeld partition (IGMH). The IRI,^[^ ^84^ ^]^ a modified form of RDG, is a simple function that visually reveals all types of interactions in a chemical system, including both bonding and weak interactions. The independent gradient model (IGM) based on the Hirshfeld partition (IGMH)^[^ ^112^ ^]^ improves upon the IGM method, with the atomic densities derived by Hirshfeld partition.

Bond order concepts such as Laplacian bond order (LBO)^[^ ^113^ ^]^ and intrinsic bond strength index (IBSI), derived from the IGM formulation, are used to assess bond strength,^[^ ^114^ ^]^ with results listed in **Table** **8**. LBO, defined as a scaled integral of the negative Laplacian of electron density in fuzzy overlap space, correlates with bond polarity, dissociation energy, and vibrational frequency. Analysis of the transition state (TS) and product for bisdiazo‐H shows a clear difference in LBO, further strengthened by the FBO and IBSI, especially when comparing interatomic distances to known typical bond lengths. Moreover, the electronic density remains largely unchanged across atoms and is concentrated mostly on diazo sites and the oxygen atoms(Figure 12d, electron density). The IRI analysis shows a significant difference between TS and product, with IGMH highlighting variations in van dal Waals weak interactions (Figure 12d, IRI and IGMH). These semiquantitive visualizations confirm that the TS and product lie along the IRC path for the bisdiazo‐H compound.

**Table 8:** Indexes on the diazocarbene site 14C‐35 N‐37 N of starting material, TS, and product from bisdiazo‐H.

	Starting material		TS		Producta)	
	14C‐35 N	35 N‐37 N	14C‐35 N	35 N‐37 N	14C‐35 N	35 N‐37 N
**FBO**	1.4782	2.5354	0.6394	2.8933	0.0557	3.0967
LBO	1.0265	2.2169	N/Ac)	2.8425	N/Ac)	3.1570
**IBSI**	1.0103	2.0689	0.1840	2.3381	0.0065	2.4795
Dist.b)	1.3063	1.1395	1.8663	1.1106	3.2774	1.0942

a) Note: Fuzzy bond order (FBO). LBO, and IBSI. Typically, for bond length, C—N is of 1.48 Å, C=N of 1.35 Å, N=N of 1.25 Å, and N≡N of 1.10 Å.

b) Dist. stands for distance in unit Å is measured via GaussView 6.0 for reference.

c) N/A: Not applicable.

Furthermore, quantitative methods, including bond order density (BOD) and the natural adaptive orbitals (NAdO), were also employed to analyze the starting material, TS, and product for bisdiazo‐H, with bond order and distance listed in Table 8. Both BOD and NadO visualize the spatial distribution of the covalent bond order and its eigen components, aiding in the evaluation of chemical bonds^[^ ^115^ ^]^ for TS and product. NadO, closely related to BOD, provides an orbital‐based view of the delocalization index. These analyses (BOD and NAdO) rely on the atomic overlap matrix, obtained via fuzzy atomic space or basin analysis (for AIM partition). The NadO‐based fuzzy bond order is given in **Table** **9**, with orbital contributions detailed in Figure S4, Supporting Information. Each NAdO orbital, associated with certain eigenvalues (occupation numbers between atoms) contributed equally to bonding. This NAdO orbital decomposition offers insights into how *σ*‐ and/or *π*‐orbital evolves from a diazo to carbene, with some orbitals remaining unchanged during such a transition.

**Table 9:** NAdO indices of diazo sites (14C‐35 N‐37 N) in starting, TS, and product from IRC path.

X	Starting		TS		Product	
	14C‐35 N	35 N‐37 N	14C‐35 N	35 N‐37 N	14C‐35 N	35 N‐37 N
H	1.47296	2.51805	0.63941	2.89331	N/Aa)	3.09671
Me	1.47420	2.51685	0.63959	2.89230		3.09529
NO_2_	1.47638	2.51478	0.64269	2.90708		3.09363
NH_2_	1.46620	2.52450	0.64012	2.89091		3.09469

a) N/A: Not applicable.

#### 3.7.2 Electronic Structure of Diazo‐Carbene and Biscarbene Analysis

The IRC path calculations suggest that diazo‐carbene and biscarbenes are highly likely to be the products from bisdiazo compounds, regardless of terminal groups. The spin states (singlet or triplet) of these species were confirmed through the energy evaluations at the level of (U)M062X‐D3 with the optimized structures at the method of B3LYP‐D3BJ with the same basis set of 6 – 311 + G(d, p). As shown in **Table** **10**, both singlet and triplet states were evaluated, with the triplet state of diazo‐carbene and biscarbene species being the lowest in energy, regardless of the number of carbene sites.

**Table 10:** Minimum energy (a.u.) of both singlet and triplet diazocarbene and biscarbene species.

Carbene‐X		*E*_Single*t*_	*E*_Triplet_	*E*_S–T_	*E*_min_ a)
H	Diazo‐carbene	−1492.947056	−1492.950298	−0.006488	*E*_*T*_
	Biscarbene	−1382.647029	−1382.602097	−0.044932	*E*_*S*_
Me	Diazo‐carbene	−1570.814792	−1570.820278	0.005486	*E*_*T*_
	Biscarbene	−1461.260757	−1461.262931	0.002174	*E*_*T*_
NO_2_	Diazo‐carbene	−1901.180669	−1901.188333	0.007664	*E*_*T*_
	Biscarbene	−1791.629121	−1791.637734	0.008613	*E*_*T*_
NH_2_	Diazo‐carbene	−1602.909905	−1602.914962	0.005057	*E*_*T*_
	Biscarbene	−1493.356205	−1493.358450	0.002245	*E*_*T*_

a) Note: energy unit 1.0 a.u. = 27.211 eV = 627.5 kcal mol^−1^ = 2625 kJ mol^−1^.

Carbenes are usually considered *sp* ^2^‐hybridized structures, with spin multiplicity determining whether they exist in a singlet or triplet GS. Singlet carbene possesses a vacant p‐orbital, thereby making them highly electrophilic, while triplet carbenes have two nonbonding *sp* ^2^ and p‐orbitals containing one electron each,^[^ ^116^ ^]^ therefore giving them biradical character. Triplet carbenes are less ionic, more radical‐like, whereas singlet carbenes are electron‐deficient and reactive toward nucleophiles, including tertiary amines, phosphines, ethers, sulfides, and sulfoxides.^[^ ^117^, ^118^, ^119^ ^]^ Substituents significantly influence carbene electronic properties, with singlet carbenes generally being highly electrophilic.^[^ ^118^, ^119^, ^120^, ^121^, ^122^ ^]^

**Figure 13:**
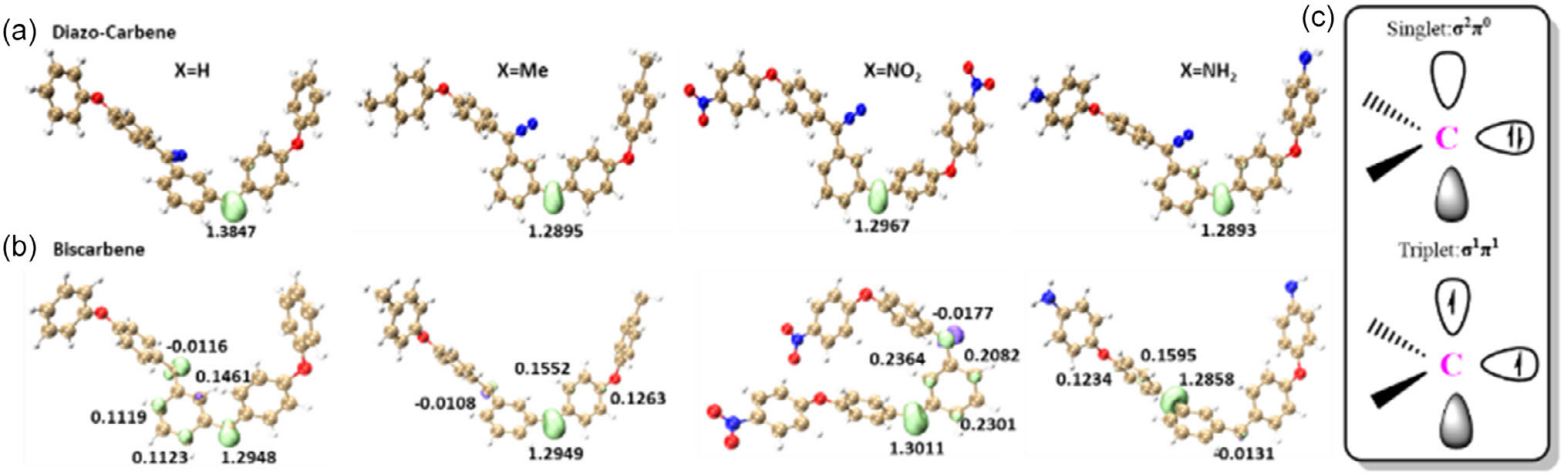
Spin density isoplots of bisdiazo‐X compounds derived carbene species at triplet state of a) diazo‐carbene and b) biscarbene (lime (+) and purple (−)) spin density isoplots are at isovalue of 0.02 a.u., and the numbers are the calculated Hirshfeld atomic spin population, and c) electronic structures of carbine‐centered carbon atom in both singlet and triplet states.

Spin density, denoting the difference between spin‐up and spin‐down electron contributions, is zero in diamagnetic systems (all electrons paired) and nonzero overall in paramagnetic ones (at least one unpaired electron). Positive spin density aligns with singly occupied MOs (SOMOs), whereas negative spin density tends to be small and arises at sites which are formally nodal with respect to the SOMOs. The spin density calculation for diazo‐monocarbene and biscarbene species from various bisdiazo compounds was performed to probe their paramagnetic character. For bisdiazo‐H, only the triplet state exhibits significant spin density at the carbene‐centered carbon atom (**Figure** **13**a,b). Both singlet and triplet states demonstrate low‐lying electronic structures.^[^ ^123^, ^124^ ^]^ In triplet‐state species, the two unpaired electrons generally occupy *π*‐MOs, resulting in a total *p*‐spin populations of +1 (not +2), typically true for *π* radicals. Thus, the *π*‐spin distribution in a triplet molecule is comparable to that in a structurally similar *π* radical, relating the coupling constants with the *π*‐spin populations, which can also be used for triplet states. The spin populations at carbene centers are similar for diazo‐carbenes (Figure 13a), while in biscarbenes, the second carbene center shows small negative values (Figure 13b). Overall, the spin density distributions (Figure 13c) align with expected triplet carbene electronic structures.

**Figure 14:**
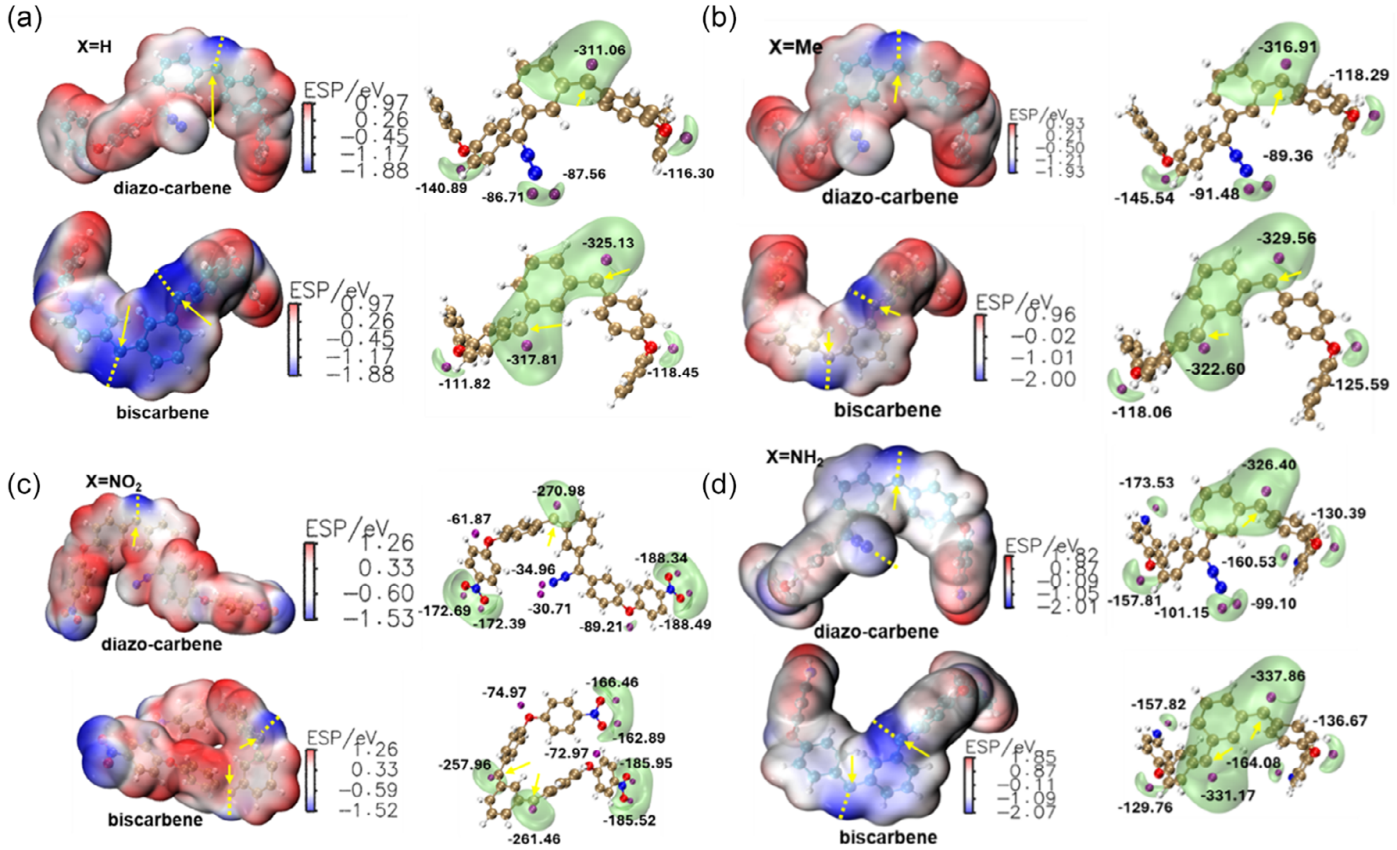
ESP isosurface plots and corresponding min energy spots *V*_min_ of diazo‐carbene and biscarbene in singlet states from bisdiazo‐X compounds with varied terminal groups: a) X = H, b) X = Me, c) X = NO_2_, and d) X = NH_2_. (EPS *V*_min_ is located at the isovalue of −0.0275 a.u. = −72.20 kJ mol^−1^ with the purple beads marked in kcal mol^−1^, yellow arrow points out the carbene‐centered C atoms).

Similarly to the quantum theory of atoms in molecules (i.e., *ESP*_min_) analysis of locating lone pair electrons in bisdiazo compounds (Figure 7), ESP surface mapping and ESP_min_ calculations of diazocarbene and biscarbenes (**Figure** **14**) show that the carbene carbon atom is the most reactive site. Among all species, biscarbene‐NO_2_ has an extended geometry, exposing both carbene carbons and enhancing the chemically reactivity, and the nitro groups also form a secondary reactive region.

**Figure 15:**
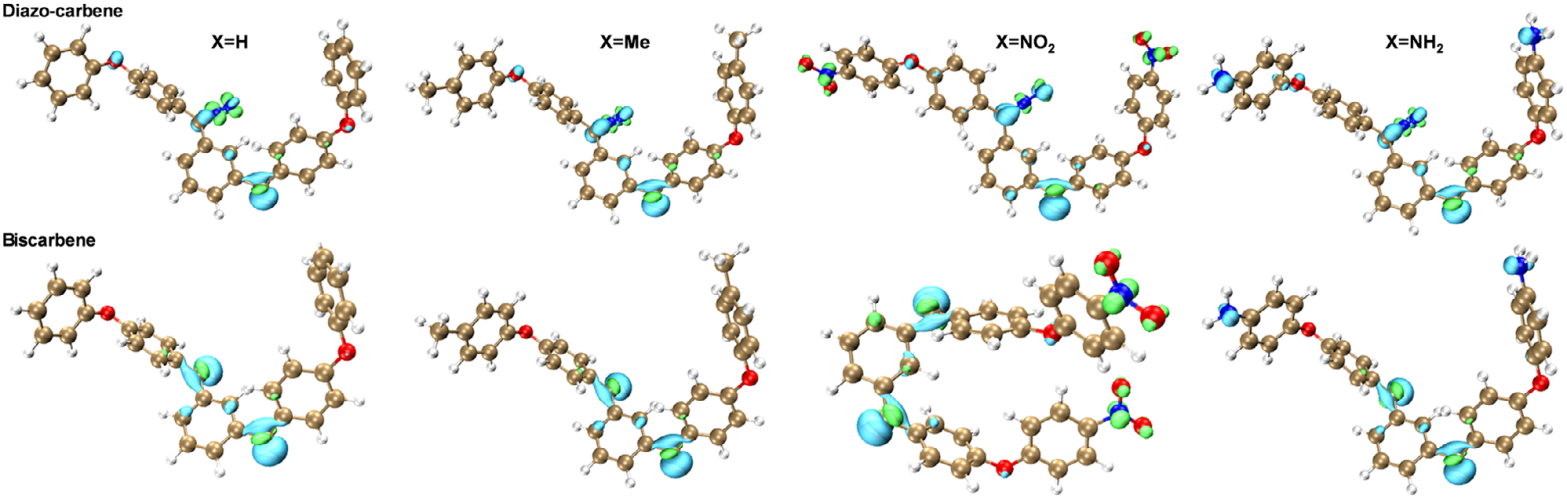
Reaction site predictions by orbital‐weighted Fukui dual descriptor of diazo‐carbene and biscarbene in singlet states from bisdiazo‐X compounds. (X = H, Me, NO_2_, NH_2_, isoplots with isovalue of 0.0025 a.u., green and cyan isosurfaces represent positive and negative parts, respectively.)

In addition, the triplet state is the lowest in energy for all the carbene species (Table 10), and the singlet is an excited one. The orbital‐weighted Fukui dual descriptor (**Figure** **15**) predicts reactive sites by isosurface mapping, where green and cyan isosurfaces indicate negative and positive regions, respectively. In the diazo group (i.e., C=N^−^=N^+^), the carbon is the electrophilic reaction site and the N^−^ is the nucleophilic one, although this varies with terminal groups. For X = H, electrophilic reactivity dominates over nucleophilicity, while the opposite is true for the other three systems. No reactivity is predicted on the X = H and X = Me terminal groups as expected, while nucleophilic reactivity occurs on nitro (—NO_2_) and electrophilic reactivity on amine (—NH_2_) (Figure 15, Diazo‐carbene). The same pattern is observed for the corresponding biscarbenes as shown in biscarbene plots (Figure 15).

#### 3.7.3 Electron Spin Resonance (ESR) and Electron Paramagnetic Resonance (EPR)

Electron spin resonance (ESR) spectroscopy is key tool for identifying triplet carbenes and probing their molecular and electronic structures.^[^ ^125^, ^126^, ^127^ ^]^ A triplet state has three spin‐magnetic quantum numbers (i.e., *m*_s_ = 1, 0, and −1). Dipole coupling between the two unpaired electrons creates an internal magnetic field, splitting the energy levels. The energy separation between the stabilized level (*m*_s_ = 0) and the destabilized ones (*m*_s_ = ±1) is denoted as *D*. If the molecule lacks cylindrical symmetry, the internal field differs along the *x* and *y* axes, causing further splitting of the *m*_s_ = ±1 levels, referred to as *E*. The constants *D* and *E* are known as zero‐field splitting (ZFS) parameters. Triplet EPR spectroscopy reveals the spatial distribution of unpaired electrons. *D* and *E* are obtained from the spectrum and converted to energy units (cm^−1^), reported as *D/hc* and *E/hc*. *D* reflects the strength of spin–spin interaction along the *z*‐axis, with larger values indicating stronger interaction and closer proximity of the spins. *E* reflects the difference in magnetic dipole interactions along the *x* and *y* axes. Consequently, spin‐orbit coupling can also influence ZFS parameters.^[^ ^128^, ^129^ ^]^

In carbenes with a conjugated *π*‐system, greater electron delocalization reduces the repulsive interaction *D*. Conversely, a larger bond angle at the carbene center increases p‐orbital contribution and decreases *E*. The *E*/*D* ratio correlates with the bond angle at the divalent carbon atom in triplet carbene,^[^ ^130^ ^]^ as observed in X‐band EPR under both inert atmosphere and extremely low‐temperature conditions (even down to 4 K). Here, ZFS tensors were calculated at the UKS B3LYP EPR‐II UNO level using ORCA 5.0.3, with the carbene carbon centered in all species. EPR spectroscopy^[^ ^131^ ^]^ is widely used to study systems with unpaired electrons (usually unstable radicals). Moreover, the *g*‐tensor and *A*‐tensor provide insights into radical conformation, electronic structure, and other properties.^[^ ^132^, ^133^ ^]^ The calculated ZFS parameters for triplet carbenes derived from bisdiazo compounds are similar across terminal groups (**Table** **11**). Their *D* values are very close to that of 0.365 cm^−1^ for carbene anthronylidene (II),^[^ ^134^ ^]^ but differ from other reported carbenes, such as a lower value of 0.105 cm^−1^ for triplet di(9‐anthryl)carbene^[^ ^135^ ^]^ and a higher value of ≈0.508 cm^−1^ for 3‐thienylcarbene,^[^ ^136^ ^]^ with literature values observed for several carbene species ranging from 0.0890 to 0.69.^[^ ^137^ ^]^

**Table 11:** Predicted ZFS parameters of bisdiazo derivatized carbene species at triplet states.

Carbene derivatives		|*D/hc*| [cm^−1^]	|*E/hc*| [cm^−1^]	*E/D*
H	DiazoCarbene	0.336096	0.01323	0.039377
	Biscarbene	0.329277	0.01199	0.036418
Me	DiazoCarbene	0.335994	0.01323	0.039365
	Biscarbene	0.328997	0.01198	0.036412
NO_2_	DiazoCarbene	0.342525	0.01352	0.039459
	Biscarbene	0.317527	0.01223	0.038512
NH_2_	DiazoCarbene	0.336236	0.01320	0.039235
	Biscarbene	0.315596	0.01170	0.037078

EPR is also a powerful tool for identifying structural defects in solids. Spectra from spin ½ centers arise from the *g*‐tensor and the hyperfine couplings (HFC). The *g*‐tensor results from the interaction between electronic spin and the external magnetic field, modified by induced currents in the sample. The EPR spectrum can be modeled using an effective Hamiltonian bilinear in total electron spin *S* and magnetic field *B* or nuclear spins *I*. This model combines contributions from the *g*‐tensor and hyperfine tensor *A*. The total *g*‐tensor is derived from corrections including the electron Zeeman kinetic energy (Δ*g*_Z‐KE_), spin‐orbit (Δ*g*_SO_), and the spin‐other‐orbit correction (Δ*g*_SOO_). As shown in **Table** **12**, all bisdiazo compounds‐derived carbenes exhibit similar *g*‐tenor but differ in their *A*‐tensor.

**Table 12:** Predicted EPR *g*‐tensor and HFC of bisdiazo derivatized carbene species at triplet states.

Carbene species		*g*‐tensor				HFC (Ai, MHz)			
		*g*_*xx*_	*g*_*yy*_	*g*_*zz*_	*g*_iso_	*A*_*xx*_	*A*_*yy*_	*A*_*zz*_	*A*_*iso*_
H	DiazoCarbene	2.0023203	2.0024704	2.0025832	2.0024580	112.1987	186.3277	207.2702	168.5989
	Biscarbene	2.0021771	2.0024068	2.0025778	2.0023872	108.9364	182.6290	204.0021	165.1892
Me	DiazoCarbene	2.0023238	2.0024794	2.0025885	2.0024639	112.2317	186.3357	207.2075	168.5916
	Biscarbene	2.0021654	2.0024164	2.0025922	2.0023913	109.0241	182.6465	204.0317	165.2341
NO_2_	DiazoCarbene	2.0023053	2.0023675	2.0025980	2.0024236	114.2594	188.7509	210.3152	171.1085
	Biscarbene	2.0022133	2.0023417	2.0024859	2.0023470	−0.5824	58.8180	−60.8071	−0.8572
NH_2_	DiazoCarbene	2.0023310	2.0024998	2.0025850	2.0024719	111.8594	186.0671	206.6007	168.1757
	Biscarbene	2.0022485	2.0024591	2.0025792	2.0024289	−11.2942	41.7274	−69.0448	−12.8706

### 3.8 Physisorption of Bisdiazo and Its Carbene Species on the Surface of Single‐Layer Graphene

#### 3.8.1 Physisorption Observation

**Figure 16:**
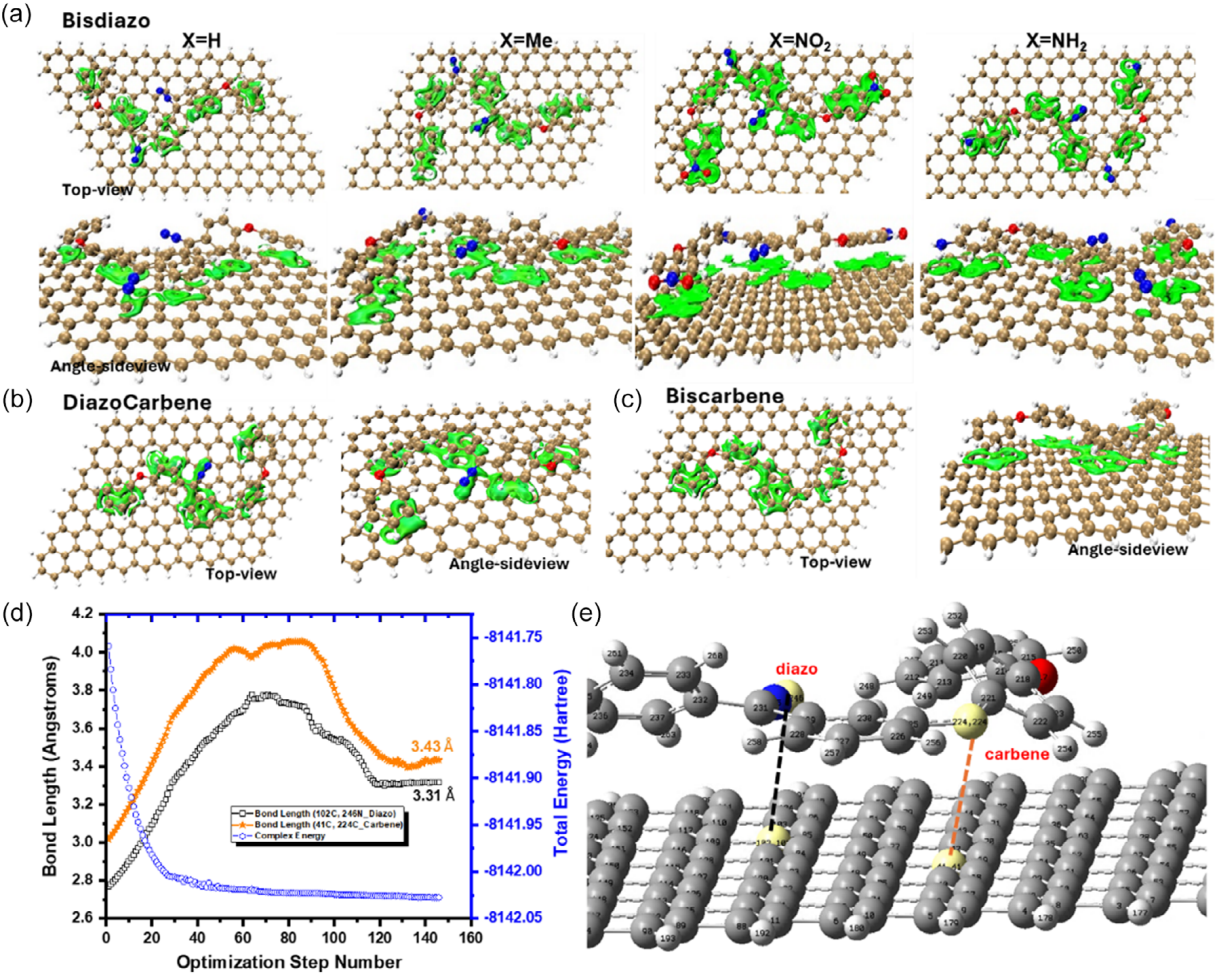
IGMH analysis on physisorption of a) bisdiazo compounds, bisdiazo‐H derivatives, b) diazo‐carbene, and c) biscarbene onto the surface of single‐layered graphene (IGMH isoplots at the isovalue of *δ*_*g*_ = 0.004 a.u. under different view angles), and d) distance between the atoms of interest between the compound and graphene that e) uses diazocarbene onto the graphene surface as an example.

**Figure** **16**a–c presents an analysis using IGMs based on Hirshfeld partition (IGMH) to investigate the physisorption of bisdiazo compounds and their carbene species on a single‐layer graphene surface. In this analysis, the system was divided into two fragments: the organic compound and the graphene surface. As shown in the top‐view images in Figure 16, the physisorption occurs through two primary interactions. The first is mainly the well‐known *π–π* stacking effect clearly between the aromatic rings of the molecule and the hexagonal lattice of the graphene layer. The second involves the outstanding associative lone pair–*π* interactions,^[^ ^138^ ^]^ where lone pairs originate from the oxygen and nitrogen atoms as supported in both Figure 7 and 14 (i.e., the lone pair is indicated by purple beads in the doom‐shaped ESP_min_ mappings).

From all the calculations, the calculated distance between the atomic centers of interest—such as the carbene atom and the outermost nitrogen atom—is ≈3.50 Å, as shown in Figure 16d,e. This distance closely matches the measured interlayer spacing observed in AA‐ and AB‐stacked bilayer graphene,^[^ ^139^, ^140^ ^]^ highlighting the significant vdW force^[^ ^141^, ^142^ ^]^ arising from *π–π* stacking. This effect is also evident in the green isosurfaces shown in Figure 16a–c, which confirm the strong noncovalent interactions for aromatic‐ring‐enriched bisdiazo compounds and their derivatives, including diazocarbene and biscarbene species.

#### 3.8.2 Constant Height STM Image Simulation

**Figure 17:**
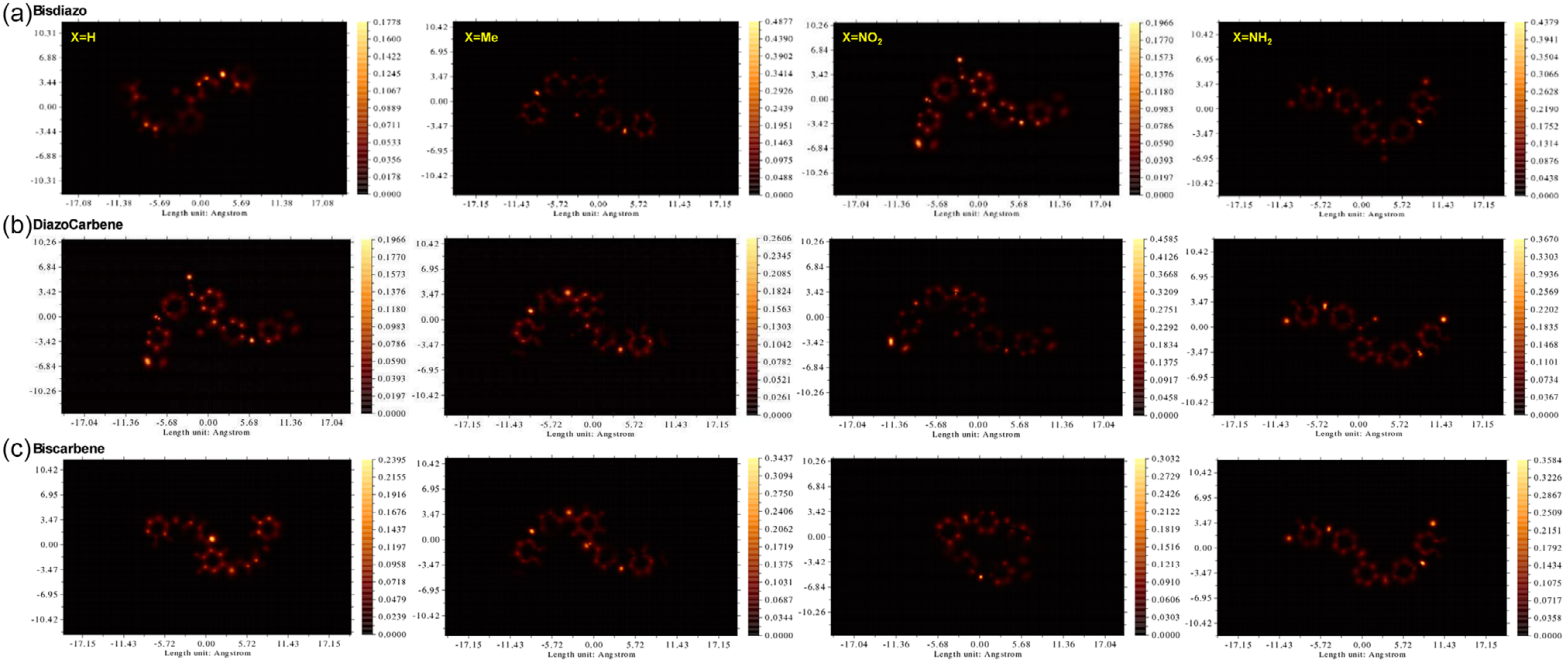
Calculated STM images for a) bisdiazo compounds and their b) diazo‐carbene, and c) biscarbene species on the surface of single‐layer graphene. (constant height = 3.65 Å).

Scanning tunneling microscopy (STM) is a powerful tool for probing electronic properties of materials, and theoretical simulations have shown good agreements with results in describing MO distributions, electron density,^[^ ^143^, ^144^, ^145^ ^]^ and spin excitation.^[^ ^146^ ^]^ STM images reflect electron wave functions, which are indirectly related to molecular structure and intermolecular bonds,^[^ ^147^, ^148^, ^149^ ^]^ as revealed through topographic and differential conductance images. In STM experiments, a conducting tip is positioned over the sample, and a bias voltage (*V*) is applied between them. Due to the quantum tunneling effect, a tunneling current (*I*) flows when the tip‐sample distance (*r*) and voltage *V* are appropriate. Since *I* varies with position depending on the local tip‐sample interaction, it effectively maps the function *I(r)*, which is proportional to the local density of states (LDOS) at position *r*.^[^ ^150^ ^]^ In STM images, brighter regions correspond to higher LDOS and thus the stronger the tunneling current (*I*). Here, the simulated STM images were calculated through Multiwfn 3.8 dev and are illustrated in **Figure** **17**. Based on the Tersoff–Hamann model,^[^ ^151^, ^152^, ^153^ ^]^ the tunneling current is positively proportional to LDOS. In these simulations, *I* is most intense over the benzene rings, where most atoms lie in the same plane, consistent with the planarity analysis in Figure 4 and the physisorption behavior of the molecule on single‐layer graphene (Figure 16). The simulated STM image of graphene (Figure S5, Supporting Information) clearly shows hexagonal rings, even more distinctly than those observed in chemical vapor deposition‐grown graphene.^[^ ^154^ ^]^ In contrast, the STM image of bisdiazo compounds and their carbene derivatives appears blurred, possibly due to their nonplanar structures (Figure 4 and Figure S2, Supporting Information), or their physisorbed configuration on graphene, similar to observations in cobalt phthalocyanine on noble metal surfaces.^[^ ^155^ ^]^

Using single‐layer graphene as support, the absorbed highly active carbene species could serve as active sites for further surface functionalization, enabling the growth of patterned organic layers with functional polymers^[^ ^156^ ^]^ and controlled textures.^[^ ^157^ ^]^ Such systems may find applications in hierarchical structures for proteins at nano‐ and microscales,^[^ ^158^ ^]^ as well as ultrahigh‐resolution microelectronics and optoelectronics via organic semiconductor growth.^[^ ^159^ ^]^ To our best knowledge, although STM imaging of NHCs has been reported on various substrates,^[^ ^160^, ^161^, ^162^ ^]^ experimental studies on the controlled adsorption and stabilization of biecarbenes on surfaces remain unexplored and represent a promising direction for future research.

## 4 Conclusion

The theoretical properties of bisdiazo compounds bearing various terminal functional groups, along with their derived carbene species (diazocarbene and biscarbene), have been thoroughly probed via DFT. These studies encompass both GS and excited states, conducted in different solvent environments (THF, toluene, and chlorobenzene). Key molecular properties analyzed include FMOs, ESP, dipole moment, polarity, and overall electronic structures. Infrared (IR) and UV–visible spectra calculated for both ground and excited states show good agreement with the experimental results, and the predicted colors closely match the observed results. Furthermore, a generalized IRC path for the formation of carbene species from bisdiazo compounds has been validated through an IGM based on Hirshfeld partition (IGMH). Potential sites for lone pair electron location were identified using minimum ESP (ESP_min_) mapping and orbital‐weighted Fukui dual descriptor, aiding in the prediction of reactive sites in their well‐known intermediates. Additionally, spin density, spin population, and EPR/ESR parameters were computed to characterize the radical‐like behavior of the carbene species. Further insights into the physisorption behavior of bisdiazo and their carbene species onto single‐layer graphene were evaluated through geometry optimization and simulated STM imaging. These computational models provide a valuable reference for interpreting potential experimental STM observations.

## Conflict of Interest

The authors declare no conflict of interest.

## Author Contributions

**Xiaosong Liu**: methodology (lead); data curation (lead); software (equal); validation (lead); visualization (lead); writing—original draft (lead); writing—review & editing (equal). **Mark Gerard Moloney**: conceptualization (equal); software (lead); formal analysis (equal); supervision (equal).

## Supporting information

Supplementary Material

## Data Availability

The data that support the findings of this study are available from the corresponding author upon reasonable request.
