# Supplementary material for: Properties of Bisdiazo Compounds and Their Derived Carbenes via Density Functional Theory
Source: Chemphyschem. 2025 Dec 5;27(1):e202500438. doi: 10.1002/cphc.202500438 (PMC12810606; doi:10.1002/cphc.202500438)
Supplement: Supplementary file 1 — Supplementary Material [file CPHC-27-e202500438-s001.pdf]

Support Information

Properties of Bisdiazo Compounds and Their Carbenes via Density Function Theory

Xiaosong Liu[1], Mark Moloney[1,2*]

1. Oxford Suzhou Center for Advanced Research (OSCAR), Suzhou, China 215123

2. Department of Chemistry, Oxford University, Oxford, UK

1. Preparation of bisdiazo-X compounds with varied terminal groups

1.1 Reagents for synthesis

All the chemicals and solvents were purchased from commercial manufacturers and used as received unless stated otherwise. Here is the basic information about each one chemical reagent used in synthesis and purification.

4-Phenoxyaniline (purity >97.0%) and Celite® (325 mesh powder) were purchased from Alfa Aesar.

Trifluoroacetic anhydride (TFAA, purity >98.0%GC), aluminum chloride ($AlCl_3$, purity>98.0%), isophthaloyl chloride (purity >99.0%), glacial acetic acid (99.9985% metal basis) obtained from Tokyo Chemical Industry (TCI, Shanghai, China).

Acetone (AR, >98.5%), Potassium permanganate ($KMnO_4$, AR, purity > 99.5%) and anhydrous Magnesium sulfate ($MgSO_4$, AR) were purchased from General-Reagent.

Dichloromethane (DCM, AR), ethanol anhydrous (EtOH, AR), sodium hydroxide (NaOH, AR). manganese sulphate tetrahydrate ($MnSO_4 \cdot 4H_2O$, AR, purity > 98.0%), sodium sulfate (Na_2SO_4, AR), potassium hydroxide (KOH, AR) supplied by Sinopharm Chemical Reagent Co., Ltd.

Pyridine (99.5%) was from Adams-beta, and hydrazine monohydrate (37%, purity >98.0%(T)) from Aladdin.

All deuterated-Substituted solvents are provided by Cambridge Isotope Laboratories, Inc.

Active Manganese dioxide (MnO_2) was prepared freshly based on a modified method reported by Attenburow et al.[1] and the details were showed below. Briefly, an aqueous solution by dissolving 16 g of $KMnO_4$ in deionized (DI) water of 100 mL, stirring and being heat up to 90°C on a hotplate. Another aqueous solution of 18.5 g $MnSO_4 \cdot H_2O$ in DI water of 25 mL and 19.5 mL of 40%wt NaOH aqueous solution were added into the above prepared KMnO4 solution in portion wise at the same time. Please be careful since too much of such addition made the stirring solution mixture split out and could hurt the exposed skin. After addition, keep stirring at that temperature for another hour. Then cool it down to room temperature naturally. Vacuum filtration to get wet cakes. Redissolved some of the wet cakes in DI water and centrifugation at 15000 rpm for 20 min and repeat such centrifugation and wash with DI water until the supernatant after centrifugation become colorless. Afterwards, the settlement was collected and dried in an oven of 120°C overnight (~16 h). Finally, powder was collected by griding and stored sealed vial in dark isolated from air as much as possible.

Bisdiazo compounds are quite high energy compounds and should be handled with caution, which are stored at 4°C refrigerator in dark before using, and all the experiment upon heating is fulfilled in fume hood with slash hard enough for safety concerns. Hydrazine monohydrate is highly toxic and needs to be handled based on the established safety protocols.

1.2 General Characterization

The analysis of functional groups present was done using an attenuated total reflection (ATR) Fourier transform infrared spectroscopy (ATR-FTIR, IRAffinity-1s, Shimadzu). The spectral window for the analysis was 4000-600 cm^{-1} at a resolution of 4 cm^{-1} over 16 scans.
The ^{1}H and ^{13}C Nuclear Magnetic Resonance (NMR, JNM-ECZ400S, JEOL, Japan) spectra were recorded at 400 MHz (varied frequencies for ^{13}C due to different solvents) at room temperature.
Ultraviolet visible spectroscopy (UV-vis, UV-1900 spectrometer, Shimadzu) were used for the bisdiazo compounds and their corresponding polymerized ones in tetrahydrofuran (THF) solution in the wavelength of 200-700 nm with a scanning speed of 10 nm/min at room temperature.

1.3 Synthesis of bisdiazo compounds with varied terminal groups

1.3.1 Synthesis of compound Bisdiazo-H

The bisdiazo-H compound was synthesized in house by the procedure described in previously publications that used in polymer surface modification [2-4]. Here, the characterized results of each one involved were showed in **Scheme S1a** alongside the synthesis details in our latest work[5].

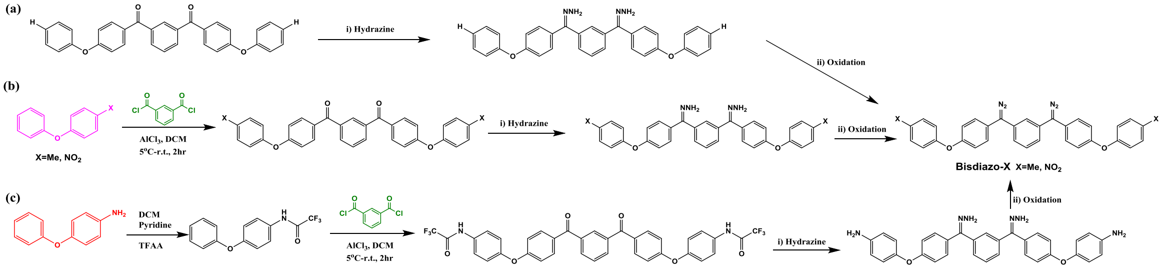

Scheme S1. Simplified procedure of synthesizing bisdiazo-X compound: (a)X=H, (b) X=Me and NO_2, and (c) X=NH_2. Step i) hydrazine is hydrazine monohydrate reduction with catalytic amount of AcOH under refluxing for 40 hr, and ii) oxidation is for active MnO_2 with desired amount of Na_2SO_4, KOH in dark at room temperature for 2 hr.

1-((E)-Hydrazineylidene(4-phenoxyphenyl) methyl)-3-((Z)-hydrazineylidene(4 phenoxyphenyl)methyl)benzene

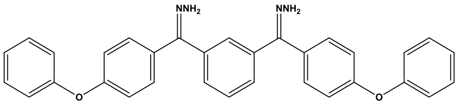

^{1}H-NMR (500.3 MHz, DMSO-d6) δ(ppm): 6.16–6.54 (m, 4H), 6.90–7.56 (m, 22H).
^{13}C-NMR (125.8 MHz, DMSO-d6) δ(ppm): 118.7, 118.8, 119.1, 119.5, 119.6, 119.7, 123.3, 123.9, 124.3, 124.4, 125.0, 125.9, 126.2, 127.5, 127.6, 127.8, 127.9, 128.3, 129.6, 129.8, 130.5, 130.6, 130.8, 131.1, 131.2, 133.3, 134.7, 134.8, 135.0, 139.3, 140.6, 143.3,143.4, 144.2, 144.4, 156.5, 156.7, 157.0, 157.2, 157.4.
IR υ_{max} (cm^{-1}): 3403, 3039, 2922, 1586, 1502, 1486, 1232, 1164, 1070.

1,3-Bis(diazo(4-phenoxyphenyl)methyl)benzene

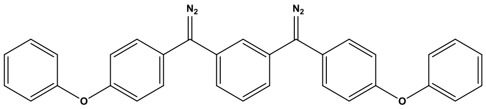

^{1}H-NMR (400 MHz, DCM-D_2) δ(ppm): 7.88 – 5.98 (m, 22H).

^{13}C-NMR (101 MHz, DCM-D_2) δ(ppm): 159.67, 157.51, 156.74, 156.12, 133.48 – 132.55, 131.75, 130.99 – 129.91, 129.22, 128.37, 128.18 – 127.27, 126.31, 126.12 – 125.43, 124.96, 124.67 – 123.52, 121.83, 120.61, 120.30 – 119.63, 119.33, 118.37 – 117.27, 62.28, 54.31.
IR υ_{max} (cm^{-1}): 3039, 2031, 1588, 1503, 1486, 1234, 1165.

1.3.2 Synthesis of compound Bisdiazo-Me

The synthesis procedure of both bisdiazo-Me and bisdiazo-NO_2 are almost the same with different starting materials as showed in **Scheme 1b**, which are 1-methyl-4-phenoxybenzene and 1-nitro-4-phenoxybenzene, respectively.

1,3-Phenylenebis((4-(p-tolyloxy)phenyl)methanone)

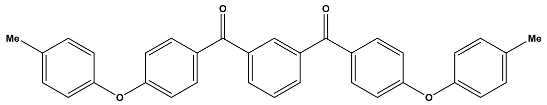

^{1}H-NMR (400 MHz, DMSO-D_6) δ(ppm): 7.96 (dd, J = 7.7, 1.7 Hz, 2H), 7.89 (t, J = 1.7 Hz, 1H), 7.83 – 7.75 (m, 4H), 7.71 (t, J = 7.7 Hz, 1H), 7.30 – 7.17 (m, 4H), 7.06 – 6.97 (m, 8H), 2.29 (s, 6H).
^{13}C-NMR (101 MHz, DMSO-D_6) δ(ppm): 193.67, 161.76, 152.42, 137.42, 134.18, 132.88, 132.47, 130.71, 130.21, 129.10, 120.19, 116.70, 20.34.
IR(cm^{-1}): 2955, 2927, 2853, 1653 (C=O), 1590, 1497, 1236, 1161.

1-((E)-Hydrazineylidene(4-(p-tolyloxy)phenyl)methyl)-3-((Z)-hydrazineylidene(4-(p-tolyloxy)phenyl)methyl)benzene

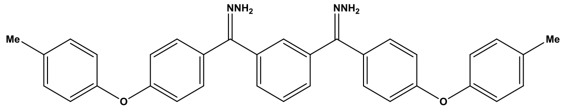

^{1}H-NMR δ(ppm): 6.65-7.50 (aromatic H), 3.92-4.06 (amine 4H), 2.20-2.35 (methyl 2H)
^{13}C-NMR δ(ppm): 20.85, 158.06, 157.86, 157.12, 154.61, 154.57, 154.18, 154.05, 144.58, 144.33, 143.36,140.65, 139.38, 134.36, 133.75, 133.66, 133.37, 133.22, 131.20, 131.09, 131.06, 130.93, 130.25, 130.20, 129.81, 128.33, 127.93, 127.88, 127.55, 120.03, 119.89, 119.41, 119.22, 118.97, 118.94, 118.83, 118.27.
IR(cm^{-1}): 2959,2926, 2855, 1599 (C=N), 1590, 1496, 1235.

1,3-Bis(diazo(4-(p-tolyloxy)phenyl)methyl)benzene

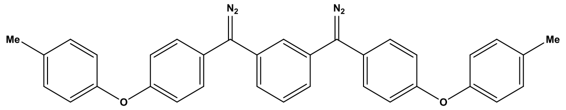

^{1}H-NMR(400 MHz, DMSO-D_6) δ(ppm): 7.55 – 6.55 (m, 20H), 2.31 – 2.16 (m, 6H).
^{13}C-NMR(101 MHz, DMSO-D_6) δ(ppm): 154.44, 133.49, 130.98, 130.48, 128.15, 121.52, 119.49, 118.59, 61.84, 20.80.
IR(cm^{-1}): 2959,2926, 2855, 2032 (N=N), 1590, 1497, 1230, 1161.

1.3.3 Synthesis of compound Bisdiazo-NO_2

Bisdiazo-NO_2 are almost the same synthesis procedure as shown in **Scheme 2**, but with different starting materials, 1-nitro-4-phenoxybenzene.

1,3-Phenylenebis((4-(4-nitrophenoxy)phenyl)methanone)

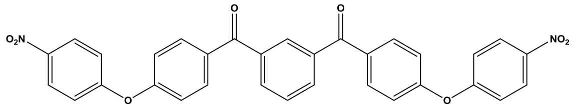

^{1}H-NMR(400 MHz, DCM-D$_2$) δ(ppm): 8.31 – 8.21 (m, 4H), 8.18 (td, *J* = 1.8, 0.6 Hz, 1H), 8.03 (dd, *J* = 7.7, 1.7 Hz, 2H), 7.97 – 7.83 (m, 4H), 7.75 – 7.63 (m, 1H), 7.30 – 7.08 (m, 8H).
^{13}C-NMR(101 MHz, DCM-D$_2$) δ(ppm): 194.57, 162.14, 159.54, 144.08, 138.27, 133.98, 133.71, 133.01, 131.20, 129.08, 126.44, 119.84, 118.99.
IR(cm^{-1}): 3093, 1654 (C=O), 1577, 1508, 1338 (N=O), 1226, 1161, 1103

1-((E)-hydrazineylidene(4-(4-nitrophenoxy)phenyl)methyl)-3-((Z)-hydrazineylidene(4-(4-nitrophenoxy)phenyl)methyl)benzene

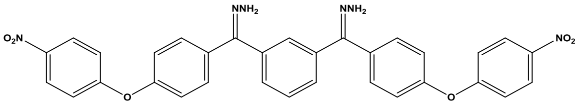

^{1}H-NMR δ(ppm): 8.14-8.27 (aromatic 4H next to NO$_2$), 7.86-6.99 (aromatic H), 5.51 (amine H, 4H)
^{13}C-NMR δ(ppm): 117.3, 117.85, 120.1, 121.2, 124.2, 125.8, 126.3, 126.9, 128.0, 128.3, 129.4, 129.7, 130.7, 131.1, 132.8, 134.1, 135.8, 138.5, 140.0, 142.7, 143.3, 146.4, 147.3, 154.5, 155.6, 162.6, 163.1
IR(cm^{-1}): 3417, 3294, 3070, 1581, 1481, 1342 (N=O), 1234, 1157, 1103, 1010

1,3-Bis(diazo(4-(4-nitrophenoxy)phenyl)methyl)benzene

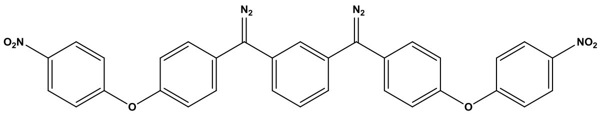

^{1}H-NMR (400 MHz, DMSO-*D*$_6$) δ(ppm): 8.39 – 8.10 (m, 4H), 7.80 – 6.91 (m, 16H), 6.50 (d, *J* = 38.1 Hz, 1H).
^{13}C-NMR (101 MHz, DMSO-*D*$_6$) δ(ppm): 163.30, 153.09, 142.92, 130.65, 128.22 – 127.59, 126.75, 122.14, 120.81, 118.04,
IR(cm^{-1}): 3394, 3086, 2036 (N=N), 1581, 1489, 1335 (N=O), 1242, 1157, 1110.

1.3.4 Synthesis of compound Bisdiazo-NH$_2$

Here, the resulting bisdiazo compound labeled as bisdiazo-NH$_2$ was similar to that of bisdiazo-H compound and **Scheme 1c** briefly describes the synthesis procedure.

2,2,2-Trifluoro-N-(4-phenoxyphenyl)acetamide

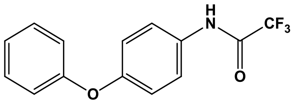

ATR-IR(cm^{-1}): 3290 (N-H),1701 (C=O), 1172 (C-F), 1149 (C-F),
^{1}H-NMR (400 MHz, CDCl$_3$) δ(ppm): 6.99-7.05(m, 4H), 7.13(t, 1H), 7.33-7.38 (m, 2H), 7.50-7.54(m, 2H).
^{13}C-NMR(101 MHz, CDCl$_3$) δ(ppm): 114.4, 117.3, 119.1, 119.5, 122.4, 123.8, 130.0, 130.2, 154.7, 155.0, 155.6, 158.9.
HRMS (ESI-): observed *m/z* 281.07376, $C_{14}H_{10}F_3NO_2$- requires *m/z* 281.07364.

N,N'-(((Isophthaloylbis(4,1-phenylene))bis(oxy))bis(4,1-phenylene))bis(2,2,2-trifluoroacetamide)

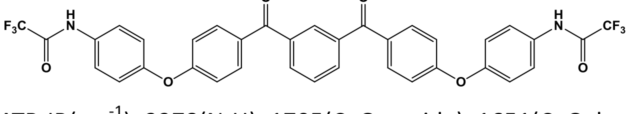

ATR-IR(cm^{-1}): 3278(N-H), 1705(C=O, amide), 1651(C=O, ketone), 1178(C-F), 1150(C-F).

^{1}H NMR (400 MHz, DMSO-D_6) δ(ppm): 11.34 (s, 1H), 8.09 – 7.91 (m, 2H), 7.89 – 7.66 (m, 5H), 7.33 – 6.98 (m, 4H).
^{13}C NMR(101 MHz, DMSO-D_6) δ(ppm): 193.71, 161.29, 154.62, 154.26, 152.03, 137.43, 133.02, 132.53, 131.08, 130.15, 129.07, 123.02, 120.73, 120.12, 117.16, 114.38.
^{19}F NMR (376 MHz, DMSO-D_6) δ(ppm): -73.76, -73.81 (d, J = 12.7 Hz).
HRMS (ESI+): observed *m/z* 693.1450, $C_{36}H_{23}F_6N_2O_6$+ requires *m/z* 693.1454.

4,4'-((((1Z,1'Z)-1,3-Phenylenebis(hydrazineylidenemethylene))bis(4,1-phenylene))bis(oxy))dianiline

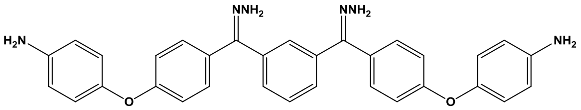

IR (neat, cm^{-1}): 3350 (N-H, amine), 3426 (N-H, hydrazone), 3040 (C-H, aromatic), 1601 (C=N).
^{1}H-NMR (400 MHz, $CDCl_3$) δ(ppm): 3.52 (s, 4H), 5.12-5.64(m, 4H), 6.63-6.74(m, 5H, aromatic), 6.84-6.93(m, 7H, aromatic), 6.98-7.05(m, 1H, aromatic), 7.09-7.22(m, 3H, aromatic), 7.30-7.62 (m, 4H, aromatic).
HRMS (ESI+): observed *m/z* 529.23462, $C_{32}H_{29}N_6O_2$+ requires *m/z* 529.23465.

4,4'-(((1,3-Phenylenebis(diazomethylene))bis(4,1-phenylene))bis(oxy))dianiline

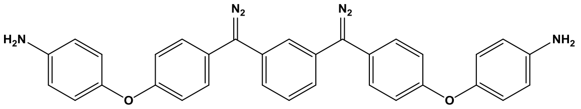

ATR-IR(cm^{-1}): 3437, 3344, 3039(C-H, aromatic), 2022 (N=N), 1593, 1496, 1222.
^{1}H NMR (400 MHz, DCM-D_2) δ(ppm): 7.51 – 6.73 (m, 4H), 6.72 – 6.58 (m, 1H), 3.66 (s, 1H).
^{13}C NMR (101 MHz, DCM-D_2) δ(ppm): 158.01, 148.44, 147.83, 143.97, 131.79, 130.46, 130.02, 129.07, 127.79, 125.76, 122.51, 122.35 – 121.63, 121.43, 120.21, 118.33, 117.47, 116.63, 116.34, 114.93, 62.13, 54.31.
HRMS (ESI+): observed *m/z* 525.2035, $C_{32}H_{25}N_6O_2$+ requires *m/z* 525.2033.

2 Theoretical calculation details

2.1 DFT calculation level validation

Theory validation level of DFT calculation for geomeotry optimization and frequency check at theory level of B3LYP-D3BJ/6-311+G(d,p) for all the bisdiazo compounds are as detailed in Table S1. And the validation criteria are to obtain the min Energy without any imaginary frequency and 4-Yes on convergence on the Max Force, RMS Force, Max displacement and RMA displacement.

Table S1. DFT Calculation Summary of varied bisdiazo compounds at level of B3LYP/6-311+G(d,p) with D3(BJ) dispersion effect under additional keyword of opt=tight.

Bisdiazo-X	Energy /eV	MaxF/RMS/MaxD/RMAD	Step#	Diazo site	Vibration
**H**	-43607.3608	Y/Y/Y/Y	60	2-√	No imaginary
**Me**	-45747.9468	Y/Y/Y/Y	79	2-√	No imaginary
**NO_2**	-54740.6685	Y/Y/Y/Y	67	2-√	No imaginary
**NH_2**	-46621.2511	Y/Y/Y/Y	61	2-√	No imaginary

*Note: Max Force=MaxF, RMS Force=RMSF, Max displacement=MaxD, RMA displacement=RMAD, Two Diazo sites (C=N=N, 2-√).

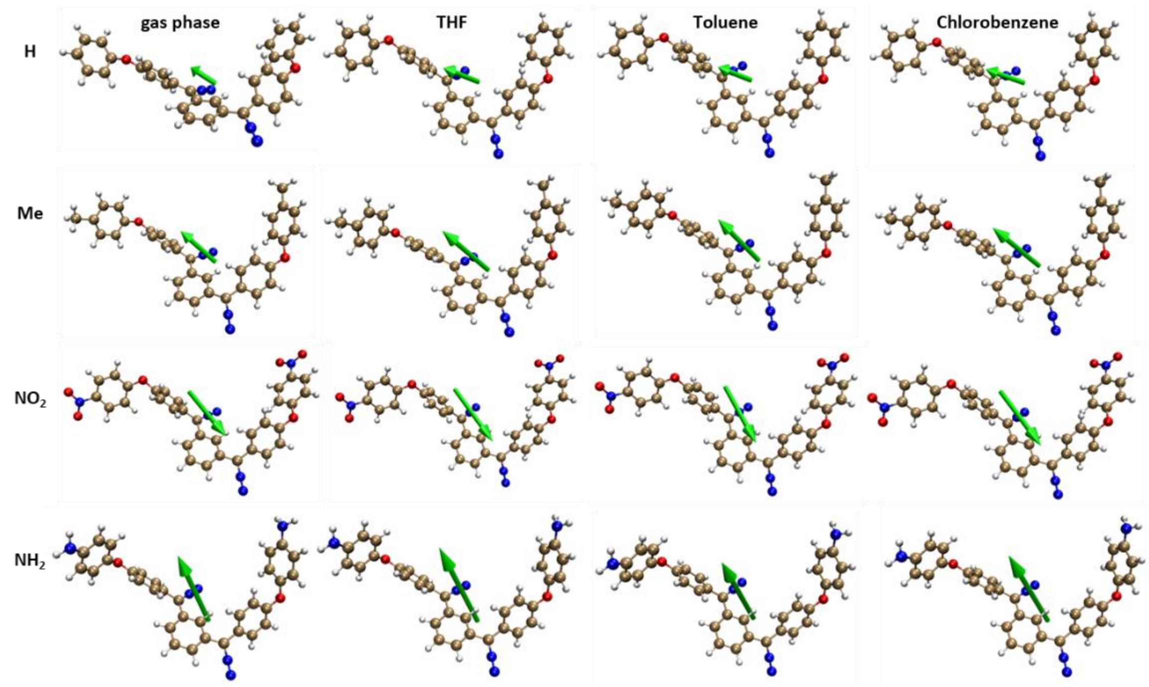

Figure S1. Dipole moment of bisdiazo-X in gas phase and different solvents (green arrow points out the dipole moment direction)

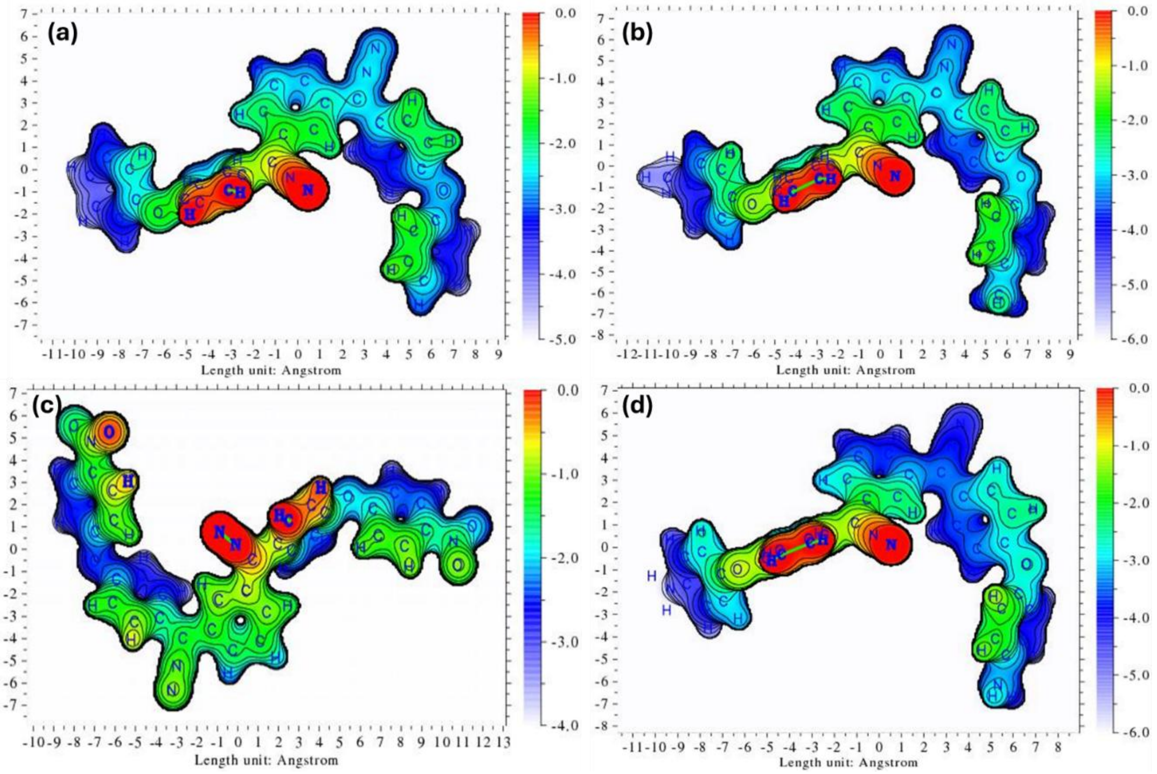

Figure S2. Surface distance projection map in the X-Y plane of the bisdiazo-X compounds: (a) X=H, (b) X=Me, (c) X=NO_2, (d) X=NH_2. (isosurface plots at promolecular density of 0.05 a.u. Red is the screen, and blue is the distance away from the screen.)

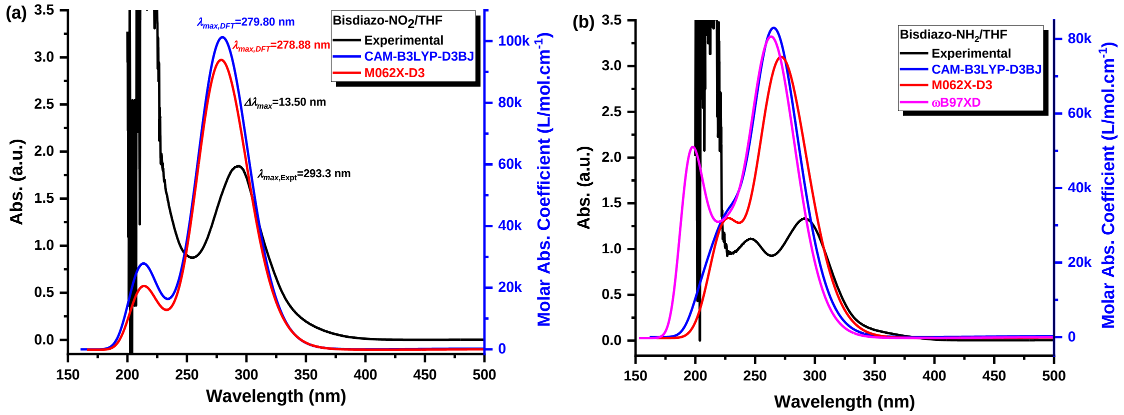

Figure S3. Effect of different theory levels on UV-vis spectrum of both (a) bisdiazo-NO_2 and (b) bisdiazo-NH_2 in THF obtained by DFT calculation and experiment.

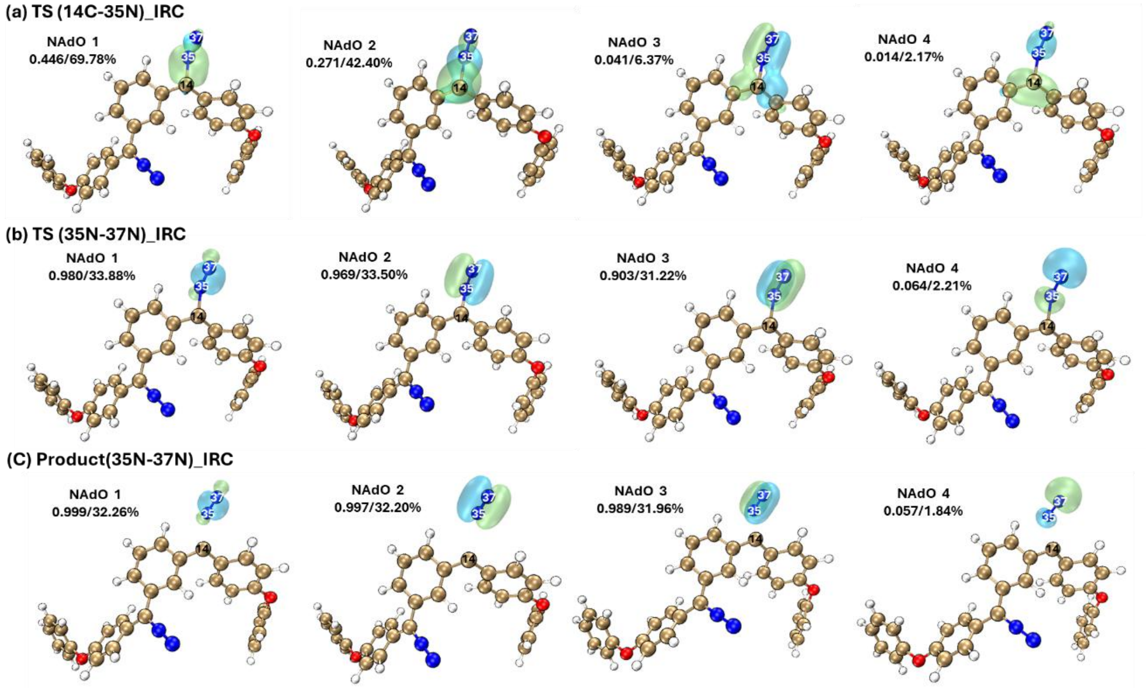

Figure S4. Isoplots of NAdO orbital decomposition contribution analysis of IRC confirmed TS and product from bisdiazo-H compounds (isovalue of 0.05 a.u.).

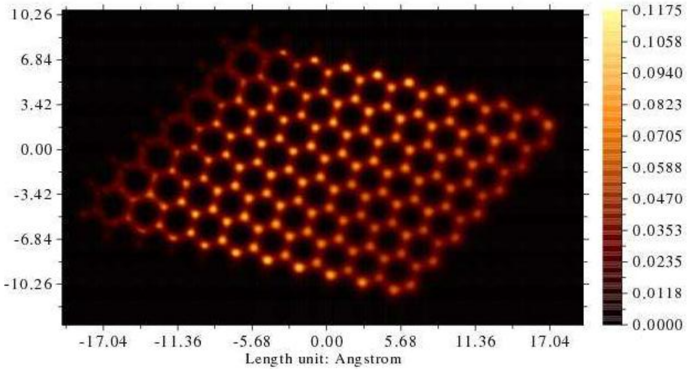

Figure S5. Simulated STM image of single-layer graphene.

2.2 Geometry optimized coordinate of Bisdiazo Compounds

2.2.1 Ground state

(1) Bisdiazo-H

C	5.71675700	-4.99921100	-0.33905000
C	5.01391200	-4.12283800	0.48685200
C	5.27927500	-2.75651300	0.45830400
C	6.25138100	-2.27136600	-0.41522000
C	6.96223500	-3.13425900	-1.24334700
C	6.69314300	-4.50011400	-1.19912000
O	6.60343300	-0.93408000	-0.44783400
C	5.61934600	0.02838300	-0.39739500
C	4.39642500	-0.11598700	-1.05286600
C	3.46778800	0.91496000	-1.00737500
C	3.73373800	2.09879300	-0.30387400
C	4.97477600	2.22770300	0.33329600
C	5.91436800	1.20514000	0.28465400
C	2.72874900	3.17986100	-0.25225300
C	1.28022800	2.99962200	-0.08551300
C	0.36795900	4.00521100	-0.44678000
C	-0.99412800	3.80745100	-0.25661600
C	-1.48100600	2.62008000	0.27920700
C	-0.58690300	1.59885700	0.63749000
C	0.78610500	1.80580800	0.45399000
C	-1.08599000	0.34540500	1.22416600
C	-2.43671700	-0.22037300	1.04669500
C	-3.10916100	-0.09020500	-0.17733000
C	-4.37603200	-0.62906400	-0.36131600
C	-4.99072200	-1.31949900	0.68299100
C	-4.33469900	-1.47472500	1.90089700
C	-3.07179100	-0.92480300	2.07927300
O	-6.21676700	-1.93385900	0.54820600
C	-7.19466800	-1.34552000	-0.23227600
C	-7.48130900	0.01614900	-0.14894400
C	-8.52114700	0.53902800	-0.91263200

C	-9.27477100	-0.28787400	-1.74491500
C	-8.98271600	-1.64888700	-1.80979500
C	-7.93978300	-2.18274800	-1.05648800
N	3.17539200	4.40462500	-0.33583800
N	-0.25693300	-0.33566200	1.96965000
N	3.56114900	5.47449800	-0.40630100
N	0.46941300	-0.92515900	2.62084400
H	5.50651600	-6.06141800	-0.31013600
H	4.25864100	-4.50248000	1.16507000
H	4.74053100	-2.07392500	1.10314900
H	7.71462300	-2.72523400	-1.90596100
H	7.24619800	-5.17361100	-1.84334100
H	4.17884900	-1.02450800	-1.59936500
H	2.52766400	0.80492200	-1.53295500
H	5.20328700	3.13012800	0.88876500
H	6.87093100	1.29844300	0.78314300
H	0.72101800	4.93350700	-0.87947800
H	-1.68970400	4.59253300	-0.53019200
H	-2.54338400	2.48941300	0.43362000
H	1.48520600	1.03062100	0.73807200
H	-2.62908000	0.42489200	-0.99949200
H	-4.88001000	-0.52562300	-1.31354700
H	-4.82786400	-2.01578300	2.69859800
H	-2.57950300	-1.03449800	3.03880500
H	-6.89759600	0.65169000	0.50491100
H	-8.74776100	1.59698900	-0.84895400
H	-10.08392900	0.12514200	-2.33481400
H	-9.56434400	-2.29982600	-2.45199000
H	-7.69461200	-3.23675200	-1.09363200

(2) Bisdiazo-Me

C	5.96356900	-4.60330800	-0.42164300
C	5.32344500	-3.76448100	0.49851800
C	5.55212700	-2.39335500	0.51105400
C	6.43031100	-1.83993600	-0.41842100
C	7.08268400	-2.64810800	-1.33950800
C	6.84877400	-4.02214600	-1.33203600
O	6.74108600	-0.48929900	-0.40550900
C	5.72221000	0.43492800	-0.40697000
C	4.48469200	0.19901700	-1.00649300
C	3.52063600	1.19895000	-1.00722100
C	3.76315200	2.44089700	-0.40435400
C	5.01815200	2.66021100	0.17959100
C	5.99304800	1.67129100	0.17502300
C	2.72103700	3.48791300	-0.39985400
C	1.28691600	3.27227700	-0.16515000
C	0.32641700	4.21614700	-0.56624600
C	-1.02010300	3.98698600	-0.31153500
C	-1.44445900	2.82769300	0.32872200
C	-0.50163800	1.86771300	0.72938700
C	0.85590100	2.10686300	0.48016700
C	-0.93512100	0.64652000	1.42503500
C	-2.27528500	0.03306600	1.34956100
C	-2.99478300	0.03567500	0.14714400
C	-4.25361600	-0.54740100	0.05933500
C	-4.81070400	-1.15819500	1.18276800
C	-4.10484600	-1.18639700	2.38434800
C	-2.85392700	-0.59098200	2.46456500
O	-6.02775500	-1.79961000	1.16871300
C	-6.96334800	-1.45546300	0.20545800
C	-7.44924500	-0.15403300	0.10734600
C	-8.43078100	0.13728100	-0.83327400
C	-8.94912100	-0.85270100	-1.67664300
C	-8.44712800	-2.14995900	-1.55133100
C	-7.45728300	-2.45678700	-0.61920400
N	3.11860200	4.71611300	-0.59843100

N	-0.05458400	0.04305600	2.17788600
N	3.46216600	5.78948600	-0.76822500
N	0.71576800	-0.47871500	2.83670200
H	4.64089600	-4.19158700	1.22571100
H	5.05674900	-1.75589100	1.23295100
H	7.76875400	-2.19676100	-2.04542000
H	7.36432600	-4.64989000	-2.05083800
H	4.28173700	-0.75488400	-1.47516700
H	2.56901600	1.01574100	-1.49010700
H	5.23001400	3.60953000	0.65830200
H	6.96047200	1.83553800	0.63262700
H	0.63009500	5.12040200	-1.07978200
H	-1.75310500	4.72490200	-0.61699000
H	-2.49540100	2.67239700	0.53106500
H	1.59262700	1.37990200	0.79500800
H	-2.56043200	0.48830600	-0.73518200
H	-4.79229300	-0.53932800	-0.87890100
H	-4.55369000	-1.66532800	3.24547400
H	-2.32447400	-0.60140900	3.41052000
H	-7.05631900	0.61696100	0.75880200
H	-8.80621400	1.15240700	-0.90863600
H	-8.82982400	-2.93506700	-2.19450900
H	-7.06437100	-3.46141600	-0.52399000
C	5.68578400	-6.08554600	-0.43741200
H	5.62045400	-6.48835200	0.57640800
H	4.73559200	-6.30276000	-0.93667200
H	6.46924800	-6.63028400	-0.96788200
C	-10.03793500	-0.52939000	-2.66877000
H	-10.07637000	-1.26915700	-3.47087300
H	-11.01972700	-0.51716600	-2.18372500
H	-9.88525600	0.45344400	-3.12109900

(3) Bisdiazo-NO_2

C	-7.03412700	3.45018800	0.04134200
C	-6.05161200	2.63511800	0.59229300
C	-5.99079500	1.29763800	0.22570100
C	-6.91744600	0.79468300	-0.69269800
C	-7.90574900	1.62227600	-1.23663600
C	-7.96624100	2.95495300	-0.86926200
O	-6.94768400	-0.51130200	-1.09295300
C	-5.86967000	-1.33765900	-0.80190700
C	-4.64712800	-1.15015100	-1.43996300
C	-3.59642200	-2.01598300	-1.17078400
C	-3.74986000	-3.07240600	-0.25819000
C	-4.99664000	-3.24801600	0.35741800
C	-6.05598200	-2.38856200	0.08515000
C	-2.62171100	-3.97837500	0.02520800
C	-1.20699900	-3.58902000	0.12144600
C	-0.17472300	-4.52031600	-0.07407500
C	1.15148400	-4.12367400	0.04971700
C	1.48178700	-2.80800600	0.35578400
C	0.46475900	-1.85940800	0.54344100
C	-0.86982800	-2.26539200	0.42846000
C	0.79500900	-0.46680300	0.89200600
C	2.02470800	0.25554200	0.52715300
C	2.69728000	-0.04568200	-0.66858900
C	3.84753700	0.64293400	-1.03058000
C	4.33376100	1.64886400	-0.20256600
C	3.68044500	1.97751400	0.97720500
C	2.53493700	1.27852600	1.34060500
O	5.45083700	2.38456900	-0.58284200
C	6.69122300	1.83026300	-0.44928800
C	6.92906300	0.62342000	0.21546100
C	8.22853300	0.14533300	0.31597200

C	9.27040000	0.87460800	-0.24548800
C	9.04238600	2.08140800	-0.90557800
C	7.74802400	2.55902200	-1.00684500
N	-2.91268800	-5.23507200	0.23951700
N	-0.09529600	0.19569900	1.58389800
N	-3.16180600	-6.33007000	0.42451600
N	-0.87678000	0.76611700	2.18395800
H	-5.34996500	3.05116200	1.30147000
H	-5.23516200	0.65230800	0.65104200
H	-8.61023600	1.20157500	-1.94218800
H	-8.71735400	3.61696500	-1.27615500
H	-4.52859100	-0.33814400	-2.14685700
H	-2.65157600	-1.88226000	-1.68129700
H	-5.13879800	-4.05365100	1.06823000
H	-7.01852900	-2.51660400	0.56399200
H	-0.40693500	-5.54782900	-0.32696600
H	1.94131600	-4.85192500	-0.09300300
H	2.51885000	-2.52122300	0.46585500
H	-1.66119400	-1.54466100	0.58637400
H	2.30646400	-0.81106200	-1.32592100
H	4.36155900	0.41545500	-1.95644800
H	4.07580500	2.76718400	1.60367200
H	2.03870600	1.52784900	2.27116800
H	6.11002700	0.06864300	0.65107800
H	8.44381800	-0.78309600	0.82582400
H	9.87635500	2.62301600	-1.32887300
H	7.53101500	3.49086700	-1.51249200
N	-7.09236300	4.86481400	0.42914100
O	-7.97586600	5.55644800	-0.06615700
O	-6.25433700	5.27440500	1.22547800
N	10.64182100	0.36279200	-0.13922100
O	10.81057200	-0.70461600	0.44122300
O	11.54095100	1.03155300	-0.63757900

(4) Bisdiazo-NH_2

C	5.71530800	-4.81032300	-0.34973100
C	5.24247100	-4.08409000	0.75342000
C	5.53195600	-2.73233400	0.89068200
C	6.29067100	-2.08494200	-0.07858800
C	6.76619800	-2.78587300	-1.17847500
C	6.48248400	-4.14128000	-1.31264300
O	6.63173900	-0.74495100	0.07308400
C	5.66641300	0.20794800	-0.14346300
C	4.42862100	-0.06281000	-0.72736100
C	3.52027700	0.97219500	-0.92142000
C	3.81532800	2.28238400	-0.52614000
C	5.06811700	2.53226600	0.05283800
C	5.98861500	1.51124600	0.23683700
C	2.83348900	3.36824100	-0.72801400
C	1.38582600	3.27303300	-0.49740800
C	0.48804300	4.18450900	-1.07876000
C	-0.87349300	4.07490200	-0.82473300
C	-1.37456200	3.06716200	-0.00788000
C	-0.49540300	2.14095500	0.57577200
C	0.87790900	2.26003100	0.32496300
C	-1.01186800	1.08430800	1.45806800
C	-2.39168800	0.55847800	1.46672600
C	-3.09667900	0.38105500	0.27047800
C	-4.39418800	-0.12149100	0.26320800
C	-5.00269700	-0.47506900	1.46742700
C	-4.31203800	-0.31814500	2.67005100
C	-3.02601900	0.19983700	2.66615100
O	-6.27738900	-0.97967100	1.56215300
C	-6.99849600	-1.18537400	0.39045200

C	-7.85451100	-0.19525300	-0.07497300
C	-8.61567000	-0.41793600	-1.21703400
C	-8.53073300	-1.63507700	-1.90773600
C	-7.66163200	-2.62236900	-1.42154000
C	-6.90180900	-2.39910100	-0.27908300
N	3.30261200	4.51911700	-1.12855000
N	-0.17893700	0.55971900	2.31633400
N	3.71004900	5.52662900	-1.47339500
N	0.54849500	0.10682700	3.06867200
H	4.64606300	-4.58429400	1.50887800
H	5.16900300	-2.17532500	1.74595300
H	7.35934100	-2.26685200	-1.92131600
H	6.85514800	-4.68496400	-2.17411300
H	4.18002000	-1.06907000	-1.03605600
H	2.56876600	0.75715200	-1.39151200
H	5.32040300	3.53628600	0.37494600
H	6.95388600	1.70129600	0.68917200
H	0.85199300	4.96960700	-1.73052400
H	-1.55782100	4.78744100	-1.27103900
H	-2.43595700	3.00515400	0.18973300
H	1.56667400	1.55911800	0.77754400
H	-2.62481100	0.63362200	-0.67073900
H	-4.92147500	-0.24868200	-0.67242200
H	-4.80240500	-0.59379100	3.59532000
H	-2.50817000	0.33424700	3.60913500
H	-7.92190400	0.74356000	0.46109200
H	-9.28180900	0.35812700	-1.57839900
H	-7.58196200	-3.57021700	-1.94293400
H	-6.23298200	-3.16291600	0.09855000
N	5.37814900	-6.15701100	-0.51350700
N	-9.25204200	-1.83799600	-3.08706900
H	-10.08050100	-1.27151200	-3.19306100
H	-9.41815800	-2.80210400	-3.33483300
H	6.00193700	-6.69198100	-1.09941200
H	5.14020200	-6.65039100	0.33420400

2.2.2 Optimized geometry of compounds in varied solvents by SMD model

(1) THF as solvent

Bisdiazo-H

C	5.82707200	-4.95491400	-0.24307300
C	5.13680500	-4.06411600	0.57010300
C	5.37021500	-2.69888300	0.48060900
C	6.29751900	-2.23045600	-0.44136800
C	6.99602000	-3.10738700	-1.25757500
C	6.75916700	-4.47124700	-1.15249500
O	6.61789200	-0.89434900	-0.53810800
C	5.62477400	0.05200100	-0.44935400
C	4.40240700	-0.09979300	-1.09191600
C	3.46130700	0.91219800	-1.01637200
C	3.72021400	2.08298900	-0.29968700
C	4.95926700	2.21896900	0.32639600
C	5.91002600	1.21338500	0.25031900
C	2.70992200	3.15821000	-0.22728200
C	1.25813300	2.97774800	-0.07460500
C	0.35872100	3.98170200	-0.44612500
C	-1.00250900	3.78988200	-0.27195400
C	-1.49568700	2.60863100	0.25949100
C	-0.61172200	1.59124200	0.62893600
C	0.75857600	1.79013100	0.45922100
C	-1.11830700	0.34017100	1.21768200
C	-2.45886400	-0.24221300	1.01380500
C	-3.10096100	-0.13327800	-0.22212900

C	-4.35229000	-0.68886600	-0.42726400
C	-4.97908400	-1.36840700	0.60953900
C	-4.35349400	-1.50196500	1.83868100
C	-3.10346600	-0.93819700	2.03742200
O	-6.19229100	-1.99437200	0.44590100
C	-7.19363600	-1.36948500	-0.26355600
C	-7.46048700	-0.01401000	-0.11930600
C	-8.52354800	0.54321500	-0.81632000
C	-9.31911900	-0.24310300	-1.64096500
C	-9.04574700	-1.59933100	-1.76602400
C	-7.98060100	-2.16729100	-1.08040700
N	3.15154400	4.37685800	-0.27952600
N	-0.31204000	-0.31301800	1.99646900
N	3.53563800	5.44226600	-0.32350200
N	0.39284100	-0.88128800	2.67849800
H	5.64078400	-6.01918600	-0.16633500
H	4.41337500	-4.43218400	1.28808300
H	4.83792000	-2.00568900	1.11964000
H	7.71636300	-2.71333900	-1.96384200
H	7.30431700	-5.15725700	-1.79002800
H	4.18940900	-0.99975500	-1.65481600
H	2.51762900	0.79263800	-1.53335600
H	5.18374900	3.11503900	0.89379000
H	6.86930100	1.31646900	0.74207600
H	0.71808900	4.90862100	-0.87711700
H	-1.69232000	4.57582300	-0.55614200
H	-2.56110800	2.48243800	0.39766800
H	1.45088600	1.01035600	0.74865200
H	-2.61220900	0.37829500	-1.04133100
H	-4.83346600	-0.60199700	-1.39324100
H	-4.85504800	-2.03724000	2.63526800
H	-2.63161400	-1.03392700	3.00864800
H	-6.84557300	0.59679000	0.52963300
H	-8.73416900	1.60047800	-0.70579200
H	-10.14828600	0.19860000	-2.18004200
H	-9.66113400	-2.22208200	-2.40452900
H	-7.75131600	-3.22204900	-1.16935900

Bisdiazo-Me

C	-5.78568900	4.71379400	-0.32460800
C	-5.26624000	3.87573700	0.66454400
C	-5.54087400	2.51840500	0.67635600
C	-6.34015500	1.97879500	-0.32262400
C	-6.86929600	2.78348000	-1.31358200
C	-6.59114900	4.14628500	-1.30639800
O	-6.68114200	0.63851700	-0.30917400
C	-5.68935300	-0.30488800	-0.37312500
C	-4.44373800	-0.05961700	-0.93790000
C	-3.50801900	-1.07999000	-0.99826500
C	-3.78995000	-2.34922800	-0.49241400
C	-5.05209700	-2.57632300	0.05966200
C	-5.99780100	-1.56745900	0.11538500
C	-2.78198800	-3.42799700	-0.55471000
C	-1.33913500	-3.28531700	-0.30794400
C	-0.42500200	-4.23348900	-0.77806800
C	0.92623500	-4.08233000	-0.51150800
C	1.39636000	-2.99611400	0.21007200
C	0.49801800	-2.03444300	0.68054600
C	-0.86292700	-2.19358700	0.41699400
C	0.97624500	-0.88563700	1.46692900
C	2.33543600	-0.30937900	1.44000600
C	3.06120400	-0.24561500	0.25192900
C	4.33376200	0.30587800	0.21366900
C	4.89346300	0.81827500	1.37704800
C	4.18011000	0.77662200	2.56967700

C	2.91857800	0.21210700	2.59838000
O	6.13203100	1.39852700	1.43304000
C	6.95703800	1.32959900	0.32249700
C	7.69384800	0.18044400	0.08053000
C	8.55366900	0.14314700	-1.00603400
C	8.69469400	1.24152200	-1.85565400
C	7.94453000	2.38252700	-1.58491800
C	7.07569300	2.43342700	-0.50112000
N	-3.21817300	-4.61940700	-0.82382000
N	0.12179200	-0.32268200	2.26403100
N	-3.59594300	-5.66275300	-1.05637100
N	-0.62649400	0.16493500	2.96289200
H	-4.63987300	4.29544300	1.44449300
H	-5.13641700	1.87790400	1.45102400
H	-7.49350100	2.34371700	-2.08193200
H	-7.00831000	4.77570300	-2.08460200
H	-4.20436900	0.91684000	-1.33781300
H	-2.54649600	-0.88195900	-1.45515700
H	-5.29767000	-3.55154800	0.46476100
H	-6.97382300	-1.74332200	0.55041300
H	-0.76522700	-5.08451300	-1.35614700
H	1.62737800	-4.82508100	-0.87364800
H	2.45405500	-2.90323400	0.41621500
H	-1.56620900	-1.45736300	0.78304000
H	2.62914300	-0.62403600	-0.66575500
H	4.87483300	0.34303300	-0.72202100
H	4.62984600	1.17860300	3.46913700
H	2.38243700	0.17102600	3.53990700
H	7.58905000	-0.67372800	0.73899700
H	9.12966800	-0.75603900	-1.19649800
H	8.03724400	3.24830400	-2.23116700
H	6.49131100	3.32175500	-0.29370800
C	-5.48670800	6.18748800	-0.31284400
H	-5.92993600	6.67097400	0.56210800
H	-4.41030200	6.37186200	-0.27495100
H	-5.88326900	6.67736500	-1.20319000
C	9.65087200	1.19499500	-3.01519400
H	9.46548000	2.01194100	-3.71397500
H	10.68563800	1.27998000	-2.67077400
H	9.56612800	0.25267700	-3.56069400

Bisdiazo-NO_2

C	7.29065300	-3.33038600	0.10251100
C	6.27828600	-2.55362100	0.64252800
C	6.12060900	-1.24917300	0.21171000
C	6.98158700	-0.74186900	-0.75885700
C	8.00140100	-1.53116600	-1.29128500
C	8.15864500	-2.83013200	-0.86098200
O	6.91003600	0.52766700	-1.23257700
C	5.83292200	1.32929200	-0.88389300
C	4.60516200	1.14160000	-1.49756200
C	3.55176100	1.98034800	-1.17774500
C	3.71433100	3.00702200	-0.24266300
C	4.96559000	3.18053100	0.35132800
C	6.02675600	2.34799400	0.02985800
C	2.58919400	3.90169400	0.08581600
C	1.16724200	3.52713400	0.15361500
C	0.16020200	4.47616100	-0.04006400
C	-1.17103400	4.10201300	0.05348100
C	-1.52570600	2.79090200	0.32877700
C	-0.53120300	1.82758000	0.51644900
C	0.80700400	2.20869300	0.42931900
C	-0.88791600	0.43443600	0.83828200
C	-2.11745400	-0.26975000	0.43501800

C	-2.75134000	0.04697600	-0.77004800
C	-3.89948700	-0.62022400	-1.16216000
C	-4.41664000	-1.61650200	-0.35150600
C	-3.80067300	-1.96212700	0.83700600
C	-2.65719000	-1.28422200	1.22921500
O	-5.53714200	-2.32452800	-0.76386200
C	-6.77145300	-1.80709900	-0.54279600
C	-6.99526200	-0.63210800	0.17127700
C	-8.29133300	-0.18547500	0.35232900
C	-9.34256900	-0.91475200	-0.17913900
C	-9.12874200	-2.09011400	-0.88992800
C	-7.83810600	-2.53542500	-1.07091400
N	2.88526100	5.13512500	0.36226700
N	-0.03373700	-0.23885800	1.54744600
N	3.14150300	6.21142400	0.60324300
N	0.71358300	-0.82295100	2.16623000
H	5.62031400	-2.96484900	1.39490600
H	5.33611000	-0.63566000	0.63148700
H	8.65790500	-1.11008300	-2.04155700
H	8.94187500	-3.45532600	-1.26523300
H	4.47948800	0.34891600	-2.22518400
H	2.59818800	1.84295700	-1.67057600
H	5.11604400	3.96737000	1.08135600
H	6.99696800	2.47888300	0.49274900
H	0.41416600	5.50422800	-0.27004700
H	-1.94594400	4.84560800	-0.09049200
H	-2.57003400	2.52034100	0.41021000
H	1.58212600	1.46879400	0.58232600
H	-2.33783800	0.81077500	-1.41516300
H	-4.38867400	-0.37719700	-2.09765100
H	-4.22020700	-2.74739900	1.45353900
H	-2.18665200	-1.54706000	2.16947500
H	-6.16995600	-0.07016800	0.58477000
H	-8.48510300	0.72315600	0.90440900
H	-9.96588600	-2.64145600	-1.29371200
H	-7.63500600	-3.44544600	-1.62025300
N	7.45053800	-4.70584900	0.55741300
O	8.35012500	-5.37282700	0.07219500
O	6.67791500	-5.12882800	1.40209500
N	-10.70647700	-0.43851000	0.01300700
O	-10.87494500	0.59605500	0.63822300
O	-11.61898900	-1.09609300	-0.46063200

Bisdiazo-NH_2

C	-6.11271600	4.69126700	-0.48319200
C	-5.68604500	4.16228900	0.73849700
C	-5.84871900	2.81532200	1.02147600
C	-6.43754300	1.98146700	0.08615100
C	-6.87203000	2.48656100	-1.12731900
C	-6.71025200	3.83353900	-1.41131800
O	-6.64483300	0.64232600	0.38851800
C	-5.66845800	-0.26312300	0.07883700
C	-4.46558000	0.07414700	-0.52984500
C	-3.53681800	-0.91888700	-0.80547800
C	-3.77871200	-2.24931500	-0.46948000
C	-4.99377100	-2.56618800	0.14317600
C	-5.93375600	-1.58802700	0.40908200
C	-2.78338200	-3.29980300	-0.77246300
C	-1.32836800	-3.19981200	-0.58783700
C	-0.45242200	-4.07234600	-1.24249900
C	0.91274000	-3.96730500	-1.03220200
C	1.43470300	-3.00141700	-0.18590000
C	0.57524600	-2.11622500	0.47067900
C	-0.80043500	-2.22865500	0.26206100
C	1.11225200	-1.10138800	1.38979100

C	2.49745300	-0.58736600	1.41528100
C	3.18472000	-0.32453100	0.23206400
C	4.48077400	0.17083100	0.24645600
C	5.10322500	0.42664800	1.46216500
C	4.42903000	0.18124000	2.65384000
C	3.14378900	-0.32687600	2.62664200
O	6.37454600	0.91668600	1.57857000
C	7.09350500	1.19637000	0.42429900
C	7.92171800	0.22976100	-0.11997800
C	8.67844100	0.52297500	-1.24367000
C	8.61673300	1.78818000	-1.83486400
C	7.77800500	2.75161900	-1.26684900
C	7.02206700	2.45733600	-0.14309100
N	-3.24436000	-4.42451100	-1.22400900
N	0.29718700	-0.61508100	2.27367000
N	-3.64388800	-5.41104300	-1.61530700
N	-0.41787500	-0.19548200	3.04736300
H	-5.22515600	4.81565200	1.47101500
H	-5.51807100	2.40863200	1.96989100
H	-7.33678200	1.82397700	-1.84786600
H	-7.05090800	4.22915900	-2.36171800
H	-4.25206800	1.09962100	-0.79849200
H	-2.61055100	-0.64597200	-1.29565000
H	-5.20670600	-3.59211400	0.42233900
H	-6.87448100	-1.83538300	0.88535800
H	-0.83369300	-4.82803000	-1.91892900
H	1.58426500	-4.65144500	-1.53767600
H	2.50294400	-2.94368000	-0.02731800
H	-1.47408400	-1.55271800	0.77208700
H	2.70403800	-0.50195400	-0.72187500
H	4.99245400	0.36515200	-0.68623900
H	4.92774800	0.38231500	3.59398500
H	2.63658100	-0.52886100	3.56355300
H	7.97334400	-0.75113800	0.33755100
H	9.32797300	-0.23372600	-1.66973300
H	7.72255000	3.73925600	-1.71105300
H	6.37461800	3.20714000	0.29604700
N	-5.90018900	6.03237300	-0.78937400
N	9.33552600	2.06669000	-2.99382900
H	10.15285200	1.49182200	-3.14357200
H	9.52369200	3.04539600	-3.16045400
H	-6.51619700	6.41804200	-1.49124500
H	-5.79924000	6.64640700	0.00662100

(2) Toluene as solvent

Bisdiazo-H

C	5.78796700	-4.96929800	-0.27965900
C	5.09967900	-4.08954400	0.54626300
C	5.34454100	-2.72536100	0.48479400
C	6.28092700	-2.24636400	-0.42199600
C	6.97742600	-3.11256800	-1.25037700
C	6.72930500	-4.47582000	-1.17323100
O	6.61093300	-0.91117300	-0.48995300
C	5.62274000	0.03939300	-0.42166100
C	4.40530500	-0.10931000	-1.07412600
C	3.46943400	0.90826900	-1.01401200
C	3.72651000	2.08181900	-0.30203700
C	4.96119000	2.21502000	0.33294000
C	5.90726200	1.20495000	0.27165000
C	2.71902100	3.16023800	-0.24092100
C	1.26782000	2.98479200	-0.08327600
C	0.36966900	3.99146400	-0.45008200
C	-0.99095200	3.80365900	-0.27129200
C	-1.48623900	2.62368300	0.25999500

C	-0.60466600	1.60292100	0.62486600
C	0.76528500	1.79859400	0.45046100
C	-1.11150600	0.35202800	1.21268000
C	-2.45398200	-0.22746600	1.01553400
C	-3.10548100	-0.11298800	-0.21456300
C	-4.35839900	-0.66688000	-0.41351900
C	-4.97842800	-1.35193800	0.62358200
C	-4.34325100	-1.49087600	1.84729000
C	-3.09256100	-0.92806300	2.03976100
O	-6.19150800	-1.97707400	0.46882700
C	-7.19134300	-1.36494200	-0.25240200
C	-7.47162800	-0.01159400	-0.11668000
C	-8.53262500	0.53333300	-0.82540100
C	-9.31329900	-0.26322400	-1.65351600
C	-9.02715500	-1.61699400	-1.77011500
C	-7.96382200	-2.17257000	-1.07271900
N	3.16374300	4.37826200	-0.30507300
N	-0.30027200	-0.30709500	1.98296400
N	3.54898500	5.44240300	-0.35920400
N	0.40878100	-0.87948400	2.65665000
H	5.59320000	-6.03321100	-0.22465100
H	4.36922600	-4.46579300	1.25255200
H	4.81485900	-2.03929100	1.13341800
H	7.70537400	-2.70855800	-1.94266000
H	7.27320800	-5.15393700	-1.81995100
H	4.19365300	-1.01211100	-1.63246700
H	2.52984400	0.79184400	-1.53898400
H	5.18426200	3.11387900	0.89641300
H	6.86350700	1.30419500	0.76950000
H	0.73060600	4.91786300	-0.88064300
H	-1.67903800	4.59251800	-0.55114800
H	-2.55111000	2.50035600	0.40366000
H	1.45652600	1.01693600	0.73711600
H	-2.62104000	0.40175700	-1.03425600
H	-4.84714700	-0.57610900	-1.37508300
H	-4.84074000	-2.03126200	2.64265500
H	-2.61343600	-1.02804100	3.00695500
H	-6.86730600	0.60542100	0.53609200
H	-8.75412200	1.58886700	-0.72117800
H	-10.14139900	0.16866300	-2.20173600
H	-9.63184400	-2.24744600	-2.41102000
H	-7.72361000	-3.22536000	-1.15232000

Bisdiazo-Me

C	-5.81717200	4.72540300	-0.32785900
C	-5.27932700	3.90863100	0.66845800
C	-5.54608800	2.55074800	0.70910400
C	-6.35608000	1.98846600	-0.26800400
C	-6.90318100	2.77224700	-1.26562200
C	-6.63265500	4.13576900	-1.28745400
O	-6.68839400	0.64772700	-0.22558400
C	-5.69784900	-0.29269700	-0.31925300
C	-4.46105100	-0.04178500	-0.90016700
C	-3.52741600	-1.06194000	-0.98704200
C	-3.80105200	-2.33652600	-0.49163300
C	-5.05408900	-2.56829300	0.07877800
C	-5.99821500	-1.56072200	0.16018500
C	-2.79509300	-3.41487800	-0.58381400
C	-1.35030000	-3.27808700	-0.34880500
C	-0.44186600	-4.21967200	-0.84227200
C	0.91167200	-4.07346400	-0.58795900
C	1.39053300	-2.99891300	0.14431600
C	0.49873700	-2.04388400	0.63981400
C	-0.86487700	-2.19838400	0.38757300
C	0.98796700	-0.91000900	1.43917600

C	2.35829200	-0.35904100	1.43032900
C	3.07929800	-0.25506400	0.24273300
C	4.36276000	0.27103900	0.22212300
C	4.93865100	0.71739900	1.40448800
C	4.23043200	0.63506200	2.59780900
C	2.95824400	0.09586600	2.60753000
O	6.19036100	1.26404700	1.48308700
C	6.99276200	1.27596700	0.35578300
C	7.73272800	0.15262900	0.02323500
C	8.57029300	0.19446600	-1.08044300
C	8.68619500	1.34678200	-1.85771600
C	7.93343900	2.46078600	-1.49695600
C	7.08782600	2.43281000	-0.39546200
N	-3.23513800	-4.60090400	-0.87411300
N	0.13761400	-0.33981100	2.23705800
N	-3.61465500	-5.63910000	-1.12470700
N	-0.60684800	0.15358800	2.93539700
H	-4.64469700	4.34581600	1.43183000
H	-5.12845100	1.92605100	1.48936100
H	-7.53617800	2.31303400	-2.01478600
H	-7.06472800	4.74830600	-2.07081500
H	-4.22869300	0.93938100	-1.29192300
H	-2.57296700	-0.86069800	-1.45703100
H	-5.29245100	-3.54782500	0.47756300
H	-6.96792800	-1.73850800	0.60772300
H	-0.78961600	-5.06160500	-1.42892200
H	1.60818400	-4.81152300	-0.96794700
H	2.44994400	-2.90916100	0.34203300
H	-1.56477800	-1.46899000	0.77323700
H	2.63289700	-0.58237100	-0.68773400
H	4.90110300	0.34181100	-0.71306200
H	4.69515300	0.98630000	3.51045800
H	2.42488700	0.02147400	3.54852300
H	7.64709000	-0.74291600	0.62670600
H	9.14891000	-0.68507300	-1.34111600
H	8.00714600	3.36852400	-2.08544300
H	6.50239300	3.30003000	-0.11557100
C	-5.52773300	6.20111900	-0.34541600
H	-5.97178800	6.69942900	0.52069100
H	-4.45272300	6.39448200	-0.31605400
H	-5.92983900	6.67271700	-1.24307800
C	9.62210300	1.38855700	-3.03409400
H	9.36503900	2.19916900	-3.71754000
H	10.65377000	1.54727600	-2.70668900
H	9.59842400	0.45194700	-3.59465500

Bisdiazo-NO_2

C	7.30651400	-3.33203500	0.11147400
C	6.29980500	-2.55247700	0.65539800
C	6.12919500	-1.25464800	0.20797300
C	6.97231000	-0.75757600	-0.78261800
C	7.98633500	-1.55048100	-1.31951600
C	8.15620900	-2.84242600	-0.87252500
O	6.88816200	0.50488600	-1.27487500
C	5.82067900	1.31140100	-0.91458800
C	4.58492500	1.13099800	-1.51414500
C	3.53937400	1.97486400	-1.18341000
C	3.71562000	3.00060900	-0.24985600
C	4.97454400	3.16694800	0.32986000
C	6.02802800	2.33006300	-0.00360800
C	2.59858900	3.90002900	0.09046500
C	1.17475800	3.53496100	0.16277400
C	0.17283700	4.48987100	-0.02655100
C	-1.16004200	4.12420400	0.06998500
C	-1.52227500	2.81534300	0.34396900

C	-0.53395500	1.84539300	0.52794300
C	0.80589600	2.21890400	0.43754100
C	-0.89699100	0.45403000	0.84930400
C	-2.12515200	-0.24831700	0.44118900
C	-2.76088100	0.07630000	-0.76075000
C	-3.90599900	-0.59184300	-1.15910500
C	-4.42062600	-1.59768500	-0.35865700
C	-3.80252700	-1.95059700	0.82667300
C	-2.66246000	-1.27170400	1.22555900
O	-5.53565600	-2.30680500	-0.77810800
C	-6.77450300	-1.80091200	-0.55181800
C	-7.00874200	-0.63670600	0.17548700
C	-8.30900800	-0.20233000	0.36020600
C	-9.35317500	-0.93279500	-0.18082200
C	-9.12924300	-2.09734500	-0.90468900
C	-7.83477600	-2.53070400	-1.08938100
N	2.90437900	5.13112300	0.37292800
N	-0.04498100	-0.22203000	1.56064800
N	3.16876600	6.20372000	0.61881700
N	0.69990400	-0.80744600	2.18029200
H	5.65789100	-2.95973900	1.42354800
H	5.34903000	-0.63714000	0.62978500
H	8.62732300	-1.13504400	-2.08589300
H	8.93413100	-3.47388800	-1.27710500
H	4.44931600	0.34039000	-2.24195100
H	2.58016200	1.84449800	-1.66690000
H	5.13635500	3.95293600	1.05828300
H	7.00486300	2.45644900	0.44604000
H	0.43278100	5.51649400	-0.25584000
H	-1.93032900	4.87310600	-0.07005500
H	-2.56767200	2.55064100	0.42976200
H	1.57690500	1.47427900	0.58846600
H	-2.34824100	0.84639500	-1.39871200
H	-4.39508600	-0.34551100	-2.09357700
H	-4.21888800	-2.74490500	1.43332500
H	-2.19067100	-1.54103500	2.16328700
H	-6.18699300	-0.07477600	0.59581800
H	-8.51559900	0.69741100	0.92196500
H	-9.96462000	-2.64723800	-1.31391200
H	-7.62111200	-3.43218600	-1.64832000
N	7.48034400	-4.70304300	0.58492200
O	8.37847700	-5.36747000	0.09736800
O	6.71881800	-5.11466200	1.44295900
N	-10.72397800	-0.46796000	0.01533000
O	-10.89687400	0.55565400	0.65432800
O	-11.62694600	-1.12681600	-0.47056400

Bisdiazo-NH_2

C	-5.99844100	4.71713900	-0.44098200
C	-5.55372300	4.10773200	0.73536500
C	-5.76068200	2.75592800	0.95368600
C	-6.40865100	1.99422800	-0.00429300
C	-6.85817900	2.57921700	-1.17468700
C	-6.65459200	3.93290300	-1.39234100
O	-6.66206300	0.65152900	0.23191000
C	-5.67994500	-0.26311000	-0.02655600
C	-4.46799700	0.05221400	-0.62825600
C	-3.53767700	-0.95041300	-0.85801400
C	-3.78611000	-2.26872200	-0.48210300
C	-5.01123900	-2.56315200	0.12110100
C	-5.95316500	-1.57610700	0.34066500
C	-2.78731200	-3.32933700	-0.73276800
C	-1.33588000	-3.22472500	-0.52675500
C	-0.45004000	-4.11810800	-1.13795700
C	0.91130300	-4.00676700	-0.91009600

C	1.42073500	-3.01375400	-0.08885100
C	0.55226900	-2.10693000	0.52444000
C	-0.81975900	-2.22636400	0.29848800
C	1.07659300	-1.06176900	1.41635200
C	2.45623600	-0.53385500	1.43011800
C	3.15025400	-0.31403700	0.24230100
C	4.44200900	0.19195800	0.24470300
C	5.05395400	0.50291500	1.45240800
C	4.37294700	0.30218100	2.64808700
C	3.09273200	-0.21738000	2.63323800
O	6.31942200	1.00951100	1.55794100
C	7.05774200	1.21806200	0.40298000
C	7.87692100	0.21265200	-0.08104300
C	8.65351700	0.43657500	-1.20631900
C	8.62323200	1.67134800	-1.85949400
C	7.79355400	2.67477500	-1.35268200
C	7.01701400	2.44963400	-0.22748800
N	-3.24204800	-4.46906900	-1.15493000
N	0.25325900	-0.55839600	2.28427100
N	-3.63469100	-5.46797600	-1.52000700
N	-0.46762400	-0.12442800	3.04407500
H	-5.04311600	4.70164600	1.48516100
H	-5.41857000	2.28696400	1.86842300
H	-7.36894700	1.97186500	-1.91199700
H	-7.00694900	4.38936100	-2.31063200
H	-4.25062500	1.06868600	-0.92605000
H	-2.60403000	-0.69650400	-1.34428200
H	-5.22862100	-3.57901200	0.43165800
H	-6.90171900	-1.80360900	0.81060900
H	-0.82161000	-4.89525000	-1.79506700
H	1.58976700	-4.70801100	-1.38147800
H	2.48596400	-2.95060100	0.08646100
H	-1.50129700	-1.53425800	0.77546400
H	2.67660400	-0.53465700	-0.70599900
H	4.95999600	0.35268200	-0.69077400
H	4.86558700	0.54672200	3.58077400
H	2.58090200	-0.38551800	3.57418500
H	7.90488200	-0.74363900	0.42716500
H	9.29300800	-0.35310400	-1.58478700
H	7.75963700	3.63967000	-1.84629000
H	6.37714000	3.23006800	0.16630100
N	-5.74731900	6.06531800	-0.68061700
N	9.36479800	1.88079600	-3.01869700
H	10.17010800	1.28432500	-3.13726900
H	9.56603900	2.84445900	-3.24087100
H	-6.35978400	6.51227600	-1.34655000
H	-5.59360500	6.63381200	0.13889500

(3) Chlorobenzene as solvent

Bisdiazo-H

C	5.84333500	-4.95500800	-0.24550900
C	5.14778400	-4.06838000	0.56764000
C	5.37704200	-2.70220000	0.48196300
C	6.30546500	-2.22865300	-0.43622400
C	7.00919200	-3.10146500	-1.25238700
C	6.77652000	-4.46626400	-1.15108800
O	6.62218500	-0.89172700	-0.52931300
C	5.62832000	0.05338200	-0.44367400
C	4.40719300	-0.09914900	-1.08840200
C	3.46544700	0.91248000	-1.01497300
C	3.72213800	2.08363500	-0.29812800
C	4.96027400	2.22044300	0.32984300
C	5.91168200	1.21545600	0.25584100
C	2.71113300	3.15850800	-0.22687300

C	1.25911000	2.97895600	-0.07473100
C	0.36075800	3.98572800	-0.44155400
C	-1.00060800	3.79502900	-0.26780400
C	-1.49538500	2.61219000	0.25866100
C	-0.61285300	1.59188700	0.62340700
C	0.75769700	1.78985500	0.45399700
C	-1.12001500	0.33856100	1.20714100
C	-2.46138100	-0.24266000	1.00446600
C	-3.10899200	-0.12577000	-0.22784800
C	-4.36086200	-0.68085200	-0.43154300
C	-4.98278500	-1.36816300	0.60304700
C	-4.35171100	-1.50965300	1.82861300
C	-3.10139500	-0.94629100	2.02594000
O	-6.19556500	-1.99437400	0.44114400
C	-7.20179400	-1.37006100	-0.26117000
C	-7.47355400	-0.01635800	-0.10989300
C	-8.54164200	0.53961300	-0.80019300
C	-9.33734400	-0.24632000	-1.62499200
C	-9.05908200	-1.60089000	-1.75704700
C	-7.98890700	-2.16747200	-1.07830800
N	3.15304800	4.37730300	-0.27794900
N	-0.31210700	-0.31907900	1.98079900
N	3.53721000	5.44263700	-0.32094100
N	0.39407600	-0.89112900	2.65817600
H	5.66034700	-6.01998100	-0.17169800
H	4.42351600	-4.44041100	1.28261800
H	4.84077500	-2.01192900	1.12070800
H	7.73027400	-2.70277800	-1.95516600
H	7.32578800	-5.14906600	-1.78841200
H	4.19593500	-0.99959500	-1.65101400
H	2.52276700	0.79243700	-1.53349700
H	5.18306000	3.11704600	0.89699900
H	6.87028700	1.31862300	0.74871800
H	0.72142100	4.91407200	-0.86824800
H	-1.68932300	4.58321600	-0.54815100
H	-2.56080300	2.48674300	0.39678200
H	1.44898500	1.00792500	0.73975500
H	-2.62390200	0.39174500	-1.04538400
H	-4.84652800	-0.58774600	-1.39459600
H	-4.84996200	-2.05119900	2.62293700
H	-2.62504200	-1.04841600	2.99428600
H	-6.85823100	0.59361300	0.53931400
H	-8.75615900	1.59543200	-0.68416500
H	-10.17046100	0.19431700	-2.15867400
H	-9.67467700	-2.22324800	-2.39561700
H	-7.75530400	-3.22079000	-1.17203500

Bisdiazo-Me

C	-5.81716300	4.72441600	-0.31319400
C	-5.30279100	3.89814400	0.68835000
C	-5.57090000	2.53957600	0.71004400
C	-6.35809800	1.98708200	-0.29125500
C	-6.88172400	2.77989300	-1.29469800
C	-6.61024600	4.14394700	-1.29760700
O	-6.69208100	0.64538800	-0.26780600
C	-5.69948000	-0.29513500	-0.34712100
C	-4.45344400	-0.04169000	-0.90710100
C	-3.51759100	-1.06129900	-0.98181800
C	-3.79924500	-2.33778700	-0.49478700
C	-5.06189900	-2.57247100	0.05371300
C	-6.00765100	-1.56492300	0.12327100
C	-2.79254300	-3.41670500	-0.57379400
C	-1.34727700	-3.27991000	-0.33672800
C	-0.43975800	-4.22488800	-0.82599300
C	0.91402000	-4.08050500	-0.56937300

C	1.39330600	-3.00431100	0.16108400
C	0.50189900	-2.04612800	0.65165000
C	-0.86184700	-2.19867000	0.39764900
C	0.99073400	-0.90942300	1.44859900
C	2.35851200	-0.35187500	1.43451700
C	3.08297900	-0.26450900	0.24731800
C	4.36377300	0.26863900	0.22212500
C	4.93298700	0.73858100	1.39877100
C	4.22107600	0.67302300	2.59134700
C	2.95136700	0.12694500	2.60624300
O	6.18167700	1.29347800	1.47161100
C	6.98848200	1.28983800	0.34602300
C	7.74059400	0.16641600	0.04081700
C	8.58250800	0.19312500	-1.06046300
C	8.68981100	1.33031200	-1.86200900
C	7.92454700	2.44464500	-1.52808000
C	7.07443600	2.43171100	-0.42896800
N	-3.23236200	-4.60492400	-0.85190300
N	0.14105000	-0.34234100	2.24813600
N	-3.61333300	-5.64528400	-1.09223800
N	-0.60309600	0.14901400	2.94869300
H	-4.68583700	4.32818300	1.47006200
H	-5.17080700	1.90771000	1.49392700
H	-7.49635300	2.32916400	-2.06431000
H	-7.02282700	4.76423400	-2.08543300
H	-4.21381000	0.94047900	-1.29223600
H	-2.55601200	-0.85650200	-1.43531900
H	-5.30767200	-3.55349600	0.44428900
H	-6.98412500	-1.74649300	0.55474000
H	-0.78767600	-5.06787400	-1.41110700
H	1.61005600	-4.82055500	-0.94641900
H	2.45288800	-2.91624200	0.35881900
H	-1.56028300	-1.46583300	0.77921700
H	2.64326700	-0.61054000	-0.67947400
H	4.90424600	0.32497200	-0.71286700
H	4.67898200	1.04229500	3.50054500
H	2.41542300	0.06699300	3.54681900
H	7.66130200	-0.71779000	0.66195300
H	9.17046200	-0.68607100	-1.30072600
H	7.99131600	3.33990900	-2.13622900
H	6.47895300	3.29924200	-0.17148400
C	-5.52804900	6.20028900	-0.31069000
H	-6.00663500	6.69240500	0.54054100
H	-4.45517200	6.39293900	-0.23596800
H	-5.89517700	6.67571400	-1.22122800
C	9.62813500	1.35499800	-3.03693800
H	9.38468000	2.16933900	-3.72084400
H	10.66158300	1.49647400	-2.70726400
H	9.58989000	0.41708100	-3.59469400

Bisdiazo-NO_2

C	7.31631600	-3.32625200	0.10512400
C	6.30307100	-2.55287500	0.64807400
C	6.13583200	-1.25081400	0.21313800
C	6.98827200	-0.74276600	-0.76444900
C	8.00917100	-1.52867100	-1.29983200
C	8.17594400	-2.82511700	-0.86533800
O	6.90719800	0.52406300	-1.24352000
C	5.83119900	1.32473300	-0.89099700
C	4.60085900	1.13560400	-1.49905400
C	3.54831700	1.97435300	-1.17617800
C	3.71398100	3.00279600	-0.24350600
C	4.96794600	3.17779500	0.34470600
C	6.02818400	2.34543800	0.02003400
C	2.59029900	3.89822100	0.08798400

C	1.16723300	3.52730300	0.15573300
C	0.16284600	4.47942300	-0.03691900
C	-1.16947100	4.10922200	0.05613300
C	-1.52808700	2.79890000	0.33009000
C	-0.53657700	1.83240700	0.51711200
C	0.80276100	2.20979900	0.43026700
C	-0.89657800	0.43979800	0.83834700
C	-2.12646300	-0.26341000	0.43434100
C	-2.76252100	0.05664100	-0.76875700
C	-3.91066200	-0.61025700	-1.16152200
C	-4.42623400	-1.60954300	-0.35347100
C	-3.80783400	-1.95875000	0.83285200
C	-2.66441900	-1.28121200	1.22572000
O	-5.54574500	-2.31767500	-0.76675700
C	-6.78176500	-1.80529900	-0.54406800
C	-7.01018100	-0.63321600	0.17320100
C	-8.30809100	-0.19191600	0.35520700
C	-9.35637400	-0.92358900	-0.17851500
C	-9.13797200	-2.09615600	-0.89243700
C	-7.84556000	-2.53619100	-1.07438500
N	2.88903200	5.13067000	0.36715700
N	-0.04364400	-0.23468100	1.54827900
N	3.14774400	6.20571700	0.61047000
N	0.70243900	-0.81962600	2.16757700
H	5.65226900	-2.96560200	1.40576700
H	5.35071300	-0.63933700	0.63462900
H	8.65840600	-1.10624600	-2.05555700
H	8.95967000	-3.44852100	-1.27124400
H	4.47301000	0.34207100	-2.22525500
H	2.59285200	1.83602700	-1.66486400
H	5.12102200	3.96625900	1.07236600
H	7.00062600	2.47771700	0.47767700
H	0.42006100	5.50689200	-0.26570500
H	-1.94204900	4.85528400	-0.08702800
H	-2.57307800	2.53111500	0.41125400
H	1.57552900	1.46740200	0.58276700
H	-2.35026700	0.82270700	-1.41181900
H	-4.40115300	-0.36535500	-2.09575800
H	-4.22574000	-2.74701200	1.44653500
H	-2.19187100	-1.54699200	2.16409200
H	-6.18661200	-0.06981300	0.58802000
H	-8.50647700	0.71427200	0.90955600
H	-9.97360000	-2.64880900	-1.29744700
H	-7.63806900	-3.44376600	-1.62599400
N	7.48599800	-4.69988700	0.56419300
O	8.38839200	-5.36202500	0.07868200
O	6.71800900	-5.12435800	1.41170000
N	-10.72271400	-0.45260800	0.01459200
O	-10.89445900	0.57885200	0.64318300
O	-11.63188200	-1.11183000	-0.46199600

Bisdiazo-NH_2

C	-6.14569400	4.68390900	-0.48843200
C	-5.71759200	4.15997100	0.73486200
C	-5.87130500	2.81239100	1.01980300
C	-6.45225000	1.97273400	0.08470600
C	-6.88823500	2.47294900	-1.13031500
C	-6.73529400	3.82048600	-1.41629000
O	-6.65157100	0.63332800	0.38923700
C	-5.67257700	-0.26919900	0.08059400
C	-4.46963600	0.07090800	-0.52622000
C	-3.53814600	-0.91992800	-0.80067200
C	-3.77727400	-2.25100300	-0.46517300
C	-4.99258600	-2.57056800	0.14585300
C	-5.93521800	-1.59473900	0.41048600

C	-2.77986500	-3.29994700	-0.76728600
C	-1.32462000	-3.19856800	-0.58428700
C	-0.44907200	-4.07258300	-1.23765300
C	0.91624700	-3.96673200	-1.02922700
C	1.43898700	-2.99861700	-0.18596800
C	0.58022200	-2.11186300	0.46940600
C	-0.79572400	-2.22519300	0.26245400
C	1.11762100	-1.09456900	1.38578200
C	2.50257000	-0.57949000	1.41079100
C	3.19181200	-0.32103900	0.22773000
C	4.48751700	0.17533200	0.24227900
C	5.10767700	0.43673700	1.45788600
C	4.43156000	0.19601800	2.64944200
C	3.14683200	-0.31316500	2.62213200
O	6.37810500	0.92818900	1.57497500
C	7.10234900	1.20222300	0.42321000
C	7.93174400	0.23242800	-0.11369100
C	8.69445400	0.52048600	-1.23460300
C	8.63814800	1.78384300	-1.83018200
C	7.79853000	2.75068300	-1.26946100
C	7.03645800	2.46153500	-0.14854800
N	-3.23965300	-4.42631100	-1.21639900
N	0.30243800	-0.60698500	2.26912700
N	-3.63788800	-5.41413700	-1.60556600
N	-0.41253300	-0.18632100	3.04220800
H	-5.26244400	4.81771000	1.46699100
H	-5.54004500	2.40921800	1.96940300
H	-7.34719200	1.80563600	-1.85008500
H	-7.07651900	4.21200900	-2.36812300
H	-4.25876700	1.09705300	-0.79414300
H	-2.61181300	-0.64501800	-1.28942600
H	-5.20326400	-3.59709900	0.42436700
H	-6.87619800	-1.84349300	0.88534600
H	-0.83121900	-4.83008600	-1.91148000
H	1.58728500	-4.65207800	-1.53357100
H	2.50725600	-2.94011000	-0.02846100
H	-1.46897700	-1.54824300	0.77155000
H	2.71276800	-0.50297300	-0.72609200
H	5.00116600	0.36641400	-0.68993700
H	4.92912600	0.40177500	3.58909100
H	2.63791600	-0.51152100	3.55884800
H	7.97923500	-0.74665400	0.34795200
H	9.34441100	-0.23887700	-1.65512500
H	7.74704800	3.73689600	-1.71719100
H	6.38849500	3.21371800	0.28551100
N	-5.94241900	6.02597800	-0.79628500
N	9.36338700	2.05728600	-2.98624100
H	10.17929300	1.47993100	-3.13258600
H	9.55306200	3.03464400	-3.15760900
H	-6.55768700	6.40710700	-1.50095000
H	-5.84438100	6.64274100	-0.00233300

2.3 Dipole moment analysis

2.3.1 Ground state in gas phase

(1) Bisdiazo-H

X, Y, Z of center of positive charges (nuclear charges) in Angstrom 0.000000 -0.000000 0.000000

X, Y, Z of center of negative charges (electronic charges) in Angstrom 0.001940 0.000623 0.001047

Dipole moment from nuclear charges (a.u.): 0.000000 -0.000000 0.000000

Dipole moment from electrons (a.u.): -0.945837 -0.303833 -0.510564

Dipole moment (a.u.): -0.945837 -0.303833 -0.510564

Dipole moment (Debye): -2.404077 -0.772267 -1.297725
Magnitude of dipole moment: 1.116959 a.u. 2.839027 Debye

(2) Bisdiazo-Me
X, Y, Z of center of positive charges (nuclear charges) in Angstrom -0.000000 -0.000000 -0.000000
X, Y, Z of center of negative charges (electronic charges) in Angstrom 0.002360 0.001379 0.001393
Dipole moment from nuclear charges (a.u.): -0.000000 -0.000000 -0.000000
Dipole moment from electrons (a.u.): -1.222058 -0.713918 -0.721076
Dipole moment (a.u.): -1.222058 -0.713918 -0.721076
Dipole moment (Debye): -3.106162 -1.814598 -1.832791
Magnitude of dipole moment: 1.588413 a.u. 4.037342 Debye

(3) Bisdiazo-NO_2
X, Y, Z of center of positive charges (nuclear charges) in Angstrom 0.000000 -0.000000 0.000000
X, Y, Z of center of negative charges (electronic charges) in Angstrom 0.002230 0.002972 0.001164
Dipole moment from nuclear charges (a.u.): 0.000002 -0.000000 0.000000
Dipole moment from electrons (a.u.): -1.272742 -1.695927 -0.664503
Dipole moment (a.u.): -1.272741 -1.695927 -0.664503
Dipole moment (Debye): -3.234984 -4.310616 -1.688997
Magnitude of dipole moment: 2.222072 a.u. 5.647942 Debye

(4) Bisdiazo-NH_2
X, Y, Z of center of positive charges (nuclear charges) in Angstrom 0.000000 0.000000 -0.000000
X, Y, Z of center of negative charges (electronic charges) in Angstrom 0.002742 0.003693 0.001651
Dipole moment from nuclear charges (a.u.): 0.000001 0.000000 -0.000000
Dipole moment from electrons (a.u.): -1.419692 -1.912383 -0.854707
Dipole moment (a.u.): -1.419691 -1.912383 -0.854707
Dipole moment (Debye): -3.608495 -4.860792 -2.172448
Magnitude of dipole moment: 2.530465 a.u. 6.431801 Debye

2.3.2 SMD model in THF

(1) Bisdiazo-H
X, Y, Z of center of positive charges (nuclear charges) in Angstrom -0.000000 0.000000 0.000000
X, Y, Z of center of negative charges (electronic charges) in Angstrom 0.002529 0.000359 0.001278
Dipole moment from nuclear charges (a.u.): -0.000001 0.000000 0.000000
Dipole moment from electrons (a.u.): -1.233089 -0.175265 -0.622860
Dipole moment (a.u.): -1.233089 -0.175265 -0.622860
Dipole moment (Debye): -3.134200 -0.445480 -1.583151
Magnitude of dipole moment: 1.392545 a.u. 3.539496 Debye

(2) Bisdiazo-Me
X, Y, Z of center of positive charges (nuclear charges) in Angstrom -0.000000 -0.000000 0.000000
X, Y, Z of center of negative charges (electronic charges) in Angstrom -0.003168 -0.001928 0.002127
Dipole moment from nuclear charges (a.u.): -0.000000 -0.000000 0.000000
Dipole moment from electrons (a.u.): 1.640183 0.998043 -1.101439
Dipole moment (a.u.): 1.640183 0.998043 -1.101438
Dipole moment (Debye): 4.168929 2.536771 -2.799577
Magnitude of dipole moment: 2.213472 a.u. 5.626083 Debye

(3) Bisdiazo-NO_2
X, Y, Z of center of positive charges (nuclear charges) in Angstrom -0.000000 -0.000000 0.000000
X, Y, Z of center of negative charges (electronic charges) in Angstrom -0.002076 -0.003459 0.001861
Dipole moment from nuclear charges (a.u.): -0.000001 -0.000000 0.000000
Dipole moment from electrons (a.u.): 1.184485 1.974078 -1.062327
Dipole moment (a.u.): 1.184484 1.974078 -1.062327
Dipole moment (Debye): 3.010657 5.017605 -2.700166
Magnitude of dipole moment: 2.535454 a.u. 6.444479 Debye

(4) Bisdiazo-NH_2
X, Y, Z of center of positive charges (nuclear charges) in Angstrom 0.000000 -0.000000 0.000000
X, Y, Z of center of negative charges (electronic charges) in Angstrom -0.003109 -0.004348 0.002438
Dipole moment from nuclear charges (a.u.): 0.000001 -0.000000 0.000000
Dipole moment from electrons (a.u.): 1.609577 2.251537 -1.262132
Dipole moment (a.u.): 1.609578 2.251536 -1.262132
Dipole moment (Debye): 4.091138 5.722834 -3.208019
Magnitude of dipole moment: 3.041896 a.u. 7.731728 Debye

2.3.3 SMD model in Toluene

(1) Bisdiazo-H
X, Y, Z of center of positive charges (nuclear charges) in Angstrom -0.000000 -0.000000 -0.000000
X, Y, Z of center of negative charges (electronic charges) in Angstrom 0.002261 0.000472 0.001165
Dipole moment from nuclear charges (a.u.): -0.000001 -0.000000 -0.000000
Dipole moment from electrons (a.u.): -1.102140 -0.230107 -0.568177
Dipole moment (a.u.): -1.102141 -0.230108 -0.568177
Dipole moment (Debye): -2.801362 -0.584875 -1.444162
Magnitude of dipole moment: 1.261146 a.u. 3.205513 Debye

(2) Bisdiazo-Me
X, Y, Z of center of positive charges (nuclear charges) in Angstrom 0.000000 0.000000 0.000000
X, Y, Z of center of negative charges (electronic charges) in Angstrom -0.002859 -0.002042 0.001927
Dipole moment from nuclear charges (a.u.): 0.000000 0.000000 0.000000
Dipole moment from electrons (a.u.): 1.480169 1.057181 -0.998011
Dipole moment (a.u.): 1.480169 1.057182 -0.998011
Dipole moment (Debye): 3.762215 2.687088 -2.536691
Magnitude of dipole moment: 2.074743 a.u. 5.273471 Debye

(3) Bisdiazo-NO_2
X, Y, Z of center of positive charges (nuclear charges) in Angstrom 0.000000 0.000000 -0.000000
X, Y, Z of center of negative charges (electronic charges) in Angstrom -0.001989 -0.003301 0.001729
Dipole moment from nuclear charges (a.u.): 0.000000 0.000001 -0.000000
Dipole moment from electrons (a.u.): 1.134847 1.884077 -0.986956
Dipole moment (a.u.): 1.134847 1.884077 -0.986956
Dipole moment (Debye): 2.884493 4.788846 -2.508592
Magnitude of dipole moment: 2.410748 a.u. 6.127510 Debye

(4) Bisdiazo-NH_2

X, Y, Z of center of positive charges (nuclear charges) in Angstrom -0.000000 -0.000000 -0.000000
X, Y, Z of center of negative charges (electronic charges) in Angstrom -0.002962 -0.003977 0.002071
Dipole moment from nuclear charges (a.u.): -0.000000 -0.000000 -0.000000
Dipole moment from electrons (a.u.): 1.533873 2.059083 -1.072459
Dipole moment (a.u.): 1.533873 2.059082 -1.072460
Dipole moment (Debye): 3.898716 5.233665 -2.725920
Magnitude of dipole moment: 2.782581 a.u. 7.072614 Debye

2.3.4 SMD model in Chlorobenzene

(1) Bisdiazo-H
X, Y, Z of center of positive charges (nuclear charges) in Angstrom 0.000000 -0.000000 0.000000
X, Y, Z of center of negative charges (electronic charges) in Angstrom 0.002481 0.000374 0.001245
Dipole moment from nuclear charges (a.u.): 0.000000 -0.000000 0.000000
Dipole moment from electrons (a.u.): -1.209515 -0.182256 -0.607052
Dipole moment (a.u.): -1.209515 -0.182256 -0.607052
Dipole moment (Debye): -3.074280 -0.463249 -1.542971
Magnitude of dipole moment: 1.365524 a.u. 3.470815 Debye

(2) Bisdiazo-Me
X, Y, Z of center of positive charges (nuclear charges) in Angstrom 0.000000 -0.000000 -0.000000
X, Y, Z of center of negative charges (electronic charges) in Angstrom -0.003114 -0.002062 0.002119
Dipole moment from nuclear charges (a.u.): 0.000001 -0.000000 -0.000000
Dipole moment from electrons (a.u.): 1.612170 1.067906 -1.097211
Dipole moment (a.u.): 1.612171 1.067906 -1.097211
Dipole moment (Debye): 4.097731 2.714345 -2.788833
Magnitude of dipole moment: 2.223374 a.u. 5.651252 Debye

(3) Bisdiazo-NO_2
X, Y, Z of center of positive charges (nuclear charges) in Angstrom 0.000000 0.000000 -0.000000
X, Y, Z of center of negative charges (electronic charges) in Angstrom -0.002046 -0.003445 0.001846
Dipole moment from nuclear charges (a.u.): 0.000001 0.000000 -0.000000
Dipole moment from electrons (a.u.): 1.167736 1.966189 -1.053313
Dipole moment (a.u.): 1.167737 1.966189 -1.053313
Dipole moment (Debye): 2.968091 4.997552 -2.677255
Magnitude of dipole moment: 2.517732 a.u. 6.399436 Debye

(4) Bisdiazo-NH_2
X, Y, Z of center of positive charges (nuclear charges) in Angstrom 0.000000 -0.000000 0.000000
X, Y, Z of center of negative charges (electronic charges) in Angstrom-0.003055 -0.004300 0.002400
Dipole moment from nuclear charges (a.u.): 0.000000 -0.000000 0.000000
Dipole moment from electrons (a.u.): 1.581578 2.226248 -1.242486
Dipole moment (a.u.): 1.581578 2.226247 -1.242486
Dipole moment (Debye): 4.019971 5.658556 -3.158084
Magnitude of dipole moment: 3.000223 a.u. 7.625806 Debye

2.4 Geometry coordinates of Transit state (TS) in gas phase

2.4.1 TS1 (one C-N≡N)

(1) Bisdiazo-H

C	5.58944000	-4.91448800	-1.25244300
C	4.93325700	-4.44093900	-0.11634100
C	5.19081500	-3.15875200	0.36076000
C	6.10462700	-2.35737000	-0.31647600
C	6.76888100	-2.81355200	-1.44728900
C	6.50778000	-4.10132300	-1.91307200
O	6.42190800	-1.09476200	0.17253100
C	5.48867300	-0.09921100	0.11820400
C	4.32040600	-0.18225700	-0.64853200
C	3.43818900	0.88635900	-0.64856200
C	3.69600400	2.05620600	0.09230100
C	4.89613000	2.11784700	0.82711300
C	5.77938700	1.05362400	0.85494800
C	2.79194100	3.19779300	0.14600200
C	1.36636000	3.00407500	-0.00247900
C	0.49962800	3.91154100	-0.65191800
C	-0.87273900	3.74490200	-0.58083900
C	-1.44248800	2.70349000	0.15259700
C	-0.61081900	1.78505500	0.80464600
C	0.77923000	1.92924000	0.69788000
C	-1.18785700	0.67872300	1.58780900
C	-2.49210900	0.03515200	1.35120300
C	-3.00299700	-0.08082900	0.04907900
C	-4.22387800	-0.69763900	-0.18997900
C	-4.95632700	-1.21701600	0.87679300
C	-4.46243200	-1.12674800	2.17422700
C	-3.24319500	-0.50227600	2.40654600
O	-6.14199800	-1.89896800	0.69523900
C	-7.06489800	-1.43268300	-0.22052300
C	-7.33281100	-0.07360900	-0.37900700
C	-8.32044400	0.32390600	-1.27638200
C	-9.04148900	-0.62191300	-2.00365100
C	-8.76920000	-1.97742600	-1.82704300
C	-7.77913500	-2.38794900	-0.93826000
N	3.33038700	4.28731900	-1.27041300
N	-0.47561400	0.21866000	2.58154600
N	3.95340200	5.16666600	-1.53886000
N	0.14548200	-0.18241900	3.44935400
H	5.38801000	-5.91420900	-1.61774300
H	4.22374400	-5.07261900	0.40484300
H	4.69416300	-2.77726200	1.24443400
H	7.47800000	-2.16312400	-1.94378200
H	7.02302500	-4.46635100	-2.79359500
H	4.11498000	-1.06902300	-1.23277000
H	2.53189100	0.82785000	-1.23941000
H	5.10617200	3.01924300	1.39032500
H	6.69432600	1.08659600	1.43271000
H	0.91104100	4.73402600	-1.22237700
H	-1.52015900	4.44819600	-1.09267200
H	-2.51780400	2.61220500	0.22427100
H	1.42443300	1.21475100	1.19209600
H	-2.43068100	0.30247100	-0.78567100
H	-4.60323000	-0.78556900	-1.20008800
H	-5.04494100	-1.53799400	2.98904200
H	-2.87859800	-0.42104700	3.42411400
H	-6.77608700	0.65774700	0.19274500
H	-8.53128000	1.37986800	-1.40000000
H	-9.80943700	-0.30532400	-2.69885300
H	-9.32531300	-2.72099800	-2.38596100
H	-7.55054200	-3.43564200	-0.78806500

(2) Bisdiazo-Me

C	5.81642800	-4.58231200	-0.96215100

C	5.23912400	-4.07455600	0.20746900
C	5.46724200	-2.76453400	0.61564600
C	6.27836600	-1.94474900	-0.16065000
C	6.86448700	-2.41774900	-1.32537300
C	6.63006800	-3.73422600	-1.71797400
O	6.55965800	-0.64608100	0.25555700
C	5.59747100	0.31444500	0.13845200
C	4.40402300	0.12668700	-0.56911000
C	3.48962400	1.16589200	-0.63459800
C	3.73729200	2.40753200	-0.01751200
C	4.96139900	2.57142200	0.66062900
C	5.87823700	1.54021500	0.75215900
C	2.80073500	3.52299100	-0.03535700
C	1.37776200	3.27816000	-0.11954200
C	0.46549600	4.09654900	-0.82274700
C	-0.89859500	3.90021700	-0.69188400
C	-1.41515300	2.91712300	0.15280800
C	-0.53750100	2.08677700	0.86075900
C	0.84396100	2.25852200	0.69652100
C	-1.05817700	1.04364400	1.76066400
C	-2.35768100	0.35883300	1.63620300
C	-2.91093600	0.10301400	0.37319500
C	-4.12922400	-0.55185800	0.24035600
C	-4.81518900	-0.97262900	1.37936300
C	-4.27654900	-0.74281700	2.64307000
C	-3.06313700	-0.07958100	2.76674000
O	-5.99801300	-1.67672000	1.32521700
C	-6.86916000	-1.46103200	0.27103900
C	-7.28520000	-0.17908000	-0.08194300
C	-8.21006300	-0.02133200	-1.10816000
C	-8.74206800	-1.12320800	-1.78787300
C	-8.31159100	-2.39654500	-1.40823400
C	-7.37891800	-2.57218800	-0.38794900
N	3.26327900	4.50012000	-1.55644200
N	-0.29732800	0.68509500	2.75991700
N	3.85338800	5.36938700	-1.91702100
N	0.36555800	0.37326100	3.63377300
H	4.60409300	-4.71415500	0.81093700
H	5.02051100	-2.37448100	1.52210500
H	7.49264500	-1.75799400	-1.91098100
H	7.08703000	-4.10461000	-2.62916300
H	4.20439200	-0.81750300	-1.05734400
H	2.56405500	1.02607700	-1.18018500
H	5.16349700	3.52751100	1.12857400
H	6.81238800	1.65320900	1.28756800
H	0.83493800	4.87223700	-1.48074600
H	-1.58165700	4.53444800	-1.24597900
H	-2.48463100	2.80340100	0.26662600
H	1.52489900	1.61214600	1.23461400
H	-2.37555100	0.40848900	-0.51648700
H	-4.53917100	-0.74509000	-0.74237600
H	-4.82313200	-1.07742400	3.51583700
H	-2.66566000	0.11062000	3.75717800
H	-6.88355400	0.68080200	0.43969500
H	-8.52975000	0.97894400	-1.38107000
H	-8.70514000	-3.26799200	-1.92045300
H	-7.04149800	-3.55898200	-0.09621000
C	5.58904100	-6.01440500	-1.37585500
H	6.28366600	-6.68517400	-0.85939000
H	4.57597000	-6.34242400	-1.13228500
H	5.74040500	-6.14671300	-2.44898500
C	-9.76989900	-0.93798100	-2.87592400
H	-9.81076000	-1.80742500	-3.53521200
H	-10.77005400	-0.79807700	-2.45215700
H	-9.54886900	-0.05962300	-3.48716000

(3) Bisdiazo-NO_2

C	-6.02346500	3.45434700	0.55822200
C	-4.94346800	2.66328500	0.18155200
C	-5.16683300	1.35179000	-0.21177300
C	-6.47088100	0.84818600	-0.21564300
C	-7.55060900	1.65334800	0.15719900
C	-7.32786000	2.96415800	0.54468200
O	-6.78002700	-0.42459700	-0.62005100
C	-5.78722300	-1.38795500	-0.63207300
C	-5.54436000	-2.06289700	-1.82159400
C	-4.56543000	-3.05219100	-1.85111800
C	-3.78667700	-3.32762200	-0.71814100
C	-4.07998200	-2.64723800	0.48049300
C	-5.09096800	-1.70315600	0.53618300
C	-2.64227200	-4.23040700	-0.87639000
C	-1.36297100	-3.76582900	-0.36700600
C	-0.31312000	-4.63447200	0.00503400
C	0.92642400	-4.12422200	0.34930400
C	1.18593800	-2.75209300	0.29825600
C	0.17711000	-1.86922000	-0.10357600
C	-1.08837300	-2.38619800	-0.40998600
C	0.40988500	-0.41425000	-0.15128300
C	1.70027100	0.25921100	-0.37173900
C	2.68710700	-0.34202600	-1.16969700
C	3.90078800	0.29318800	-1.39738400
C	4.13682900	1.54286400	-0.83463400
C	3.17267200	2.16660900	-0.05476700
C	1.96296600	1.52249400	0.17886900
O	5.32519400	2.21338900	-1.10215900
C	6.46593800	1.83105900	-0.45692200
C	6.48428400	0.91441300	0.59867300
C	7.69197000	0.59869000	1.20618100
C	8.86159100	1.19944000	0.75490300
C	8.85127500	2.11827500	-0.29376800
C	7.64866200	2.43375600	-0.90035700
N	-3.09314700	-5.71863800	0.15797100
N	-0.63766100	0.35018800	0.00782300
N	-3.40985700	-6.78074000	0.15834200
N	-1.56416300	0.99991700	0.14483300
H	-3.94352900	3.07272700	0.19644400
H	-4.33234500	0.73400500	-0.51090300
H	-8.54854800	1.23525000	0.13837300
H	-8.14286600	3.60874000	0.84171000
H	-6.11899200	-1.80721600	-2.70266800
H	-4.35998400	-3.59406700	-2.76592800
H	-3.49786000	-2.86678400	1.36764700
H	-5.32236800	-1.18334600	1.45760100
H	-0.48672000	-5.70278900	0.03589500
H	1.71420700	-4.79940400	0.66308800
H	2.15948300	-2.37560000	0.58236300
H	-1.87136200	-1.71040700	-0.72445600
H	2.49432400	-1.30260600	-1.62880800
H	4.65912900	-0.16675900	-2.01909200
H	3.37805000	3.14150400	0.36916400
H	1.22036400	2.00582600	0.80286600
H	5.56669000	0.45793200	0.94217800
H	7.73743600	-0.10431200	2.02593100
H	9.77935900	2.56736200	-0.61780000
H	7.60032300	3.14123600	-1.71781200
N	-5.78382000	4.84167300	0.97737700
O	-6.75693500	5.51982800	1.28883600
O	-4.62483500	5.24188900	0.99258700
N	10.13601000	0.85942400	1.39855400
O	10.11448600	0.04355100	2.31432900

O	11.15008600	1.41023300	0.98346100

(4) Bisdiazo-NH_2

C	5.53469300	-4.79534700	-0.75564400
C	5.05286600	-4.25843400	0.44758000
C	5.33453900	-2.94479100	0.80188200
C	6.09581000	-2.15045100	-0.04647900
C	6.58387700	-2.66128600	-1.24109600
C	6.30495600	-3.97707800	-1.59408800
O	6.42474800	-0.84719600	0.32742500
C	5.51701800	0.14887700	0.11916300
C	4.30482700	-0.03942200	-0.55605600
C	3.44601200	1.03719300	-0.71136500
C	3.76731800	2.31627800	-0.21695900
C	5.00750500	2.47619000	0.43308500
C	5.86940300	1.41044500	0.61330600
C	2.89251800	3.47435600	-0.33414000
C	1.45751800	3.30184200	-0.39073100
C	0.58619100	4.10008500	-1.16547900
C	-0.78551600	3.99245000	-1.01283900
C	-1.34862000	3.12294200	-0.07824400
C	-0.51245600	2.31564900	0.70318900
C	0.87530000	2.39462800	0.51936200
C	-1.08413500	1.39360400	1.69800600
C	-2.43488500	0.80149500	1.65599700
C	-3.02103500	0.44455400	0.43562800
C	-4.29075000	-0.12226900	0.38086200
C	-4.99211300	-0.35755300	1.56295200
C	-4.42039500	-0.02161500	2.79086900
C	-3.16108600	0.55678500	2.83229900
O	-6.24690900	-0.91829100	1.61108000
C	-6.88081800	-1.22845500	0.41290000
C	-7.65809300	-0.27165300	-0.22865300
C	-8.33307100	-0.59829300	-1.39911300
C	-8.24205200	-1.88799600	-1.94199800
C	-7.45457100	-2.83986800	-1.27932300
C	-6.77869200	-2.51167100	-0.10923800
N	3.39557500	4.28405600	-1.93800900
N	-0.32908500	1.06446800	2.71108200
N	4.03179300	5.08363300	-2.37430500
N	0.32802900	0.77984500	3.59905300
H	4.45463100	-4.87579500	1.10884700
H	4.96560100	-2.53251100	1.73314400
H	7.18053500	-2.02852600	-1.88657000
H	6.68507300	-4.37390100	-2.52915400
H	4.04822700	-1.01371500	-0.94909200
H	2.50614500	0.89658600	-1.23178600
H	5.26629400	3.45972300	0.80688500
H	6.81535400	1.52237800	1.12793800
H	0.99256300	4.78852600	-1.89489500
H	-1.43738900	4.60815700	-1.62267800
H	-2.42177900	3.07890300	0.04800500
H	1.52449500	1.76531500	1.11400500
H	-2.47668300	0.60625200	-0.48603200
H	-4.72451700	-0.38897200	-0.57330500
H	-4.98132700	-0.20783200	3.69823700
H	-2.73807100	0.83025600	3.79234400
H	-7.73129300	0.72349800	0.19286000
H	-8.93714800	0.15185500	-1.89815900
H	-7.37103200	-3.84249300	-1.68481800
H	-6.17199400	-3.24707700	0.40490400
N	5.20554600	-6.09728500	-1.13620000
N	-8.87520000	-2.20033400	-3.14824200
H	-9.66997900	-1.62434900	-3.38307400
H	-9.06092100	-3.18068900	-3.29979100

H	5.82808200	-6.52921200	-1.80258200
H	4.95696600	-6.72320600	-0.38477200

2.4.2 TS2 (two C-N≡N)

Bisdiazo-H

C	8.19830000	-4.25177900	-1.42616700
C	6.84344600	-4.43061100	-1.14710100
C	6.15463200	-3.49500700	-0.38004100
C	6.83554800	-2.37934800	0.09658900
C	8.18480900	-2.18683300	-0.16979000
C	8.86638600	-3.13231600	-0.93457100
O	6.18189800	-1.46288200	0.91213500
C	5.25585500	-0.61942600	0.36433300
C	5.09129800	-0.44589900	-1.01240200
C	4.14581500	0.46381500	-1.46625600
C	3.33014700	1.18548000	-0.57988100
C	3.52571500	0.98962600	0.80451400
C	4.48064700	0.10865600	1.27523500
C	2.33067400	2.08841800	-1.14106300
C	1.08282500	2.30646400	-0.43955300
C	0.36989900	3.53360900	-0.46547800
C	-0.90780700	3.60895400	0.05185800
C	-1.55728000	2.47493900	0.54934400
C	-0.87955900	1.23939500	0.60913900
C	0.45482900	1.19666100	0.14307100
C	-1.47243200	0.14707400	1.34694900
C	-2.90700200	-0.07165500	1.26198300
C	-3.67726800	0.19008100	0.11100200
C	-5.04101800	-0.05876800	0.07657000
C	-5.67027000	-0.55476900	1.22369400
C	-4.93442800	-0.82627700	2.38232100
C	-3.56900200	-0.60737800	2.38571500
O	-7.00660500	-0.83636700	1.28647800
C	-7.83454500	-0.44599900	0.23996400
C	-8.21490300	0.88626100	0.11762100
C	-9.08947600	1.24891900	-0.90335600
C	-9.57911700	0.28583900	-1.78527800
C	-9.19299000	-1.04550200	-1.64399900
C	-8.31458000	-1.41820700	-0.62785300
N	3.19438100	3.74178300	-1.20123900
N	-0.76803580	-1.40946012	0.59578155
N	3.76184300	4.43645000	-1.85626800
N	-0.33386080	-2.38644712	0.88803355
H	8.73076800	-4.98420800	-2.02078500
H	6.32183800	-5.30350900	-1.52143000
H	5.10385100	-3.61975500	-0.14904100
H	8.68232100	-1.30895700	0.22298900
H	9.91982300	-2.99127500	-1.14548400
H	5.69969600	-1.00497300	-1.70997600
H	4.01292800	0.62412500	-2.52965600
H	2.91582700	1.54580400	1.50619100
H	4.64509000	-0.04106400	2.33508100
H	0.83616200	4.41685400	-0.88260900
H	-1.43081600	4.55907000	0.04609600
H	-2.56339500	2.55681600	0.93742000
H	0.98726600	0.25733600	0.18770600
H	-3.18408200	0.57650000	-0.77288200
H	-5.61371400	0.12526500	-0.82239800
H	-5.45191500	-1.21782800	3.24898900
H	-2.97907300	-0.83186100	3.26628300
H	-7.82735200	1.61887900	0.81482700
H	-9.39264500	2.28415400	-1.00591200
H	-10.26150900	0.57208900	-2.57643500

H	-9.57421000	-1.79758000	-2.32452800
H	-8.00012900	-2.44622200	-0.49824200

2.5 Geometry of Diazo-carbene and biscarabene in gas phase

2.5.1 Coordinates of Diazo-carbene

Here it should be noted that the wavefunction stability check of the singlet stated DiazoCarbeneH stated that the wavefunction has an RHF -> UHF instability at theory level of B3LYP-D3BJ/6-311+G**, suggesting the triplet state wavefunction lower in energy than the current singlet state. While for the triplet state that wavefunction is stable under the perturbations considered (UB3LYP-D3BJ). Here shows the coordinates of triplet state carbene species.

(1) Diazocarbene-H

C	-5.85540500	4.79830800	-0.35546000
C	-5.65841800	4.42746200	0.97306900
C	-5.77817400	3.09196900	1.35471000
C	-6.09808100	2.14401100	0.39253000
C	-6.30586400	2.49509000	-0.93696900
C	-6.17918200	3.83139800	-1.30714600
O	-6.27405600	0.82124300	0.79302800
C	-5.33420000	-0.10702300	0.46819900
C	-4.08328500	0.21911900	-0.07222800
C	-3.18417600	-0.79524900	-0.34575300
C	-3.51456200	-2.16266600	-0.14342600
C	-4.81096900	-2.44730500	0.36595300
C	-5.69027100	-1.44347600	0.70581100
C	-2.66291600	-3.25327000	-0.47613400
C	-1.24961800	-3.02540500	-0.53269000
C	-0.49123400	-3.63097700	-1.56295800
C	0.87826000	-3.43044400	-1.63149100
C	1.54696800	-2.71698900	-0.63611700
C	0.83180500	-2.14484600	0.42833100
C	-0.56060300	-2.27287900	0.44731400
C	1.54177500	-1.41976900	1.49725700
C	2.80995200	-0.68465400	1.35139600
C	3.13058400	-0.05217100	0.14011600
C	4.31676900	0.65446100	-0.00863700
C	5.20537100	0.74587500	1.06195900
C	4.90127100	0.13955800	2.27683900
C	3.71619000	-0.57188400	2.41593100
O	6.36592000	1.48874700	0.99799900
C	7.12942700	1.46660400	-0.15304200
C	7.35148500	0.29385300	-0.87360100
C	8.18322400	0.32958300	-1.98964500
C	8.79570700	1.51929600	-2.38081200
C	8.57201100	2.68143100	-1.64460000
C	7.73659000	2.66080600	-0.53078700
N	0.99197800	-1.42355300	2.68225700
N	0.51437000	-1.42863500	3.71714200
H	-5.76103600	5.83696000	-0.64836400
H	-5.41063000	5.17605900	1.71611800
H	-5.62987900	2.77918700	2.38066600
H	-6.55895700	1.72913100	-1.65912800
H	-6.33962400	4.11747600	-2.33987000
H	-3.82950200	1.25227400	-0.26712100
H	-2.21643500	-0.54707700	-0.76285100
H	-5.08174600	-3.48863000	0.49006100
H	-6.67070400	-1.65353500	1.11427100
H	-1.00761600	-4.23889700	-2.29518000
H	1.44905800	-3.87484800	-2.43900700
H	2.62529100	-2.63051900	-0.66647300
H	-1.13178300	-1.81803600	1.24762200
H	2.43649600	-0.10055800	-0.68901500

H	4.54812400	1.14218200	-0.94697800
H	5.60291900	0.22382100	3.09717000
H	3.50056000	-1.05582500	3.36158000
H	6.88017200	-0.62958400	-0.56227800
H	8.35864400	-0.58123300	-2.55037000
H	9.44289800	1.53922300	-3.24915600
H	9.04502300	3.61102200	-1.93896400
H	7.54794000	3.55268200	0.05353200

(2) Diazocarbene-Me

C	5.61313500	-4.57604300	-1.05953000
C	5.25188500	-4.06504500	0.19224800
C	5.53990900	-2.75083900	0.54629700
C	6.19220900	-1.93212000	-0.36754500
C	6.56374500	-2.40759900	-1.61629000
C	6.27136400	-3.72748800	-1.95388600
O	6.53367700	-0.62785300	-0.01362300
C	5.56882400	0.33128200	0.00937800
C	4.27100300	0.13098700	-0.48076900
C	3.35576900	1.16461300	-0.40259300
C	3.71002500	2.44141100	0.11338900
C	5.05592600	2.61669100	0.53744600
C	5.95930300	1.57704200	0.52464700
C	2.82914900	3.55662600	0.17535400
C	1.41806900	3.30936100	0.21625300
C	0.54565700	4.14291200	-0.52376300
C	-0.82255500	3.92579100	-0.49006800
C	-1.37222600	2.95267100	0.34548100
C	-0.53913100	2.14046700	1.13169300
C	0.84640700	2.30289400	1.02948800
C	-1.12082700	1.13456100	2.03755500
C	-2.41180100	0.44698600	1.85484000
C	-2.88188000	0.14297500	0.56928000
C	-4.09253200	-0.51313600	0.38124600
C	-4.85421500	-0.88736900	1.48789100
C	-4.39848400	-0.60918000	2.77478400
C	-3.19302400	0.05554100	2.95255000
O	-6.03574100	-1.58726100	1.38633100
C	-6.81017800	-1.44973200	0.24631300
C	-7.22421000	-0.19824200	-0.20419600
C	-8.05206600	-0.11485100	-1.31814200
C	-8.48855100	-1.26225300	-1.99083300
C	-8.06283800	-2.50356400	-1.51273100
C	-7.22602300	-2.60453700	-0.40289800
N	-0.42579600	0.81616700	3.09635000
N	0.17932200	0.54023200	4.02253100
H	4.74020400	-4.70486600	0.90302500
H	5.26071700	-2.35740400	1.51610900
H	7.07170000	-1.74757900	-2.30846200
H	6.55938900	-4.10053600	-2.93064400
H	3.99448200	-0.82389400	-0.90709500
H	2.35213600	1.01293400	-0.77925700
H	5.34313200	3.59759400	0.89562500
H	6.97621600	1.69655900	0.87670000
H	0.97386800	4.93923200	-1.11951100
H	-1.48287400	4.55100500	-1.08046700
H	-2.44679400	2.84368700	0.41247200
H	1.50640400	1.67082600	1.61115500
H	-2.28758600	0.41083400	-0.29495600
H	-4.43701800	-0.74342000	-0.61836800
H	-5.00313600	-0.90799700	3.62186100
H	-2.86096500	0.28380900	3.95891700
H	-6.89554900	0.69560400	0.31155500
H	-8.37085700	0.86176000	-1.66712400
H	-8.38387600	-3.40868700	-2.01702200

H	-6.89203200	-3.56642200	-0.03418200
C	5.32624000	-6.01203100	-1.41902000
H	6.11988300	-6.67192400	-1.05319400
H	4.38763000	-6.35267900	-0.97633200
H	5.26030000	-6.14527500	-2.50066600
C	-9.41380800	-1.15759000	-3.17720100
H	-9.37084700	-2.05727800	-3.79438100
H	-10.45244900	-1.02772100	-2.85507900
H	-9.15828400	-0.30212900	-3.80715300

(3) Diazocarbene-NO_2

C	-7.25884600	3.25871700	0.79573500
C	-6.12817100	2.87863600	0.07911900
C	-6.03481200	1.57904400	-0.39668500
C	-7.07223500	0.67975800	-0.13882500
C	-8.20543200	1.07163400	0.57339400
C	-8.30228800	2.37231300	1.04483100
O	-7.07111300	-0.60201900	-0.63357900
C	-5.91048200	-1.33413900	-0.69176000
C	-5.80373200	-2.24688500	-1.74484300
C	-4.70000700	-3.07552900	-1.80996300
C	-3.63124800	-2.95741200	-0.88444300
C	-3.78702800	-2.02576100	0.17898200
C	-4.92346900	-1.24706200	0.29553400
C	-2.49294400	-3.80077900	-1.04806400
C	-1.23642700	-3.31594300	-0.55880000
C	-0.31280400	-4.22860600	0.00433800
C	0.90395800	-3.77941300	0.49359200
C	1.27526200	-2.44132700	0.35802000
C	0.41342200	-1.52225300	-0.26242800
C	-0.84188700	-1.96296300	-0.68680700
C	0.80337900	-0.10641400	-0.40551100
C	2.16625100	0.40325000	-0.61654300
C	3.12785800	-0.39315200	-1.26025600
C	4.41452800	0.08197100	-1.47809300
C	4.75183300	1.36464100	-1.06097000
C	3.81698300	2.17860800	-0.43577900
C	2.53306600	1.69592900	-0.21084700
O	6.02037500	1.87050200	-1.32482700
C	7.06541800	1.49302700	-0.53194200
C	6.91465800	0.73879400	0.63566300
C	8.03608300	0.41414100	1.38691500
C	9.28892400	0.84513200	0.96604500
C	9.44746900	1.60110700	-0.19480500
C	8.33112900	1.92498900	-0.94477400
N	-0.16230100	0.77234500	-0.34783700
N	-1.01200200	1.52926700	-0.29474300
H	-5.34419800	3.59973300	-0.10392700
H	-5.16902300	1.26631300	-0.96462100
H	-8.99338300	0.35074000	0.74746800
H	-9.16510100	2.70645700	1.60323300
H	-6.60371200	-2.30220100	-2.47216600
H	-4.60850000	-3.81679000	-2.59386900
H	-3.01621300	-1.95839500	0.93596300
H	-5.04853100	-0.56899500	1.12998100
H	-0.59008100	-5.27368200	0.05870100
H	1.58816400	-4.47469700	0.96599000
H	2.23626300	-2.10920100	0.72945400
H	-1.51942300	-1.26937200	-1.16916700
H	2.86020700	-1.38209400	-1.60838000
H	5.15335300	-0.52967300	-1.98126300
H	4.10174500	3.17541100	-0.12316900
H	1.81297500	2.33043400	0.29238600
H	5.93406900	0.41334700	0.95323900
H	7.95079200	-0.16589000	2.29497000

H	10.43639700	1.92018400	-0.49184200
H	8.41397900	2.50894400	-1.85210300
N	-7.35504500	4.63797600	1.30106200
O	-8.36824100	4.94894700	1.91596900
O	-6.41604800	5.39264400	1.07713100
N	10.47128400	0.49689500	1.76265400
O	10.30129400	-0.17369000	2.77566100
O	11.56227600	0.89621400	1.36971300

(4) Diazocarbene -NH_2

C	5.29933400	-4.79341800	-0.80810900
C	5.01055400	-4.22932100	0.44364600
C	5.36247700	-2.91580300	0.72862100
C	6.00088200	-2.14952800	-0.23816900
C	6.29831700	-2.68671800	-1.48255800
C	5.94902100	-4.00230900	-1.76639900
O	6.40434200	-0.84601300	0.05868200
C	5.49331900	0.16109700	-0.00992300
C	4.18398300	-0.01201800	-0.48004600
C	3.32512100	1.07171200	-0.49318100
C	3.74835500	2.36860700	-0.09192200
C	5.10360600	2.50849600	0.31590100
C	5.95049500	1.42546100	0.39486100
C	2.92835600	3.53018000	-0.12889400
C	1.50593200	3.36280700	-0.07077000
C	0.67806500	4.17295700	-0.88432700
C	-0.69966200	4.03229800	-0.83587900
C	-1.29924100	3.16719400	0.08023600
C	-0.50945700	2.38490200	0.93846900
C	0.88290500	2.46417700	0.82647600
C	-1.14336000	1.49599100	1.92685600
C	-2.48274000	0.88863500	1.81222600
C	-2.98064100	0.48700300	0.56673400
C	-4.23912500	-0.09396900	0.44320700
C	-5.01812000	-0.29931100	1.58145600
C	-4.53502100	0.08173100	2.83416800
C	-3.28661800	0.67455700	2.94320400
O	-6.26909700	-0.86950300	1.56150200
C	-6.80594700	-1.24706600	0.33538000
C	-7.56880600	-0.34335700	-0.39405900
C	-8.14821200	-0.73635600	-1.59507600
C	-7.97396700	-2.03999200	-2.08120100
C	-7.20225900	-2.93772500	-1.33003000
C	-6.62275500	-2.54345700	-0.12921500
N	-0.45662000	1.21385600	3.00071000
N	0.14061500	0.97091700	3.94166300
H	4.50795700	-4.82539600	1.19749800
H	5.14313400	-2.48267600	1.69689700
H	6.80209000	-2.07481800	-2.22061500
H	6.17909500	-4.42015200	-2.74039600
H	3.85553000	-0.98514000	-0.81918800
H	2.31272200	0.94065800	-0.85387400
H	5.44368700	3.50058900	0.58637600
H	6.97382900	1.52124400	0.73555100
H	1.14712600	4.88961000	-1.54676400
H	-1.32688200	4.63659100	-1.48163700
H	-2.37793400	3.12091000	0.15236800
H	1.50963900	1.85213600	1.46369600
H	-2.37554800	0.62368100	-0.32060400
H	-4.60406400	-0.39490900	-0.52938300
H	-5.15547000	-0.08161000	3.70651500
H	-2.93312000	0.98282200	3.92065300
H	-7.70616700	0.66254500	-0.01640000
H	-8.74155600	-0.02751300	-2.16263900
H	-7.05552800	-3.95025000	-1.69046800

H	-6.02869000	-3.23721400	0.45308400
N	4.89691000	-6.09431300	-1.11201100
N	-8.50899100	-2.41843000	-3.31552700
H	-9.30428500	-1.88098900	-3.62732400
H	-8.64909300	-3.40976900	-3.44260500
H	5.40420000	-6.54786100	-1.85700900
H	4.75091600	-6.70435100	-0.32166700

2.5.2 Coordinates of Biscarbene

(1) Biscarbene-H

C	4.55391900	-4.82814600	-1.39188000
C	4.24374300	-4.29130500	-0.14246000
C	4.67663000	-3.01353100	0.20074500
C	5.41440100	-2.28044300	-0.72334200
C	5.73486300	-2.79999600	-1.97046800
C	5.30113600	-4.08279800	-2.30141600
O	5.91061900	-1.02596700	-0.38500800
C	5.04828200	0.01338200	-0.19832900
C	3.70564500	-0.02042700	-0.59580500
C	2.90681600	1.08454500	-0.36206600
C	3.42552200	2.27277900	0.22046600
C	4.80954700	2.28394400	0.54358000
C	5.59983300	1.16744700	0.37739900
C	2.66825700	3.45791100	0.44286600
C	1.24796700	3.33111100	0.60271600
C	0.41009400	4.36054400	0.09659300
C	-0.96633600	4.30292300	0.25324800
C	-1.55184000	3.24883800	0.95190900
C	-0.75100400	2.24289900	1.55258500
C	0.64621600	2.30940400	1.36736100
C	-1.29411600	1.20263000	2.37590600
C	-2.49856300	0.57188300	1.95113400
C	-2.92034000	0.47952500	0.59704300
C	-4.06942100	-0.20293700	0.23642800
C	-4.86737800	-0.76993900	1.23773800
C	-4.48826200	-0.69806300	2.58659700
C	-3.30703800	-0.07475300	2.92522900
O	-6.01653800	-1.45811400	0.98164500
C	-6.56113700	-1.43000600	-0.29835600
C	-7.20671300	-0.28502500	-0.75289900
C	-7.79523100	-0.29747800	-2.01467900
C	-7.74116100	-1.44536100	-2.80496700
C	-7.09678900	-2.58581300	-2.33008900
C	-6.50043100	-2.58268500	-1.07011700
H	4.21765200	-5.82435200	-1.65261700
H	3.66887000	-4.87000400	0.57074900
H	4.44886200	-2.58374900	1.16838100
H	6.31660300	-2.20154400	-2.66023600
H	5.54800500	-4.49705300	-3.27171600
H	3.30316400	-0.90423800	-1.07237500
H	1.86836700	1.06063200	-0.66655000
H	5.22039800	3.19915900	0.95160700
H	6.64678300	1.16011500	0.65341100
H	0.88048500	5.19511000	-0.40848500
H	-1.58954700	5.09142100	-0.15417600
H	-2.62458000	3.22465400	1.09930900
H	1.26133800	1.56880000	1.86228700
H	-2.30151100	0.91866500	-0.17528400
H	-4.35497900	-0.29630700	-0.80282700
H	-5.12281800	-1.16508000	3.32935200
H	-2.97197000	-0.04291100	3.95466100
H	-7.24305000	0.59506500	-0.12274700
H	-8.30188400	0.58895900	-2.37744200

H	-8.20327400	-1.45124600	-3.78469800
H	-7.05638800	-3.48116200	-2.93895200
H	-5.99467900	-3.45667700	-0.67925800

(2) Biscarbene -Me

C	4.74598600	-4.47007800	-1.15449700
C	4.52893400	-3.89349600	0.10222800
C	4.93973200	-2.59430400	0.38164800
C	5.56976800	-1.85516400	-0.61288100
C	5.79892800	-2.39725600	-1.86878200
C	5.38568800	-3.70201800	-2.13095200
O	6.03697000	-0.57168800	-0.34106100
C	5.14593400	0.44852700	-0.19658200
C	3.78926800	0.33909600	-0.52836900
C	2.96007900	1.43005800	-0.33898500
C	3.45730300	2.67519600	0.13291500
C	4.85267500	2.75776400	0.39172100
C	5.67704600	1.66073900	0.26963700
C	2.66619700	3.84617600	0.30526300
C	1.25829000	3.68303800	0.53020700
C	0.36498500	4.64381900	-0.01448600
C	-1.00166100	4.55075200	0.20014300
C	-1.52189700	3.53087000	0.99465800
C	-0.66287300	2.59909700	1.63328200
C	0.72315700	2.69970700	1.38892500
C	-1.13736600	1.60349600	2.54935400
C	-2.33031500	0.90070900	2.21563900
C	-2.79703000	0.69797700	0.88858300
C	-3.93197400	-0.04781000	0.62056600
C	-4.67149400	-0.57105700	1.68869100
C	-4.24643500	-0.39055200	3.01391200
C	-3.07845400	0.29638300	3.26266100
O	-5.80516000	-1.30967100	1.52632500
C	-6.36838700	-1.42429600	0.25752600
C	-7.11936300	-0.37856000	-0.26543400
C	-7.71634600	-0.53066800	-1.51290000
C	-7.58011700	-1.71632500	-2.24368000
C	-6.82129400	-2.75022900	-1.68824700
C	-6.21404800	-2.61229500	-0.44180600
H	4.03425400	-4.47011700	0.87635500
H	4.77148700	-2.15111700	1.35556400
H	6.29258600	-1.79908400	-2.62455500
H	5.56296300	-4.12634700	-3.11318600
H	3.40060800	-0.59130000	-0.92003900
H	1.91104800	1.34751900	-0.59277300
H	5.24521000	3.71412300	0.71461500
H	6.73437200	1.70999100	0.49773900
H	0.78526300	5.45518700	-0.59581000
H	-1.66814000	5.28589800	-0.23753300
H	-2.58685000	3.48086000	1.18559900
H	1.38310200	2.01838200	1.91056900
H	-2.22385300	1.10380200	0.06450900
H	-4.25221200	-0.22313500	-0.39772200
H	-4.83586500	-0.82581800	3.81117100
H	-2.70792200	0.41290300	4.27370200
H	-7.22713200	0.53908600	0.29987300
H	-8.30105100	0.28542900	-1.92350100
H	-6.70019700	-3.67797700	-2.23676300
H	-5.62420800	-3.41149900	-0.01034400
C	4.32753900	-5.89152400	-1.43413100
H	5.09553000	-6.59751900	-1.10100800
H	3.40250300	-6.14381100	-0.91084100
H	4.17009900	-6.05652400	-2.50189900
C	-8.26022200	-1.88278200	-3.57913400
H	-7.74400700	-2.61864100	-4.19892400

H	-9.29249600	-2.22526700	-3.45150000
H	-8.29414900	-0.93928300	-4.12837200

(3) Biscarbene -NO_2

C	-1.93868400	3.83504500	-1.50884000
C	-1.34780700	2.66008600	-1.05543300
C	-2.04975600	1.46561300	-1.15322400
C	-3.33555200	1.46708000	-1.70482300
C	-3.92227900	2.65733800	-2.14980300
C	-3.22367100	3.84617000	-2.05145400
O	-4.10112900	0.34427000	-1.83929800
C	-3.54909300	-0.88541700	-1.50526900
C	-2.85801500	-1.60587200	-2.47689800
C	-2.35624500	-2.85726400	-2.15660200
C	-2.43857300	-3.35824500	-0.83488200
C	-3.17086500	-2.60372500	0.11872300
C	-3.75203700	-1.39330100	-0.22520900
C	-1.76443900	-4.58549900	-0.54099200
C	-1.07182300	-4.65590800	0.71270900
C	-0.77444100	-5.92332500	1.27399800
C	-0.06020200	-6.03162800	2.46216600
C	0.41195900	-4.88784500	3.09761800
C	0.22834400	-3.60099300	2.53092400
C	-0.53724700	-3.51270800	1.34895700
C	0.68294100	-2.44130700	3.24340600
C	1.02053100	-1.28187600	2.48019000
C	1.58004400	-1.31102900	1.17362400
C	1.89781800	-0.14507600	0.49895800
C	1.61473300	1.08619800	1.09881400
C	1.04925500	1.15791300	2.37370300
C	0.80354000	-0.01134800	3.07018900
O	1.81802400	2.28097900	0.45019900
C	2.95386900	2.51051700	-0.29282500
C	4.18075900	1.91483700	0.00820600
C	5.29440100	2.23395200	-0.75536600
C	5.16363500	3.14579900	-1.79772600
C	3.94766000	3.75925500	-2.08620600
C	2.83298500	3.44004800	-1.32605500
H	-0.35582500	2.68662700	-0.62597100
H	-1.60364400	0.54589100	-0.80133400
H	-4.91909500	2.62401500	-2.56958000
H	-3.65165600	4.77904100	-2.38993500
H	-2.75059600	-1.19033700	-3.47126100
H	-1.85316900	-3.46105900	-2.90122700
H	-3.29126100	-2.99186600	1.12219100
H	-4.32700100	-0.81800100	0.49010000
H	-1.13912500	-6.80294100	0.75721300
H	0.12651400	-7.00696700	2.89569500
H	0.95173700	-4.95461500	4.03486600
H	-0.72115400	-2.53980800	0.91759900
H	1.78787900	-2.26632400	0.70852600
H	2.34337800	-0.17786400	-0.48716400
H	0.85386300	2.13172200	2.80463500
H	0.41800400	0.01684600	4.08172300
H	4.26632600	1.21878000	0.83136900
H	6.25981400	1.79416200	-0.54907900
H	3.88772300	4.47455500	-2.89409700
H	1.87630700	3.91305400	-1.50260500
N	-1.19457800	5.09385300	-1.42111500
O	-1.76747100	6.12518800	-1.74941100
O	-0.03153600	5.04565400	-1.02398000
N	6.34530300	3.47591200	-2.60941500
O	7.40161400	2.91933800	-2.33049600
O	6.20439300	4.28609200	-3.51770300

(4) Biscarbene -NH_2

C	4.64998000	-4.81013100	-0.58814700
C	4.49290800	-4.14565200	0.63737900
C	4.93906300	-2.83985100	0.79929200
C	5.54061600	-2.18107000	-0.26563800
C	5.70746200	-2.81898400	-1.48666300
C	5.26433900	-4.12722000	-1.64733400
O	6.03960700	-0.88870800	-0.09635300
C	5.18070800	0.16576600	-0.14919000
C	3.82713100	0.04345100	-0.48997000
C	3.03043400	1.17444100	-0.49873800
C	3.55842700	2.46507800	-0.22358000
C	4.95061100	2.54781300	0.05359000
C	5.74087800	1.42246300	0.13009500
C	2.80290100	3.67054500	-0.26537100
C	1.38500400	3.58656800	-0.05983400
C	0.53646100	4.46282000	-0.78762800
C	-0.83750100	4.44232800	-0.60286400
C	-1.40954300	3.58747900	0.33751800
C	-0.59633100	2.75819600	1.15269300
C	0.79786300	2.77900900	0.93700300
C	-1.12648600	1.95035400	2.21218700
C	-2.32471300	1.22229000	1.96411200
C	-2.75712800	0.80353900	0.67656300
C	-3.89996000	0.04229900	0.50214500
C	-4.68175300	-0.27955300	1.61914900
C	-4.29231000	0.12010700	2.90752900
C	-3.11780900	0.82007600	3.07370800
O	-5.82985700	-1.00810300	1.54848700
C	-6.30867700	-1.39949900	0.29719400
C	-7.11718600	-0.53919500	-0.43421300
C	-7.63457500	-0.95202500	-1.65612800
C	-7.35358000	-2.23160700	-2.15777600
C	-6.53848100	-3.08482500	-1.39994300
C	-6.01951100	-2.67107300	-0.17832800
H	4.01892100	-4.65716400	1.46801300
H	4.82102300	-2.32929100	1.74725600
H	6.18403900	-2.29022700	-2.30287900
H	5.39279100	-4.62357400	-2.60306100
H	3.41600200	-0.92685000	-0.73362800
H	1.98372800	1.08190600	-0.75868500
H	5.36757600	3.53226900	0.22702400
H	6.79469000	1.47728300	0.37297900
H	0.99617600	5.15155100	-1.48563100
H	-1.46978500	5.10766500	-1.18066100
H	-2.48027900	3.59749900	0.50037200
H	1.42332200	2.17967200	1.58623900
H	-2.15189800	1.05413800	-0.18555300
H	-4.19534800	-0.29779500	-0.48126300
H	-4.91483600	-0.16217500	3.74740900
H	-2.77476600	1.10231800	4.06155900
H	-7.33788800	0.44721300	-0.04496800
H	-8.26288100	-0.27795200	-2.22807900
H	-6.30900600	-4.07742600	-1.77187700
H	-5.39202700	-3.32992300	0.40921500
N	4.15304400	-6.10252000	-0.76573000
N	-7.82731900	-2.62511000	-3.40979200
H	-8.64620600	-2.13814600	-3.74203900
H	-7.88531100	-3.62056200	-3.56401500
H	4.57759800	-6.63564200	-1.50989600
H	4.04001500	-6.64676300	0.07647700

2.6 Excitation state of bisdiazo compounds in different solvents

Here, the noticeable major peaks were analyzed and the absorption peak with the most great value was further analysis with excited states that contributed more than 10% at least via Multiwfn 3.8 dev.

2.6.1 Analysis of UV-Vis Spectrum in THF

(1) Bisdiazo-H

Extrema on the spectrum curve:

Maximum	1	X:	199.2587	Value:	69261.9890
**Maximum**	**2**	**X:**	**272.0144**	**Value:**	**81390.7629**
Maximum	3	X:	503.1439	Value:	218.3452

Individual transition state contribution to the **max absorption peak @ 272.0144 nm** band in UV-Vis spectrum is as the followings:

#Transition	Contribution	%
**7**	**20842.94165**	**25.608**
**8**	**18461.43805**	**22.682**
**9**	**16105.98168**	**19.788**
**6**	**9616.38497**	**11.815**
**5**	**9465.58518**	**11.630**
12	1908.48899	2.345
4	1685.21646	2.071
11	1228.37056	1.509
10	973.59876	1.196
3	712.00720	0.875

Sum of contribution from highlighted % = 91.52 3%

(2) Bisdiazo-Me

Extrema on the spectrum curve:

Maximum	1	X:	200.2854	Value:	50578.4449
**Maximum**	**2**	**X:**	**268.8248**	**Value:**	**80071.0917**
Maximum	3	X:	506.5501	Value:	218.2844

Individual transition state contribution to the **max absorption peak @ 268.8248 nm** band in UV-Vis spectrum is as the followings:

#Transition	Contribution	%
**9**	**27427.39582**	**34.254**
**8**	**23228.30801**	**29.010**
**5**	**12956.80871**	**16.182**
7	7034.45014	8.785
12	3228.47366	4.032
11	2503.17779	3.126
4	1176.75490	1.470
6	920.48752	1.150
10	701.68252	0.876
3	475.06498	0.593

Sum of contribution from highlighted % = 79.446 %

(3) Bisdiazo-NO_2

Extrema on the spectrum curve:

Maximum	1	X:	213.4872	Value:	27866.2122

Maximum 2 X: 279.8010 Value: 101255.1018
Maximum 3 X: 496.2001 Value: 210.2717

Individual transition state contribution to the **max absorption peak @ 279.8010 nm** band in UV-Vis spectrum is as the followings:

#Transition	Contribution	%
**8**	**18782.71540**	**18.550**
**9**	**16224.60802**	**16.023**
**11**	**13285.94333**	**13.121**
**14**	**12460.48058**	**12.306**
15	7580.26498	7.486
6	6717.16804	6.634
5	6560.97250	6.480
10	4903.01416	4.842
12	3686.12557	3.640
19	2974.92608	2.938

Sum of contribution from highlighted % = 60.00 %

(4) Bisdiazo-NH_2

Extrema on the spectrum curve:
Maximum 1 X: 265.5975 Value: 82962.9572
Maximum 2 X: 506.1206 Value: 218.3818

Individual transition state contribution to the **max absorption peak @ 265.5975 nm** band in UV-Vis spectrum is as the followings:

#Transition	Contribution	%
**8**	**24658.26515**	**29.722**
**11**	**16640.25055**	**20.057**
**5**	**13066.87566**	**15.750**
12	7609.40536	9.172
14	4988.32408	6.013
13	3888.32612	4.687
10	3399.68554	4.098
7	2976.45457	3.588
9	2066.78448	2.491
6	1260.68268	1.520

Sum of contribution from highlighted % = 65.526 %

2.6.2 Analysis of UV-Vis Spectrum in Toluene

(1) Bisdiazo-H

Extrema on the spectrum curve:
Maximum 1 X: 199.6167 Value: 75124.5633
Maximum 2 X: 273.4425 Value: 83544.7109
Maximum 3 X: 502.3024 Value: 246.6213

Individual transition state contribution to the **max absorption peak @ 273.4425 nm** band in UV-Vis spectrum is as the followings:

#Transition Contribution %

**8**	**20101.85824**	**24.061**
**7**	**16894.13516**	**20.222**
**9**	**16227.78407**	**19.424**
**6**	**13182.10231**	**15.779**
**5**	**9979.84567**	**11.946**
4	1891.08349	2.264
12	1883.16089	2.254
11	1285.93491	1.539
10	944.48573	1.131
3	785.21397	0.940

Sum of contribution from highlighted % = 91.432 %

(2) Bisdiazo-Me

Extrema on the spectrum curve:

Maximum	1	X:	200.8213	Value:	50509.4382
**Maximum**	**2**	**X:**	**269.6745**	**Value:**	**80907.8286**
Maximum	3	X:	505.1288	Value:	242.6058

Individual transition state contribution to the **max absorption peak @ 269.6745 nm** band in UV-Vis spectrum is as the followings:

#Transition	Contribution	%
**9**	**26788.91015**	**33.110**
**8**	**24438.21527**	**30.205**
**5**	**14638.08420**	**18.092**
7	4779.25350	5.907
12	3317.28423	4.100
11	2611.98931	3.228
4	1424.97480	1.761
10	1083.00835	1.339
6	942.98874	1.166
3	484.46408	0.599

Sum of contribution from highlighted % = 81.407 %

(3) Bisdiazo-NO_2

Extrema on the spectrum curve:

Maximum	1	X:	212.5486	Value:	30644.9050
**Maximum**	**2**	**X:**	**278.5767**	**Value:**	**108697.6909**
Maximum	3	X:	494.3268	Value:	234.4992

Individual transition state contribution to the **max absorption peak @ 278.5767 nm** band in UV-Vis spectrum is as the followings:

#Transition	Contribution	%
**11**	**21075.25380**	**19.389**
**8**	**19785.29956**	**18.202**
**7**	**12171.52462**	**11.198**
**10**	**11032.46134**	**10.150**
6	9560.75929	8.796
9	9478.34629	8.720
13	5971.54829	5.494

18	4561.72906	4.197
14	4091.12331	3.764
17	2645.16841	2.434

Sum of contribution from highlighted % = 58.939%

(4) Bisdiazo-NH_2

Extrema on the spectrum curve:

Maximum 1 X: 267.2416 Value: 85224.3122

Maximum 2 X: 505.5660 Value: 238.6010

Individual transition state contribution to the **max absorption peak @ 267.2416 nm** band in UV-Vis spectrum is as the followings:

#Transition	Contribution	%
**8**	**25946.46071**	**30.445**
**5**	**13811.73879**	**16.206**
**11**	**13329.82447**	**15.641**
**9**	**9993.11770**	**11.726**
14	4820.03899	5.656
7	3897.83214	4.574
10	3710.20679	4.353
13	3072.01585	3.605
12	2697.07302	3.165
6	1505.54572	1.767

Sum of contribution from highlighted % = 74.018 %

2.6.3 Analysis of UV-Vis Spectrum in Chlorobenzene

(1) Bisdiazo-H

Extrema on the spectrum curve:

Maximum 1 X: 199.6309 Value: 77789.0753

Maximum 2 X: 273.1670 Value: 84601.1468

Maximum 3 X: 503.2598 Value: 238.5571

Individual transition state contribution to the **max absorption peak @ 273.1670 nm** band in UV-Vis spectrum is as the followings:

#Transition	Contribution	%
**7**	**20276.04491**	**23.967**
**8**	**18703.80613**	**22.108**
**9**	**15852.65766**	**18.738**
**6**	**12010.75751**	**14.197**
**5**	**10663.93362**	**12.605**
4	1861.15877	2.200
12	1835.31558	2.169
11	1223.45390	1.446
3	974.56604	1.152
10	837.72534	0.990

Sum of contribution from highlighted % = 91.615 %

(2) Bisdiazo-Me

Extrema on the spectrum curve:

Maximum	1	X:	200.3308	Value: 55955.0514
**Maximum**	**2**	**X:**	**269.5644**	**Value: 82283.4552**
Maximum	3	X:	506.7125	Value: 234.5110

Individual transition state contribution to the **max absorption peak @ 269.5644 nm** band in UV-Vis spectrum is as the followings:

#Transition	Contribution	%
**9**	**27940.33694**	**33.956**
**8**	**23948.27274**	**29.105**
**5**	**14152.94143**	**17.200**
7	6076.48240	7.385
12	3172.10030	3.855
11	2727.04297	3.314
4	1365.95943	1.660
10	1095.51781	1.331
6	856.10440	1.040
3	587.50661	0.714

Sum of contribution from highlighted % = 80.261 %

(3) Bisdiazo-NO_2

Extrema on the spectrum curve:

Maximum	1	X:	213.5681	Value: 29957.0821
**Maximum**	**2**	**X:**	**280.3759**	**Value: 106118.1457**
Maximum	3	X:	496.1196	Value: 226.4420

Individual transition state contribution to the **max absorption peak @ 280.3759 nm** band in UV-Vis spectrum is as the followings:

#Transition	Contribution	%
**9**	**15551.80360**	**14.655**
**8**	**14779.55104**	**13.927**
**11**	**13961.97851**	**13.157**
**14**	**12374.03919**	**11.661**
10	10381.97546	9.783
6	9849.46167	9.282
15	7571.61485	7.135
5	7158.78006	6.746
18	3772.07680	3.555
12	3581.40340	3.375

Sum of contribution from highlighted % = 50.400 %

(4) Bisdiazo-NH_2

Extrema on the spectrum curve:

**Maximum**	**1**	**X:**	**266.5425**	**Value: 86096.7542**
Maximum	2	X:	506.3797	Value: 238.6058

Individual transition state contribution to the **max absorption peak @ 266.5425 nm** band in UV-Vis spectrum is as the followings:

#Transition Contribution %

**8**	**26774.31536**	**31.098**
**11**	**18570.57634**	**21.569**
**5**	**13485.71378**	**15.663**
12	5117.44575	5.944
14	4887.47167	5.677
10	3812.61675	4.428
13	3720.86375	4.322
7	3338.22896	3.877
9	2366.88595	2.749
6	1396.67738	1.622

Sum of contribution from highlighted % = 68.330 %

2.6.4. Hole and electron distribution of bisdiazo-X compounds

Hereby the following isoplots from Figure S6-S9 are the electron and hole distribution of bisdiazo-X compounds via SMD mode in another solvents, like in both toluene and chlorobenzene. In the plot, the isosurface in green stands for electron distribution and that in blue for hole distribution, and the state transition from ground (S0) to certain ones (S#) are labeled as S0 -> S# as portrayed in each plot, which tells the electron transfer and distribution of the electron and hole when the compounds is excited to the specific one from its ground state.

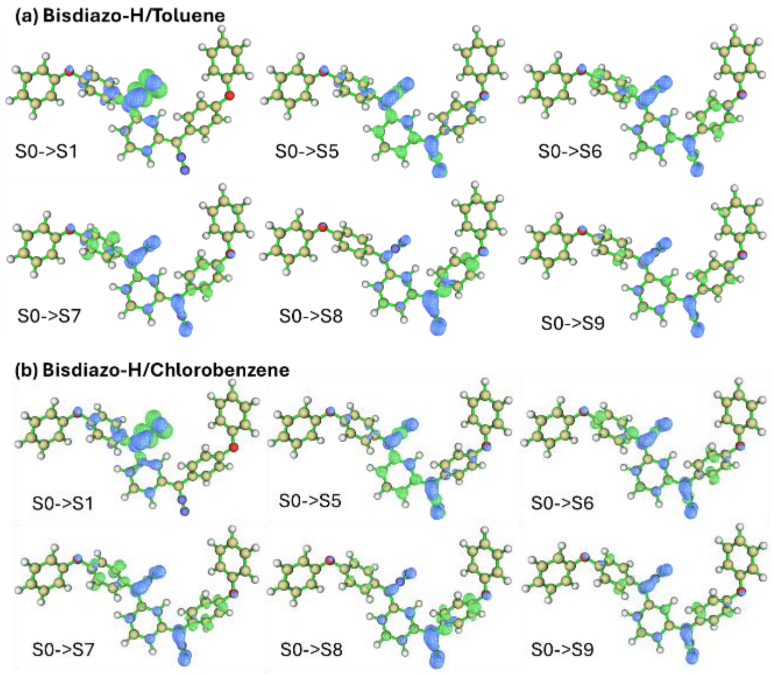

Figure S6. Hole and electron distribution of bisdiazo-H in (a) toluene and (b) chlorobenezene (isovalue=0.0025 a.u.)

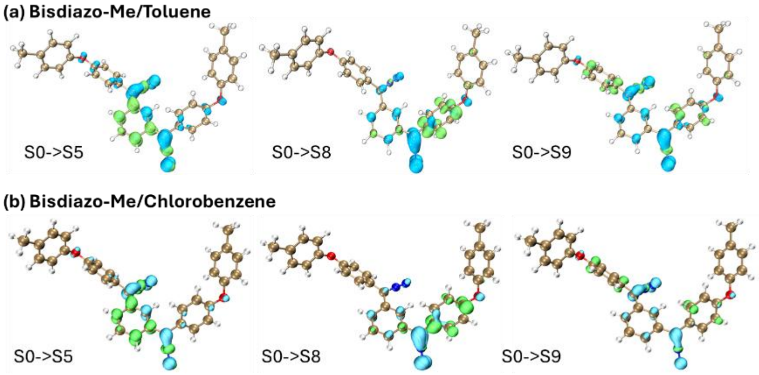

Figure S7. Hole and electron distribution of bisdiazo-Me in (a) toluene and (b) chlorobenezene

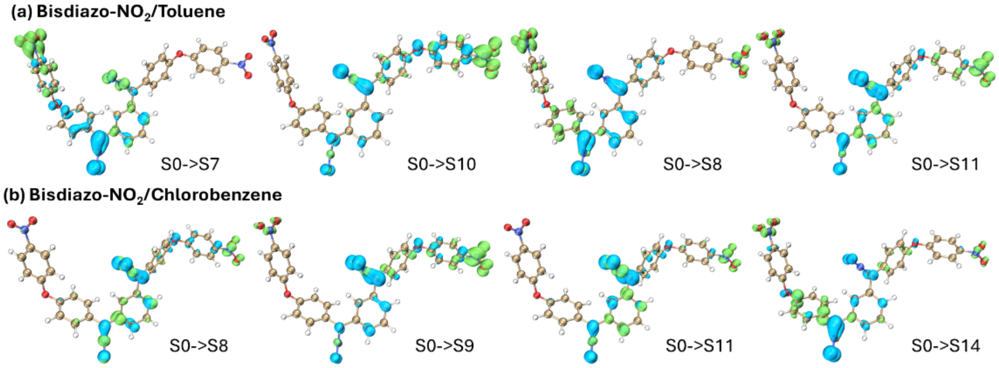

Figure S8. Hole and electron distribution of bisdiazo-NO_2 in (a) toluene and (b) chlorobenezene

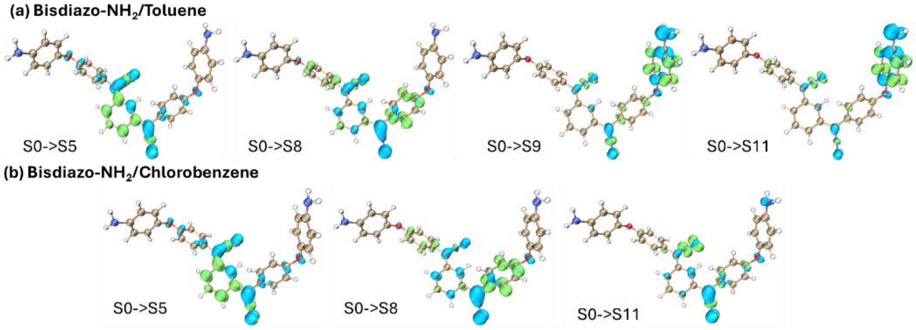

Figure S9. Hole and electron distribution of bisdiazo-NH_2 in (a) toluene and (b) chlorobenezene

2.7 Physisorption of Bidiazo and its carbene species onto Graphene

2.7.1 Optimization of Single-layer Graphene

Here, as single-layer graphene as example and download a unit cell of graphene (1200017.cif) from the Crystallography Open Database (COD), and this type of unit cell graphene is reported by paper DOI:10.1007/BF01327534. Then such unit cell was expanded to be a single-layer one for cluster model to be built up, followed by a geometry pre-optimization at theory level of B3LYP-D3BJ/6-311G* with the edge carbon atoms saturated by hydrogens manually, and all the carbon atoms were fixed (labeled with a number of -1 in the coordinate file showing bellowed) before optimization was carried out.

Coordinate of geometry optimized single-layer graphene

C	-1	6.44518200	-9.16715300	-0.01757900
C	-1	5.06531100	-9.52723900	0.01603400
C	-1	2.68371000	-8.87237100	0.01571000
C	-1	0.30210500	-8.21750100	0.01538300
C	-1	-2.07950100	-7.56263000	0.01505800
C	-1	-4.46110600	-6.90775900	0.01473300

C	-1	4.06357300	-8.51226600	-0.01790500
C	-1	1.68196400	-7.85739800	-0.01822900
C	-1	-0.69964100	-7.20252600	-0.01855500
C	-1	-3.08124600	-6.54765600	-0.01888000
C	-1	-5.46285100	-5.89278500	-0.01920500
C	-1	8.20311700	-7.43206200	-0.01689300
C	-1	9.96105500	-5.69696700	-0.01620500
C	-1	11.71899300	-3.96187200	-0.01551700
C	-1	13.47693000	-2.22677700	-0.01483400
C	-1	15.23486800	-0.49168200	-0.01413600
C	-1	16.99280100	1.24341300	-0.01353000
C	-1	6.82324500	-7.79212800	0.01672200
C	-1	8.58118500	-6.05703300	0.01740800
C	-1	10.33912200	-4.32193800	0.01809600
C	-1	12.09706000	-2.58684200	0.01878200
C	-1	13.85499700	-0.85174700	0.01947300
C	-1	15.61293100	0.88335500	0.02014000
C	-1	17.37087600	2.61842800	0.02087000
C	-1	4.44165000	-7.13726600	0.01639400
C	-1	6.19958800	-5.40217000	0.01708300
C	-1	7.95752500	-3.66707500	0.01777000
C	-1	9.71546200	-1.93198000	0.01845700
C	-1	11.47340000	-0.19688400	0.01914500
C	-1	13.23133700	1.53821100	0.01983200
C	-1	14.98927200	3.27329700	0.02054300
C	-1	2.06004500	-6.48239500	0.01607000
C	-1	3.81798200	-4.74729900	0.01675800
C	-1	5.57592000	-3.01220400	0.01744500
C	-1	7.33385700	-1.27710900	0.01813200
C	-1	9.09179400	0.45798700	0.01881900
C	-1	10.84973200	2.19308200	0.01950700
C	-1	12.60766500	3.92816600	0.02018400
C	-1	-0.32156000	-5.82752400	0.01574500
C	-1	1.43637700	-4.09242900	0.01643200
C	-1	3.19431400	-2.35733300	0.01712000
C	-1	4.95225200	-0.62223800	0.01780700
C	-1	6.71018900	1.11285700	0.01849400
C	-1	8.46812600	2.84795300	0.01918100
C	-1	10.22606000	4.58303700	0.01987000
C	-1	-2.70316600	-5.17265300	0.01542000
C	-1	-0.94522800	-3.43755800	0.01610700
C	-1	0.81270900	-1.70246200	0.01679400
C	-1	2.57064600	0.03263300	0.01748200
C	-1	4.32858400	1.76772800	0.01816900
C	-1	6.08652100	3.50282300	0.01885600
C	-1	7.84445500	5.23790700	0.01954400
C	-1	5.82150400	-6.77718400	-0.01721500
C	-1	7.57944200	-5.04208900	-0.01652900
C	-1	9.33737900	-3.30699300	-0.01584200
C	-1	11.09531600	-1.57189800	-0.01515500
C	-1	12.85325400	0.16319800	-0.01446300
C	-1	14.61119400	1.89828900	-0.01372400
C	-1	16.36912800	3.63340400	-0.01305000
C	-1	3.43989900	-6.12231300	-0.01754200
C	-1	5.19783600	-4.38721800	-0.01685500
C	-1	6.95577400	-2.65212200	-0.01616800
C	-1	8.71371100	-0.91702700	-0.01548000
C	-1	10.47164800	0.81806800	-0.01479300
C	-1	12.22958600	2.55316300	-0.01409900
C	-1	13.98752500	4.28826600	-0.01348500
C	-1	1.05829400	-5.46744200	-0.01786700
C	-1	2.81623100	-3.73234700	-0.01718000
C	-1	4.57416800	-1.99725200	-0.01649300
C	-1	6.33210600	-0.26215600	-0.01580600
C	-1	8.09004300	1.47293900	-0.01511800

C	-1	9.84798100	3.20803400	-0.01442800
C	-1	11.60592100	4.94314100	-0.01374400
C	-1	-1.32331200	-4.81257100	-0.01819300
C	-1	0.43462600	-3.07747600	-0.01750500
C	-1	2.19256300	-1.34238100	-0.01681800
C	-1	3.95050000	0.39271500	-0.01613100
C	-1	5.70843800	2.12781000	-0.01544400
C	-1	7.46637500	3.86290500	-0.01475600
C	-1	9.22431600	5.59801300	-0.01407100
C	-1	-3.70491700	-4.15770100	-0.01851800
C	-1	-1.94698000	-2.42260500	-0.01783100
C	-1	-0.18904200	-0.68751000	-0.01714300
C	-1	1.56889500	1.04758500	-0.01645600
C	-1	3.32683200	2.78268100	-0.01576900
C	-1	5.08477000	4.51777600	-0.01508100
C	-1	6.84271000	6.25288300	-0.01439500
C	-1	-6.84271100	-6.25288800	0.01440800
C	-1	-7.84445700	-5.23791400	-0.01953100
C	-1	-9.22431700	-5.59801800	0.01408300
C	-1	-11.60592300	-4.94314800	0.01375700
C	-1	-13.98752800	-4.28827700	0.01343300
C	-1	-16.36912600	-3.63340300	0.01310700
C	-1	-10.22606200	-4.58304300	-0.01985600
C	-1	-12.60766700	-3.92817200	-0.02018100
C	-1	-14.98927400	-3.27329800	-0.02050600
C	-1	-17.37088300	-2.61844300	-0.02083200
C	-1	-5.08477100	-4.51778200	0.01509500
C	-1	-3.32683400	-2.78268700	0.01578200
C	-1	-1.56889600	-1.04759200	0.01646900
C	-1	0.18904100	0.68750400	0.01715600
C	-1	1.94697800	2.42259900	0.01784400
C	-1	3.70491600	4.15769400	0.01853100
C	-1	5.46285000	5.89277900	0.01921900
C	-1	-6.08652200	-3.50283000	-0.01884300
C	-1	-4.32858500	-1.76773400	-0.01815600
C	-1	-2.57064800	-0.03263900	-0.01746900
C	-1	-0.81271000	1.70245600	-0.01678100
C	-1	0.94522700	3.43755100	-0.01609400
C	-1	2.70316400	5.17264700	-0.01540700
C	-1	4.46110500	6.90775300	-0.01472000
C	-1	-7.46637700	-3.86291100	0.01476900
C	-1	-5.70843900	-2.12781600	0.01545700
C	-1	-3.95050200	-0.39272100	0.01614400
C	-1	-2.19256400	1.34237400	0.01683100
C	-1	-0.43462700	3.07747000	0.01751900
C	-1	1.32331000	4.81256500	0.01820600
C	-1	3.08124500	6.54765000	0.01889300
C	-1	-9.84798200	-3.20804100	0.01444400
C	-1	-8.09004500	-1.47294500	0.01513100
C	-1	-6.33210700	0.26215000	0.01581900
C	-1	-4.57417000	1.99724500	0.01650600
C	-1	-2.81623200	3.73234100	0.01719300
C	-1	-1.05829500	5.46743600	0.01788100
C	-1	0.69963900	7.20252000	0.01856800
C	-1	-12.22958700	-2.55317000	0.01411900
C	-1	-10.47165000	-0.81807500	0.01480600
C	-1	-8.71371300	0.91702100	0.01549400
C	-1	-6.95577500	2.65211600	0.01618100
C	-1	-5.19783800	4.38721100	0.01686800
C	-1	-3.43990000	6.12230700	0.01755500
C	-1	-1.68196600	7.85739100	0.01824300
C	-1	-14.61119300	-1.89829900	0.01379200
C	-1	-12.85325500	-0.16320400	0.01448100
C	-1	-11.09531800	1.57189200	0.01516800
C	-1	-9.33738100	3.30698700	0.01585500

C	-1	-7.57944300	5.04208200	0.01654300
C	-1	-5.82150600	6.77717800	0.01722800
C	-1	-4.06357400	8.51226000	0.01791800
C	-1	-16.99280100	-1.24341700	0.01346900
C	-1	-15.23486700	0.49167300	0.01415700
C	-1	-13.47693100	2.22677100	0.01484300
C	-1	-11.71899400	3.96186600	0.01553100
C	-1	-9.96105700	5.69696100	0.01621800
C	-1	-8.20311900	7.43205600	0.01690600
C	-1	-6.44518300	9.16714600	0.01759200
C	-1	-8.46812800	-2.84795900	-0.01916800
C	-1	-6.71019000	-1.11286400	-0.01848100
C	-1	-4.95225300	0.62223200	-0.01779400
C	-1	-3.19431600	2.35732700	-0.01710600
C	-1	-1.43637800	4.09242200	-0.01641900
C	-1	0.32155900	5.82751800	-0.01573200
C	-1	2.07949900	7.56262400	-0.01504500
C	-1	-10.84973300	-2.19308800	-0.01949400
C	-1	-9.09179600	-0.45799300	-0.01880600
C	-1	-7.33385800	1.27710200	-0.01811900
C	-1	-5.57592100	3.01219800	-0.01743200
C	-1	-3.81798400	4.74729300	-0.01674400
C	-1	-2.06004600	6.48238800	-0.01605700
C	-1	-0.30210600	8.21749500	-0.01537000
C	-1	-13.23133900	-1.53821700	-0.01981900
C	-1	-11.47340100	0.19687800	-0.01913100
C	-1	-9.71546400	1.93197300	-0.01844400
C	-1	-7.95752600	3.66706900	-0.01775700
C	-1	-6.19958900	5.40216400	-0.01707000
C	-1	-4.44165200	7.13725900	-0.01638100
C	-1	-2.68371200	8.87236500	-0.01569600
C	-1	-15.61293600	-0.88336000	-0.02014400
C	-1	-13.85499900	0.85174200	-0.01945700
C	-1	-12.09706100	2.58683600	-0.01877000
C	-1	-10.33912400	4.32193100	-0.01808200
C	-1	-8.58118700	6.05702700	-0.01739500
C	-1	-6.82324600	7.79212100	-0.01670900
C	-1	-5.06531300	9.52723200	-0.01602100
H	0	7.20990900	-9.93504000	-0.01741300
H	0	4.77329000	-10.57086100	0.01559700
H	0	2.39736500	-9.91749900	0.02306600
H	0	0.01528300	-9.26239400	0.02209100
H	0	-2.36644700	-8.60744900	0.02201700
H	0	-4.74823300	-7.95255100	0.02227300
H	0	8.96353200	-8.20416600	-0.02461600
H	0	10.72187600	-6.46858700	-0.02357500
H	0	12.48012300	-4.73306300	-0.02298300
H	0	14.23784900	-2.99804400	-0.02145700
H	0	15.99500100	-1.26387900	-0.02193800
H	0	17.75031300	0.46820800	-0.01894600
H	0	18.41939700	2.89238400	0.01519500
H	0	14.27356700	5.33371100	-0.02321800
H	0	11.89198700	5.98821100	-0.02045500
H	0	9.51138600	6.64267200	-0.02018200
H	0	7.13030900	7.29748400	-0.02135200
H	0	-7.13040400	-7.29749300	0.02260300
H	0	-9.51236700	-6.64232400	0.02181000
H	0	-11.89327900	-5.98746200	0.02009100
H	0	-14.27355700	-5.33306900	0.02066100
H	0	-16.64908000	-4.68020300	0.01550400
H	0	-18.41997000	-2.89124800	-0.01729900
H	0	4.74846200	7.95253800	-0.02231700
H	0	-17.74916400	-0.46737100	0.01284000
H	0	-15.99570000	1.26348500	0.02476700
H	0	-14.23739900	2.99852900	0.02220000

H	0	-12.47988700	4.73312900	0.02249000
H	0	-10.72196400	6.46835800	0.02337600
H	0	-8.96360800	8.20403400	0.02465700
H	0	-7.20985300	9.93505700	0.01748000
H	0	2.36638500	8.60756300	-0.02250900
H	0	-0.01552200	9.26250800	-0.02233500
H	0	-2.39750300	9.91753300	-0.02307100
H	0	-4.77332200	10.57085300	-0.01556500
H	0	16.65197100	4.67974900	-0.01637500

2.7.2 Optimizations of Bisdiazo onto Graphene via cluster model

In the cluster model, all the edged atoms (including all the involved carbon and hydrogen atoms) were fixed and then do the optimization at theory level of r^2SCAN-3c via ORCA 5.0.3. Besides, it should be noted that the charge for all the bisdiazo compounds and its carbene species is 0, while the spin-multiplicity is 1 for bisdiazo and 3 for carbene species, respectively.

(1) Bisdiazo-H/Graphene

C	7.22603900	8.84237500	-0.66655800
C	5.85689100	9.23509000	-0.74455600
C	3.46029500	8.63895100	-0.78705800
C	1.06369900	8.04281200	-0.82956100
C	-1.33290800	7.44667300	-0.87207000
C	-3.72951800	6.85053600	-0.91458100
C	4.82939800	8.24617400	-0.70917200
C	2.43277100	7.64999300	-0.75165300
C	0.03616500	7.05377600	-0.79413500
C	-2.36038600	6.45764800	-0.83662900
C	-4.75697500	5.86158900	-0.87914100
C	8.93927400	7.06540200	-0.57703000
C	10.65252300	5.28842400	-0.48750000
C	12.36577000	3.51144500	-0.39795500
C	14.07901500	1.73446400	-0.30839500
C	15.79226300	-0.04251800	-0.21887000
C	17.50550200	-1.81950100	-0.12924500
C	7.57008800	7.45805500	-0.65491700
C	9.28334300	5.68107500	-0.56540800
C	10.99660000	3.90409100	-0.47588100
C	12.70985100	2.12710300	-0.38634100
C	14.42309600	0.35012000	-0.29679500
C	16.13633100	-1.42685200	-0.20728100
C	17.84962100	-3.20378700	-0.11775600
C	5.17899700	6.85025000	-0.67429100
C	6.88649100	5.07630100	-0.64050900
C	8.59611600	3.30383300	-0.54741300
C	10.30778200	1.52915000	-0.44951100
C	12.02005400	-0.24623500	-0.36287800
C	13.73358900	-2.02179800	-0.26891700
C	15.45300500	-3.79988400	-0.16027900
C	2.78205200	6.25432300	-0.70358500
C	4.49058300	4.47956600	-0.66893800
C	6.20036600	2.70642700	-0.60431700
C	7.91091600	0.93275900	-0.50726000
C	9.62318700	-0.84221500	-0.41633600
C	11.33723500	-2.61754800	-0.31645900
C	13.05641200	-4.39601100	-0.20272700
C	0.38517100	5.65874400	-0.74052700
C	2.09476800	3.88413300	-0.70593200
C	3.80600100	2.11018700	-0.66298900
C	5.51624900	0.33658400	-0.55987600
C	7.22806100	-1.43777500	-0.46029900
C	8.94168900	-3.21352800	-0.35764500
C	10.65981600	-4.99215400	-0.24526000

C	-2.01127700	5.06327600	-0.77582000
C	-0.29962600	3.28912300	-0.72835200
C	1.41153900	1.51482800	-0.72576100
C	3.12179600	-0.25916600	-0.62039800
C	4.83340800	-2.03342600	-0.50758900
C	6.54649600	-3.80937100	-0.40182200
C	8.26322000	-5.58830300	-0.28776500
C	6.53133400	6.46162500	-0.67589200
C	8.24432100	4.68554800	-0.60334400
C	9.95704300	2.90954400	-0.50148400
C	11.67076500	1.13338300	-0.41079500
C	13.38523700	-0.64240700	-0.32386900
C	15.10334700	-2.41490300	-0.22402300
C	16.82214000	-4.19265700	-0.08232700
C	4.14082300	5.86561600	-0.69036100
C	5.85315300	4.08850000	-0.65353000
C	7.56439300	2.31454200	-0.56479900
C	9.27668900	0.53990700	-0.46983800
C	10.99013900	-1.23409600	-0.37953500
C	12.70549200	-3.00543500	-0.27738200
C	14.42553500	-4.78878600	-0.12473000
C	1.74749300	5.27104300	-0.72159000
C	3.45984100	3.49400300	-0.69497900
C	5.17138500	1.71894000	-0.61959400
C	6.88243100	-0.05502800	-0.52003000
C	8.59498500	-1.82859900	-0.42384900
C	10.30883900	-3.60020500	-0.31826700
C	12.02893600	-5.38493300	-0.16730300
C	-0.64669500	4.67608600	-0.75268400
C	1.06585700	2.89858300	-0.73494700
C	2.77731500	1.12328500	-0.69185500
C	4.48820700	-0.65031700	-0.57092800
C	6.20005600	-2.42385800	-0.46813700
C	7.91291900	-4.19573900	-0.36091600
C	9.63233200	-5.98107500	-0.20982100
C	-3.04174500	4.08128600	-0.78651100
C	-1.32877800	2.30399400	-0.73894600
C	0.38287000	0.52812600	-0.72497200
C	2.09373000	-1.24577700	-0.62448000
C	3.80502800	-3.01922600	-0.51530100
C	5.51707200	-4.79131300	-0.40463200
C	7.23572700	-6.57721400	-0.25233500
C	-6.12612200	6.25440300	-0.95709900
C	-7.15362900	5.26549400	-0.92165900
C	-8.52272500	5.65826800	-0.99961600
C	-10.91932900	5.06212900	-1.04213100
C	-13.31593300	4.46598600	-1.08463900
C	-15.71253200	3.86984000	-1.12715300
C	-9.55023300	4.66934600	-0.96415900
C	-11.94682400	4.07320400	-1.00668400
C	-14.34340800	3.47707000	-1.04917300
C	-16.74002200	2.88098600	-1.09170300
C	-4.40740700	4.46788000	-0.81591400
C	-2.69506700	2.69469700	-0.74841900
C	-0.98314300	0.91966600	-0.71670100
C	0.72739100	-0.85478100	-0.66491700
C	2.43877300	-2.62894000	-0.55739300
C	4.15139600	-4.40493100	-0.44621300
C	5.86661700	-6.18445500	-0.33027800
C	-5.43675500	3.48576200	-0.82975200
C	-3.72308800	1.70904500	-0.77540900
C	-2.01060700	-0.06687400	-0.72393200
C	-0.30029800	-1.84118600	-0.66591500
C	1.40994700	-3.61456200	-0.56732400
C	3.12093600	-5.38691700	-0.44778600

C	4.83912300	-7.17335200	-0.29484700
C	-6.80342100	3.87224600	-0.86006100
C	-5.08962100	2.09923700	-0.80030800
C	-3.37720100	0.32483800	-0.75212500
C	-1.66551000	-1.44967300	-0.68915100
C	0.04506200	-3.22398800	-0.61096800
C	1.75649600	-5.00014900	-0.49248100
C	3.46998700	-6.78057300	-0.37279700
C	-9.19942800	3.27661900	-0.90500300
C	-7.48527500	1.50420000	-0.83918100
C	-5.77171300	-0.27058300	-0.82179300
C	-4.05908300	-2.04409000	-0.73355500
C	-2.34829200	-3.81818500	-0.63867700
C	-0.63773700	-5.59460400	-0.53459200
C	1.07337000	-7.37664400	-0.41532200
C	-11.59459500	2.68196300	-0.95031400
C	-9.88002200	0.91069000	-0.86943900
C	-8.16609100	-0.86415100	-0.81106600
C	-6.45374200	-2.63781100	-0.74051700
C	-4.74224400	-4.41165700	-0.64114900
C	-3.03106300	-6.18913200	-0.55926100
C	-1.32319700	-7.97274100	-0.45780400
C	-13.99304600	2.09183700	-0.98930400
C	-12.27456100	0.31928100	-0.90279800
C	-10.56063800	-1.45593100	-0.81687700
C	-8.84743800	-3.23169700	-0.72855100
C	-7.13423600	-5.00745200	-0.63986000
C	-5.42099400	-6.78489000	-0.56127000
C	-3.71978200	-8.56891800	-0.50026000
C	-16.39589900	1.49669000	-1.08011700
C	-14.68265700	-0.28029200	-0.99057200
C	-12.96941100	-2.05727800	-0.90103400
C	-11.25616600	-3.83425800	-0.81148200
C	-9.54292000	-5.61123300	-0.72193300
C	-7.82967000	-7.38821100	-0.63240100
C	-6.11642000	-9.16519200	-0.54287200
C	-7.83241600	2.89015700	-0.87354300
C	-6.11755000	1.11299500	-0.83353700
C	-4.40469400	-0.66190200	-0.79415600
C	-2.69408100	-2.43548000	-0.69817500
C	-0.98378500	-4.20946900	-0.61285200
C	0.72458700	-5.98263700	-0.49066500
C	2.44251800	-7.76948900	-0.33736500
C	-10.22697400	2.29389000	-0.91741100
C	-8.51352500	0.51801900	-0.85060000
C	-6.80064000	-1.25732800	-0.81616000
C	-5.08942000	-3.02999400	-0.71847400
C	-3.37910400	-4.80366100	-0.62631300
C	-1.67250000	-6.57799300	-0.52710400
C	0.04591300	-8.36562400	-0.37988800
C	-12.62280200	1.69845900	-0.95808600
C	-10.90948300	-0.07695100	-0.87828000
C	-9.19792100	-1.85220500	-0.79110400
C	-7.48620800	-3.62600800	-0.70819700
C	-5.77594200	-5.39956200	-0.62021100
C	-4.06914700	-7.17401000	-0.55364400
C	-2.35069300	-8.96175800	-0.42237500
C	-15.02673900	1.10404300	-1.00213200
C	-13.31350600	-0.67292800	-0.91262700
C	-11.60025400	-2.44990700	-0.82307900
C	-9.88697200	-4.22688200	-0.73354900
C	-8.17370300	-6.00384500	-0.64402500
C	-6.46046600	-7.78081500	-0.55451000
C	-4.74729300	-9.55789400	-0.46487400
H	8.00959100	9.59104100	-0.66773800

H	5.59127500	10.28518000	-0.77903200
H	3.20057700	9.69019900	-0.82922100
H	0.80347500	9.09385400	-0.87109600
H	-1.59324800	8.49763600	-0.91387100
H	-3.99001900	7.90145700	-0.95697300
H	9.71843800	7.81859600	-0.57053900
H	11.43208800	6.04111000	-0.48132300
H	13.14563100	4.26369400	-0.39165600
H	14.85868800	2.48677000	-0.30294600
H	16.57114500	0.71076800	-0.21227000
H	18.28189900	-1.06320600	-0.12516900
H	18.89046600	-3.50306800	-0.07835800
H	14.68488000	-5.84026800	-0.08015200
H	12.28838700	-6.43612200	-0.12576500
H	9.89281200	-7.03189300	-0.16886800
H	7.49671700	-7.62796500	-0.21052800
H	-6.38717600	7.30512600	-1.00015700
H	-8.78415600	6.70871000	-1.04221000
H	-11.18010100	6.11259900	-1.08331600
H	-13.57534500	5.51688000	-1.12670200
H	-15.96593800	4.92272600	-1.16428500
H	-17.78151500	3.17918800	-1.12894500
H	5.09985100	-8.22426400	-0.25240100
H	-17.17131800	0.73969100	-1.07810000
H	-15.46215900	-1.03324900	-1.00001200
H	-13.74862800	-2.81010200	-0.90720300
H	-12.03580400	-4.58656800	-0.81727300
H	-10.32257100	-6.36368900	-0.72790800
H	-8.60890600	-8.14127800	-0.63892700
H	-6.89993000	-9.91387700	-0.54174700
H	2.70277500	-8.82055000	-0.29505200
H	0.30588100	-9.41677000	-0.33807900
H	-2.09111200	-10.01304400	-0.38019400
H	-4.48170200	-10.60798800	-0.43042200
H	17.07842200	-5.24513500	-0.04420700
C	-10.58639500	2.93463200	2.65344800
C	-9.30924600	2.71655000	3.14701900
C	-8.80333300	1.42982600	3.26929300
C	-9.58035500	0.35289700	2.85888400
C	-10.86879200	0.56098300	2.38070800
C	-11.36965200	1.84769400	2.29115400
O	-9.19966700	-0.96707900	2.96424800
C	-7.89277800	-1.38249300	2.92281000
C	-6.85916100	-0.69774900	2.30094100
C	-5.59392700	-1.25375500	2.25203200
C	-5.31323500	-2.49758500	2.81654400
C	-6.37425400	-3.17544400	3.43096000
C	-7.64102400	-2.63136200	3.48281000
C	-3.97966700	-3.11049800	2.73159500
C	-2.66876400	-2.45990300	2.75053500
C	-1.49246600	-3.21389700	2.61031200
C	-0.26213600	-2.59786300	2.70746400
C	-0.14799700	-1.23033900	2.89643400
C	-1.30082600	-0.44306500	2.97467300
C	-2.54643900	-1.08464500	2.94058900
C	-1.23131700	1.01244100	3.09799000
C	-0.03805400	1.86804400	3.20381000
C	1.10642600	1.63419600	2.44166600
C	2.20077000	2.47700700	2.49990200
C	2.17252200	3.59214900	3.32534300
C	1.04094700	3.84863800	4.09187000
C	-0.04413300	2.99636200	4.03162300
O	3.16689900	4.53611500	3.38400900
C	4.49095200	4.24422500	3.13472200
C	5.07813700	3.01151200	3.39429700

C	6.43837800	2.84827300	3.17176900
C	7.21732800	3.90415900	2.72427300
C	6.62322100	5.13702200	2.49316700
C	5.26261000	5.30756300	2.68204900
N	-3.97392000	-4.41579600	2.70103900
N	-2.36663700	1.65559300	3.09907200
N	-3.96901000	-5.55692600	2.67144300
N	-3.36537400	2.21041400	3.08086800
H	-10.96991000	3.94028000	2.55071000
H	-8.69357000	3.55287700	3.45016000
H	-7.82155700	1.27258100	3.68749800
H	-11.46021000	-0.29272100	2.08369000
H	-12.37028300	2.00120300	1.91298300
H	-7.03576500	0.24725800	1.81498600
H	-4.83313200	-0.72660100	1.70568000
H	-6.20776800	-4.14235800	3.88627200
H	-8.45231100	-3.16549900	3.95637200
H	-1.53636100	-4.27849600	2.43299100
H	0.63598900	-3.19461100	2.62220500
H	0.82985500	-0.78970700	2.99009200
H	-3.43653000	-0.50231700	3.10227100
H	1.13021700	0.81178700	1.74968400
H	3.05472200	2.28503300	1.87195300
H	1.02604000	4.72015500	4.73064600
H	-0.91042100	3.20898200	4.64393900
H	4.48824600	2.19293500	3.77709700
H	6.89078200	1.88410600	3.36211500
H	8.27499900	3.76706000	2.54886300
H	7.21759100	5.96881500	2.14288900
H	4.78505300	6.25712800	2.48758100

(2) Bisdiazo-Me/Graphene

C	6.12240000	-10.15026000	0.41537400
C	4.74252900	-10.51034600	0.44898700
C	2.36092800	-9.85547800	0.44866300
C	-0.02067700	-9.20060800	0.44833600
C	-2.40228300	-8.54573700	0.44801100
C	-4.78388800	-7.89086600	0.44768600
C	3.74079100	-9.49537300	0.41504800
C	1.35918200	-8.84050500	0.41472400
C	-1.02242300	-8.18563300	0.41439800
C	-3.40402800	-7.53076300	0.41407300
C	-5.78563300	-6.87589200	0.41374800
C	7.88033500	-8.41516900	0.41606000
C	9.63827300	-6.68007400	0.41674800
C	11.39621100	-4.94497900	0.41743600
C	13.15414800	-3.20988400	0.41811900
C	14.91208600	-1.47478900	0.41881700
C	16.67001900	0.26030600	0.41942300
C	6.50046300	-8.77523500	0.44967500
C	8.25840300	-7.04014000	0.45036100
C	10.01634000	-5.30504500	0.45104900
C	11.77427800	-3.56994900	0.45173500
C	13.53221500	-1.83485400	0.45242600
C	15.29014900	-0.09975200	0.45309300
C	17.04809400	1.63532100	0.45382300
C	4.12501500	-8.10835500	0.42239900
C	5.87607100	-6.37607700	0.45477100
C	7.63032400	-4.64505300	0.45427600
C	9.38635000	-2.91377500	0.43865100
C	11.14336700	-1.18074200	0.46735300
C	12.90172600	0.55341600	0.47229700
C	14.66649000	2.29019000	0.45349600
C	1.74347000	-7.45349500	0.39234500
C	3.49510600	-5.72010300	0.39743400

C	5.24969700	-3.98888900	0.42052400
C	7.00447200	-2.25845700	0.42373300
C	8.76085200	-0.52678100	0.46290600
C	10.52014800	1.20757300	0.47505900
C	12.28488300	2.94505900	0.45313700
C	-0.63834000	-6.79911100	0.38137900
C	1.11556000	-5.06674900	0.33980000
C	2.86999700	-3.33434400	0.36597200
C	4.62522300	-1.60395600	0.40923000
C	6.38093100	0.12731200	0.45546700
C	8.13960000	1.86234100	0.48359000
C	9.90327800	3.59993000	0.45282300
C	-3.01966600	-6.14462700	0.38922300
C	-1.26397100	-4.41315600	0.34485000
C	0.49121100	-2.68053800	0.33007800
C	2.24516900	-0.95017900	0.39946100
C	4.00117300	0.78263600	0.43806900
C	5.75925200	2.51685900	0.47198500
C	7.52167300	4.25480000	0.45249700
C	5.48672300	-7.75311300	0.43929700
C	7.24397000	-6.01846500	0.45304100
C	9.00126700	-4.28488100	0.46187200
C	10.75900200	-2.55154700	0.46363000
C	12.51858100	-0.81770600	0.47938200
C	14.28155300	0.91317400	0.47111700
C	16.04634600	2.65029700	0.41990300
C	3.11177300	-7.09808100	0.39077100
C	4.86777800	-5.36288400	0.41553800
C	6.62357300	-3.63117600	0.42397400
C	8.37952800	-1.90018400	0.44766500
C	10.13773700	-0.16813200	0.48443700
C	11.89814900	1.56211100	0.48149300
C	13.66474300	3.30515900	0.41946800
C	0.73364700	-6.44512200	0.35720700
C	2.49005800	-4.70919800	0.34907000
C	4.24500200	-2.97734100	0.38741100
C	6.00102700	-1.24637100	0.40772700
C	7.75759300	0.48525200	0.45791700
C	9.51651500	2.21555800	0.46954500
C	11.28313900	3.96003400	0.41920900
C	-1.64581300	-5.79169700	0.35927200
C	0.11097400	-4.05586000	0.31173900
C	1.86646300	-2.32329700	0.34584500
C	3.62135300	-0.59257400	0.41470900
C	5.37742100	1.13910400	0.45922100
C	7.13555600	2.86979200	0.46598900
C	8.90153400	4.61490600	0.41888200
C	-4.02559000	-5.13731100	0.37176800
C	-2.26867200	-3.40236100	0.34751500
C	-0.51220000	-1.66959300	0.34388300
C	1.24201800	0.06198100	0.39844000
C	2.99688600	1.79328900	0.45917700
C	4.75449100	3.52414100	0.47010200
C	6.51992800	5.26977600	0.41855800
C	-7.16549300	-7.23599500	0.44736100
C	-8.16723900	-6.22102100	0.41342200
C	-9.54709900	-6.58112500	0.44703600
C	-11.92870500	-5.92625500	0.44671000
C	-14.31031000	-5.27138400	0.44638600
C	-16.69190800	-4.61651000	0.44606000
C	-10.54884400	-5.56615000	0.41309700
C	-12.93044900	-4.91127900	0.41277200
C	-15.31205600	-4.25640500	0.41244700
C	-17.69366500	-3.60155000	0.41212100
C	-5.40086200	-5.49017400	0.39276800

C	-3.64442200	-3.75938700	0.37313800
C	-1.88801100	-2.02745700	0.36145200
C	-0.13304000	-0.29537300	0.38751400
C	1.62150700	1.43727400	0.41777500
C	3.37904600	3.17127800	0.45573700
C	5.14006800	4.90967200	0.45217200
C	-6.40579700	-4.48314800	0.37355300
C	-4.64780400	-2.74891700	0.36806900
C	-2.89145600	-1.01619300	0.37216600
C	-1.13574100	0.71588500	0.39394300
C	0.61799300	2.44758500	0.42135600
C	2.37305500	4.17844000	0.46181200
C	4.13832300	5.92464600	0.41823300
C	-7.78211300	-4.83557500	0.39107600
C	-6.02456400	-3.10512600	0.37382900
C	-4.26727200	-1.37434500	0.38308400
C	-2.51108000	0.35736700	0.40054700
C	-0.75639500	2.09014300	0.42704100
C	0.99904600	3.82478200	0.44837300
C	2.75846300	5.56454300	0.45184600
C	-10.16205000	-4.18210700	0.37211600
C	-8.40398500	-2.45170100	0.35456500
C	-6.64741200	-0.72097300	0.36314100
C	-4.89107600	1.01104800	0.39850200
C	-3.13439600	2.74285500	0.42250600
C	-1.37937600	4.47828300	0.45809900
C	0.37685700	6.21941300	0.45152100
C	-12.54308700	-3.52836300	0.38892000
C	-10.78292800	-1.79887600	0.37036100
C	-9.02621000	-0.06767400	0.36392900
C	-7.26940200	1.66443400	0.35408700
C	-5.51266500	3.39670900	0.36795900
C	-3.75680000	5.13188700	0.43016400
C	-2.00474800	6.87428400	0.45119600
C	-14.92679200	-2.87936500	0.39691600
C	-13.16353400	-1.14870400	0.39072900
C	-11.40476800	0.58458200	0.39408900
C	-9.64729200	2.31839100	0.38515600
C	-7.88976600	4.05280200	0.36963300
C	-6.13202400	5.78705300	0.40373200
C	-4.38635600	7.52915300	0.45087100
C	-17.31558300	-2.22652400	0.44642200
C	-15.55764900	-0.49143400	0.44711000
C	-13.79971300	1.24366400	0.44779600
C	-12.04177600	2.97875900	0.44848400
C	-10.28383900	4.71385400	0.44917100
C	-8.52590100	6.44894900	0.44985900
C	-6.76796500	8.18403900	0.45054500
C	-8.78595700	-3.82820300	0.36450100
C	-7.02806500	-2.09482400	0.34629600
C	-5.27121700	-0.36300600	0.37466500
C	-3.51521700	1.36879500	0.40427900
C	-1.76073900	3.10029300	0.42762200
C	-0.00782100	4.83259700	0.44999000
C	1.75671700	6.57951700	0.41790800
C	-11.16504400	-3.17408900	0.38916700
C	-9.40700700	-1.44083800	0.37043200
C	-7.65115200	0.29154700	0.37387200
C	-5.89561100	2.02303300	0.37682600
C	-4.14096500	3.75437900	0.42247900
C	-2.38868400	5.48705500	0.45689300
C	-0.62488800	7.23438800	0.41758300
C	-13.54688200	-2.51968100	0.39895200
C	-11.78818700	-0.78645600	0.39417700
C	-10.03217400	0.94633200	0.39069400

C	-8.27687800	2.67882000	0.36929200
C	-6.52160100	4.41043200	0.37735700
C	-4.77015900	6.14206500	0.44192500
C	-3.00649400	7.88925800	0.41725700
C	-15.93571800	-1.86646700	0.41280900
C	-14.17778100	-0.13136500	0.41349600
C	-12.41984300	1.60372900	0.41418300
C	-10.66190600	3.33882400	0.41487100
C	-8.90396900	5.07392000	0.41555800
C	-7.14602800	6.80901400	0.41624400
C	-5.38809500	8.54412500	0.41693200
H	6.88712700	-10.91814700	0.41554000
H	4.45050800	-11.55396800	0.44855000
H	2.07458300	-10.90060600	0.45601900
H	-0.30749900	-10.24550100	0.45504400
H	-2.68922900	-9.59055600	0.45497000
H	-5.07101500	-8.93565800	0.45522600
H	8.64075000	-9.18727300	0.40833700
H	10.39909400	-7.45169400	0.40937800
H	12.15734100	-5.71617000	0.40997000
H	13.91506700	-3.98115100	0.41149600
H	15.67221900	-2.24698600	0.41101500
H	17.42753100	-0.51489900	0.41400700
H	18.09661500	1.90927700	0.44814800
H	13.95078500	4.35060400	0.40973500
H	11.56920500	5.00510400	0.41249800
H	9.18860400	5.65956500	0.41277100
H	6.80752700	6.31437700	0.41160100
H	-7.45318600	-8.28060000	0.45555600
H	-9.83514900	-7.62543100	0.45476300
H	-12.21606100	-6.97056900	0.45304400
H	-14.59633900	-6.31617600	0.45361400
H	-16.97186200	-5.66331000	0.44845700
H	-18.74275200	-3.87435500	0.41565400
H	4.42568000	6.96943100	0.41063600
H	-18.07194600	-1.45047800	0.44579300
H	-16.31848200	0.28037800	0.45772000
H	-14.56018100	2.01542200	0.45515300
H	-12.80266900	3.75002200	0.45544300
H	-11.04474600	5.48525100	0.45632900
H	-9.28639000	7.22092700	0.45761000
H	-7.53263500	8.95195000	0.45043300
H	2.04360300	7.62445600	0.41044400
H	-0.33830400	8.27940100	0.41061800
H	-2.72028500	8.93442600	0.40988200
H	-5.09610400	9.58774600	0.41738800
H	16.32918900	3.69664200	0.41657800
C	-9.41927600	-2.96704700	3.75822900
C	-8.11167500	-2.54892900	3.54053200
C	-7.74361100	-1.21323400	3.61493400
C	-8.70249300	-0.26092300	3.93169500
C	-10.02491500	-0.64890000	4.11472300
C	-10.37199200	-1.98376700	4.02083800
O	-8.47626800	1.09669300	4.01454900
C	-7.22126300	1.63973300	4.13143300
C	-6.18205900	1.08666400	4.87141100
C	-4.94256600	1.70560400	4.88617000
C	-4.70244300	2.87285200	4.15302400
C	-5.79853700	3.47328700	3.52642900
C	-7.03934700	2.87351600	3.52181500
C	-3.36334500	3.46636800	4.02450900
C	-2.07898500	2.77675400	3.90365200
C	-0.87895000	3.49956200	3.80152300
C	0.31773600	2.83190300	3.64495400
C	0.37839700	1.44826700	3.62512200

C	-0.79301800	0.70072400	3.77160100
C	-2.01110400	1.38489100	3.86445600
C	-0.76131200	-0.76255800	3.81505400
C	0.41569800	-1.62627600	3.97002500
C	1.55398300	-1.21302000	4.67058900
C	2.70094900	-1.98848700	4.70894000
C	2.73062900	-3.21280700	4.05043600
C	1.56455900	-3.69919100	3.47375900
C	0.43103200	-2.91633300	3.42995400
O	3.81641300	-4.04973400	3.98772700
C	5.12097100	-3.60595300	3.95079400
C	5.52292300	-2.32041600	3.61603300
C	6.87643700	-2.01698400	3.58222400
C	7.85305900	-2.96756700	3.85374800
C	7.42352600	-4.26248300	4.14175800
C	6.07979700	-4.58227500	4.19882200
N	-3.33845200	4.76648100	3.90725200
N	-1.90797800	-1.37604700	3.71004600
N	-3.32176600	5.90303700	3.80311400
N	-2.91567000	-1.90700900	3.62127000
H	-7.35450500	-3.28096400	3.28995900
H	-6.72555500	-0.92828600	3.40387900
H	-10.76796900	0.10829400	4.32149900
H	-11.40749000	-2.26810200	4.16193600
H	-6.33448500	0.18100800	5.43805000
H	-4.16006800	1.27784400	5.49371600
H	-5.67559800	4.40585800	2.99672400
H	-7.86962400	3.32972600	3.00648900
H	-0.88137000	4.57996100	3.81930800
H	1.22910000	3.40100100	3.52176200
H	1.32551400	0.96291500	3.46117800
H	-2.92643100	0.81965800	3.89323800
H	1.54459000	-0.27735400	5.20759700
H	3.56064500	-1.64163100	5.26068200
H	1.57339400	-4.67943400	3.02318100
H	-0.44514500	-3.30588700	2.93378500
H	4.80136300	-1.56543000	3.35051300
H	7.17391200	-1.01096300	3.31509600
H	8.15534700	-5.03913100	4.32673900
H	5.75441500	-5.58863100	4.42160200
C	-9.78930800	-4.41708800	3.73782000
H	-9.05695000	-5.00958200	3.19469100
H	-9.84692300	-4.82023200	4.75092800
H	-10.75945700	-4.57852700	3.27206500
C	9.30818300	-2.61513800	3.85028800
H	9.67813500	-2.45668100	4.86505300
H	9.91057200	-3.40629700	3.40781600
H	9.49610900	-1.70284500	3.28942400

(3) Bisdiazo-NO_2/Graphene

C	6.49777100	-10.28462400	-0.36683900
C	5.13311300	-10.69622700	-0.31093500
C	2.72964800	-10.13029100	-0.24806800
C	0.32618000	-9.56435400	-0.18520500
C	-2.07728900	-8.99841600	-0.12233900
C	-4.48075800	-8.43247700	-0.05947400
C	4.09429800	-9.71867000	-0.30397400
C	1.69082600	-9.15273500	-0.24110800
C	-0.71264200	-8.58679500	-0.17824300
C	-3.11611100	-8.02085800	-0.11537800
C	-5.51957900	-7.45491900	-0.05251200
C	8.19044000	-8.48580700	-0.36988000
C	9.88311100	-6.68698700	-0.37291800
C	11.57578200	-4.88816600	-0.37595700
C	13.26845200	-3.08934500	-0.37900000

C	14.96112300	-1.29052400	-0.38202900
C	16.65378800	0.50829800	-0.38514900
C	6.82577900	-8.89739000	-0.31397300
C	8.51845200	-7.09856900	-0.31701300
C	10.21112200	-5.29974800	-0.32005200
C	11.90379300	-3.50092700	-0.32309200
C	13.59646400	-1.70210600	-0.32612800
C	15.28913000	0.09672200	-0.32918700
C	16.98181000	1.89552000	-0.33218400
C	4.42724100	-8.31870800	-0.27840200
C	6.11423200	-6.52399800	-0.25125800
C	7.80462300	-4.72902200	-0.23749100
C	9.49549100	-2.93348400	-0.24093300
C	11.18688700	-1.13685700	-0.24591000
C	12.87940300	0.66006000	-0.23921600
C	14.57834300	2.46145700	-0.26932100
C	2.02313600	-7.75223300	-0.24038300
C	3.70981300	-5.95546500	-0.24281600
C	5.39991800	-4.15998900	-0.22713200
C	7.09187900	-2.36647600	-0.20043100
C	8.78337000	-0.57142400	-0.19870300
C	10.47708800	1.22584700	-0.18501800
C	12.17487200	3.02739400	-0.20648900
C	-0.38058000	-7.18663700	-0.18093900
C	1.30707000	-5.39006700	-0.21002300
C	2.99709600	-3.59298800	-0.23587800
C	4.68869500	-1.80014600	-0.17339200
C	6.38131300	-0.00588600	-0.13036900
C	8.07483100	1.79124300	-0.11906300
C	9.77140300	3.59333200	-0.14361300
C	-2.78390300	-6.62146500	-0.11536600
C	-1.09451100	-4.82573200	-0.13910300
C	0.59589200	-3.02831200	-0.16589000
C	2.28710100	-1.23398500	-0.13662100
C	3.97859900	0.55979000	-0.07329400
C	5.67218000	2.35728500	-0.05148600
C	7.36793500	4.15927000	-0.08074800
C	5.77489000	-7.91386600	-0.28563500
C	7.46753500	-6.11589700	-0.26938000
C	9.16035000	-4.31824900	-0.26811600
C	10.85309900	-2.52076700	-0.27412100
C	12.54702400	-0.72335500	-0.27410800
C	14.24389400	1.07082000	-0.27197500
C	15.94298600	2.87308000	-0.32520500
C	3.37678800	-7.34665000	-0.26615500
C	5.06847300	-5.54779800	-0.25099900
C	6.76042800	-3.75251200	-0.22738700
C	8.45238500	-1.95755400	-0.22572900
C	10.14505300	-0.16193700	-0.22488200
C	11.83969600	1.63093600	-0.21446600
C	13.53951800	3.43901100	-0.26245000
C	0.97637200	-6.78176300	-0.22273900
C	2.66681400	-4.98106900	-0.24546000
C	4.35826600	-3.18552900	-0.23338500
C	6.05054400	-1.39251400	-0.17812800
C	7.74314800	0.40191400	-0.16253100
C	9.43735700	2.19525900	-0.15199500
C	11.13605200	4.00495200	-0.19951800
C	-1.42487000	-6.21708800	-0.15824600
C	0.26552600	-4.41713000	-0.18524500
C	1.95681800	-2.61946800	-0.20820700
C	3.64877600	-0.82699300	-0.13452900
C	5.34098500	0.96697800	-0.09027900
C	7.03394600	2.76082200	-0.08613500
C	8.73258300	4.57089200	-0.13665400

C	-3.82676300	-5.65276400	-0.09453600
C	-2.13495000	-3.85332800	-0.10649500
C	-0.44398400	-2.05622600	-0.11117000
C	1.24806600	-0.26106500	-0.08141500
C	2.93859700	1.53268100	-0.04410700
C	4.63044700	3.32630300	-0.02338400
C	6.32911400	5.13682900	-0.07378800
C	-6.88422600	-7.86653900	0.00339200
C	-7.92304800	-6.88898100	0.01035200
C	-9.28769500	-7.30060100	0.06625800
C	-11.69116500	-6.73466400	0.12912200
C	-14.09463300	-6.16872600	0.19198900
C	-16.49809500	-5.60278400	0.25485300
C	-10.32651700	-6.32304300	0.07321800
C	-12.72998500	-5.75710400	0.13608300
C	-15.13345500	-5.19116300	0.19894900
C	-17.53692700	-4.62524100	0.26181300
C	-5.18688500	-6.05648600	-0.05928000
C	-3.49597000	-4.26174600	-0.07266000
C	-1.80460000	-2.46586200	-0.07165300
C	-0.11343000	-0.66974400	-0.05628600
C	1.57726200	1.12577900	-0.02487700
C	3.26930000	2.92263200	0.00209000
C	4.96446700	4.72520900	-0.01788300
C	-6.22809000	-5.08752100	-0.04639000
C	-4.53615400	-3.28924600	-0.04779000
C	-2.84374700	-1.49329400	-0.02416000
C	-1.15254700	0.30276000	-0.00280100
C	0.53724300	2.09828500	0.01260600
C	2.22679300	3.89179400	0.02770100
C	3.92564500	5.70276700	-0.01092300
C	-7.58872500	-5.49108500	0.00140200
C	-5.89780700	-3.69700400	-0.02439600
C	-4.20575600	-1.90209100	-0.00273200
C	-2.51351700	-0.10641400	0.02393300
C	-0.82215100	1.69054400	0.04346100
C	0.86794300	3.48783800	0.05268500
C	2.56099800	5.29114700	0.04498200
C	-9.99062100	-4.92567700	0.07533100
C	-8.29804900	-3.13185600	0.04990400
C	-6.60677100	-1.33722700	0.04442000
C	-4.91510800	0.45883500	0.05185100
C	-3.22335800	2.25460300	0.07675000
C	-1.53219000	4.05282400	0.11558400
C	0.15752900	5.85708500	0.10784800
C	-12.39440400	-4.36070600	0.13997400
C	-10.69929500	-2.56743000	0.12717500
C	-9.00681400	-0.77216700	0.11820700
C	-7.31537300	1.02423000	0.09709800
C	-5.62414200	2.82039400	0.08565300
C	-3.93210600	4.61746400	0.14870400
C	-2.24593900	6.42302300	0.17071300
C	-14.79969700	-3.80073900	0.20187200
C	-13.10160700	-2.00626400	0.19880400
C	-11.40789400	-0.20885000	0.19471800
C	-9.71550200	1.58873700	0.18760400
C	-8.02277200	3.38671900	0.17811900
C	-6.32992200	5.18468400	0.19621800
C	-4.64941100	6.98895900	0.23357900
C	-17.20890100	-3.23800500	0.31468000
C	-15.51623300	-1.43918900	0.31164100
C	-13.82356400	0.35963400	0.30860100
C	-12.13089400	2.15845500	0.30556200
C	-10.43822400	3.95727600	0.30252300
C	-8.74555300	5.75609700	0.29948400

C	-7.05288400	7.55491300	0.29644400
C	-8.62942800	-4.52173900	0.02719500
C	-6.93714800	-2.72386900	0.00840100
C	-5.24532800	-0.92817900	0.02241900
C	-3.55421000	0.86714600	0.04818400
C	-1.86361500	2.66247100	0.07153600
C	-0.17517200	4.45729600	0.08923900
C	1.52217600	6.26870500	0.05194300
C	-11.03101400	-3.95591800	0.10148900
C	-9.33776400	-2.15838900	0.08754600
C	-7.64666900	-0.36261800	0.07785200
C	-5.95620100	1.43341400	0.05539100
C	-4.26572500	3.22829300	0.07858300
C	-2.57824100	5.02321600	0.14492200
C	-0.88129200	6.83464300	0.11480900
C	-13.43472900	-3.39018300	0.16623600
C	-11.74170300	-1.59271800	0.16075100
C	-10.05039600	0.20402700	0.15450100
C	-8.35983000	2.00015400	0.13921500
C	-6.66925100	3.79535500	0.13593000
C	-4.98214000	5.58947200	0.19016500
C	-3.28476200	7.40058100	0.17767300
C	-15.84424700	-2.82643100	0.25877400
C	-14.15157700	-1.02760400	0.25573500
C	-12.45890600	0.77121600	0.25269500
C	-10.76623600	2.57003700	0.24965700
C	-9.07356500	4.36885900	0.24661700
C	-7.38089100	6.16767900	0.24357700
C	-5.68822600	7.96651500	0.24053900
H	7.28998300	-11.02347600	-0.39775100
H	4.87964800	-11.74977800	-0.32536500
H	2.48208100	-11.18528200	-0.25485900
H	0.07811300	-10.61911500	-0.19262800
H	-2.32547700	-10.05311200	-0.12950800
H	-4.72911500	-9.48716400	-0.06605700
H	8.97832500	-9.22888600	-0.40866700
H	10.67139200	-7.42957400	-0.41135200
H	12.36435400	-5.63031100	-0.41448500
H	14.05683500	-3.83158900	-0.41668300
H	15.74872900	-2.03370500	-0.42089100
H	17.43893700	-0.23803000	-0.42162900
H	18.01919500	2.20825400	-0.35480000
H	13.78671900	4.49435100	-0.25802200
H	11.38335700	5.05986200	-0.19207800
H	8.98091900	5.62541700	-0.12864400
H	6.57796200	6.19133200	-0.06663500
H	-7.13314100	-8.92107200	-0.00252100
H	-9.53698800	-8.35484000	0.05989000
H	-11.93979500	-7.78885900	0.12134700
H	-14.34190000	-7.22336600	0.18507000
H	-16.73932400	-6.65911600	0.24293700
H	-18.57496700	-4.93680500	0.28232200
H	4.17423100	6.75745600	-0.00440100
H	-17.99306600	-2.49068200	0.34510900
H	-16.30446300	-0.69647100	0.35331700
H	-14.61149800	1.10237300	0.34701600
H	-12.91924300	2.90069100	0.34357900
H	-11.22658800	4.69964100	0.34074200
H	-9.53350700	6.49904700	0.33829800
H	-7.84503800	8.29379000	0.32740800
H	1.77028900	7.32352800	0.05861100
H	-0.63347300	7.88952000	0.12198300
H	-3.03733200	8.45560600	0.18444900
H	-5.43479100	9.02006400	0.25498900
H	16.18709800	3.92908100	-0.31428700

C	-7.11893700	-4.22775600	3.31626300
C	-5.88440000	-3.61773800	3.15384100
C	-5.76954500	-2.24791500	3.28823600
C	-6.89550300	-1.49440900	3.61817000
C	-8.14306600	-2.11162000	3.73039500
C	-8.25739000	-3.47414900	3.57319500
O	-6.90754700	-0.14725200	3.81899700
C	-5.75384800	0.61216000	3.88030200
C	-4.62936100	0.25345600	4.61152600
C	-3.51158600	1.07060900	4.59134400
C	-3.48883700	2.24909900	3.83700200
C	-4.67920300	2.64423400	3.22021400
C	-5.79964800	1.84019800	3.24160600
C	-2.27059600	3.05493800	3.68114800
C	-0.89526700	2.57788100	3.53643700
C	0.17522800	3.47837500	3.43072100
C	1.46026700	3.00671300	3.25043600
C	1.73421400	1.65064500	3.21184400
C	0.69413800	0.72829300	3.36426000
C	-0.61268100	1.21283300	3.47788200
C	0.95516300	-0.71289200	3.38583100
C	2.25576000	-1.37941200	3.51121400
C	3.30911600	-0.81073000	4.23526200
C	4.56542400	-1.39307600	4.25515000
C	4.78922800	-2.56543600	3.54550300
C	3.72839300	-3.21081900	2.92982100
C	2.48081500	-2.62363700	2.91440600
O	6.00601600	-3.21783000	3.48041200
C	7.19879600	-2.58028000	3.32753800
C	7.36132900	-1.22291200	3.05141400
C	8.63411000	-0.69680100	2.94976700
C	9.73822500	-1.52305100	3.09210200
C	9.58356300	-2.88739700	3.30247400
C	8.31700900	-3.41147200	3.42671000
N	-2.45746500	4.34311300	3.57626600
N	-0.08267600	-1.49656100	3.28248800
N	-2.61312200	5.47008000	3.49405600
N	-1.00662600	-2.16232500	3.20068400
H	-5.02093900	-4.21865500	2.91824700
H	-4.81388700	-1.77864500	3.12542800
H	-9.00811500	-1.50143900	3.94412200
H	-9.21291500	-3.96616500	3.66028600
H	-4.62234000	-0.65183100	5.19961200
H	-2.65490100	0.79200500	5.18511200
H	-4.72287300	3.57514400	2.67649900
H	-6.70417000	2.13921100	2.73558300
H	0.00515300	4.54512600	3.45806800
H	2.26992600	3.71106800	3.11932400
H	2.74287300	1.31841400	3.03108300
H	-1.42886000	0.51115900	3.50641600
H	3.14585300	0.09094300	4.80481600
H	5.35545000	-0.93984600	4.83390300
H	3.89946700	-4.15547900	2.43761800
H	1.68068300	-3.12453700	2.39156600
H	6.50908500	-0.58370700	2.89543200
H	8.77981700	0.35210000	2.74777300
H	10.45573900	-3.51704800	3.37616900
H	8.16669800	-4.46663700	3.60068300
N	-7.22442000	-5.68022100	3.22741000
O	-8.34628200	-6.18291100	3.31042200
O	-6.18487100	-6.32553700	3.08442700
N	11.07961300	-0.95418300	3.01366600
O	11.18108800	0.26944600	2.91436400
O	12.03710900	-1.72803700	3.05329600

(4) Bisdiazo-NH_2/Graphene

C	6.44518200	-9.16715300	-0.01757900
C	5.06531100	-9.52723900	0.01603400
C	2.68371000	-8.87237100	0.01571000
C	0.30210500	-8.21750100	0.01538300
C	-2.07950100	-7.56263000	0.01505800
C	-4.46110600	-6.90775900	0.01473300
C	4.06357300	-8.51226600	-0.01790500
C	1.68196400	-7.85739800	-0.01822900
C	-0.69964100	-7.20252600	-0.01855500
C	-3.08124600	-6.54765600	-0.01888000
C	-5.46285100	-5.89278500	-0.01920500
C	8.20311700	-7.43206200	-0.01689300
C	9.96105500	-5.69696700	-0.01620500
C	11.71899300	-3.96187200	-0.01551700
C	13.47693000	-2.22677700	-0.01483400
C	15.23486800	-0.49168200	-0.01413600
C	16.99280100	1.24341300	-0.01353000
C	6.82324500	-7.79212800	0.01672200
C	8.58118500	-6.05703300	0.01740800
C	10.33912200	-4.32193800	0.01809600
C	12.09706000	-2.58684200	0.01878200
C	13.85499700	-0.85174700	0.01947300
C	15.61293100	0.88335500	0.02014000
C	17.37087600	2.61842800	0.02087000
C	4.44713500	-7.12546500	-0.01093800
C	6.19884300	-5.39322900	-0.00204100
C	7.95364000	-3.66155500	0.01633900
C	9.70910000	-1.92962500	0.05309300
C	11.46538500	-0.19677000	0.06318700
C	13.22445200	1.53663000	0.05779700
C	14.98927200	3.27329700	0.02054300
C	2.06585900	-6.47031900	-0.02922200
C	3.81771500	-4.73749900	-0.01567800
C	5.57232100	-3.00574700	-0.00354600
C	7.32767200	-1.27477400	0.02996100
C	9.08358100	0.45749500	0.04936500
C	10.84221600	2.19047400	0.05425100
C	12.60766500	3.92816600	0.02018400
C	-0.31507700	-5.81602000	-0.04235800
C	1.43758900	-4.08381000	-0.03547200
C	3.19179800	-2.35227100	-0.00379400
C	4.94789500	-0.62038800	0.02393500
C	6.70470900	1.11120600	0.02237500
C	8.46277700	2.84403800	0.03049400
C	10.22606000	4.58303700	0.01987000
C	-2.69591400	-5.16164900	-0.04454000
C	-0.94053400	-3.43097200	-0.07191000
C	0.81290400	-1.69922700	-0.04476800
C	2.56786300	0.03287700	-0.00488300
C	4.32442000	1.76515000	0.02960600
C	6.08236500	3.49918400	0.04255000
C	7.84445500	5.23790700	0.01954400
C	5.80924600	-6.77010900	-0.00161800
C	7.56676900	-5.03529500	0.00375300
C	9.32421400	-3.30137500	0.02553000
C	11.08186700	-1.56781400	0.04300900
C	12.84080100	0.16569200	0.04320100
C	14.60421400	1.89630300	0.03844700
C	16.36912800	3.63340400	-0.01305000
C	3.43416400	-6.11519400	-0.03177400
C	5.19025600	-4.37929700	-0.02887100
C	6.94624800	-2.64710300	-0.00070000
C	8.70282500	-0.91558300	0.03823700
C	10.45985300	0.81543000	0.05009500

C	12.22051400	2.54511700	0.04235400
C	13.98752500	4.28826600	-0.01348500
C	1.05650600	-5.46193800	-0.05118200
C	2.81129500	-3.72623400	-0.02675500
C	4.56758200	-1.99433200	0.00266100
C	6.32410200	-0.26227800	0.01401700
C	8.08081900	1.46803600	0.01910800
C	9.83896300	3.19801000	0.02441100
C	11.60592100	4.94314100	-0.01374400
C	-1.32193700	-4.80837300	-0.07015400
C	0.43369800	-3.07366400	-0.06404600
C	2.18822000	-1.34088200	-0.02852400
C	3.94391300	0.39064600	0.00995800
C	5.70116600	2.12125900	0.02079200
C	7.45876800	3.85177600	0.02406300
C	9.22431600	5.59801300	-0.01407100
C	-3.70201200	-4.15468500	-0.06433600
C	-1.94416500	-2.42069800	-0.08709600
C	-0.18951500	-0.68811700	-0.07195500
C	1.56519000	1.04428900	-0.03580600
C	3.32119000	2.77587900	0.01792800
C	5.07762700	4.50679800	0.03269900
C	6.84271000	6.25288300	-0.01439500
C	-6.84271100	-6.25288800	0.01440800
C	-7.84445700	-5.23791400	-0.01953100
C	-9.22431700	-5.59801800	0.01408300
C	-11.60592300	-4.94314800	0.01375700
C	-13.98752800	-4.28827700	0.01343300
C	-16.36912600	-3.63340300	0.01310700
C	-10.22606200	-4.58304300	-0.01985600
C	-12.60766700	-3.92817200	-0.02018100
C	-14.98927400	-3.27329800	-0.02050600
C	-17.37088300	-2.61844300	-0.02083200
C	-5.07743400	-4.50721700	-0.04323900
C	-3.32000700	-2.77673000	-0.07055700
C	-1.56465000	-1.04518800	-0.07383300
C	0.18947900	0.68679500	-0.06423200
C	1.94531800	2.41886400	-0.00181900
C	3.70223000	4.15366600	0.03920400
C	5.46285000	5.89277900	0.01921900
C	-6.08207000	-3.50015000	-0.07009500
C	-4.32373400	-1.76570500	-0.09299200
C	-2.56772500	-0.03323900	-0.09995700
C	-0.81328700	1.69779100	-0.10293800
C	0.94152800	3.42993400	-0.02295000
C	2.69672700	5.16131700	0.02324500
C	4.46110500	6.90775300	-0.01472000
C	-7.45825100	-3.85292700	-0.04849200
C	-5.70039000	-2.12196800	-0.08413500
C	-3.94393200	-0.39030700	-0.09867400
C	-2.18850500	1.34051500	-0.10636500
C	-0.43333800	3.07258900	-0.06028200
C	1.32264500	4.80793700	0.01963500
C	3.08124500	6.54765000	0.01889300
C	-9.83923600	-3.19872200	-0.04712200
C	-8.07975800	-1.46799600	-0.09178400
C	-6.32317300	0.26353800	-0.12969900
C	-4.56721100	1.99414300	-0.10454500
C	-2.81225300	3.72587100	-0.06790700
C	-1.05652900	5.46119500	0.00351800
C	0.69963900	7.20252000	0.01856800
C	-12.22126900	-2.54519400	-0.04100900
C	-10.46047600	-0.81511000	-0.07066800
C	-8.70163400	0.91744000	-0.10882700
C	-6.94609500	2.64779500	-0.09004200

C	-5.19049400	4.37916900	-0.04710600
C	-3.43437000	6.11438000	0.00339000
C	-1.68196600	7.85739100	0.01824300
C	-14.60458000	-1.89635500	-0.03656000
C	-12.84174100	-0.16582100	-0.04256200
C	-11.08182100	1.56837100	-0.05390700
C	-9.32386800	3.30188600	-0.05256600
C	-7.56688000	5.03522600	-0.03755800
C	-5.80950100	6.76995400	-0.01256900
C	-4.06357400	8.51226000	0.01791800
C	-16.99280100	-1.24341700	0.01346900
C	-15.23486700	0.49167300	0.01415700
C	-13.47693100	2.22677100	0.01484300
C	-11.71899400	3.96186600	0.01553100
C	-9.96105700	5.69696100	0.01621800
C	-8.20311900	7.43205600	0.01690600
C	-6.44518300	9.16714600	0.01759200
C	-8.46218700	-2.84547600	-0.07315900
C	-6.70323100	-1.11057800	-0.11941100
C	-4.94775600	0.62071900	-0.13117800
C	-3.19234800	2.35128800	-0.11153700
C	-1.43793400	4.08396600	-0.05703300
C	0.31554400	5.81571100	0.01110000
C	2.07949900	7.56262400	-0.01504500
C	-10.84316300	-2.19129500	-0.06510600
C	-9.08328400	-0.45616900	-0.10756000
C	-7.32698000	1.27553500	-0.13052500
C	-5.57200800	3.00574900	-0.09195300
C	-3.81783200	4.73726300	-0.04844000
C	-2.06618100	6.47014300	0.00805500
C	-0.30210600	8.21749500	-0.01537000
C	-13.22501500	-1.53684200	-0.05332300
C	-11.46703200	0.19744700	-0.07125300
C	-9.70883300	1.93082300	-0.08929100
C	-7.95314700	3.66219900	-0.07212600
C	-6.19892300	5.39314700	-0.04391600
C	-4.44772700	7.12515400	0.00052300
C	-2.68371200	8.87236500	-0.01569600
C	-15.61293600	-0.88336000	-0.02014400
C	-13.85499900	0.85174200	-0.01945700
C	-12.09706100	2.58683600	-0.01877000
C	-10.33912400	4.32193100	-0.01808200
C	-8.58118700	6.05702700	-0.01739500
C	-6.82324600	7.79212100	-0.01670900
C	-5.06531300	9.52723200	-0.01602100
H	7.20990900	-9.93504000	-0.01741300
H	4.77329000	-10.57086100	0.01559700
H	2.39736500	-9.91749900	0.02306600
H	0.01528300	-9.26239400	0.02209100
H	-2.36644700	-8.60744900	0.02201700
H	-4.74823300	-7.95255100	0.02227300
H	8.96353200	-8.20416600	-0.02461600
H	10.72187600	-6.46858700	-0.02357500
H	12.48012300	-4.73306300	-0.02298300
H	14.23784900	-2.99804400	-0.02145700
H	15.99500100	-1.26387900	-0.02193800
H	17.75031300	0.46820800	-0.01894600
H	18.41939700	2.89238400	0.01519500
H	14.27356700	5.33371100	-0.02321800
H	11.89198700	5.98821100	-0.02045500
H	9.51138600	6.64267200	-0.02018200
H	7.13030900	7.29748400	-0.02135200
H	-7.13040400	-7.29749300	0.02260300
H	-9.51236700	-6.64232400	0.02181000
H	-11.89327900	-5.98746200	0.02009100

H	-14.27355700	-5.33306900	0.02066100
H	-16.64908000	-4.68020300	0.01550400
H	-18.41997000	-2.89124800	-0.01729900
H	4.74846200	7.95253800	-0.02231700
H	-17.74916400	-0.46737100	0.01284000
H	-15.99570000	1.26348500	0.02476700
H	-14.23739900	2.99852900	0.02220000
H	-12.47988700	4.73312900	0.02249000
H	-10.72196400	6.46835800	0.02337600
H	-8.96360800	8.20403400	0.02465700
H	-7.20985300	9.93505700	0.01748000
H	2.36638500	8.60756300	-0.02250900
H	-0.01552200	9.26250800	-0.02233500
H	-2.39750300	9.91753300	-0.02307100
H	-4.77332200	10.57085300	-0.01556500
H	16.65197100	4.67974900	-0.01637500
C	8.81496400	2.35388700	3.32621900
C	7.52012300	1.86145800	3.16858600
C	7.23979200	0.50960000	3.27948000
C	8.25681700	-0.38414300	3.58495100
C	9.55514200	0.08804400	3.73308800
C	9.83181400	1.43442400	3.59798100
O	8.11806300	-1.75794400	3.67879300
C	6.90294500	-2.38046600	3.78190500
C	5.81731600	-1.89695300	4.50519900
C	4.61798700	-2.59060700	4.49524300
C	4.46231000	-3.76544200	3.75164400
C	5.60603500	-4.29735300	3.14807500
C	6.80805300	-3.62417100	3.16965300
C	3.16269200	-4.43276600	3.58558500
C	1.84338300	-3.81538000	3.44987600
C	0.68775500	-4.60370000	3.32231400
C	-0.54271200	-4.00371800	3.15403600
C	-0.68103100	-2.62562400	3.14503100
C	0.44350400	-1.81486200	3.32096200
C	1.69717600	-2.42940800	3.42341200
C	0.32661000	-0.35682400	3.38796400
C	-0.90203600	0.43278800	3.53633200
C	-2.02634400	-0.05920700	4.20828400
C	-3.21978700	0.64277500	4.23124400
C	-3.31495500	1.87199500	3.58558800
C	-2.16600800	2.43933600	3.04639100
C	-0.98504100	1.72943100	3.01909100
O	-4.44967200	2.63203900	3.49356700
C	-5.72502700	2.09969500	3.42518500
C	-6.03583800	0.78900200	3.09077000
C	-7.36001600	0.39307500	3.00586400
C	-8.40581800	1.29238600	3.20964400
C	-8.07436800	2.61624800	3.51143500
C	-6.75548500	3.01056600	3.62616900
N	3.21259700	-5.72971900	3.44473300
N	1.43756400	0.32303200	3.31574300
N	3.26190200	-6.86300500	3.31548500
N	2.41380000	0.91383300	3.25428100
H	6.71619900	2.54333500	2.92358300
H	6.23411500	0.16688500	3.09667900
H	10.34802700	-0.61787700	3.93651900
H	10.85255900	1.77990200	3.70369100
H	5.90403900	-0.98632600	5.07756500
H	3.80020500	-2.21451500	5.09041100
H	5.54806600	-5.23429000	2.61560100
H	7.67474400	-4.02837600	2.67103100
H	0.75146600	-5.68237100	3.33199200
H	-1.41814800	-4.62284900	3.01337000
H	-1.65079400	-2.19098200	2.97101000

H	2.57779600	-1.81290600	3.47377300
H	-1.97023300	-0.99940000	4.73451000
H	-4.06669900	0.23576600	4.76099800
H	-2.22327300	3.42464300	2.61077100
H	-0.12303400	2.17973500	2.55021400
H	-5.26123200	0.07605500	2.86044100
H	-7.58349000	-0.63109000	2.73770900
H	-8.86077500	3.34642600	3.65487500
H	-6.50689700	4.03806100	3.85161500
N	9.07893300	3.72077400	3.26647400
N	-9.73325800	0.87351700	3.16536800
H	-9.88674700	0.06606900	2.58221600
H	-10.38381400	1.60101500	2.91583000
H	10.01597500	3.94074500	2.96907300
H	8.41782100	4.23863500	2.70924500

2.7.3 Optimizations of DiazoCarbene onto Graphene via cluster model

(1) DiazoCarbene-H/Graphene

C	6.65373800	-9.50536300	0.72194000
C	5.27386700	-9.86544900	0.75555300
C	2.89226600	-9.21058100	0.75522900
C	0.51066100	-8.55571100	0.75490200
C	-1.87094500	-7.90084000	0.75457600
C	-4.25255000	-7.24596900	0.75425100
C	4.27212900	-8.85047600	0.72161300
C	1.89052000	-8.19560800	0.72128900
C	-0.49108500	-7.54073600	0.72096400
C	-2.87269000	-6.88586600	0.72063900
C	-5.25429500	-6.23099500	0.72031400
C	8.41167300	-7.77027200	0.72262600
C	10.16961100	-6.03517700	0.72331400
C	11.92754900	-4.30008200	0.72400200
C	13.68548600	-2.56498700	0.72468400
C	15.44342400	-0.82989200	0.72538200
C	17.20135700	0.90520300	0.72598900
C	7.03180100	-8.13033800	0.75624000
C	8.78974100	-6.39524300	0.75692600
C	10.54767800	-4.66014800	0.75761400
C	12.30561600	-2.92505200	0.75830100
C	14.06355300	-1.18995700	0.75899200
C	15.82148700	0.54514500	0.75965900
C	17.57943200	2.28021800	0.76038900
C	4.65603600	-7.46339300	0.72816300
C	6.40662700	-5.73157400	0.74540000
C	8.16121800	-3.99993000	0.75051200
C	9.91768100	-2.26776700	0.78211300
C	11.67443300	-0.53516200	0.80146700
C	13.43294400	1.19870600	0.79698200
C	15.19782800	2.93508700	0.76006200
C	2.27488500	-6.80852900	0.71328100
C	4.02596400	-5.07564700	0.71986900
C	5.78034100	-3.34360900	0.71116600
C	7.53624600	-1.61248000	0.74629300
C	9.29252900	0.12035100	0.79624100
C	11.05128700	1.85363300	0.80392000
C	12.81622100	3.58995600	0.75970300
C	-0.10619300	-6.15411300	0.70753100
C	1.64737300	-4.42166500	0.70386500
C	3.40101300	-2.69010300	0.72031100
C	5.15695200	-0.95882800	0.75137700
C	6.91373000	0.77434700	0.77750700
C	8.67155800	2.50822600	0.78369800
C	10.43461600	4.24482700	0.75938900

C	-2.48773700	-5.49977300	0.70435400
C	-0.73197600	-3.76830100	0.68779200
C	1.02284200	-2.03688500	0.69365800
C	2.77735100	-0.30515200	0.73339200
C	4.53367400	1.42813800	0.76761700
C	6.29179400	3.16222400	0.77225000
C	8.05301100	4.89969700	0.75906300
C	6.01771100	-7.10866800	0.74044700
C	7.77483800	-5.37404100	0.75024200
C	9.53276200	-3.63960500	0.76179000
C	11.29113700	-1.90592200	0.77858800
C	13.05005200	-0.17247400	0.78257200
C	14.81274700	1.55813400	0.77781200
C	16.57768400	3.29519400	0.72646900
C	3.64297100	-6.45294400	0.70742300
C	5.39810400	-4.71823500	0.70778300
C	7.15474400	-2.98539800	0.71034200
C	8.91168900	-1.25312100	0.76532000
C	10.66918100	0.47827100	0.79666000
C	12.42910700	2.20724200	0.78354400
C	14.19608100	3.95005600	0.72603400
C	1.26573000	-5.79967600	0.69533600
C	3.02068100	-4.06436600	0.70569400
C	4.77652000	-2.33182400	0.71476500
C	6.53293700	-0.59992600	0.75061500
C	8.28991600	1.13185200	0.77703400
C	10.04790200	2.86102500	0.77938700
C	11.81447700	4.60493100	0.72577500
C	-1.11334500	-5.14613400	0.68797700
C	0.64329900	-3.41115800	0.67939100
C	2.39806400	-1.67954500	0.70452000
C	4.15347800	0.05314100	0.74918800
C	5.91052600	1.78527200	0.76207100
C	7.66819900	3.51502800	0.76073500
C	9.43287200	5.25980300	0.72544800
C	-3.49347000	-4.49266000	0.68603200
C	-1.73579900	-2.75774500	0.67458200
C	0.02011100	-1.02578500	0.67877400
C	1.77468300	0.70634700	0.70584800
C	3.53031000	2.43859900	0.74626200
C	5.28682400	4.16897300	0.76428900
C	7.05126600	5.91467300	0.72512400
C	-6.63415500	-6.59109800	0.75392600
C	-7.63590100	-5.57612400	0.71998800
C	-9.01576100	-5.93622800	0.75360100
C	-11.39736700	-5.28135800	0.75327600
C	-13.77897200	-4.62648700	0.75295200
C	-16.16057000	-3.97161300	0.75262600
C	-10.01750600	-4.92125300	0.71966300
C	-12.39911100	-4.26638200	0.71933800
C	-14.78071800	-3.61150800	0.71901300
C	-17.16232700	-2.95665300	0.71868700
C	-4.86939300	-4.84551500	0.70164000
C	-3.11226800	-3.11466400	0.68975600
C	-1.35601800	-1.38276300	0.68343200
C	0.39878800	0.34876000	0.69470600
C	2.15439000	2.08125400	0.72797500
C	3.91100000	3.81582500	0.76957200
C	5.67140600	5.55456900	0.75873800
C	-5.87387400	-3.83830600	0.68062800
C	-4.11614700	-2.10387200	0.67237100
C	-2.35946400	-0.37151000	0.67284900
C	-0.60414000	1.36006400	0.66892100
C	1.15083600	3.09167100	0.70005200
C	2.90582400	4.82310700	0.75921400

C	4.66966100	6.56954300	0.72479900
C	-7.25060700	-4.19114400	0.69817100
C	-5.49263200	-2.46045300	0.67949200
C	-3.73599600	-0.72859400	0.67862600
C	-1.97994500	1.00321400	0.67821500
C	-0.22477900	2.73495200	0.67242600
C	1.53144300	4.46929600	0.74770500
C	3.28980100	6.20944000	0.75841200
C	-9.63138100	-3.53685700	0.69575300
C	-7.87240500	-1.80621300	0.66956800
C	-6.11592600	-0.07397100	0.65084700
C	-4.35960700	1.65712600	0.66428700
C	-2.60331800	3.38863400	0.66827700
C	-0.84805100	5.12235000	0.72396900
C	0.90819500	6.86431000	0.75808700
C	-12.01251400	-2.88337800	0.69927300
C	-10.25254600	-1.15328300	0.67688100
C	-8.49458900	0.57942500	0.64838100
C	-6.73872800	2.31108200	0.65000500
C	-4.98244800	4.04197400	0.69118000
C	-3.22616200	5.77624200	0.73447000
C	-1.47341000	7.51918100	0.75776200
C	-14.39564700	-2.23451700	0.70291300
C	-12.63309300	-0.50362700	0.69858600
C	-10.87362800	1.23052600	0.69151700
C	-9.11565500	2.96446800	0.68641200
C	-7.35846400	4.69757600	0.69887900
C	-5.60110100	6.43183200	0.72570100
C	-3.85501800	8.17405000	0.75743700
C	-16.78424500	-1.58162700	0.75298800
C	-15.02631100	0.15346300	0.75367600
C	-13.26837500	1.88856100	0.75436200
C	-11.51043800	3.62365600	0.75505000
C	-9.75250100	5.35875100	0.75573700
C	-7.99456300	7.09384600	0.75642500
C	-6.23662700	8.82893600	0.75711100
C	-8.25442600	-3.18362000	0.67518300
C	-6.49587800	-1.44897000	0.65452400
C	-4.73959100	0.28323000	0.65323000
C	-2.98371600	2.01433200	0.65973600
C	-1.22907900	3.74599100	0.65951000
C	0.52422500	5.47723800	0.74422100
C	2.28805500	7.22441400	0.72447400
C	-10.63489300	-2.52907300	0.67714700
C	-8.87572900	-0.79430400	0.65254500
C	-7.11937300	0.93849300	0.62671600
C	-5.36446000	2.66877300	0.65414000
C	-3.60952300	4.39941100	0.68347600
C	-1.85777600	6.13177800	0.74008700
C	-0.09355000	7.87928500	0.72414900
C	-13.01599200	-1.87487000	0.68625700
C	-11.25770800	-0.14036300	0.67445700
C	-9.50081100	1.59343300	0.65918000
C	-7.74518400	3.32493800	0.66411700
C	-5.99085300	5.05546900	0.69334000
C	-4.23953500	6.78708600	0.73889400
C	-2.47515600	8.53415500	0.72382300
C	-15.40438000	-1.22157000	0.71937500
C	-13.64644300	0.51353200	0.72006200
C	-11.88850500	2.24862600	0.72074900
C	-10.13056800	3.98372100	0.72143700
C	-8.37263100	5.71881700	0.72212400
C	-6.61469000	7.45391100	0.72281000
C	-4.85675700	9.18902200	0.72349800
H	7.41846500	-10.27325000	0.72210500

H	4.98184600	-10.90907100	0.75511600
H	2.60592100	-10.25570900	0.76258400
H	0.22383900	-9.60060400	0.76161000
H	-2.15789100	-8.94565900	0.76153600
H	-4.53967700	-8.29076100	0.76179200
H	9.17208800	-8.54237600	0.71490200
H	10.93043200	-6.80679700	0.71594300
H	12.68867900	-5.07127300	0.71653600
H	14.44640500	-3.33625400	0.71806200
H	16.20355700	-1.60208900	0.71758100
H	17.95886900	0.12999800	0.72057300
H	18.62795300	2.55417400	0.75471400
H	14.48212300	4.99550100	0.71630100
H	12.10054300	5.65000100	0.71906400
H	9.71994200	6.30446200	0.71933700
H	7.33886500	6.95927400	0.71816700
H	-6.92184800	-7.63570300	0.76212200
H	-9.30381100	-6.98053400	0.76132900
H	-11.68472300	-6.32567200	0.75961000
H	-14.06500100	-5.67127900	0.76018000
H	-16.44052400	-5.01841300	0.75502300
H	-18.21141400	-3.22945800	0.72222000
H	4.95701800	7.61432800	0.71720200
H	-17.54060800	-0.80558100	0.75235900
H	-15.78714400	0.92527500	0.76428600
H	-14.02884300	2.66031900	0.76171900
H	-12.27133100	4.39491900	0.76200900
H	-10.51340800	6.13014800	0.76289500
H	-8.75505200	7.86582400	0.76417600
H	-7.00129700	9.59684700	0.75699900
H	2.57494100	8.26935300	0.71701000
H	0.19303400	8.92429800	0.71718400
H	-2.18894700	9.57932300	0.71644800
H	-4.56476600	10.23264300	0.72395400
H	16.86052700	4.34153900	0.72314400
C	8.72697700	3.80421000	4.08697600
C	7.45858800	3.26690900	3.93444200
C	7.24812100	1.89901600	4.02520000
C	8.32694900	1.06869900	4.30012300
C	9.60549100	1.59290800	4.43988200
C	9.80124400	2.95838300	4.32493400
O	8.25783600	-0.31132600	4.35686500
C	7.09469800	-0.99876700	4.50067500
C	6.01263100	-0.57004000	5.28374500
C	4.84101700	-1.28705100	5.26791000
C	4.70270300	-2.46279300	4.47792200
C	5.89290800	-2.97369400	3.89973200
C	7.05643500	-2.24593800	3.88164600
C	3.52087200	-3.14276900	4.12213200
C	2.25300000	-2.48796900	4.08367700
C	1.10754100	-3.31161000	4.01721300
C	-0.15085500	-2.75326700	3.93558300
C	-0.31475000	-1.37704900	3.92280900
C	0.78815800	-0.51439100	4.04121200
C	2.05724500	-1.08976500	4.05369300
C	0.60241700	0.93480500	4.13901900
C	-0.66478600	1.65198200	4.33423900
C	-1.71329300	1.10528200	5.08199800
C	-2.95104700	1.72224900	5.14235800
C	-3.16347600	2.91258700	4.45546200
C	-2.09388400	3.53883100	3.83135800
C	-0.86458300	2.91573300	3.77330900
O	-4.36160200	3.58306200	4.41536000
C	-5.57349800	2.94866900	4.25001900
C	-5.74006800	1.60935200	3.91793500

C	-7.02341600	1.10115800	3.77286900
C	-8.13543300	1.91320600	3.92327100
C	-7.95535400	3.25897900	4.21303900
C	-6.68377800	3.77683800	4.38312100
N	1.67696100	1.66935700	4.06223800
N	2.62944300	2.29671400	4.00346400
H	8.88118200	4.86900900	3.98665500
H	6.61902300	3.91140100	3.71481000
H	6.26468900	1.49565300	3.84660500
H	10.43168800	0.91916900	4.61654300
H	10.80139900	3.35985200	4.41461200
H	6.10001600	0.32903700	5.87523800
H	4.01198000	-0.95571400	5.87623300
H	5.83054200	-3.92891000	3.40351400
H	7.94033000	-2.60345600	3.37696900
H	1.25650200	-4.38147600	4.00322700
H	-1.02121900	-3.38638700	3.83028600
H	-1.30215200	-0.96822300	3.78038300
H	2.92945300	-0.45609800	4.01302200
H	-1.56306200	0.18970500	5.63360500
H	-3.74340400	1.27991700	5.72633800
H	-2.24955200	4.49685900	3.35971300
H	-0.06125000	3.40274300	3.24211800
H	-4.88996400	0.96969100	3.74698700
H	-7.14656000	0.05768000	3.51775000
H	-9.12869800	1.50932800	3.78810800
H	-8.81073900	3.91508500	4.30214400
H	-6.52892900	4.82269900	4.60736200

(2) DiazoCarbene-Me/Graphene

C	6.12240000	-10.15026000	0.41537400
C	4.74252900	-10.51034600	0.44898700
C	2.36092800	-9.85547800	0.44866300
C	-0.02067700	-9.20060800	0.44833600
C	-2.40228300	-8.54573700	0.44801100
C	-4.78388800	-7.89086600	0.44768600
C	3.74079100	-9.49537300	0.41504800
C	1.35918200	-8.84050500	0.41472400
C	-1.02242300	-8.18563300	0.41439800
C	-3.40402800	-7.53076300	0.41407300
C	-5.78563300	-6.87589200	0.41374800
C	7.88033500	-8.41516900	0.41606000
C	9.63827300	-6.68007400	0.41674800
C	11.39621100	-4.94497900	0.41743600
C	13.15414800	-3.20988400	0.41811900
C	14.91208600	-1.47478900	0.41881700
C	16.67001900	0.26030600	0.41942300
C	6.50046300	-8.77523500	0.44967500
C	8.25840300	-7.04014000	0.45036100
C	10.01634000	-5.30504500	0.45104900
C	11.77427800	-3.56994900	0.45173500
C	13.53221500	-1.83485400	0.45242600
C	15.29014900	-0.09975200	0.45309300
C	17.04809400	1.63532100	0.45382300
C	4.12338500	-8.10899800	0.42187500
C	5.87500800	-6.37740200	0.45521000
C	7.63036800	-4.64626900	0.45599700
C	9.38697300	-2.91402300	0.43873200
C	11.14308600	-1.18047300	0.46607100
C	12.90152300	0.55243300	0.47208300
C	14.66649000	2.29019000	0.45349600
C	1.74138400	-7.45494100	0.39119600
C	3.49475700	-5.72166400	0.39681600
C	5.25013400	-3.98967000	0.42247600
C	7.00446200	-2.25858600	0.42640100

C	8.76057500	-0.52697700	0.46217100
C	10.52146000	1.20762200	0.47436800
C	12.28488300	2.94505900	0.45313700
C	-0.63963400	-6.80088200	0.38108100
C	1.11562300	-5.06768800	0.33883700
C	2.86972200	-3.33470000	0.36525400
C	4.62495600	-1.60458000	0.41173400
C	6.38168900	0.12723700	0.45698400
C	8.13997500	1.86367300	0.48394400
C	9.90327800	3.59993000	0.45282300
C	-3.01993700	-6.14540900	0.38807200
C	-1.26466800	-4.41347600	0.33970200
C	0.49061000	-2.68116200	0.32791100
C	2.24534300	-0.95067800	0.40252400
C	4.00117500	0.78286900	0.44230300
C	5.75828500	2.51694000	0.47324800
C	7.52167300	4.25480000	0.45249700
C	5.48864300	-7.75277000	0.43945700
C	7.24669600	-6.01835500	0.45428200
C	9.00350000	-4.28519100	0.46308800
C	10.76011100	-2.55145400	0.46290000
C	12.52002500	-0.81688800	0.47847800
C	14.28341600	0.91324900	0.47077300
C	16.04634600	2.65029700	0.41990300
C	3.11362500	-7.09862500	0.38990700
C	4.86906200	-5.36388200	0.41588600
C	6.62428200	-3.63168600	0.42716700
C	8.38075600	-1.89956200	0.44707400
C	10.13948000	-0.16767800	0.48380600
C	11.89853400	1.56225900	0.48073000
C	13.66474300	3.30515900	0.41946800
C	0.73447000	-6.44626800	0.35594500
C	2.49021800	-4.70991300	0.34805800
C	4.24578800	-2.97737200	0.38928000
C	6.00242300	-1.24638600	0.41028900
C	7.75823400	0.48565200	0.45824700
C	9.51768800	2.21806700	0.46969900
C	11.28313900	3.96003400	0.41920900
C	-1.64623000	-5.79240400	0.35862200
C	0.11121200	-4.05601100	0.30917300
C	1.86749500	-2.32343500	0.34507100
C	3.62165800	-0.59287600	0.41807900
C	5.37783500	1.13996500	0.46124900
C	7.13764600	2.87148800	0.46603700
C	8.90153400	4.61490600	0.41888200
C	-4.02557900	-5.13675000	0.36854300
C	-2.26777600	-3.40244400	0.34277700
C	-0.51200600	-1.66979400	0.34228900
C	1.24181200	0.06245600	0.40372000
C	2.99796600	1.79386900	0.46331200
C	4.75545600	3.52387400	0.47125400
C	6.51992800	5.26977600	0.41855800
C	-7.16549300	-7.23599500	0.44736100
C	-8.16723900	-6.22102100	0.41342200
C	-9.54709900	-6.58112500	0.44703600
C	-11.92870500	-5.92625500	0.44671000
C	-14.31031000	-5.27138400	0.44638600
C	-16.69190800	-4.61651000	0.44606000
C	-10.54884400	-5.56615000	0.41309700
C	-12.93044900	-4.91127900	0.41277200
C	-15.31205600	-4.25640500	0.41244700
C	-17.69366500	-3.60155000	0.41212100
C	-5.40149300	-5.48996900	0.39019100
C	-3.64534300	-3.75994300	0.37162200
C	-1.88805700	-2.02790100	0.35785200

C	-0.13359000	-0.29474400	0.38562100
C	1.62047600	1.43751400	0.42253600
C	3.37900600	3.17078900	0.45897400
C	5.14006800	4.90967200	0.45217200
C	-6.40460800	-4.48289600	0.38774600
C	-4.64758200	-2.74902900	0.36915500
C	-2.89167400	-1.01538200	0.36664200
C	-1.13539700	0.71716500	0.38688500
C	0.61839300	2.44801900	0.42905300
C	2.37332500	4.17915900	0.46490900
C	4.13832300	5.92464600	0.41823300
C	-7.78384200	-4.83739800	0.37886900
C	-6.02457900	-3.10614900	0.35751300
C	-4.26746400	-1.37369600	0.36827400
C	-2.51232300	0.35837000	0.39352100
C	-0.75736000	2.09094600	0.42569200
C	0.99932700	3.82555800	0.45319400
C	2.75846300	5.56454300	0.45184600
C	-10.16338700	-4.18463700	0.37285300
C	-8.40464300	-2.45215200	0.34794000
C	-6.64861400	-0.72061700	0.35699300
C	-4.89151300	1.01168400	0.38040700
C	-3.13490000	2.74481300	0.41476000
C	-1.38071000	4.47956300	0.46055800
C	0.37685700	6.21941300	0.45152100
C	-12.54364000	-3.52853900	0.38810000
C	-10.78457800	-1.79919100	0.37075600
C	-9.02728800	-0.06805100	0.36280900
C	-7.26979000	1.66557700	0.35524200
C	-5.51344600	3.39856400	0.36486900
C	-3.75870400	5.13258500	0.42960300
C	-2.00474800	6.87428400	0.45119600
C	-14.92879600	-2.87940900	0.39710800
C	-13.16510800	-1.14952700	0.38951800
C	-11.40577500	0.58455500	0.39251000
C	-9.64936900	2.31893300	0.38274100
C	-7.89224200	4.05300600	0.36788300
C	-6.13391200	5.78693400	0.40185300
C	-4.38635600	7.52915300	0.45087100
C	-17.31558300	-2.22652400	0.44642200
C	-15.55764900	-0.49143400	0.44711000
C	-13.79971300	1.24366400	0.44779600
C	-12.04177600	2.97875900	0.44848400
C	-10.28383900	4.71385400	0.44917100
C	-8.52590100	6.44894900	0.44985900
C	-6.76796500	8.18403900	0.45054500
C	-8.78609300	-3.82958400	0.37220300
C	-7.02880500	-2.09483200	0.35773100
C	-5.27099800	-0.36201200	0.38475400
C	-3.51479400	1.37026200	0.40451100
C	-1.76120000	3.10219400	0.42274000
C	-0.00688600	4.83439900	0.45364900
C	1.75671700	6.57951700	0.41790800
C	-11.16647200	-3.17413100	0.38649300
C	-9.40668400	-1.44053400	0.36702000
C	-7.65081900	0.29209300	0.36781300
C	-5.89561500	2.02444800	0.36938500
C	-4.14058300	3.75655300	0.41613600
C	-2.38699700	5.48852400	0.45771300
C	-0.62488800	7.23438800	0.41758300
C	-13.54691300	-2.51870900	0.39930300
C	-11.78782700	-0.78664800	0.39568100
C	-10.03263700	0.94674600	0.39252100
C	-8.27682200	2.68054500	0.37181700
C	-6.52030100	4.41214900	0.37742400

C	-4.76830000	6.14245200	0.44094000
C	-3.00649400	7.88925800	0.41725700
C	-15.93571800	-1.86646700	0.41280900
C	-14.17778100	-0.13136500	0.41349600
C	-12.41984300	1.60372900	0.41418300
C	-10.66190600	3.33882400	0.41487100
C	-8.90396900	5.07392000	0.41555800
C	-7.14602800	6.80901400	0.41624400
C	-5.38809500	8.54412500	0.41693200
H	6.88712700	-10.91814700	0.41554000
H	4.45050800	-11.55396800	0.44855000
H	2.07458300	-10.90060600	0.45601900
H	-0.30749900	-10.24550100	0.45504400
H	-2.68922900	-9.59055600	0.45497000
H	-5.07101500	-8.93565800	0.45522600
H	8.64075000	-9.18727300	0.40833700
H	10.39909400	-7.45169400	0.40937800
H	12.15734100	-5.71617000	0.40997000
H	13.91506700	-3.98115100	0.41149600
H	15.67221900	-2.24698600	0.41101500
H	17.42753100	-0.51489900	0.41400700
H	18.09661500	1.90927700	0.44814800
H	13.95078500	4.35060400	0.40973500
H	11.56920500	5.00510400	0.41249800
H	9.18860400	5.65956500	0.41277100
H	6.80752700	6.31437700	0.41160100
H	-7.45318600	-8.28060000	0.45555600
H	-9.83514900	-7.62543100	0.45476300
H	-12.21606100	-6.97056900	0.45304400
H	-14.59633900	-6.31617600	0.45361400
H	-16.97186200	-5.66331000	0.44845700
H	-18.74275200	-3.87435500	0.41565400
H	4.42568000	6.96943100	0.41063600
H	-18.07194600	-1.45047800	0.44579300
H	-16.31848200	0.28037800	0.45772000
H	-14.56018100	2.01542200	0.45515300
H	-12.80266900	3.75002200	0.45544300
H	-11.04474600	5.48525100	0.45632900
H	-9.28639000	7.22092700	0.45761000
H	-7.53263500	8.95195000	0.45043300
H	2.04360300	7.62445600	0.41044400
H	-0.33830400	8.27940100	0.41061800
H	-2.72028500	8.93442600	0.40988200
H	-5.09610400	9.58774600	0.41738800
H	16.32918900	3.69664200	0.41657800
C	-9.45111300	-2.92201500	3.77167000
C	-8.13632500	-2.52629700	3.55060500
C	-7.75068900	-1.19549500	3.60548300
C	-8.69866500	-0.22759700	3.90597700
C	-10.02561000	-0.59133700	4.09627000
C	-10.39051200	-1.92320300	4.02188200
O	-8.45163100	1.13142300	3.94040700
C	-7.20648600	1.66302800	4.10033700
C	-6.18583300	1.08493600	4.86381600
C	-4.93608000	1.66126500	4.87507600
C	-4.65141200	2.83833400	4.12951400
C	-5.76598400	3.49196900	3.54752100
C	-7.00847300	2.91060400	3.51214800
C	-3.38576500	3.39922700	3.87294200
C	-2.15352400	2.68802400	3.84446900
C	-0.95659300	3.43902400	3.80993200
C	0.26143400	2.80468800	3.70255800
C	0.34325200	1.41949400	3.65315800
C	-0.81213400	0.62950100	3.74319500
C	-2.04389100	1.28401500	3.77988700

C	-0.73662000	-0.83343000	3.78337500
C	0.45683300	-1.67149700	3.95291600
C	1.58144900	-1.23681100	4.66263200
C	2.74048800	-1.99364400	4.71438300
C	2.79629300	-3.22002000	4.06153100
C	1.64370300	-3.72812800	3.47649500
C	0.49848000	-2.96378000	3.41908600
O	3.89597400	-4.03929800	4.01476700
C	5.19238700	-3.57288800	3.96867700
C	5.57033700	-2.28208700	3.62658900
C	6.91821600	-1.95621800	3.58236600
C	7.91246200	-2.88867400	3.85299900
C	7.50679300	-4.18873600	4.15141300
C	6.16902300	-4.53118100	4.21750000
N	-1.86816300	-1.47448000	3.67995900
N	-2.86228600	-2.03011100	3.59317100
H	-7.39015100	-3.27158400	3.30674200
H	-6.73081300	-0.92531100	3.38408600
H	-10.75827300	0.17990400	4.28679000
H	-11.42978000	-2.19163900	4.16453200
H	-6.37586600	0.18425800	5.42828600
H	-4.15783500	1.21223300	5.47464800
H	-5.59473700	4.44405900	3.06981000
H	-7.83615500	3.38331400	3.00685300
H	-1.03241800	4.51654500	3.83455200
H	1.17001200	3.38607700	3.62126600
H	1.30496500	0.95805300	3.50094000
H	-2.95376800	0.70671400	3.73555100
H	1.55186500	-0.30226000	5.20076800
H	3.58933000	-1.63163400	5.27325700
H	1.67172900	-4.71027600	3.03098300
H	-0.36666900	-3.36997600	2.91725400
H	4.83499700	-1.54032600	3.36138200
H	7.19673200	-0.94707900	3.30691400
H	8.25281300	-4.95182500	4.33600000
H	5.86212700	-5.54170900	4.44742300
C	-9.83976900	-4.36709700	3.76299700
H	-9.15146500	-4.96295200	3.16898000
H	-9.83673100	-4.77822100	4.77452100
H	-10.83974100	-4.51220400	3.35938900
C	9.36113900	-2.51127000	3.83816300
H	9.72964800	-2.31968200	4.84777100
H	9.97655800	-3.30276400	3.41498200
H	9.53201300	-1.61068300	3.25370500

(3) DiazoCarbene -NO_2/Graphene

C	6.49777100	-10.28462400	-0.36683900
C	5.13311300	-10.69622700	-0.31093500
C	2.72964800	-10.13029100	-0.24806800
C	0.32618000	-9.56435400	-0.18520500
C	-2.07728900	-8.99841600	-0.12233900
C	-4.48075800	-8.43247700	-0.05947400
C	4.09429800	-9.71867000	-0.30397400
C	1.69082600	-9.15273500	-0.24110800
C	-0.71264200	-8.58679500	-0.17824300
C	-3.11611100	-8.02085800	-0.11537800
C	-5.51957900	-7.45491900	-0.05251200
C	8.19044000	-8.48580700	-0.36988000
C	9.88311100	-6.68698700	-0.37291800
C	11.57578200	-4.88816600	-0.37595700
C	13.26845200	-3.08934500	-0.37900000
C	14.96112300	-1.29052400	-0.38202900
C	16.65378800	0.50829800	-0.38514900
C	6.82577900	-8.89739000	-0.31397300
C	8.51845200	-7.09856900	-0.31701300

C	10.21112200	-5.29974800	-0.32005200
C	11.90379300	-3.50092700	-0.32309200
C	13.59646400	-1.70210600	-0.32612800
C	15.28913000	0.09672200	-0.32918700
C	16.98181000	1.89552000	-0.33218400
C	4.42579300	-8.31973500	-0.27657500
C	6.11359000	-6.52536900	-0.24491900
C	7.80416800	-4.73032000	-0.24320900
C	9.49547300	-2.93425400	-0.24456400
C	11.18633400	-1.13814300	-0.23054300
C	12.87971400	0.65957800	-0.23335600
C	14.57834300	2.46145700	-0.26932100
C	2.02149400	-7.75382400	-0.24245000
C	3.70977800	-5.95730100	-0.23326000
C	5.40064800	-4.16215600	-0.21195100
C	7.09120400	-2.36730200	-0.20745100
C	8.78338800	-0.57219100	-0.17789400
C	10.47704700	1.22523000	-0.16669100
C	12.17487200	3.02739400	-0.20648900
C	-0.38199200	-7.18802200	-0.19083700
C	1.30693600	-5.39104600	-0.22957500
C	2.99714300	-3.59543600	-0.19673900
C	4.68898300	-1.80148300	-0.15664400
C	6.38138600	-0.00638700	-0.12175800
C	8.07469800	1.79189500	-0.10656500
C	9.77140300	3.59333200	-0.14361300
C	-2.78484400	-6.62211800	-0.11980100
C	-1.09482800	-4.82610000	-0.16368000
C	0.59536800	-3.02949500	-0.17634300
C	2.28654400	-1.23602200	-0.10793500
C	3.97865400	0.55980300	-0.06273200
C	5.67186800	2.35742100	-0.04687500
C	7.36793500	4.15927000	-0.08074800
C	5.77728700	-7.91359300	-0.28163900
C	7.47037400	-6.11593700	-0.26822100
C	9.16234400	-4.31853100	-0.27391100
C	10.85471500	-2.52098200	-0.26916900
C	12.54819100	-0.72370100	-0.26628100
C	14.24587600	1.07155300	-0.27153700
C	15.94298600	2.87308000	-0.32520500
C	3.37903300	-7.34732100	-0.26416100
C	5.07018700	-5.54927500	-0.23857500
C	6.76119100	-3.75343700	-0.23312100
C	8.45343100	-1.95738700	-0.22492100
C	10.14654000	-0.16283000	-0.19921700
C	11.84072300	1.63148200	-0.20633300
C	13.53951800	3.43901100	-0.26245000
C	0.97745800	-6.78266100	-0.23480500
C	2.66825000	-4.98278900	-0.23730800
C	4.35966100	-3.18736000	-0.19926100
C	6.05150900	-1.39249300	-0.17822400
C	7.74465900	0.40183900	-0.14182200
C	9.43819200	2.19653600	-0.14370300
C	11.13605200	4.00495200	-0.19951800
C	-1.42440900	-6.21762400	-0.17047700
C	0.26629200	-4.41691600	-0.21776800
C	1.95785500	-2.62148900	-0.17655500
C	3.64918400	-0.82762900	-0.11606200
C	5.34177200	0.96764300	-0.08240700
C	7.03572400	2.76174600	-0.08352400
C	8.73258300	4.57089200	-0.13665400
C	-3.82590000	-5.65200800	-0.09434400
C	-2.13471500	-3.85310000	-0.11746500
C	-0.44364000	-2.05612500	-0.12449700
C	1.24710100	-0.26110900	-0.07313400

C	2.93911500	1.53322600	-0.03470700
C	4.63222800	3.32688100	-0.02164600
C	6.32911400	5.13682900	-0.07378800
C	-6.88422600	-7.86653900	0.00339200
C	-7.92304800	-6.88898100	0.01035200
C	-9.28769500	-7.30060100	0.06625800
C	-11.69116500	-6.73466400	0.12912200
C	-14.09463300	-6.16872600	0.19198900
C	-16.49809500	-5.60278400	0.25485300
C	-10.32651700	-6.32304300	0.07321800
C	-12.72998500	-5.75710400	0.13608300
C	-15.13345500	-5.19116300	0.19894900
C	-17.53692700	-4.62524100	0.26181300
C	-5.18825400	-6.05684100	-0.05214300
C	-3.49673700	-4.26204600	-0.07063100
C	-1.80502100	-2.46539600	-0.08463500
C	-0.11464900	-0.66948200	-0.06020400
C	1.57576500	1.12625800	-0.02647100
C	3.26940400	2.92287200	0.00802800
C	4.96446700	4.72520900	-0.01788300
C	-6.22814600	-5.08764600	-0.02836100
C	-4.53614300	-3.28913100	-0.03387700
C	-2.84468200	-1.49182700	-0.03267900
C	-1.15272900	0.30419100	-0.01316500
C	0.53684500	2.09920300	0.00185100
C	2.22822100	3.89290300	0.03161300
C	3.92564500	5.70276700	-0.01092300
C	-7.59173700	-5.49219000	0.00736400
C	-5.89796700	-3.69766900	-0.00299100
C	-4.20607200	-1.90153000	0.00316400
C	-2.51486500	-0.10585000	0.01747400
C	-0.82407100	1.69089700	0.03138700
C	0.86697400	3.48867100	0.05222200
C	2.56099800	5.29114700	0.04498200
C	-9.99354900	-4.92765800	0.05520500
C	-8.30154800	-3.13236500	0.03547600
C	-6.60931300	-1.33667400	0.05498100
C	-4.91599800	0.45877500	0.08325000
C	-3.22416500	2.25555600	0.09899700
C	-1.53424000	4.05358500	0.11916600
C	0.15752900	5.85708500	0.10784800
C	-12.39560300	-4.36110400	0.12664000
C	-10.70343600	-2.56859800	0.09375300
C	-9.01030500	-0.77260300	0.09302600
C	-7.31764200	1.02467600	0.12032300
C	-5.62597100	2.82153400	0.12159600
C	-3.93398100	4.61753500	0.16202100
C	-2.24593900	6.42302300	0.17071300
C	-14.80186400	-3.80181300	0.20150200
C	-13.10269100	-2.00878100	0.19043900
C	-11.40991800	-0.20939900	0.18522300
C	-9.71869000	1.58885800	0.19012600
C	-8.02564000	3.38663300	0.17495300
C	-6.33172800	5.18379900	0.19017200
C	-4.64941100	6.98895900	0.23357900
C	-17.20890100	-3.23800500	0.31468000
C	-15.51623300	-1.43918900	0.31164100
C	-13.82356400	0.35963400	0.30860100
C	-12.13089400	2.15845500	0.30556200
C	-10.43822400	3.95727600	0.30252300
C	-8.74555300	5.75609700	0.29948400
C	-7.05288400	7.55491300	0.29644400
C	-8.63219800	-4.52297900	0.01758800
C	-6.93882600	-2.72389500	0.01796200
C	-5.24539100	-0.92764200	0.04171400

C	-3.55393600	0.86800000	0.06156400
C	-1.86405700	2.66383800	0.07463200
C	-0.17421700	4.45886400	0.08985100
C	1.52217600	6.26870500	0.05194300
C	-11.03444000	-3.95607500	0.07307500
C	-9.34013600	-2.15775000	0.05343000
C	-7.64898300	-0.36215900	0.07513600
C	-5.95711100	1.43400400	0.10242400
C	-4.26542000	3.22943900	0.11233500
C	-2.57634900	5.02394400	0.15379300
C	-0.88129200	6.83464300	0.11480900
C	-13.43485400	-3.38986200	0.15944900
C	-11.74350100	-1.59392500	0.14066500
C	-10.05275500	0.20412100	0.14411700
C	-8.36034400	2.00191900	0.14928300
C	-6.66861900	3.79678200	0.13394700
C	-4.98063000	5.58960800	0.18952700
C	-3.28476200	7.40058100	0.17767300
C	-15.84424700	-2.82643100	0.25877400
C	-14.15157700	-1.02760400	0.25573500
C	-12.45890600	0.77121600	0.25269500
C	-10.76623600	2.57003700	0.24965700
C	-9.07356500	4.36885900	0.24661700
C	-7.38089100	6.16767900	0.24357700
C	-5.68822600	7.96651500	0.24053900
H	7.28998300	-11.02347600	-0.39775100
H	4.87964800	-11.74977800	-0.32536500
H	2.48208100	-11.18528200	-0.25485900
H	0.07811300	-10.61911500	-0.19262800
H	-2.32547700	-10.05311200	-0.12950800
H	-4.72911500	-9.48716400	-0.06605700
H	8.97832500	-9.22888600	-0.40866700
H	10.67139200	-7.42957400	-0.41135200
H	12.36435400	-5.63031100	-0.41448500
H	14.05683500	-3.83158900	-0.41668300
H	15.74872900	-2.03370500	-0.42089100
H	17.43893700	-0.23803000	-0.42162900
H	18.01919500	2.20825400	-0.35480000
H	13.78671900	4.49435100	-0.25802200
H	11.38335700	5.05986200	-0.19207800
H	8.98091900	5.62541700	-0.12864400
H	6.57796200	6.19133200	-0.06663500
H	-7.13314100	-8.92107200	-0.00252100
H	-9.53698800	-8.35484000	0.05989000
H	-11.93979500	-7.78885900	0.12134700
H	-14.34190000	-7.22336600	0.18507000
H	-16.73932400	-6.65911600	0.24293700
H	-18.57496700	-4.93680500	0.28232200
H	4.17423100	6.75745600	-0.00440100
H	-17.99306600	-2.49068200	0.34510900
H	-16.30446300	-0.69647100	0.35331700
H	-14.61149800	1.10237300	0.34701600
H	-12.91924300	2.90069100	0.34357900
H	-11.22658800	4.69964100	0.34074200
H	-9.53350700	6.49904700	0.33829800
H	-7.84503800	8.29379000	0.32740800
H	1.77028900	7.32352800	0.05861100
H	-0.63347300	7.88952000	0.12198300
H	-3.03733200	8.45560600	0.18444900
H	-5.43479100	9.02006400	0.25498900
H	16.18709800	3.92908100	-0.31428700
C	-9.97948500	-1.90830800	3.43385300
C	-8.61223900	-1.75985000	3.25970600
C	-8.03162900	-0.51622100	3.40581900
C	-8.82513900	0.57196600	3.76665300

C	-10.20507700	0.41968000	3.90759000
C	-10.78487000	-0.81629400	3.73061000
O	-8.36470900	1.83941400	3.96153000
C	-7.02135600	2.14675100	3.98826500
C	-6.07951800	1.42614200	4.72422900
C	-4.74974300	1.77969400	4.65959200
C	-4.31650800	2.88461700	3.87651800
C	-5.33185700	3.68244800	3.29151600
C	-6.65202200	3.30691100	3.32010100
C	-2.98887800	3.27766400	3.65509000
C	-1.83092900	2.46997600	3.53104400
C	-0.58435800	3.12366500	3.41461500
C	0.57080300	2.39306900	3.24080000
C	0.53528100	1.00754100	3.21145300
C	-0.67239900	0.31265100	3.37977700
C	-1.84464500	1.06019100	3.48642900
C	-0.68966200	-1.15123600	3.41220000
C	0.47093700	-2.03918300	3.54508900
C	1.59528600	-1.66892000	4.29214600
C	2.73748000	-2.45094400	4.30966800
C	2.77002300	-3.63039500	3.57714400
C	1.62323400	-4.07954800	2.94281100
C	0.49037200	-3.29203300	2.92668400
O	3.86642000	-4.47018000	3.50761800
C	5.14260800	-4.02334500	3.34467900
C	5.50487000	-2.70767200	3.05695800
C	6.84181300	-2.37793800	2.94956900
C	7.81024100	-3.35872900	3.09493900
C	7.45439200	-4.68381200	3.31273600
C	6.12399300	-5.01210300	3.44493500
N	-1.85216400	-1.73268500	3.30060400
N	-2.87376000	-2.23409300	3.21502000
H	-8.01528200	-2.61582000	2.98960300
H	-6.97694600	-0.39451600	3.22200100
H	-10.80342600	1.28593100	4.14770800
H	-11.85001600	-0.95161600	3.82912700
H	-6.39146900	0.58795400	5.33049000
H	-4.02926900	1.22275300	5.24040500
H	-5.03342400	4.58035800	2.77419200
H	-7.41227400	3.89300100	2.82768300
H	-0.57164800	4.20361000	3.43460800
H	1.51638100	2.89790500	3.09772700
H	1.44598500	0.46363600	3.01950400
H	-2.79913700	0.55759800	3.52711100
H	1.57754200	-0.76178600	4.87673000
H	3.58799600	-2.14524300	4.89972800
H	1.64484700	-5.02695000	2.42765700
H	-0.37660900	-3.64372500	2.38854500
H	4.75655400	-1.95034200	2.89560000
H	7.14117900	-1.36362700	2.73945100
H	8.22340400	-5.43610700	3.38486800
H	5.81894700	-6.03189700	3.62725000
N	-10.58183500	-3.23058600	3.30184500
O	-11.81037800	-3.31172800	3.34204300
O	-9.82745500	-4.19429600	3.16092000
N	9.22113100	-2.99431000	3.01632900
O	9.50248400	-1.79816100	2.93166900
O	10.05290600	-3.90232700	3.04205700

(4) DiazoCarbene -NH_2/Graphene

C	6.44518200	-9.16715300	-0.01757900
C	5.06531100	-9.52723900	0.01603400
C	2.68371000	-8.87237100	0.01571000
C	0.30210500	-8.21750100	0.01538300
C	-2.07950100	-7.56263000	0.01505800

C	-4.46110600	-6.90775900	0.01473300
C	4.06357300	-8.51226600	-0.01790500
C	1.68196400	-7.85739800	-0.01822900
C	-0.69964100	-7.20252600	-0.01855500
C	-3.08124600	-6.54765600	-0.01888000
C	-5.46285100	-5.89278500	-0.01920500
C	8.20311700	-7.43206200	-0.01689300
C	9.96105500	-5.69696700	-0.01620500
C	11.71899300	-3.96187200	-0.01551700
C	13.47693000	-2.22677700	-0.01483400
C	15.23486800	-0.49168200	-0.01413600
C	16.99280100	1.24341300	-0.01353000
C	6.82324500	-7.79212800	0.01672200
C	8.58118500	-6.05703300	0.01740800
C	10.33912200	-4.32193800	0.01809600
C	12.09706000	-2.58684200	0.01878200
C	13.85499700	-0.85174700	0.01947300
C	15.61293100	0.88335500	0.02014000
C	17.37087600	2.61842800	0.02087000
C	4.44656600	-7.12499800	-0.01326000
C	6.19834100	-5.39428900	-0.00406800
C	7.95430700	-3.66419400	0.03475400
C	9.71043100	-1.93087400	0.03380000
C	11.46520200	-0.19765300	0.03087400
C	13.22427900	1.53551000	0.04375800
C	14.98927200	3.27329700	0.02054300
C	2.06375900	-6.47085500	-0.02856100
C	3.81739400	-4.73905500	-0.01062600
C	5.57267400	-3.00812300	0.03065000
C	7.32868100	-1.27552300	0.04080400
C	9.08367700	0.45565000	0.02057700
C	10.84378100	2.18939900	0.04267900
C	12.60766500	3.92816600	0.02018400
C	-0.31707400	-5.81721700	-0.04161900
C	1.43767500	-4.08480000	-0.03579100
C	3.19150000	-2.35342100	0.01380800
C	4.94694400	-0.62196500	0.05728100
C	6.70483000	1.11095000	0.05719600
C	8.46251200	2.84642700	0.04924900
C	10.22606000	4.58303700	0.01987000
C	-2.69720000	-5.16265400	-0.04534900
C	-0.94102600	-3.43122800	-0.07172100
C	0.81228800	-1.70001800	-0.03208100
C	2.56805500	0.03215500	0.02380200
C	4.32403400	1.76579500	0.05710300
C	6.08098000	3.50030200	0.05702600
C	7.84445500	5.23790700	0.01954400
C	5.81129600	-6.76890100	-0.00594500
C	7.56922200	-5.03531000	0.00782300
C	9.32708200	-3.30243000	0.02787000
C	11.08304000	-1.56869500	0.02586600
C	12.84147000	0.16615200	0.02747500
C	14.60670000	1.89665200	0.03603500
C	16.36912800	3.63340400	-0.01305000
C	3.43622500	-6.11487400	-0.03402000
C	5.19201600	-4.38149700	-0.01321300
C	6.94747100	-2.64978400	0.03040200
C	8.70460400	-0.91634900	0.01179300
C	10.46208500	0.81460700	0.01305100
C	12.22177900	2.54495500	0.03168800
C	13.98752500	4.28826600	-0.01348500
C	1.05798400	-5.46227500	-0.04984500
C	2.81220800	-3.72760000	-0.02042300
C	4.56804500	-1.99530600	0.03085200
C	6.32511700	-0.26357600	0.04433000

C	8.08147300	1.46785000	0.03315900
C	9.83999800	3.20111100	0.03452400
C	11.60592100	4.94314100	-0.01374400
C	-1.32170400	-4.80896700	-0.07150700
C	0.43434400	-3.07372600	-0.06273500
C	2.18961000	-1.34158100	-0.00723300
C	3.94430700	0.39025500	0.04184900
C	5.70084000	2.12321200	0.05234400
C	7.46024300	3.85474900	0.03778000
C	9.22431600	5.59801300	-0.01407100
C	-3.70190500	-4.15454000	-0.06313900
C	-1.94351700	-2.42066800	-0.08485100
C	-0.18906300	-0.68865900	-0.05386100
C	1.56547100	1.04470100	-0.00818500
C	3.32215600	2.77684100	0.03440700
C	5.07853500	4.50712000	0.03745600
C	6.84271000	6.25288300	-0.01439500
C	-6.84271100	-6.25288800	0.01440800
C	-7.84445700	-5.23791400	-0.01953100
C	-9.22431700	-5.59801800	0.01408300
C	-11.60592300	-4.94314800	0.01375700
C	-13.98752800	-4.28827700	0.01343300
C	-16.36912600	-3.63340300	0.01310700
C	-10.22606200	-4.58304300	-0.01985600
C	-12.60766700	-3.92817200	-0.02018100
C	-14.98927400	-3.27329800	-0.02050600
C	-17.37088300	-2.61844300	-0.02083200
C	-5.07897900	-4.50755400	-0.04161900
C	-3.32094600	-2.77728200	-0.06738000
C	-1.56509500	-1.04593200	-0.06378100
C	0.18934400	0.68679700	-0.03582700
C	1.94457900	2.41905300	0.01767200
C	3.70200800	4.15349200	0.04793900
C	5.46285000	5.89277900	0.01921900
C	-6.08170200	-3.50006800	-0.06639900
C	-4.32371600	-1.76587600	-0.08494900
C	-2.56797200	-0.03308800	-0.08329800
C	-0.81270700	1.69831500	-0.07560200
C	0.94254800	3.43026700	-0.00859100
C	2.69721000	5.16209900	0.02812100
C	4.46110500	6.90775300	-0.01472000
C	-7.46032800	-3.85367200	-0.04587700
C	-5.70124500	-2.12268200	-0.07599100
C	-3.94470200	-0.39036200	-0.08448200
C	-2.18952600	1.34067200	-0.08312600
C	-0.43375100	3.07276700	-0.04141800
C	1.32305900	4.80854800	0.02610400
C	3.08124500	6.54765000	0.01889300
C	-9.84082000	-3.19999600	-0.04550700
C	-8.08074300	-1.46837800	-0.08431700
C	-6.32467300	0.26385600	-0.11722500
C	-4.56835700	1.99446700	-0.08971400
C	-2.81249200	3.72641200	-0.05727800
C	-1.05717100	5.46227100	0.00832400
C	0.69963900	7.20252000	0.01856800
C	-12.22222500	-2.54610200	-0.04072000
C	-10.46213100	-0.81531200	-0.06896000
C	-8.70329400	0.91739000	-0.10564300
C	-6.94732900	2.64836500	-0.08431100
C	-5.19175700	4.38016900	-0.04044900
C	-3.43617000	6.11509500	0.00652700
C	-1.68196600	7.85739100	0.01824300
C	-14.60602900	-1.89692800	-0.03622900
C	-12.84358900	-0.16612400	-0.04270000
C	-11.08338300	1.56829900	-0.05544200

C	-9.32615400	3.30211000	-0.05253900
C	-7.56970700	5.03526600	-0.03624400
C	-5.81166100	6.76968500	-0.01198100
C	-4.06357400	8.51226000	0.01791800
C	-16.99280100	-1.24341700	0.01346900
C	-15.23486700	0.49167300	0.01415700
C	-13.47693100	2.22677100	0.01484300
C	-11.71899400	3.96186600	0.01553100
C	-9.96105700	5.69696100	0.01621800
C	-8.20311900	7.43205600	0.01690600
C	-6.44518300	9.16714600	0.01759200
C	-8.46217400	-2.84597300	-0.06942800
C	-6.70371000	-1.11046300	-0.10899500
C	-4.94783000	0.62147800	-0.11471400
C	-3.19188700	2.35175800	-0.09370600
C	-1.43766300	4.08466900	-0.04651900
C	0.31678500	5.81734700	0.01473900
C	2.07949900	7.56262400	-0.01504500
C	-10.84355000	-2.19160600	-0.06360100
C	-9.08341600	-0.45578900	-0.10349300
C	-7.32710000	1.27614800	-0.12374400
C	-5.57239800	3.00658300	-0.08256000
C	-3.81750600	4.73863300	-0.04243900
C	-2.06418400	6.47168600	0.01060500
C	-0.30210600	8.21749500	-0.01537000
C	-13.22492600	-1.53650900	-0.05289300
C	-11.46692500	0.19761600	-0.07196200
C	-9.70922100	1.93120400	-0.08980200
C	-7.95341100	3.66335900	-0.06966600
C	-6.19801600	5.39454400	-0.04085200
C	-4.44598600	7.12597000	0.00202600
C	-2.68371200	8.87236500	-0.01569600
C	-15.61293600	-0.88336000	-0.02014400
C	-13.85499900	0.85174200	-0.01945700
C	-12.09706100	2.58683600	-0.01877000
C	-10.33912400	4.32193100	-0.01808200
C	-8.58118700	6.05702700	-0.01739500
C	-6.82324600	7.79212100	-0.01670900
C	-5.06531300	9.52723200	-0.01602100
H	7.20990900	-9.93504000	-0.01741300
H	4.77329000	-10.57086100	0.01559700
H	2.39736500	-9.91749900	0.02306600
H	0.01528300	-9.26239400	0.02209100
H	-2.36644700	-8.60744900	0.02201700
H	-4.74823300	-7.95255100	0.02227300
H	8.96353200	-8.20416600	-0.02461600
H	10.72187600	-6.46858700	-0.02357500
H	12.48012300	-4.73306300	-0.02298300
H	14.23784900	-2.99804400	-0.02145700
H	15.99500100	-1.26387900	-0.02193800
H	17.75031300	0.46820800	-0.01894600
H	18.41939700	2.89238400	0.01519500
H	14.27356700	5.33371100	-0.02321800
H	11.89198700	5.98821100	-0.02045500
H	9.51138600	6.64267200	-0.02018200
H	7.13030900	7.29748400	-0.02135200
H	-7.13040400	-7.29749300	0.02260300
H	-9.51236700	-6.64232400	0.02181000
H	-11.89327900	-5.98746200	0.02009100
H	-14.27355700	-5.33306900	0.02066100
H	-16.64908000	-4.68020300	0.01550400
H	-18.41997000	-2.89124800	-0.01729900
H	4.74846200	7.95253800	-0.02231700
H	-17.74916400	-0.46737100	0.01284000
H	-15.99570000	1.26348500	0.02476700

H	-14.23739900	2.99852900	0.02220000
H	-12.47988700	4.73312900	0.02249000
H	-10.72196400	6.46835800	0.02337600
H	-8.96360800	8.20403400	0.02465700
H	-7.20985300	9.93505700	0.01748000
H	2.36638500	8.60756300	-0.02250900
H	-0.01552200	9.26250800	-0.02233500
H	-2.39750300	9.91753300	-0.02307100
H	-4.77332200	10.57085300	-0.01556500
H	16.65197100	4.67974900	-0.01637500
C	10.91244800	-0.14620800	3.32372500
C	9.53659200	0.00030500	3.14867900
C	8.67662600	-1.08241500	3.22235800
C	9.17737600	-2.34228300	3.51995800
C	10.54589100	-2.50385200	3.70724800
C	11.40081400	-1.42663500	3.60350900
O	8.45375600	-3.51983900	3.55512200
C	7.09899000	-3.61392100	3.62715000
C	6.24058200	-2.66303800	4.18693900
C	4.87824600	-2.86181900	4.15374100
C	4.30994500	-4.01892300	3.55799200
C	5.21658900	-5.01700800	3.11757600
C	6.57149800	-4.81851400	3.15516100
C	2.94748200	-4.21424900	3.38996300
C	1.70733600	-3.58072500	3.35747300
C	0.53179300	-4.35802500	3.21583000
C	-0.69122600	-3.73793800	3.10248200
C	-0.80721800	-2.35534100	3.15267400
C	0.32625900	-1.55307500	3.35182900
C	1.57153700	-2.17649900	3.41904100
C	0.20972800	-0.09643300	3.45318800
C	-1.02107100	0.68860000	3.60425200
C	-2.13777000	0.19243400	4.28485000
C	-3.33919700	0.88071700	4.29838600
C	-3.44821800	2.09899600	3.63469100
C	-2.30702900	2.67372700	3.08726500
C	-1.11826300	1.97637600	3.06762200
O	-4.59411700	2.84032600	3.53121400
C	-5.85643700	2.27823200	3.45203500
C	-6.13059800	0.96162900	3.10910300
C	-7.44329400	0.53248900	3.01186700
C	-8.51309800	1.40313400	3.21609500
C	-8.21800800	2.73393400	3.52464400
C	-6.91050400	3.16177000	3.64964300
N	1.31906900	0.58407700	3.37046900
N	2.29451600	1.17394500	3.29410200
H	9.13055400	0.97371100	2.90841500
H	7.63476200	-0.93097800	2.99624900
H	10.92976200	-3.49411300	3.90701900
H	12.46510500	-1.57807900	3.73093200
H	6.63680000	-1.78346400	4.66847900
H	4.23327700	-2.12126700	4.60240600
H	4.81819500	-5.93779800	2.71794300
H	7.25284200	-5.57062000	2.78705000
H	0.61741100	-5.43376800	3.16333200
H	-1.58089200	-4.33344900	2.94883200
H	-1.77286500	-1.90330200	3.00116700
H	2.46563300	-1.57992000	3.50993800
H	-2.07023100	-0.74253300	4.81985000
H	-4.18321600	0.47110000	4.83095100
H	-2.37737000	3.65210800	2.63818700
H	-0.26269200	2.42879200	2.58944100
H	-5.33603200	0.26986700	2.88183500
H	-7.63898500	-0.49568600	2.73687100
H	-9.02426200	3.44217900	3.66785200

H	-6.68950400	4.19437600	3.88035100
N	11.76468700	0.94929900	3.26076300
N	-9.82851400	0.94974000	3.16552100
H	-9.96015900	0.13803300	2.58306500
H	-10.49882800	1.65971600	2.91815000
H	12.70893800	0.71952400	2.99621400
H	11.42340400	1.70159200	2.68376600

2.7.4 Optimizations of Biscarbene onto Graphene via cluster model

(1) Biscarbene-H/Graphene

C	6.65373800	-9.50536300	0.72194000
C	5.27386700	-9.86544900	0.75555300
C	2.89226600	-9.21058100	0.75522900
C	0.51066100	-8.55571100	0.75490200
C	-1.87094500	-7.90084000	0.75457600
C	-4.25255000	-7.24596900	0.75425100
C	4.27212900	-8.85047600	0.72161300
C	1.89052000	-8.19560800	0.72128900
C	-0.49108500	-7.54073600	0.72096400
C	-2.87269000	-6.88586600	0.72063900
C	-5.25429500	-6.23099500	0.72031400
C	8.41167300	-7.77027200	0.72262600
C	10.16961100	-6.03517700	0.72331400
C	11.92754900	-4.30008200	0.72400200
C	13.68548600	-2.56498700	0.72468400
C	15.44342400	-0.82989200	0.72538200
C	17.20135700	0.90520300	0.72598900
C	7.03180100	-8.13033800	0.75624000
C	8.78974100	-6.39524300	0.75692600
C	10.54767800	-4.66014800	0.75761400
C	12.30561600	-2.92505200	0.75830100
C	14.06355300	-1.18995700	0.75899200
C	15.82148700	0.54514500	0.75965900
C	17.57943200	2.28021800	0.76038900
C	4.65441800	-7.46400400	0.72822400
C	6.40581000	-5.73273600	0.74686500
C	8.16149500	-4.00101800	0.75331800
C	9.91842700	-2.26771700	0.78488800
C	11.67427100	-0.53452500	0.80245600
C	13.43297800	1.19762800	0.79676200
C	15.19782800	2.93508700	0.76006200
C	2.27281600	-6.80994800	0.71259500
C	4.02586400	-5.07699700	0.72155100
C	5.78107100	-3.34423000	0.71798500
C	7.53606400	-1.61265000	0.75555500
C	9.29228400	0.12023100	0.79954300
C	11.05234300	1.85364700	0.80334500
C	12.81622100	3.58995600	0.75970300
C	-0.10759000	-6.15583300	0.70539700
C	1.64731800	-4.42243600	0.70117700
C	3.40085000	-2.69027000	0.72486600
C	5.15633100	-0.95920700	0.76158300
C	6.91436300	0.77407200	0.78429600
C	8.67176800	2.50960800	0.78449800
C	10.43461600	4.24482700	0.75938900
C	-2.48795200	-5.50035200	0.69902000
C	-0.73236300	-3.76844400	0.67281500
C	1.02200300	-2.03722300	0.68753300
C	2.77682500	-0.30491000	0.74000300
C	4.53344600	1.42789100	0.77916100
C	6.29110100	3.16211100	0.77760000
C	8.05301100	4.89969700	0.75906300
C	6.01974600	-7.10820700	0.74147300

C	7.77764700	-5.37391200	0.75199000
C	9.53499600	-3.63989600	0.76343200
C	11.29200000	-1.90562100	0.77981600
C	13.05124900	-0.17160100	0.78242600
C	14.81467700	1.55810100	0.77748200
C	16.57768400	3.29519400	0.72646900
C	3.64477300	-6.45340000	0.70826300
C	5.39942100	-4.71898300	0.71040200
C	7.15522900	-2.98588900	0.71679500
C	8.91235100	-1.25254100	0.77113300
C	10.67061900	0.47868900	0.79707400
C	12.42939500	2.20735200	0.78339500
C	14.19608100	3.95005600	0.72603400
C	1.26630500	-5.80073500	0.69360000
C	3.02087400	-4.06485800	0.70617100
C	4.77692300	-2.33180000	0.72428700
C	6.53383500	-0.60002500	0.76093300
C	8.29049100	1.13229500	0.78004600
C	10.04876400	2.86358000	0.77894800
C	11.81447700	4.60493100	0.72577500
C	-1.11402500	-5.14675700	0.68128700
C	0.64353000	-3.41103300	0.67071900
C	2.39867300	-1.67905400	0.70790800
C	4.15311300	0.05268900	0.75794900
C	5.91054000	1.78564100	0.77059800
C	7.66969600	3.51644700	0.76269400
C	9.43287200	5.25980300	0.72544800
C	-3.49365600	-4.49205300	0.68025900
C	-1.73478300	-2.75785300	0.65706500
C	0.02045700	-1.02533900	0.66571800
C	1.77457000	0.70685300	0.71223200
C	3.53025400	2.43841100	0.75809400
C	5.28745300	4.16839300	0.76857400
C	7.05126600	5.91467300	0.72512400
C	-6.63415500	-6.59109800	0.75392600
C	-7.63590100	-5.57612400	0.71998800
C	-9.01576100	-5.93622800	0.75360100
C	-11.39736700	-5.28135800	0.75327600
C	-13.77897200	-4.62648700	0.75295200
C	-16.16057000	-3.97161300	0.75262600
C	-10.01750600	-4.92125300	0.71966300
C	-12.39911100	-4.26638200	0.71933800
C	-14.78071800	-3.61150800	0.71901300
C	-17.16232700	-2.95665300	0.71868700
C	-4.86986200	-4.84511100	0.69890600
C	-3.11271700	-3.11505300	0.67944600
C	-1.35550600	-1.38302800	0.66740100
C	0.39880900	0.34946600	0.68548900
C	2.15330200	2.08085600	0.73591800
C	3.91118400	3.81513000	0.77612100
C	5.67140600	5.55456900	0.75873800
C	-5.87288800	-3.83855400	0.67692800
C	-4.11588000	-2.10410200	0.66219200
C	-2.35931500	-0.37081000	0.65861400
C	-0.60301000	1.36093800	0.65459300
C	1.15176100	3.09165400	0.70177400
C	2.90602300	4.82380400	0.76004900
C	4.66966100	6.56954300	0.72479900
C	-7.25204700	-4.19297600	0.69618900
C	-5.49262600	-2.46140600	0.67017300
C	-3.73598800	-0.72826900	0.65178200
C	-1.97995600	1.00367100	0.66326300
C	-0.22431500	2.73505900	0.66930700
C	1.53208300	4.46962700	0.74790500
C	3.28980100	6.20944000	0.75841200

C	-9.63223200	-3.53955200	0.69439500
C	-7.87282900	-1.80694100	0.66762700
C	-6.11681300	-0.07451400	0.62880500
C	-4.35972400	1.65709800	0.64410400
C	-2.60276100	3.38951300	0.66991900
C	-0.84824500	5.12370100	0.72591800
C	0.90819500	6.86431000	0.75808700
C	-12.01302900	-2.88350000	0.69938300
C	-10.25409600	-1.15403000	0.67402300
C	-8.49521000	0.57833300	0.65000000
C	-6.73899400	2.31120500	0.64958400
C	-4.98338300	4.04278500	0.69626800
C	-3.22757300	5.77682200	0.73595500
C	-1.47341000	7.51918100	0.75776200
C	-14.39771200	-2.23452700	0.70297000
C	-12.63437300	-0.50462400	0.69910700
C	-10.87467700	1.22999300	0.69128900
C	-9.11786900	2.96446900	0.68843200
C	-7.36110900	4.69746800	0.70089000
C	-5.60287200	6.43158800	0.72556400
C	-3.85501800	8.17405000	0.75743700
C	-16.78424500	-1.58162700	0.75298800
C	-15.02631100	0.15346300	0.75367600
C	-13.26837500	1.88856100	0.75436200
C	-11.51043800	3.62365600	0.75505000
C	-9.75250100	5.35875100	0.75573700
C	-7.99456300	7.09384600	0.75642500
C	-6.23662700	8.82893600	0.75711100
C	-8.25459400	-3.18533500	0.67426600
C	-6.49631100	-1.44916600	0.64699000
C	-4.73946400	0.28390100	0.65988000
C	-2.98326300	2.01469700	0.65394800
C	-1.22849900	3.74689800	0.66178800
C	0.52580500	5.47915900	0.74336600
C	2.28805500	7.22441400	0.72447400
C	-10.63607200	-2.52930900	0.67617900
C	-8.87543700	-0.79461700	0.64891200
C	-7.11923500	0.93800100	0.62520500
C	-5.36484600	2.66910100	0.65727700
C	-3.60903100	4.40094200	0.68660900
C	-1.85547500	6.13320200	0.74229900
C	-0.09355000	7.87928500	0.72414900
C	-13.01605700	-1.87387900	0.68617800
C	-11.25777300	-0.14115900	0.67447400
C	-9.50156000	1.59296500	0.66154100
C	-7.74526900	3.32582900	0.66668600
C	-5.98976700	5.05657900	0.69614900
C	-4.23776100	6.78770100	0.74057900
C	-2.47515600	8.53415500	0.72382300
C	-15.40438000	-1.22157000	0.71937500
C	-13.64644300	0.51353200	0.72006200
C	-11.88850500	2.24862600	0.72074900
C	-10.13056800	3.98372100	0.72143700
C	-8.37263100	5.71881700	0.72212400
C	-6.61469000	7.45391100	0.72281000
C	-4.85675700	9.18902200	0.72349800
H	7.41846500	-10.27325000	0.72210500
H	4.98184600	-10.90907100	0.75511600
H	2.60592100	-10.25570900	0.76258400
H	0.22383900	-9.60060400	0.76161000
H	-2.15789100	-8.94565900	0.76153600
H	-4.53967700	-8.29076100	0.76179200
H	9.17208800	-8.54237600	0.71490200
H	10.93043200	-6.80679700	0.71594300
H	12.68867900	-5.07127300	0.71653600

H	14.44640500	-3.33625400	0.71806200
H	16.20355700	-1.60208900	0.71758100
H	17.95886900	0.12999800	0.72057300
H	18.62795300	2.55417400	0.75471400
H	14.48212300	4.99550100	0.71630100
H	12.10054300	5.65000100	0.71906400
H	9.71994200	6.30446200	0.71933700
H	7.33886500	6.95927400	0.71816700
H	-6.92184800	-7.63570300	0.76212200
H	-9.30381100	-6.98053400	0.76132900
H	-11.68472300	-6.32567200	0.75961000
H	-14.06500100	-5.67127900	0.76018000
H	-16.44052400	-5.01841300	0.75502300
H	-18.21141400	-3.22945800	0.72222000
H	4.95701800	7.61432800	0.71720200
H	-17.54060800	-0.80558100	0.75235900
H	-15.78714400	0.92527500	0.76428600
H	-14.02884300	2.66031900	0.76171900
H	-12.27133100	4.39491900	0.76200900
H	-10.51340800	6.13014800	0.76289500
H	-8.75505200	7.86582400	0.76417600
H	-7.00129700	9.59684700	0.75699900
H	2.57494100	8.26935300	0.71701000
H	0.19303400	8.92429800	0.71718400
H	-2.18894700	9.57932300	0.71644800
H	-4.56476600	10.23264300	0.72395400
H	16.86052700	4.34153900	0.72314400
C	8.76276100	3.69548700	4.08200500
C	7.50033200	3.14294500	3.93663600
C	7.30215800	1.77459600	4.04902600
C	8.38715900	0.95706100	4.34101300
C	9.66060000	1.49804200	4.47255900
C	9.84402600	2.86305600	4.33475600
O	8.33039500	-0.41845000	4.43778100
C	7.16001000	-1.11230000	4.54945000
C	6.06659500	-0.69596400	5.31688700
C	4.89947700	-1.42403300	5.28671800
C	4.78069300	-2.60098500	4.50023700
C	5.97315200	-3.08730300	3.91035900
C	7.13052900	-2.34887600	3.90974000
C	3.59364500	-3.29941400	4.20605900
C	2.29648000	-2.71824100	4.17397000
C	1.17750100	-3.58358400	4.09850300
C	-0.10271500	-3.08912600	3.92342300
C	-0.32615600	-1.72623700	3.85106600
C	0.74534700	-0.80357500	3.97344000
C	2.04445300	-1.33615600	4.06362700
C	0.61925500	0.61049300	3.95586600
C	-0.56606900	1.29659900	4.29731100
C	-1.65584600	0.78373300	5.04974500
C	-2.86265800	1.44277400	5.10943600
C	-3.02716900	2.64419700	4.41035000
C	-1.92589600	3.26167000	3.82090400
C	-0.71878900	2.60910900	3.78888700
O	-4.20056400	3.33258200	4.32113300
C	-5.43775800	2.72935000	4.21137400
C	-5.64345600	1.39303900	3.89229600
C	-6.94230300	0.92224900	3.76588200
C	-8.02735000	1.76882500	3.92607300
C	-7.80650100	3.10999900	4.20670400
C	-6.51791600	3.59209900	4.35703500
H	8.90697800	4.76002500	3.96458000
H	6.65546800	3.77626900	3.70476900
H	6.32246500	1.35892000	3.87899800
H	10.49264700	0.83576100	4.66490300

H	10.84049100	3.27500100	4.41965800
H	6.13557800	0.20335500	5.91061000
H	4.05586600	-1.09280800	5.87444000
H	5.92738500	-4.03595400	3.39901100
H	8.01850500	-2.69365200	3.40253100
H	1.36193100	-4.64841100	4.12865300
H	-0.93247500	-3.77481000	3.81220800
H	-1.32033900	-1.35599200	3.65150600
H	2.86410000	-0.63635300	4.01659500
H	-1.53583100	-0.14479200	5.58888000
H	-3.68297500	1.03381600	5.68005900
H	-2.05410900	4.23357100	3.36951100
H	0.13587800	3.06029300	3.31089100
H	-4.81237600	0.73261900	3.70745800
H	-7.10080300	-0.11649900	3.51146600
H	-9.03305100	1.39390100	3.79979500
H	-8.64163300	3.79060400	4.30185900
H	-6.32870400	4.63486600	4.56824700

(2) Biscarbene-Me/Graphene

C	6.12240000	-10.15026000	0.41537400
C	4.74252900	-10.51034600	0.44898700
C	2.36092800	-9.85547800	0.44866300
C	-0.02067700	-9.20060800	0.44833600
C	-2.40228300	-8.54573700	0.44801100
C	-4.78388800	-7.89086600	0.44768600
C	3.74079100	-9.49537300	0.41504800
C	1.35918200	-8.84050500	0.41472400
C	-1.02242300	-8.18563300	0.41439800
C	-3.40402800	-7.53076300	0.41407300
C	-5.78563300	-6.87589200	0.41374800
C	7.88033500	-8.41516900	0.41606000
C	9.63827300	-6.68007400	0.41674800
C	11.39621100	-4.94497900	0.41743600
C	13.15414800	-3.20988400	0.41811900
C	14.91208600	-1.47478900	0.41881700
C	16.67001900	0.26030600	0.41942300
C	6.50046300	-8.77523500	0.44967500
C	8.25840300	-7.04014000	0.45036100
C	10.01634000	-5.30504500	0.45104900
C	11.77427800	-3.56994900	0.45173500
C	13.53221500	-1.83485400	0.45242600
C	15.29014900	-0.09975200	0.45309300
C	17.04809400	1.63532100	0.45382300
C	4.12329100	-8.10900600	0.42200200
C	5.87479300	-6.37745600	0.45514500
C	7.63029200	-4.64617900	0.45807000
C	9.38698800	-2.91390800	0.44086600
C	11.14316500	-1.18039200	0.46725600
C	12.90153300	0.55248900	0.47243200
C	14.66649000	2.29019000	0.45349600
C	1.74123000	-7.45486700	0.39251700
C	3.49438500	-5.72162800	0.39787200
C	5.24976100	-3.98938100	0.42280600
C	7.00434800	-2.25839600	0.42621500
C	8.76061800	-0.52682500	0.46282800
C	10.52149100	1.20769200	0.47470700
C	12.28488300	2.94505900	0.45313700
C	-0.63978100	-6.80082300	0.38094500
C	1.11508900	-5.06748600	0.34148600
C	2.86894100	-3.33433700	0.36130400
C	4.62457900	-1.60424700	0.40578200
C	6.38153900	0.12737200	0.45356200
C	8.13996200	1.86371900	0.48341200
C	9.90327800	3.59993000	0.45282300

C	-3.01984000	-6.14542200	0.38934600
C	-1.26471800	-4.41317000	0.33956300
C	0.48940800	-2.68031600	0.31193600
C	2.24455300	-0.95029300	0.38599300
C	4.00090400	0.78294900	0.43200700
C	5.75811400	2.51697900	0.46915900
C	7.52167300	4.25480000	0.45249700
C	5.48856300	-7.75283600	0.43936600
C	7.24658300	-6.01837200	0.45490200
C	9.00349100	-4.28515400	0.46487000
C	10.76024400	-2.55137700	0.46440200
C	12.52009400	-0.81684300	0.47871700
C	14.28345900	0.91327100	0.47079300
C	16.04634600	2.65029700	0.41990300
C	3.11344200	-7.09866200	0.39106800
C	4.86880000	-5.36380700	0.41636700
C	6.62410800	-3.63157800	0.42803600
C	8.38075900	-1.89949400	0.44849400
C	10.13955600	-0.16754700	0.48437600
C	11.89861900	1.56227800	0.48092700
C	13.66474300	3.30515900	0.41946800
C	0.73429700	-6.44622700	0.35777900
C	2.48972700	-4.70986500	0.34873000
C	4.24526500	-2.97711800	0.38685100
C	6.00225400	-1.24619500	0.40647400
C	7.75825700	0.48573200	0.45675900
C	9.51769400	2.21806100	0.47004000
C	11.28313900	3.96003400	0.41920900
C	-1.64617000	-5.79204500	0.35784400
C	0.11082900	-4.05562900	0.30817400
C	1.86673500	-2.32320300	0.33130600
C	3.62109800	-0.59268500	0.40675400
C	5.37760100	1.13999800	0.45616900
C	7.13756100	2.87151200	0.46507200
C	8.90153400	4.61490600	0.41888200
C	-4.02543400	-5.13672100	0.37234400
C	-2.26752900	-3.40157400	0.33608200
C	-0.51263200	-1.66964300	0.32320700
C	1.24097700	0.06249900	0.38333300
C	2.99765500	1.79385900	0.44994300
C	4.75529100	3.52388300	0.46905400
C	6.51992800	5.26977600	0.41855800
C	-7.16549300	-7.23599500	0.44736100
C	-8.16723900	-6.22102100	0.41342200
C	-9.54709900	-6.58112500	0.44703600
C	-11.92870500	-5.92625500	0.44671000
C	-14.31031000	-5.27138400	0.44638600
C	-16.69190800	-4.61651000	0.44606000
C	-10.54884400	-5.56615000	0.41309700
C	-12.93044900	-4.91127900	0.41277200
C	-15.31205600	-4.25640500	0.41244700
C	-17.69366500	-3.60155000	0.41212100
C	-5.40141000	-5.49009300	0.38487600
C	-3.64505500	-3.75977600	0.36633000
C	-1.88766400	-2.02757100	0.34876300
C	-0.13441400	-0.29506900	0.36593100
C	1.62026200	1.43731300	0.40437200
C	3.37878500	3.17075200	0.45190000
C	5.14006800	4.90967200	0.45217200
C	-6.40460900	-4.48285800	0.38743200
C	-4.64736600	-2.74874300	0.37126700
C	-2.89059300	-1.01530900	0.37063100
C	-1.13592700	0.71699900	0.36915900
C	0.61828100	2.44812500	0.41360200
C	2.37317400	4.17904900	0.45918100

C	4.13832300	5.92464600	0.41823300
C	-7.78375800	-4.83733800	0.37941100
C	-6.02455100	-3.10604100	0.35646700
C	-4.26648100	-1.37326000	0.36408500
C	-2.51188000	0.35857500	0.38366000
C	-0.75766900	2.09036500	0.41074700
C	0.99923000	3.82550500	0.44146400
C	2.75846300	5.56454300	0.45184600
C	-10.16333900	-4.18461700	0.37209200
C	-8.40446400	-2.45202400	0.34384100
C	-6.64812100	-0.72052400	0.35422000
C	-4.89115100	1.01176700	0.37895100
C	-3.13477700	2.74494300	0.40971900
C	-1.38071400	4.47951100	0.45683500
C	0.37685700	6.21941300	0.45152100
C	-12.54349100	-3.52862000	0.38784100
C	-10.78440100	-1.79927900	0.37073000
C	-9.02720200	-0.06795300	0.36184900
C	-7.26951800	1.66557300	0.35477600
C	-5.51332400	3.39858900	0.36592300
C	-3.75874900	5.13266300	0.42915500
C	-2.00474800	6.87428400	0.45119600
C	-14.92878100	-2.87941700	0.39708000
C	-13.16508600	-1.14959400	0.38917100
C	-11.40576800	0.58451800	0.39168100
C	-9.64928800	2.31893500	0.38324100
C	-7.89224700	4.05297000	0.37010700
C	-6.13394700	5.78686200	0.40271600
C	-4.38635600	7.52915300	0.45087100
C	-17.31558300	-2.22652400	0.44642200
C	-15.55764900	-0.49143400	0.44711000
C	-13.79971300	1.24366400	0.44779600
C	-12.04177600	2.97875900	0.44848400
C	-10.28383900	4.71385400	0.44917100
C	-8.52590100	6.44894900	0.44985900
C	-6.76796500	8.18403900	0.45054500
C	-8.78601600	-3.82950100	0.37073200
C	-7.02864800	-2.09468800	0.35227900
C	-5.27046200	-0.36173700	0.38064600
C	-3.51421400	1.37015200	0.39973000
C	-1.76117200	3.10221400	0.41524100
C	-0.00686900	4.83437700	0.44897100
C	1.75671700	6.57951700	0.41790800
C	-11.16632300	-3.17418800	0.38600800
C	-9.40645600	-1.44037400	0.36533800
C	-7.65053100	0.29211800	0.36725900
C	-5.89527300	2.02443800	0.36992100
C	-4.14043100	3.75660600	0.41542700
C	-2.38694300	5.48849500	0.45595300
C	-0.62488800	7.23438800	0.41758300
C	-13.54688300	-2.51872000	0.39955000
C	-11.78773900	-0.78676100	0.39581800
C	-10.03258600	0.94677100	0.39136800
C	-8.27666900	2.68050600	0.37291200
C	-6.52020500	4.41201300	0.37925600
C	-4.76823100	6.14249100	0.44108700
C	-3.00649400	7.88925800	0.41725700
C	-15.93571800	-1.86646700	0.41280900
C	-14.17778100	-0.13136500	0.41349600
C	-12.41984300	1.60372900	0.41418300
C	-10.66190600	3.33882400	0.41487100
C	-8.90396900	5.07392000	0.41555800
C	-7.14602800	6.80901400	0.41624400
C	-5.38809500	8.54412500	0.41693200
H	6.88712700	-10.91814700	0.41554000

H	4.45050800	-11.55396800	0.44855000
H	2.07458300	-10.90060600	0.45601900
H	-0.30749900	-10.24550100	0.45504400
H	-2.68922900	-9.59055600	0.45497000
H	-5.07101500	-8.93565800	0.45522600
H	8.64075000	-9.18727300	0.40833700
H	10.39909400	-7.45169400	0.40937800
H	12.15734100	-5.71617000	0.40997000
H	13.91506700	-3.98115100	0.41149600
H	15.67221900	-2.24698600	0.41101500
H	17.42753100	-0.51489900	0.41400700
H	18.09661500	1.90927700	0.44814800
H	13.95078500	4.35060400	0.40973500
H	11.56920500	5.00510400	0.41249800
H	9.18860400	5.65956500	0.41277100
H	6.80752700	6.31437700	0.41160100
H	-7.45318600	-8.28060000	0.45555600
H	-9.83514900	-7.62543100	0.45476300
H	-12.21606100	-6.97056900	0.45304400
H	-14.59633900	-6.31617600	0.45361400
H	-16.97186200	-5.66331000	0.44845700
H	-18.74275200	-3.87435500	0.41565400
H	4.42568000	6.96943100	0.41063600
H	-18.07194600	-1.45047800	0.44579300
H	-16.31848200	0.28037800	0.45772000
H	-14.56018100	2.01542200	0.45515300
H	-12.80266900	3.75002200	0.45544300
H	-11.04474600	5.48525100	0.45632900
H	-9.28639000	7.22092700	0.45761000
H	-7.53263500	8.95195000	0.45043300
H	2.04360300	7.62445600	0.41044400
H	-0.33830400	8.27940100	0.41061800
H	-2.72028500	8.93442600	0.40988200
H	-5.09610400	9.58774600	0.41738800
H	16.32918900	3.69664200	0.41657800
C	-9.46905000	-2.93943700	3.77527100
C	-8.15237400	-2.56222800	3.53632100
C	-7.74629600	-1.23693600	3.58818800
C	-8.67573800	-0.25618000	3.90239500
C	-10.00493500	-0.60164900	4.11261700
C	-10.39033700	-1.92742100	4.04165500
O	-8.41306300	1.09960800	3.93130300
C	-7.16672600	1.62978300	4.07917300
C	-6.12425300	1.03700800	4.80063200
C	-4.88174300	1.63022300	4.81012200
C	-4.62687000	2.83727600	4.10584500
C	-5.75534900	3.48776700	3.54811400
C	-6.99132500	2.89403500	3.51830200
C	-3.37158600	3.43124200	3.87380800
C	-2.11428100	2.77078000	3.87523700
C	-0.94685200	3.57622600	3.86351200
C	0.30751300	3.01917800	3.70650500
C	0.46043000	1.64823900	3.59137200
C	-0.66162200	0.78226300	3.64361100
C	-1.93447100	1.38230000	3.72487200
C	-0.61910100	-0.63173000	3.54728500
C	0.48407000	-1.43919900	3.90054800
C	1.62241600	-1.04133300	4.65075100
C	2.74694000	-1.83092700	4.73194900
C	2.77073300	-3.06595500	4.07353000
C	1.60684100	-3.56388400	3.48955600
C	0.49031700	-2.76980000	3.41704400
O	3.84968700	-3.89366400	4.01680900
C	5.16008500	-3.45273000	4.00145600
C	5.56399300	-2.17705500	3.63668900

C	6.91888000	-1.88241600	3.59229800
C	7.88993500	-2.83302400	3.88370900
C	7.45508500	-4.11655300	4.21298700
C	6.10990900	-4.42758400	4.28070700
H	-7.42130600	-3.31821100	3.28003000
H	-6.72718000	-0.98005300	3.34895800
H	-10.72301200	0.18015100	4.31535200
H	-11.43114800	-2.18112000	4.19926600
H	-6.29210200	0.11598200	5.33820600
H	-4.08397000	1.16652200	5.37147500
H	-5.60126500	4.45195100	3.08865200
H	-7.83258700	3.36886900	3.03779300
H	-1.07607400	4.64789100	3.92151200
H	1.17619700	3.66119300	3.63926200
H	1.43985200	1.23520600	3.40432600
H	-2.78821400	0.73091000	3.62281500
H	1.60607200	-0.09702900	5.17487100
H	3.60642700	-1.50384700	5.29752500
H	1.62129500	-4.55527100	3.06291300
H	-0.40521300	-3.12772400	2.93391300
H	4.84467100	-1.42742000	3.35009300
H	7.22132400	-0.88700300	3.29434200
H	8.18353400	-4.89118000	4.41750100
H	5.77877600	-5.42623300	4.52771700
C	-9.88158700	-4.37777500	3.76503600
H	-9.18470600	-4.99025400	3.19866500
H	-9.91957300	-4.78014600	4.77932800
H	-10.87001500	-4.50922900	3.32963900
C	9.34752500	-2.49446900	3.85187700
H	9.73682600	-2.33555600	4.85913400
H	9.93280600	-3.29440100	3.40226300
H	9.53379900	-1.58757600	3.28235300

(3) Biscarbene-NO_2/Graphene

C	6.49777100	-10.28462400	-0.36683900
C	5.13311300	-10.69622700	-0.31093500
C	2.72964800	-10.13029100	-0.24806800
C	0.32618000	-9.56435400	-0.18520500
C	-2.07728900	-8.99841600	-0.12233900
C	-4.48075800	-8.43247700	-0.05947400
C	4.09429800	-9.71867000	-0.30397400
C	1.69082600	-9.15273500	-0.24110800
C	-0.71264200	-8.58679500	-0.17824300
C	-3.11611100	-8.02085800	-0.11537800
C	-5.51957900	-7.45491900	-0.05251200
C	8.19044000	-8.48580700	-0.36988000
C	9.88311100	-6.68698700	-0.37291800
C	11.57578200	-4.88816600	-0.37595700
C	13.26845200	-3.08934500	-0.37900000
C	14.96112300	-1.29052400	-0.38202900
C	16.65378800	0.50829800	-0.38514900
C	6.82577900	-8.89739000	-0.31397300
C	8.51845200	-7.09856900	-0.31701300
C	10.21112200	-5.29974800	-0.32005200
C	11.90379300	-3.50092700	-0.32309200
C	13.59646400	-1.70210600	-0.32612800
C	15.28913000	0.09672200	-0.32918700
C	16.98181000	1.89552000	-0.33218400
C	4.42582100	-8.31939700	-0.28298600
C	6.11380300	-6.52533500	-0.26692100
C	7.80397700	-4.73096800	-0.24634500
C	9.49508500	-2.93518500	-0.22442500
C	11.18653600	-1.13797400	-0.21944200
C	12.87989900	0.65979400	-0.23119600
C	14.57834300	2.46145700	-0.26932100

C	2.02172900	-7.75382600	-0.23969600
C	3.71051300	-5.95748100	-0.24677300
C	5.40123800	-4.16236100	-0.22794000
C	7.09113900	-2.36860900	-0.18014500
C	8.78276300	-0.57313900	-0.15216400
C	10.47694900	1.22558400	-0.15898400
C	12.17487200	3.02739400	-0.20648900
C	-0.38123200	-7.18823600	-0.18269700
C	1.30856900	-5.39197400	-0.20702800
C	2.99830200	-3.59623000	-0.18859900
C	4.68957700	-1.80202000	-0.14611700
C	6.38046400	-0.00658900	-0.12029300
C	8.07375900	1.79158900	-0.10506500
C	9.77140300	3.59333200	-0.14361300
C	-2.78396600	-6.62168400	-0.12291800
C	-1.09517600	-4.82626900	-0.16924600
C	0.59649700	-3.03068600	-0.16765500
C	2.28764100	-1.23476000	-0.12022500
C	3.97852200	0.56114900	-0.09013700
C	5.67009400	2.35766000	-0.07221900
C	7.36793500	4.15927000	-0.08074800
C	5.77769000	-7.91298100	-0.29034700
C	7.47038800	-6.11594600	-0.27948500
C	9.16186800	-4.31921400	-0.26568800
C	10.85411700	-2.52133600	-0.25863800
C	12.54844800	-0.72339100	-0.26306600
C	14.24606600	1.07161100	-0.27111200
C	15.94298600	2.87308000	-0.32520500
C	3.37920700	-7.34712300	-0.27275900
C	5.07040500	-5.54936100	-0.26628700
C	6.76197600	-3.75379600	-0.23247300
C	8.45317200	-1.95882300	-0.19192800
C	10.14627700	-0.16299000	-0.18231400
C	11.84066600	1.63163200	-0.20326400
C	13.53951800	3.43901100	-0.26245000
C	0.97773500	-6.78308800	-0.22240700
C	2.66844000	-4.98338400	-0.22763000
C	4.36071400	-3.18743500	-0.20039800
C	6.05225300	-1.39373700	-0.15504700
C	7.74325000	0.40130800	-0.13319900
C	9.43793000	2.19670200	-0.13764400
C	11.13605200	4.00495200	-0.19951800
C	-1.42471400	-6.21769600	-0.16817700
C	0.26738800	-4.41871500	-0.20134600
C	1.95947600	-2.62202800	-0.16559400
C	3.65034000	-0.82728100	-0.12190400
C	5.34103600	0.96789800	-0.11088100
C	7.03486600	2.76177400	-0.09008800
C	8.73258300	4.57089200	-0.13665400
C	-3.82547300	-5.65143900	-0.10416400
C	-2.13426300	-3.85316900	-0.15293800
C	-0.44379000	-2.05572500	-0.14990200
C	1.24820300	-0.25962400	-0.10686600
C	2.93959700	1.53456800	-0.07300400
C	4.63115900	3.32709100	-0.03874000
C	6.32911400	5.13682900	-0.07378800
C	-6.88422600	-7.86653900	0.00339200
C	-7.92304800	-6.88898100	0.01035200
C	-9.28769500	-7.30060100	0.06625800
C	-11.69116500	-6.73466400	0.12912200
C	-14.09463300	-6.16872600	0.19198900
C	-16.49809500	-5.60278400	0.25485300
C	-10.32651700	-6.32304300	0.07321800
C	-12.72998500	-5.75710400	0.13608300
C	-15.13345500	-5.19116300	0.19894900

C	-17.53692700	-4.62524100	0.26181300
C	-5.18728500	-6.05580800	-0.05666100
C	-3.49627800	-4.26145900	-0.09773500
C	-1.80573800	-2.46506700	-0.13546700
C	-0.11417400	-0.66805600	-0.09658500
C	1.57681800	1.12745800	-0.06268000
C	3.26926600	2.92334500	-0.02077900
C	4.96446700	4.72520900	-0.01788300
C	-6.22630100	-5.08712500	-0.03477300
C	-4.53553300	-3.28865700	-0.06252100
C	-2.84562600	-1.49048400	-0.09309500
C	-1.15296900	0.30556900	-0.05720100
C	0.53768400	2.09966900	-0.02273300
C	2.22867000	3.89278900	0.01756800
C	3.92564500	5.70276700	-0.01092300
C	-7.59080500	-5.49267800	0.00823800
C	-5.89680100	-3.69721500	-0.01677400
C	-4.20657300	-1.90000400	-0.04534600
C	-2.51586700	-0.10418900	-0.03819300
C	-0.82326900	1.69170000	-0.00097600
C	0.86811200	3.48865400	0.04058900
C	2.56099800	5.29114700	0.04498200
C	-9.99237500	-4.92775400	0.07080200
C	-8.29880000	-3.13164000	0.05061100
C	-6.60741500	-1.33597300	0.03719000
C	-4.91614900	0.45975900	0.01641400
C	-3.22475200	2.25546300	0.05042700
C	-1.53364100	4.05313100	0.10944700
C	0.15752900	5.85708500	0.10784800
C	-12.39528400	-4.36046700	0.13554100
C	-10.70200700	-2.56768200	0.11753900
C	-9.00844900	-0.77210100	0.10932500
C	-7.31626800	1.02536400	0.09339100
C	-5.62613400	2.82091600	0.09577500
C	-3.93434600	4.61710400	0.15589300
C	-2.24593900	6.42302300	0.17071300
C	-14.80197900	-3.80123900	0.20177900
C	-13.10305400	-2.00735800	0.19518200
C	-11.40957400	-0.20851500	0.18929500
C	-9.71838100	1.58934600	0.18563700
C	-8.02545900	3.38626700	0.18179900
C	-6.33142000	5.18385100	0.20083300
C	-4.64941100	6.98895900	0.23357900
C	-17.20890100	-3.23800500	0.31468000
C	-15.51623300	-1.43918900	0.31164100
C	-13.82356400	0.35963400	0.30860100
C	-12.13089400	2.15845500	0.30556200
C	-10.43822400	3.95727600	0.30252300
C	-8.74555300	5.75609700	0.29948400
C	-7.05288400	7.55491300	0.29644400
C	-8.62984700	-4.52280300	0.02994100
C	-6.93692200	-2.72316000	0.01262000
C	-5.24492800	-0.92611400	-0.00678500
C	-3.55516700	0.86901500	-0.00037700
C	-1.86431600	2.66383000	0.04489500
C	-0.17325400	4.45847600	0.08696000
C	1.52217600	6.26870500	0.05194300
C	-11.03289800	-3.95530800	0.09358000
C	-9.33764800	-2.15770000	0.08043800
C	-7.64681500	-0.36181100	0.07016100
C	-5.95687700	1.43451700	0.04796600
C	-4.26541400	3.22971100	0.08198400
C	-2.57610500	5.02366300	0.14850700
C	-0.88129200	6.83464300	0.11480900
C	-13.43426000	-3.38907000	0.16340300

C	-11.74188400	-1.59288400	0.15352900
C	-10.05164100	0.20500200	0.14724800
C	-8.35945300	2.00206100	0.14028800
C	-6.66800200	3.79616500	0.14640700
C	-4.98111600	5.58937200	0.19369800
C	-3.28476200	7.40058100	0.17767300
C	-15.84424700	-2.82643100	0.25877400
C	-14.15157700	-1.02760400	0.25573500
C	-12.45890600	0.77121600	0.25269500
C	-10.76623600	2.57003700	0.24965700
C	-9.07356500	4.36885900	0.24661700
C	-7.38089100	6.16767900	0.24357700
C	-5.68822600	7.96651500	0.24053900
H	7.28998300	-11.02347600	-0.39775100
H	4.87964800	-11.74977800	-0.32536500
H	2.48208100	-11.18528200	-0.25485900
H	0.07811300	-10.61911500	-0.19262800
H	-2.32547700	-10.05311200	-0.12950800
H	-4.72911500	-9.48716400	-0.06605700
H	8.97832500	-9.22888600	-0.40866700
H	10.67139200	-7.42957400	-0.41135200
H	12.36435400	-5.63031100	-0.41448500
H	14.05683500	-3.83158900	-0.41668300
H	15.74872900	-2.03370500	-0.42089100
H	17.43893700	-0.23803000	-0.42162900
H	18.01919500	2.20825400	-0.35480000
H	13.78671900	4.49435100	-0.25802200
H	11.38335700	5.05986200	-0.19207800
H	8.98091900	5.62541700	-0.12864400
H	6.57796200	6.19133200	-0.06663500
H	-7.13314100	-8.92107200	-0.00252100
H	-9.53698800	-8.35484000	0.05989000
H	-11.93979500	-7.78885900	0.12134700
H	-14.34190000	-7.22336600	0.18507000
H	-16.73932400	-6.65911600	0.24293700
H	-18.57496700	-4.93680500	0.28232200
H	4.17423100	6.75745600	-0.00440100
H	-17.99306600	-2.49068200	0.34510900
H	-16.30446300	-0.69647100	0.35331700
H	-14.61149800	1.10237300	0.34701600
H	-12.91924300	2.90069100	0.34357900
H	-11.22658800	4.69964100	0.34074200
H	-9.53350700	6.49904700	0.33829800
H	-7.84503800	8.29379000	0.32740800
H	1.77028900	7.32352800	0.05861100
H	-0.63347300	7.88952000	0.12198300
H	-3.03733200	8.45560600	0.18444900
H	-5.43479100	9.02006400	0.25498900
H	16.18709800	3.92908100	-0.31428700
C	4.21922000	-4.57003900	3.09897400
C	3.37991600	-3.50992300	3.40252500
C	2.03857500	-3.74406800	3.63552200
C	1.54207000	-5.04349200	3.53749100
C	2.38691400	-6.09770300	3.19263100
C	3.72873800	-5.86424700	2.98697700
O	0.25858200	-5.40110500	3.83924200
C	-0.75244700	-4.47677200	3.96919800
C	-1.62748800	-4.65372000	5.03971900
C	-2.71372800	-3.82004600	5.17425100
C	-2.93764400	-2.75019600	4.27425500
C	-2.03498400	-2.62067400	3.18903000
C	-0.96825800	-3.47320200	3.03119300
C	-4.04625900	-1.89973600	4.44714200
C	-4.09200800	-0.56711300	3.99619600
C	-5.32581100	0.02644600	3.62507200

C	-5.37984600	1.35425300	3.24562200
C	-4.23816200	2.13521300	3.21882200
C	-2.97531300	1.59021000	3.56680400
C	-2.93565000	0.24362200	3.98661500
C	-1.81769200	2.35429700	3.45527000
C	-0.43197300	2.23621300	3.39734700
C	0.23099300	1.00797100	3.13722500
C	1.60406800	0.93316500	3.08390400
C	2.36984700	2.07883100	3.28312000
C	1.75277100	3.30713000	3.49292900
C	0.38061800	3.38638000	3.54906200
O	3.74366800	2.09079500	3.16930900
C	4.52365900	1.11790200	3.72509600
C	4.10984200	0.28804200	4.76601200
C	4.99324800	-0.63795200	5.28773500
C	6.27951900	-0.72491600	4.77909600
C	6.70392300	0.11373800	3.75846300
C	5.82539300	1.03627000	3.23421400
H	3.78903400	-2.51542200	3.48125800
H	1.39167200	-2.92824000	3.91560000
H	1.97645800	-7.09248100	3.10468900
H	4.40129000	-6.66930500	2.73874900
H	-1.44415500	-5.45782800	5.73809500
H	-3.40965700	-3.95406400	5.99007800
H	-2.21429100	-1.85423100	2.45257600
H	-0.31073500	-3.37415800	2.18376700
H	-6.21681000	-0.58415400	3.64658800
H	-6.32747900	1.79318500	2.96557600
H	-4.28934800	3.17197400	2.92163000
H	-2.00613600	-0.17524300	4.33688800
H	-0.34932900	0.12112100	2.93588700
H	2.08530700	-0.00516900	2.84975000
H	2.36619400	4.19011900	3.60162400
H	-0.09858400	4.34163800	3.70869700
H	3.11531600	0.37630300	5.17433100
H	4.69774900	-1.29101800	6.09379800
H	7.70945500	0.02605700	3.37893100
H	6.12870900	1.70326600	2.44388700
N	5.64533600	-4.32242800	2.91302500
O	6.39036600	-5.29462500	2.80825500
O	6.02080300	-3.15007100	2.87097500
N	7.20451500	-1.71547900	5.33140000
O	6.75405800	-2.53102700	6.13682300
O	8.37608100	-1.67143500	4.96476700

(4) Biscarbene-NH_2/Graphene

C	6.44518200	-9.16715300	-0.01757900
C	5.06531100	-9.52723900	0.01603400
C	2.68371000	-8.87237100	0.01571000
C	0.30210500	-8.21750100	0.01538300
C	-2.07950100	-7.56263000	0.01505800
C	-4.46110600	-6.90775900	0.01473300
C	4.06357300	-8.51226600	-0.01790500
C	1.68196400	-7.85739800	-0.01822900
C	-0.69964100	-7.20252600	-0.01855500
C	-3.08124600	-6.54765600	-0.01888000
C	-5.46285100	-5.89278500	-0.01920500
C	8.20311700	-7.43206200	-0.01689300
C	9.96105500	-5.69696700	-0.01620500
C	11.71899300	-3.96187200	-0.01551700
C	13.47693000	-2.22677700	-0.01483400
C	15.23486800	-0.49168200	-0.01413600
C	16.99280100	1.24341300	-0.01353000
C	6.82324500	-7.79212800	0.01672200
C	8.58118500	-6.05703300	0.01740800

C	10.33912200	-4.32193800	0.01809600
C	12.09706000	-2.58684200	0.01878200
C	13.85499700	-0.85174700	0.01947300
C	15.61293100	0.88335500	0.02014000
C	17.37087600	2.61842800	0.02087000
C	4.44586100	-7.12531500	-0.00852100
C	6.19797800	-5.39473300	0.00127500
C	7.95426000	-3.66369500	0.02140400
C	9.71076400	-1.93004400	0.04966100
C	11.46502600	-0.19648500	0.05611100
C	13.22441100	1.53499900	0.05586600
C	14.98927200	3.27329700	0.02054300
C	2.06331700	-6.47139100	-0.02704200
C	3.81796300	-4.73911700	-0.00749500
C	5.57368900	-3.00705500	0.01238200
C	7.32840500	-1.27518400	0.03479000
C	9.08411500	0.45672900	0.03133000
C	10.84445800	2.19021600	0.04363600
C	12.60766500	3.92816600	0.02018400
C	-0.31675900	-5.81803600	-0.04183600
C	1.43834900	-4.08538700	-0.03002700
C	3.19194300	-2.35367100	0.01216000
C	4.94824200	-0.62178300	0.04173000
C	6.70669000	1.11144800	0.02705000
C	8.46359400	2.84596200	0.02564200
C	10.22606000	4.58303700	0.01987000
C	-2.69621400	-5.16227300	-0.03912200
C	-0.94122700	-3.43134100	-0.05640000
C	0.81253300	-1.70072500	-0.02634600
C	2.56827500	0.03151800	0.02511700
C	4.32489900	1.76550700	0.04702300
C	6.08146700	3.49988900	0.04701900
C	7.84445500	5.23790700	0.01954400
C	5.81172000	-6.76926900	-0.00074200
C	7.57004500	-5.03543800	0.00343600
C	9.32680400	-3.30236200	0.02754600
C	11.08248400	-1.56789700	0.03860400
C	12.84188200	0.16667200	0.04034700
C	14.60724700	1.89550000	0.03812300
C	16.36912800	3.63340400	-0.01305000
C	3.43678800	-6.11526700	-0.02530400
C	5.19235600	-4.38098900	-0.01777700
C	6.94747500	-2.64848600	0.01120400
C	8.70504100	-0.91495900	0.03009200
C	10.46207600	0.81545500	0.03693500
C	12.22090500	2.54414300	0.03825500
C	13.98752500	4.28826600	-0.01348500
C	1.05802200	-5.46328500	-0.04952000
C	2.81212900	-3.72777900	-0.01899600
C	4.56887500	-1.99501700	0.02050200
C	6.32625100	-0.26290400	0.02325700
C	8.08232500	1.46792800	0.00858000
C	9.84096200	3.20065200	0.01666000
C	11.60592100	4.94314100	-0.01374400
C	-1.32211000	-4.80925900	-0.06287500
C	0.43451200	-3.07427200	-0.05159600
C	2.18953300	-1.34257300	-0.00523800
C	3.94467400	0.38977200	0.03257200
C	5.70220500	2.12282700	0.03206200
C	7.46128400	3.85404700	0.02575900
C	9.22431600	5.59801300	-0.01407100
C	-3.70178800	-4.15387400	-0.05580200
C	-1.94330200	-2.42035800	-0.06924800
C	-0.18909900	-0.68905900	-0.05086700
C	1.56500200	1.04411600	-0.00304000

C	3.32241800	2.77662500	0.03405500
C	5.07869000	4.50673000	0.03599100
C	6.84271000	6.25288300	-0.01439500
C	-6.84271100	-6.25288800	0.01440800
C	-7.84445700	-5.23791400	-0.01953100
C	-9.22431700	-5.59801800	0.01408300
C	-11.60592300	-4.94314800	0.01375700
C	-13.98752800	-4.28827700	0.01343300
C	-16.36912600	-3.63340300	0.01310700
C	-10.22606200	-4.58304300	-0.01985600
C	-12.60766700	-3.92817200	-0.02018100
C	-14.98927400	-3.27329800	-0.02050600
C	-17.37088300	-2.61844300	-0.02083200
C	-5.07853900	-4.50675000	-0.03863300
C	-3.32135000	-2.77714500	-0.05778400
C	-1.56496500	-1.04577000	-0.05713100
C	0.18892900	0.68640400	-0.03656600
C	1.94407900	2.41888400	0.02339300
C	3.70229600	4.15358200	0.04766300
C	5.46285000	5.89277900	0.01921900
C	-6.08118900	-3.50020500	-0.06399700
C	-4.32423800	-1.76606500	-0.08054700
C	-2.56807700	-0.03343000	-0.08518600
C	-0.81272200	1.69800300	-0.07610200
C	0.94190900	3.43002600	0.00224100
C	2.69708700	5.16218400	0.02962200
C	4.46110500	6.90775300	-0.01472000
C	-7.46078500	-3.85478300	-0.04590100
C	-5.70120800	-2.12290700	-0.07328900
C	-3.94457000	-0.39026200	-0.08383700
C	-2.18988900	1.34033400	-0.08849200
C	-0.43420700	3.07192100	-0.02901300
C	1.32275900	4.80869400	0.03308200
C	3.08124500	6.54765000	0.01889300
C	-9.84066400	-3.20141100	-0.04707500
C	-8.08132700	-1.46840800	-0.08762700
C	-6.32569000	0.26385000	-0.12050800
C	-4.56859700	1.99394000	-0.09161200
C	-2.81272900	3.72612200	-0.04622900
C	-1.05805100	5.46247600	0.01836700
C	0.69963900	7.20252000	0.01856800
C	-12.22155500	-2.54446700	-0.04186200
C	-10.46272400	-0.81560900	-0.07532000
C	-8.70387100	0.91680300	-0.11588000
C	-6.94750100	2.64842200	-0.09100500
C	-5.19254000	4.38011300	-0.03951800
C	-3.43686900	6.11459700	0.01177400
C	-1.68196600	7.85739100	0.01824300
C	-14.60751300	-1.89588400	-0.03625100
C	-12.84291300	-0.16701900	-0.04412100
C	-11.08281300	1.56869000	-0.06138500
C	-9.32694900	3.30280200	-0.05856600
C	-7.57028200	5.03500600	-0.03729700
C	-5.81134700	6.76925700	-0.01130900
C	-4.06357400	8.51226000	0.01791800
C	-16.99280100	-1.24341700	0.01346900
C	-15.23486700	0.49167300	0.01415700
C	-13.47693100	2.22677100	0.01484300
C	-11.71899400	3.96186600	0.01553100
C	-9.96105700	5.69696100	0.01621800
C	-8.20311900	7.43205600	0.01690600
C	-6.44518300	9.16714600	0.01759200
C	-8.46324100	-2.84711500	-0.07131300
C	-6.70500200	-1.11062500	-0.10871200
C	-4.94783700	0.62129200	-0.11704700

C	-3.19216400	2.35117800	-0.09431800
C	-1.43858600	4.08443400	-0.03050200
C	0.31692200	5.81766600	0.01983300
C	2.07949900	7.56262400	-0.01504500
C	-10.84501500	-2.19137200	-0.06651100
C	-9.08373200	-0.45601000	-0.11056500
C	-7.32781800	1.27592400	-0.13269600
C	-5.57332300	3.00624200	-0.08502200
C	-3.81775900	4.73859800	-0.03457900
C	-2.06397400	6.47137300	0.01512500
C	-0.30210600	8.21749500	-0.01537000
C	-13.22458000	-1.53556000	-0.05388100
C	-11.46693400	0.19682900	-0.07691500
C	-9.71068600	1.93125100	-0.10009800
C	-7.95381700	3.66380300	-0.07406200
C	-6.19771600	5.39430300	-0.04116700
C	-4.44623600	7.12531200	0.00360700
C	-2.68371200	8.87236500	-0.01569600
C	-15.61293600	-0.88336000	-0.02014400
C	-13.85499900	0.85174200	-0.01945700
C	-12.09706100	2.58683600	-0.01877000
C	-10.33912400	4.32193100	-0.01808200
C	-8.58118700	6.05702700	-0.01739500
C	-6.82324600	7.79212100	-0.01670900
C	-5.06531300	9.52723200	-0.01602100
H	7.20990900	-9.93504000	-0.01741300
H	4.77329000	-10.57086100	0.01559700
H	2.39736500	-9.91749900	0.02306600
H	0.01528300	-9.26239400	0.02209100
H	-2.36644700	-8.60744900	0.02201700
H	-4.74823300	-7.95255100	0.02227300
H	8.96353200	-8.20416600	-0.02461600
H	10.72187600	-6.46858700	-0.02357500
H	12.48012300	-4.73306300	-0.02298300
H	14.23784900	-2.99804400	-0.02145700
H	15.99500100	-1.26387900	-0.02193800
H	17.75031300	0.46820800	-0.01894600
H	18.41939700	2.89238400	0.01519500
H	14.27356700	5.33371100	-0.02321800
H	11.89198700	5.98821100	-0.02045500
H	9.51138600	6.64267200	-0.02018200
H	7.13030900	7.29748400	-0.02135200
H	-7.13040400	-7.29749300	0.02260300
H	-9.51236700	-6.64232400	0.02181000
H	-11.89327900	-5.98746200	0.02009100
H	-14.27355700	-5.33306900	0.02066100
H	-16.64908000	-4.68020300	0.01550400
H	-18.41997000	-2.89124800	-0.01729900
H	4.74846200	7.95253800	-0.02231700
H	-17.74916400	-0.46737100	0.01284000
H	-15.99570000	1.26348500	0.02476700
H	-14.23739900	2.99852900	0.02220000
H	-12.47988700	4.73312900	0.02249000
H	-10.72196400	6.46835800	0.02337600
H	-8.96360800	8.20403400	0.02465700
H	-7.20985300	9.93505700	0.01748000
H	2.36638500	8.60756300	-0.02250900
H	-0.01552200	9.26250800	-0.02233500
H	-2.39750300	9.91753300	-0.02307100
H	-4.77332200	10.57085300	-0.01556500
H	16.65197100	4.67974900	-0.01637500
C	9.46693700	1.96226500	3.32397300
C	8.13115800	1.60801200	3.13368300
C	7.71585600	0.28984700	3.19938100
C	8.63153900	-0.71029700	3.49621100

C	9.96823400	-0.37713500	3.67832900
C	10.38087800	0.93755800	3.58705300
O	8.35395400	-2.06553500	3.53402500
C	7.09775800	-2.58100800	3.66142400
C	6.03049700	-1.95316800	4.30773400
C	4.78197200	-2.53373800	4.29473500
C	4.54394900	-3.76400000	3.62817900
C	5.68108800	-4.44688800	3.13036200
C	6.92452900	-3.86773900	3.14809900
C	3.27175500	-4.29118100	3.44275000
C	1.93547700	-3.91267300	3.38341600
C	0.91663600	-4.90067500	3.30612800
C	-0.41107800	-4.53248000	3.20444700
C	-0.79114400	-3.20280200	3.19722700
C	0.18817200	-2.17559500	3.31593300
C	1.54282800	-2.55740000	3.36366700
C	-0.14885500	-0.82705300	3.35174200
C	-1.23395100	0.02401000	3.51614500
C	-2.44502900	-0.38169500	4.13541900
C	-3.55296000	0.43581000	4.15196500
C	-3.49898400	1.69605200	3.55136800
C	-2.27701900	2.17486100	3.07579500
C	-1.17062700	1.36440300	3.06217100
O	-4.54670600	2.56137000	3.43658800
C	-5.87362900	2.17269200	3.39408700
C	-6.33269700	0.90413700	3.06616600
C	-7.69270000	0.65882500	3.00119400
C	-8.62827800	1.66939700	3.22183400
C	-8.14730900	2.94862100	3.51744000
C	-6.79172200	3.19361600	3.60945600
H	7.40384400	2.37286000	2.89465400
H	6.68950200	0.05561300	2.97227400
H	10.68151500	-1.16573400	3.87165100
H	11.42879300	1.17491500	3.71969400
H	6.17894800	-1.01833000	4.82508900
H	3.96756000	-2.03986900	4.80327200
H	5.54935900	-5.42495200	2.69353200
H	7.77912400	-4.37739600	2.73033700
H	1.20144600	-5.94307100	3.30374300
H	-1.16887900	-5.29877700	3.10544200
H	-1.82949700	-2.93552700	3.07527100
H	2.29160400	-1.78117600	3.38175900
H	-2.49941900	-1.35322600	4.60405000
H	-4.45505200	0.10186100	4.63958800
H	-2.23246600	3.18072000	2.68559500
H	-0.23940100	1.73272300	2.66004800
H	-5.64723200	0.11190100	2.81656200
H	-8.03152900	-0.33339000	2.73368700
H	-8.84522900	3.76086600	3.67605400
H	-6.42638300	4.18657400	3.83017700
N	9.86639000	3.29481400	3.29721900
N	-9.99299000	1.40136800	3.19660400
H	-10.24006900	0.59850500	2.64039500
H	-10.56130600	2.18899100	2.92900100
H	10.82632200	3.42867000	3.02167400
H	9.26961900	3.88704800	2.74186800

2.7.5 Electron Spin Resonance (ESR) and Electron Paramagnetic Resonance (EPR) simulation

In ORCA 5.0.3, the following expression/format was added in the very beginning of input file for EPR calculation for D/E tensor and g-tensor, respectively. The calculated numbers summed up in Table 11 and 12 were read out from the output files.

(1) carbene species at triplet state for D/E tensor

```
! UKS B3LYP EPR-II UNO KDIIS
! tightSCF SlowConv
%maxcore 500000
%pal nproc 8 end
%SCF MaxIter 200 end
*xyz 0  3
[molecular coordinates]
*
%EPRNMR
        Dtensor ss      # D TENSOR TRUE calculation at spin-spin component
        DSS uno         # NUCLEI= 14 {AISO, ADIP, AORN} and number 14 is the carbene centered
                        # C atom of interest for EPR calculation
END
```

(2) carbene species at triplet state for carbene centered C atoms of C14 and C21's g-tensor

```
! UKS B3LYP EPR-II UNO KDIIS autoaux RIJCOSX
! tightSCF SlowConv
%maxcore 500000
%pal nproc 8 end
%SCF MaxIter 200 end
*xyz 0  3
[molecular coordinates]
*
%EPRNMR
        GTENSOR TRUE      #  g-tensor calculation
        NUCLEI= 14 {AISO, ADIP, AORN}  # number 14 is the carbene centered
        NUCLEI= 21 {AISO, ADIP, AORN}  # number 21 is the carbene centered C atom of interest
                                        # for EPR calculation
END
```

Reference:

1. Attenburrow, J.; Cameron, A. F. B.; Chapman, J. H.; Evans, R. M.; Hems, B. A.; Jansen, A. B. A.; Walker, T., 194. A synthesis of vitamin a from cyclohexanone. *Journal of the Chemical Society (Resumed)* **1952,** (0), 1094-1111.
2. Yang, P.; Moloney, M. G., Surface modification of polymers with bis(arylcarbene)s from bis(aryldiazomethane)s: preparation, dyeing and characterization. *RSC Advances* **2016,** *6* (112), 111276-111290.
3. Yang, P.; Moloney, M. G., Surface modification using crosslinking of diamine and a bis(diarylcarbene): synthesis, characterization, and antibacterial activity via binding hydrogen peroxide. *RSC Advances* **2017,** *7* (47), 29645-29655.
4. Yang, P.; Wang, Y.; Lu, L.; Yu, X.; Liu, L., Surface hydrophobic modification of polyurethanes by diaryl carbene chemistry: Synthesis and characterization. *Applied Surface Science* **2018,** *435*, 346-351.
5. X Liu, M. G. M., K. Okuda, Polymerization Behavior of Biscarbene Derived by Thermolysis of Bisdiazo Compounds. *Polymer Chemistry* **2025**.DOI: 10.1039/D4PY01474J.
